# Antimicrobial resistance in Africa: A retrospective analysis of data from 14 countries, 2016–2019

**DOI:** 10.1371/journal.pmed.1004638

**Published:** 2025-06-24

**Authors:** Gilbert Osena, Geetanjali Kapoor, Erta Kalanxhi, Timothée Ouassa, Edwin Shumba, Sehr Brar, Yewande Alimi, Manuel Moreira, Martin Matu, Abdourahmane Sow, Eili Klein, Pascale Ondoa, Ramanan Laxminarayan, the MAAP Study Group

**Affiliations:** 1 One Health Trust, Washington DC and Bengaluru, Bengaluru, India; 2 Department of Infectious Diseases, Institute for Biomedicine, University of Gothenburg, Gothenburg, Sweden; 3 African Society for Laboratory Medicine, Addis Ababa, Ethiopia; 4 Africa Centres for Disease Control and Prevention, African Union Commission, Addis Ababa, Ethiopia; 5 Innovative Support to Emergencies, Diseases and Disasters (InSTEDD), Seattle, Washington, United States of America; 6 East, Central and Southern African Health Community, Arusha, Tanzania; 7 West Africa Health Organization, Bobo Diolasso, Burkina Faso; 8 Department of Emergency Medicine, Johns Hopkins University, Baltimore, Maryland, United States of America; 9 Department of Global Health, Amsterdam Institute for Global Health and Development, Amsterdam University Medical Center, Amsterdam, The Netherlands; 10 Princeton University, Princeton, New Jersey, United States of America; University of Glasgow, UNITED KINGDOM OF GREAT BRITAIN AND NORTHERN IRELAND

## Abstract

**Background:**

Antimicrobial resistance (AMR) is a major global health issue that exacerbates the burden of infectious diseases and healthcare costs. However, the scarcity of national-level AMR data in African countries hampers our understanding of its scale and contributing factors in the region. To gain insights into AMR prevalence in Africa, we collected and analyzed retrospective AMR data from 14 countries.

**Methods and findings:**

We estimated bacterial AMR prevalence, defined as the proportion of resistant human isolates tested from antimicrobial susceptibility (AST) data collected retrospectively for 2016–2019 from 205 laboratories across 14 African countries. We generated 95% confidence intervals (CIs) for aggregated AMR estimates to account for data quality disparities across countries; the median data quality score was 73.1%, ranging from 56.4% to 80.8%. We assessed 819,584 culture records covering 9,266 pathogen–drug combinations, of which 187,832 (22.9%) were positive cultures with AST results. The most frequently cultured specimens were urine (32.0%) and purulent samples (28.1%), and the most frequently isolated pathogens were *Escherichia coli* (22.2%) and *Staphylococcus aureus* (15.0%). Aggregated AMR estimates did not change significantly across the years studied (*p* > 0.337); however, there were significant variations in AMR prevalence estimates in culture-positive samples across countries, regions, patient departments (inpatient/outpatient), and specimen sources (*p* < 0.05). Male sex (adjusted odds ratio [aOR] 1.15; 95% CI [1.09,1.21]; *p* < 0.0001), ages above 65 (aOR 1.28; 95% CI [1.16–1.41]; *p* < 0.0001), and inpatient department (aOR 1.24; 95% CI [1.13–1.35]; *p* < 0.0001) were associated with higher AMR prevalence among culture-positive samples. The lack of routine testing, as reflected in the low data volume from most contributing laboratories, and the absence of patient clinical information, represent significant limitations of this study.

**Conclusion:**

Analysis of the largest retrospective AMR dataset in Africa indicates high variability in AMR prevalence across countries, coupled with differences in AMR testing capacities, data quality, and AMR estimates. Gaps in AST practices and inadequate digital infrastructures for data collection and reporting represent barriers to estimating the true AMR burden in the region. These barriers warrant large-scale investments to expand healthcare access and strengthen bacteriology laboratory capacities.

## Author summary

**Why was this study done?:**

- Antimicrobial resistance (AMR) is a pressing global health issue that contributes to a decline in the effectiveness of antibiotics and complicates the treatment of infections.
- In Africa, AMR rates are among the highest in the world; however, the scope of AMR burden remains unclear due to many African countries lacking sufficient AMR testing and reporting systems.
- Local data is essential for creating effective strategies to combat AMR at the national and regional levels.

**What did the researchers find?:**

- We analyzed AMR data collected between 2016 and 2019 from 205 laboratories across 14 African countries and estimated antibiotic resistance rates in bacterial isolates obtained from patients in hospital and outpatient settings.
- Overall, the highest antibiotic resistance rates were observed among third-generation cephalosporin-resistant Enterobacterales isolates, while the lowest were observed for carbapenem-resistant Enterobacterales.
- Data volume and quality, as well as AMR prevalence, varied greatly across healthcare facilities and countries. Only 12% of the AMR records were linked to patient clinical information.
- The prevalence of AMR was greater among bacterial isolates collected from male patients, those aged over 50, and hospitalized patients.
- The lack of routine testing, reflected in the low data volume from most contributing laboratories, is a critical limitation of this study because it increases the likelihood that most AMR records originate from very sick patients or those who have experienced treatment failure with previous antibiotic treatments.

**What do these findings mean?:**

- The substantial variation in the ability to detect AMR across the 14 African countries warrants greater investments to increase patient access to diagnostic options and scale up AMR surveillance.
- There is a high level of antibiotic resistance in bacterial pathogens that are known to contribute to high mortality rates and treatment challenges.
- The lack of linkage between patient AMR and clinical data hinders the understanding of the key factors that can impact AMR.

## Introduction

Antimicrobial resistance (AMR) threatens our ability to effectively treat bacterial infections [1]. Although global and regional estimates suggest that Africa has one of the highest AMR rates globally, these estimates are based on data that may not entirely represent the region, despite significant efforts to improve surveillance over the years [2]. Data gaps are particularly acute in countries where bacterial culture and antibiotic susceptibility tests (ASTs) are conducted infrequently, and results are not readily available to reporting systems [3]. A 2017 study reports that AMR data were unavailable for 23 out of 55 countries in the African continent [4]. As of 2022, 38 countries in the World Health Organization (WHO) African Region were enrolled in the Global Antimicrobial Resistance and Use Surveillance System (GLASS); however, only 20 of the countries (52.8%) reported AMR data [5]. In countries with AMR surveillance infrastructure, national AMR surveillance systems are plagued by poor data quality, lack of data representativeness, and, most importantly, inadequate data reporting systems [6,7]. Insufficient human resources, supply chain issues, and stockouts of common reagents and microbiology laboratory supplies hamper laboratory quality management and diagnostic capacities in low-resource settings [8,9].

To compensate for data scarcity challenges, some studies have relied on mathematical modeling to project AMR prevalence and burden globally [10]. These estimates suggest higher AMR prevalence and associated mortality and morbidity in African countries [10]. While these global and regional averages are critical to raising awareness of the AMR burden, local data are needed to develop, implement, and monitor relevant AMR containment policies. Often, these data may exist in a country’s healthcare facilities but are inaccessible due to a lack of reporting and data-sharing systems. In 2018, the Africa Centers for Disease Control (Africa CDC) launched a continental framework to address AMR surveillance gaps in the region [11]. The Mapping AMR and Antimicrobial use Partnership (MAAP), a consortium supported by the UK Fleming Fund that included the African Society for Laboratory Medicine (ASLM); East, Central, and Southern Africa Health Community; Innovative Support to Emergencies, Diseases, and Disasters; IQVIA; One Health Trust; and West African Health Organization, collected retrospective AMR and antimicrobial consumption (AMC) data from laboratories from 14 countries in Africa (Burkina Faso, Cameroon, Gabon, Ghana, Kenya, Eswatini, Malawi, Nigeria, Senegal, Sierra Leone, Tanzania, Uganda, Zambia, and Zimbabwe). The study revealed significant gaps in bacteriology testing capacities [12]. Here, we present data collected within the MAAP study and report on AMR prevalence estimates from cultures collected from 205 laboratories across 14 countries. As aggregated AMR estimates from sources with varying data quality can overestimate or underestimate AMR prevalence, we performed data quality assessments to enhance data value for analysis. The findings can serve as a baseline for prospective AMR surveillance, highlight gaps, and recommend measures for strengthening surveillance and data utilization.

## Methods

### Laboratory selection and data collection

The laboratory selection process in each country was performed in collaboration with the respective Ministries of Health. The objective was to select a maximum of 16 facilities comprising public (*n* = 10), private (*n* = 4), and reference (*n* = 2) laboratories in each country. Details regarding the laboratory selection process have been described elsewhere [12]. Briefly, the national AMR coordinating committees (AMRCCs) and laboratory governance officials provided the list of all laboratories with reported bacteriology testing capacity in the 14 participating countries. The AMR detection capacity of each laboratory was evaluated through a self-administered electronic survey tool inquiring about (i) bacteriology testing infrastructure and equipment, (ii) quality management systems, (iii) the quality and standardization of bacteriology testing, (iv) human resources assigned to bacteriology testing, (v) specimen management, (vi) laboratory information systems (LISs), and linkage of laboratory, clinical, and demographic data (S1 Table). This survey was used to identify bacteriology laboratories, which were then assigned AMR detection readiness scores. The final selection of laboratories was made in consultation with the respective AMRCCs, considering the laboratories’ AMR detection readiness scores, geographical coverage, and representativeness [12].

We collected samples retrospectively through convenience sampling, including all recorded samples from inpatients and outpatients between 2016 and 2019 in the selected laboratories. The MAAP consortium data collection was conducted in collaboration with the participating countries’ AMRCCs and Ministries of Health, using a pre-approved data collection and analysis plan (S1 File). We trained field data collectors on AMR data collection and entry in WHONET, a free, Windows-based database software program for managing and analyzing microbiology laboratory data [13]. AMR-related data from electronic and paper-based records were transferred to a secure cloud-based repository, ResistanceMap Surveillance Network (www.surveillance.onehealthtrust.org), for further analysis. This study is reported as per the Strengthening the Reporting of Observational Studies in Epidemiology (STROBE) guidelines.

### AMR prevalence estimation

We estimated AMR prevalence, defined as the proportion of resistant isolates out of all tested isolates, where AST results of at least 30 isolates were available, regardless of the specimen type [14]. When only the minimum inhibitory concentration or zone diameter values were available, we classified isolates as resistant, intermediate, or susceptible based on the AST standard used by each laboratory. Although resistance interpretations were based on results from the tested antimicrobials, they were represented at the antimicrobial class level wherever possible.

### Data quality and verification

We assessed the retrieved data for completeness, accuracy, and redundancy. Where unique patient identification numbers were available, we assessed patient records over multiple visits, excluded duplicate AST results, and considered only the result of the first pathogen isolate per patient per year, regardless of the AST profile or specimen source.

**Fig 1:**
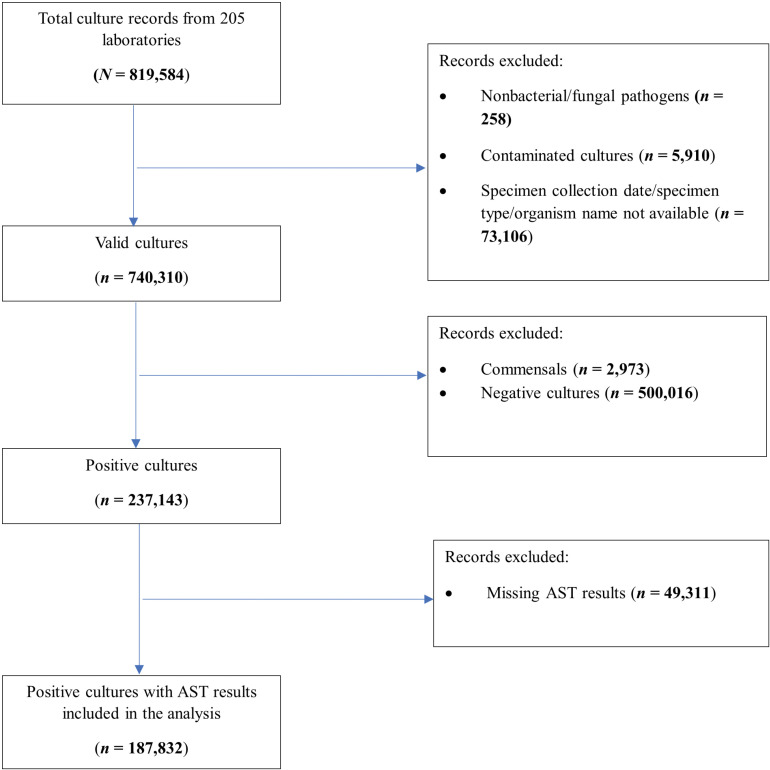
Flow chart of culture records included in the analysis.

Valid cultures were defined as the subset of total culture records with complete information on specimen source, collection date, and pathogen name. Records with nonbacterial pathogens were considered invalid (Fig 1). Positive culture records were defined as the subset of valid culture records for which pathogen growth was reported, regardless of the availability of AST results. However, only positive culture records accompanied by AST results were included in the analysis. Considering the disparities in data sources and sample volumes, the imputation of the missing AST data was not performed to prevent additional bias and uncertainty in the AMR estimates.

### Data quality scores

We developed a scoring system to assess the overall quality of the AMR data, reflecting compliance with the reported AST standards. The laboratory data quality score was calculated as the average of the proportion of test results with defined AST breakpoints and the proportion of results indicating nonsusceptibility where resistant phenotypes were expected (S3 Table) (Equation 1).


Lab data quality score=% of AST results with+% of resustant results where defined breakpointsresistant phenotyoe is expected2 (1)

We derived data quality scores for each country by calculating the average of all laboratory data quality scores within a country weighted by the proportion of AST results contributed by each respective laboratory, using Equation (2):


$$\begin{array}{c} Country\;data\;quality\;score\;=\sum_{i=1}^{n}\frac{\left({\text{laboratory\textbackslash{} data\textbackslash{} quality\textbackslash{} score}}_{\left(i\right)}\times \;\sum {\text{AST\textbackslash{} results}}_{\left(i\right)}\right)}{\sum {\text{AST\textbackslash{} results}}_{\left(i\ldots n\right)}},\; \end{array}$$ (2)

where *n* is the total number of contributing laboratories in a country, and *i* represents individual laboratories. The quality scores ranged from 0 (lowest) to 100 (highest), facilitating the comparison of data quality across facilities and countries included in the study.

### Statistical analysis

We estimated AMR prevalence by aggregating AST data from contributing laboratories within each country. We used cluster robust errors at 95% confidence interval (CI) to account for the clustered sampling design [15], nesting the laboratories within countries for regional estimates. We summarized AMR prevalence at the regional level and assessed trends across years using the Kruskal–Wallis analysis of variance. We used the Tukey–Kramer multiple-comparison correction method to investigate the differences in AMR prevalence between levels in a subgroup analysis (regions, patient departments, and specimen sources). We conducted the analyses using R Software version 4.4.0. Statistical significance was set at *P* < 0.05.

### DRI

A composite DRI [16], representing resistance to several drugs weighted by their relative use, was estimated for select groups of critical pathogens (*Acinetobacter baumannii*, *Escherichia coli*, *Klebsiella pneumoniae*, *Pseudomonas aeruginosa*, *Staphylococcus aureus*, *Enterococcus faecium*, *Enterococcus faecalis, Haemophilus influenzae, Streptococcus pneumoniae,* and *Shigella* spp.) and antimicrobials or antimicrobial classes (aminoglycosides, broad-spectrum penicillins, carbapenems, third-generation cephalosporins, glycopeptides, narrow-spectrum penicillins, macrolides, and quinolones) (S4 Table). In addition to offering a standardized means of comparing resistance levels among countries, DRI enables monitoring resistance trends over time, thereby facilitating the evaluation of intervention effectiveness:


$$R_{i,\;fixed-use}=\sum_{k}\rho_{ik}^{t}q_{ik}^{0},\;$$ (3)

where $\rho_{ik}^{t}\;$ is the proportion of resistance among organism *i* to drug *k* at time *t*, and $q_{ik}^{0}$ is the frequency of drug *k* used to treat organism *i* in the base year of the analysis.

Pharmacy-level AMC data were collected from pharmacies co-located with AST laboratories. Data were extracted from the facilities’ Health Management Information System or entered manually from stock record cards and then transferred securely to the MAAP tool. AMC data for each antibiotic were presented in defined daily dose (DDD) units, obtained by dividing the amount used by WHO-approved DDD value.

DRIs were estimated for samples collected from countries where data were available for at least 15 of the 33 possible drug–pathogen combinations. Out of the 205 laboratories included in this study, DRI was estimated using data from 158 pharmacies co-located with the respective hospital facilities. The CIs for the DRI estimates were obtained as the product of two variances (antibiotic resistance and antibiotic consumption) [17].

### AMR risk factors

A composite AMR prevalence across all years for select groups of priority pathogens (*A. baumannii*, *E. coli*, *K. pneumoniae*, *P. aeruginosa*, *S. aureus*, *E. faecium*, *E. faecalis, H. influenzae, S. pneumoniae,* and *Shigella* spp.) was used to assess associations between AMR and patient-related factors. Antimicrobials included aminoglycosides, broad- and narrow-spectrum penicillins, carbapenems, third-generation cephalosporins, glycopeptides, macrolides, and quinolones. We assessed collinearity between AMR estimates and age, sex, country of origin, department (inpatient/outpatient), specimen source, and laboratory tier level using Spearman’s correlation test, where *r*_s _= 0.7 indicated highly correlated samples. Associations between AMR prevalence and antibiotic usage (defined as antibiotic use in the last 3 months) were performed on data obtained from four countries (S5 Table). We performed logistic regression with cluster robust errors with a statistical significance of *p* < 0.05.

### Ethical considerations

This study, analyzing routine laboratory and AMR data, was approved by the Ghana Health Service Ethics Review Committee (GHS-ERC 001/11/20). Additionally, data-sharing agreements were obtained from relevant institutions in each participating country. No identifiable, patient-level information was used.

## Results

### Laboratory selection

The selection of the participating laboratories has been described in detail elsewhere [12]. Briefly, 1.3% (665) of the 50,000 facilities identified across 14 countries (Burkina Faso, Cameroon, Gabon, Ghana, Kenya, Eswatini, Malawi, Nigeria, Senegal, Sierra Leone, Tanzania, Uganda, Zambia, and Zimbabwe) had the capacity to conduct bacteriology testing. Of the 485 (73%) facilities that responded to an online questionnaire on AMR laboratory capacity, 393 indicated that they performed bacteriology testing. Finally, 205 laboratories were selected for data collection, with 9 out of 14 countries having 16 participating laboratories and population coverage ranging between 24% in the United Republic of Tanzania and 68% in Kenya (S6 Table). The median aggregated data quality score across the 14 countries was 73.1, ranging from 56.4 in Sierra Leone to 80.8 in Senegal (S7 Table). Of the 205 laboratories included in the study, 138 (67.3%) adopted the Clinical and Laboratory Standards Institute AST guidelines, 40 (19.5%) adopted the European Committee on Antimicrobial Susceptibility Testing guidelines, and 27 (13.2%) used both.

### Characteristics of bacteriology cultures

We retrieved 819,584 culture records from 2016 to 2019, of which 740,310 (90.3%) had valid records reporting the specimen type and collection date (Fig 1). Of the valid records, 187,832 (25.4%) were positive cultures with AST results; 187,771 (99.9%) included patient demographic information (age and sex), and 22,716 (12.1%) were linked to clinical data (specimen source, inpatient/outpatient). The number of positive cultures with AST results per 100,000 people varied from 9 in Sierra Leone to 452 in Eswatini (Table 1). The most frequently cultured specimens among positive cultures with AST results were urine (32.0%), purulent samples (28.1%), and blood (14.4%). The most frequently isolated pathogens were *E. coli* (22.2%), *S. aureus* (15.0%), and *K. pneumoniae* (7.7%).

**Table 1:** Demographic distribution of positive culture records with AST results across the 14 countries (*N* = 187,832).

Country	Cultures (per 100,000 people^#^)	Sex		Age in years					Department	
		**Female**	**Male**	**<1**	**1–17**	**18–49**	**50–65**	**>65**	**Inpatient**	**Outpatient**
Burkina Faso	7,765 (37.9)	4,129 (53.4)	3,610 (46.6)	407 (5.3)	1,051 (13.6)	3,237 (41.8)	1,034 (13.4)	1,219 (15.8)	870 (38.9)	1,366 (61.1)
Cameroon	32,545 (129.8)	24,420 (75.0)	8,124 (25.0)	1,998 (6.1)	2,049 (6.3)	18,736 (57.6)	2,441 (7.5)	1,782 (5.5)	2,842 (15.7)	1,5,314 (84.3)
Eswatini	5,247 (452.2)	3,224 (61.4)	2,023 (38.6)	472 (9.0)	772 (14.7)	2,504 (47.7)	718 (13.7)	484 (9.2)	1,735 (51.0)	1,665 (49.0)
Gabon	8,425 (384.4)	6,710 (79.6)	1,709 (20.3)	102 (1.2)	664 (7.9)	4,701 (55.8)	499 (5.9)	291 (3.5)	104 (2.1)	4,961 (97.9)
Ghana	4,394 (14.2)	2,928 (66.6)	1,466 (33.4)	391 (8.9)	652 (14.8)	1,084 (24.7)	254 (5.8)	226 (5.1)	1,435 (52.9)	1,280 (47.1)
Kenya	16,027 (32.1)	9,778 (61.0)	6,249 (39.0)	1,782 (11.1)	2,175 (13.6)	5,495 (34.3)	1,441 (9.0)	1,178 (7.4)	6,349 (70.3)	2,683 (29.7)
Malawi	7,196 (39.2)	3,517 (48.9)	3,679 (51.1)	806 (11.2)	2,316 (32.2)	1,878 (26.1)	317 (4.4)	252 (3.5)	510 (23.1)	1,697 (76.9)
Nigeria	23,963 (12.1)	13,803 (57.6)	10,160 (42.4)	2,711 (11.3)	4,526 (18.9)	7,629 (31.8)	1,630 (6.8)	1,806 (7.5)	6,590 (46.0)	7,748 (54.0)
Senegal	8,763 (56.3)	4,356 (49.7)	4,393 (50.1)	325 (3.7)	710 (8.1)	3,324 (37.9)	1,294 (14.8)	2,151 (24.5)	2,071 (31.2)	4,576 (68.8)
Sierra Leone	723 (9.2)	483 (66.8)	240 (33.2)	1 (0.1)	23 (3.2)	53 (7.3)	6 (0.8)	9 (1.2)	3 (4.6)	62 (95.4)
Tanzania	13,204 (22.7)	9,049 (68.5)	4,155 (31.5)	1,027 (7.8)	1,712 (13.0)	3,259 (24.7)	1,267 (9.6)	1,335 (10.1)	4,857 (51.9)	4,498 (48.1)
Uganda	22,349 (53.8)	13,711 (61.3)	8,638 (38.7)	691 (3.1)	4,310 (19.3)	9,505 (42.5)	1,571 (7.0)	1,284 (5.7)	3,596 (32.4)	7,489 (67.6)
Zambia	22,343 (125.3)	20,923 (93.6)	1,420 (6.4)	1,774 (7.9)	4,231 (18.9)	9,979 (44.7)	1,827 (8.2)	1,615 (7.2)	3,801 (66.3)	1,931 (33.7)
Zimbabwe	14,888 (98.9)	8,492 (57.0)	6,392 (42.9)	1,032 (6.9)	2,444 (16.4)	5,558 (37.3)	1,236 (8.3)	2,062 (13.9)	8,389 (57.5)	6,209 (42.5)
Total		125,523 (66.8)	62,258 (33.2)	13,519 (9.1)	27,635 (18.5)	76,942 (51.5)	15,535 (10.4)	15,694 (10.5)	43,152 (41.2)	61,479 (58.8)

# Based on the 2018 World Bank population estimates.

AST, antimicrobial susceptibility test.

### AMR prevalence

**Fig 2:**
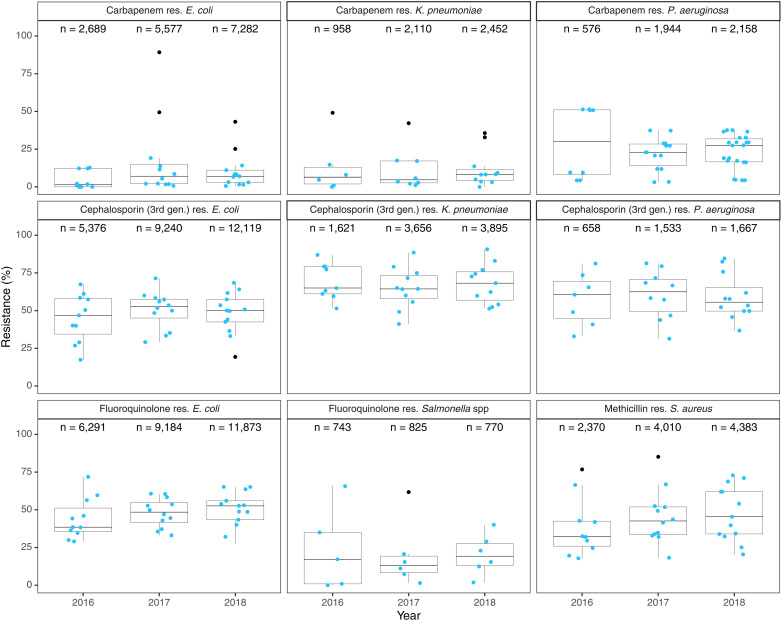
Antimicrobial resistance prevalence trends of five priority pathogens, 2016–2018. Blue data points represent the prevalence in countries; the center lines of the boxplots represent the median; the lower and upper box limits represent the first and third quartiles, respectively; the whiskers represent minimum and maximum values without outliers, and black data points represent the outliers. The total number of tested isolates (*n*) is provided for each year. *Note:* Data from 2019 were insufficient for most countries and were, therefore, excluded from the trend analysis.

**Fig 3:**
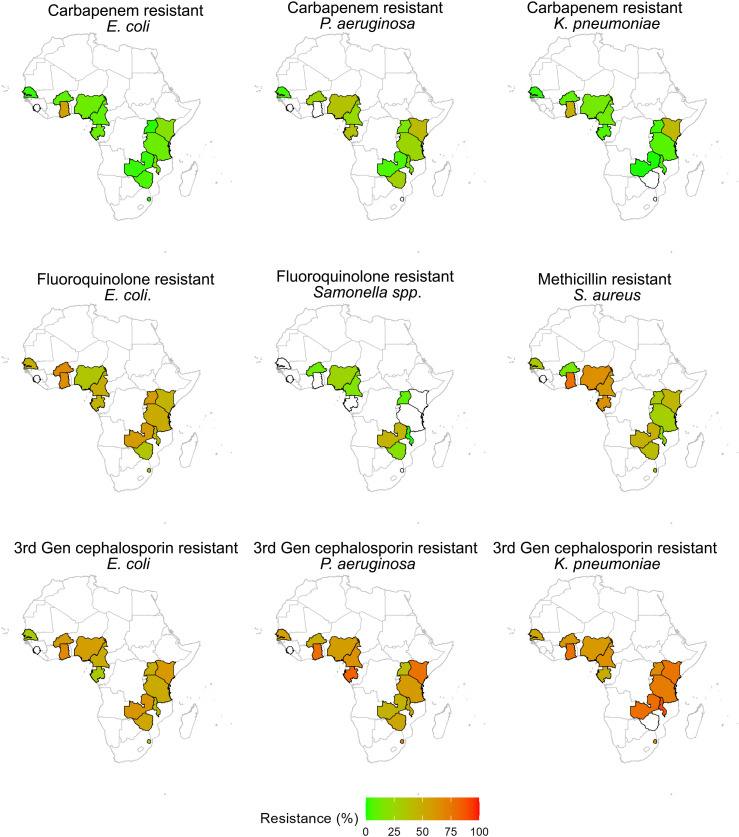
Antibiotic resistance prevalence for five priority pathogens in 13 countries in sub-Saharan Africa, 2016–2019. Aggregate percentages of resistant *Escherichia coli*, *Staphylococcus aureus*, *Salmonella* spp.*, Pseudomonas aeruginosa,* and *Klebsiella pneumoniae* isolates to various antibiotics between 2016 and 2019. The map base layer is available from Natural Earth, in the public domain. Link to map base layer: https://naciscdn.org/naturalearth/10m/cultural/ne_10m_admin_0_countries.zip.

We estimated AMR prevalence for pathogens in the 2017 WHO bacterial priority pathogen list (*A. baumannii*, *Campylobacter,* Enterobacterales*, E. faecium, H. influenzae, Helicobacter pylori, N. gonorrhoeae, P. aeruginosa, Salmonella, Shigella, S. aureus,* and *S. pneumoniae*) from samples collected from 13 countries; samples from Sierra Leone were insufficient for analysis. Overall, AMR prevalence was highest for third-generation cephalosporin-resistant Enterobacterales, while carbapenem-resistant Enterobacterales displayed the lowest resistance levels across countries (S8 Table). A comparison of aggregated AMR prevalence across years for five priority pathogens (*E.coli*, *S. aureus*, *Salmonella* spp.*, P. aeruginosa,* and *K. pneumoniae)* showed no significant changes between 2016 and 2018 (*P* > 0.337) (Fig 2). In 2018, the year that contained the most data from all countries, third-generation cephalosporin resistance among Enterobacterales ranged from 30.3% (95% CI [5.8%,75.3%]) in Eswatini to 73.5% (95% CI [67.7%,78.7%]) in Ghana (Fig 3 and S8 Table). Carbapenem-resistant Enterobacterales ranged from 1.0% (95% CI [0.2%,5.0%]) in Malawi to 49.6% (95% CI [25.8%,73.6%]) in Ghana, and carbapenem-resistant *P. aeruginosa* ranged from 4.4% (95% CI [2.1%,8.9%]) in Senegal to 37.5% (95% CI [9.7%,77.0%]) in Gabon (S9 Table). The prevalence of methicillin-resistant *S. aureus* (MRSA) ranged from 20.4% (95% CI [14.9%,27.2%]) in Burkina Faso to 72.8% (95% CI [59.9%,82.7%]) in Nigeria, exceeding 30% in 11 countries. AMR prevalence for fluoroquinolone resistance among *Salmonella* isolates varied widely between countries, from 1.8% (95% CI [0.2%,14.7%]) in Malawi to 40.0% (95% CI [17.6%,67.5%]) in Zambia (S8 Table). *E. coli* resistance to third-generation cephalosporins ranged from 19.3% (95% CI [7.1%,42.6%]) in Eswatini to 68.4% (95% CI [52.8%,80.6%]) in Ghana, and resistance to fluoroquinolones ranged from 27.6% (95% CI [19.5%,37.6%]) in Eswatini to 65.3% (95% CI [56.3%,73.2%]) in Burkina Faso. Among *K. pneumoniae* isolates, carbapenem resistance ranged from 0% in Zambia to 35.7% (95% CI [6.9%,80.6%]) in Kenya, and third-generation cephalosporin resistance ranged from 51.4% (95% CI [44.7%,58.0%]) in Senegal to 90.6% (95% CI [87.6%,92.9%]) in Malawi. AMR rates for other clinically important pathogens are included in S9 Table. Subgroup analyses based on department (inpatient/outpatient) and specimen source (blood/cerebrospinal fluid and others) are included in S10 and S11 Tables, respectively.

### AMR prevalence across regions, patient departments, and specimen sources

**Fig 4:**
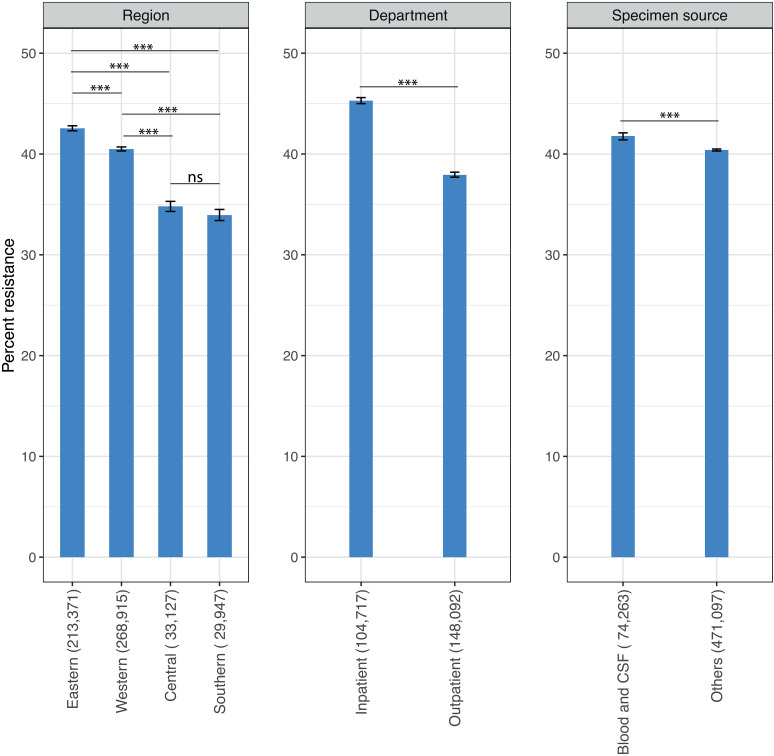
Antimicrobial resistance prevalence of all bacterial isolates by region, patient department, and specimen source. The error bars represent 95% confidence intervals, and the asterisks (***) denote a statistically significant difference in AMR prevalence among the different categories (*p* < 0.001); ‘ns’ indicates no significance (*p* > 0.05). The total number of isolates for each category is indicated in parentheses. CSF, cerebrospinal fluid. *Note*: Countries are categorized into regions based on the Global Burden of Disease classification (Central—Gabon; Eastern—Malawi, Kenya, Uganda, Tanzania, and Zambia; Southern—Eswatini and Zimbabwe; Western—Burkina Faso, Cameroon, Ghana, Nigeria, Senegal, and Sierra Leone). The error bars represent 95%.

A comparison of overall AMR prevalence among culture-positive samples collected across regions (Central, Eastern, Southern, and Western) showed significant differences, with Western and Eastern regions, having the highest percentage of resistant isolates at 40.5% (95% CI [40.3%,40.7%]) and 42.5% (95% CI [42.3%,42.8%]), respectively (Fig 4 and S12 Table). The largest difference in AMR prevalence was 8.6% (95% CI [8.0%,9.2%]; *p* < 0.0001) observed between samples from Eastern and Southern (33.9%, 95% CI [33.4%,34.5%]) regions. AMR prevalence in culture-positive samples from inpatient (45.3%, 95% CI [45.0%,45.6%]) group was significantly higher by 7.4% (95% CI [7.0%,7.8%]; *p* < 0.0001) compared with the outpatient groups (37.9%, 95% CI [37.7%,38.2%]). Furthermore, AMR prevalence from blood/ cerebrospinal fluid (41.8%, 95% CI [41.4%,42.1%]) was marginally higher by 1.4% (95% CI [1.0%,1.8%]; *p* < 0.0001) compared with other samples (40.4%, 95% CI [40.3%,40.5%]) (Fig 4 and S12 Table).

### Risk factors associated with AMR prevalence in 13 countries

In multivariable logistic regression, AMR prevalence in culture-positive samples was associated with age, sex, country, and department (inpatient/outpatient). Samples from patients aged 50–65 years (adjusted odds ratio (aOR) 1.13, 95% CI [1.06,1.21]; *p* < 0.0001) and 65 + years (aOR 1.28, 95% CI [1.16,1.41]; *p* < 0.0001) had higher odds of resistance than those collected from adults aged 18–49 (Table 2). AMR prevalence was also associated with sex, with culture-positive samples from male patients having higher odds of antibiotic resistance (aOR 1.15, 95% CI [1.09,1.21]; *p* < 0.0001) than those collected from female patients. Finally, culture-positive samples collected from inpatients were more likely to comprise resistant pathogens than those collected from outpatients (aOR 1.24, 95% CI [1.13,1.35]; *p* < 0.0001). Prior antibiotic usage was also associated with AMR prevalence (aOR 1.37, 95% CI [1.05,1.80]; *p* = 0.022) in four countries where the data were available (S5 Table).

**Table 2:** Multivariable logistic regression analysis between antimicrobial resistance prevalence and other factors.

Variable	Options	*N*^#^ (*R*^†^%)	Adjusted odds ratio (95% CI)	*P*-value
Age, years	18–49	37,655 (43.9)	Ref^*^	
	<1	7,254 (47.7)	1.05 (0.92–1.19)	0.470
	1–17	14,312 (43.4)	0.88 (0.8–0.98)	0.019
	50–65	11,529 (47.1)	1.13 (1.06–1.21)	<0.0001
	>65	11,370 (49.6)	1.28 (1.16–1.41)	<0.0001
Sex	Female	46,836 (43.5)	Ref^*^	
	Male	35,284 (48.0)	1.15 (1.09–1.21)	0.0001
Country	Nigeria	15,209 (49.6)	Ref^*^	
	Burkina Faso	4,288 (51.2)	1.03 (0.71–1.5)	0.880
	Cameroon	11,961 (44.7)	0.86 (0.59–1.26)	0.439
	Eswatini	2,799 (43.2)	0.76 (0.53–1.09)	0.138
	Gabon	3,020 (38.6)	0.74 (0.42–1.32)	0.312
	Ghana	2,329 (60.8)	1.69 (1.16–2.48)	0.007
	Kenya	3,683 (42.6)	0.71 (0.45–1.11)	0.132
	Malawi	1,545 (49.5)	1.09 (0.67 - 1.8)	0.721
	Senegal	15,685 (39.8)	0.65 (0.47–0.92)	0.014
	Tanzania	6,074 (44.1)	0.78 (0.53–1.14)	0.197
	Uganda	10,803 (43.4)	0.82 (0.58–1.16)	0.260
	Zambia	1,292 (67.0)	2.06 (1.07–3.97)	0.031
	Zimbabwe	3,432 (46.2)	0.86 (0.5–1.5)	0.598
Specimen source	Others	72,539 (45.0)	Ref^*^	
	Blood/CSF	9,581 (48.2)	0.94 (0.81–1.1)	0.426
Department	Outpatient	49,701 (42.7)	Ref^*^	
	Inpatient	32,419 (49.6)	1.24 (1.13–1.35)	<0.0001
Laboratory tier level	District or Community	3,911 (46.5)	Ref^*^	
	Reference	33,524 (46.1)	0.97 (0.61–1.55)	0.910
	Regional or Intermediate	33,686 (46.5)	0.98 (0.65–1.49)	0.933
	Unspecified	10,999 (39.5)	0.83 (0.51–1.33)	0.431

# *N* = number of tested isolates.

† *R*% = proportion of resistant isolates.

* Ref = reference category.

CSF, cerebrospinal fluid.

### DRI

DRIs estimated on available AMR and AMC data from 11 countries ranged between 40.3% (95% CI [10.6%,69.9%]) in Kenya and 80.7% (95% CI [74.7%,86.7%]) in Senegal (S1 Fig). Aminopenicillins comprised the most consumed antibiotic class (median, 32.0%) and had the highest AMR prevalence (median, 82.8%). Carbapenems displayed the lowest use and resistance levels, with medians of 0.04% and 13.0%, respectively.

## Discussion

To our knowledge, this study represents the largest and most representative AMR dataset from Africa [18]. It includes data from 187,832 culture records with interpretable AST results spanning 205 laboratories in 14 countries. Our findings indicate high AMR prevalence among WHO critical- and high-priority bacterial pathogens and wide variability across countries. Analysis of AMR estimates for select pathogens suggests that age, sex, and department (inpatient/outpatient) are associated with higher odds of resistance among culture-positive samples.

AMR surveillance gaps in Africa continue to challenge the understanding of the AMR burden and negatively affect policies and financing of mitigating interventions. To provide perspective on the depth of this issue, while AMR is a leading cause of infection-related deaths [2], the AST results reported from Africa each year are on the same scale as those from a single tertiary hospital in many parts of the world. In 2022, bacteriologically confirmed infections with AST from African countries accounted for only 4.5% (31,896/686,106) of the results reported to GLASS-AMR, translating to a median of 7.2 per 1 million population, much lower than the global median of 103.6 samples per 1 million [5]. Much work remains to be done to improve routine reporting on AMR.

Consistent with earlier studies, the most commonly isolated pathogens were *E. coli, S. aureus,* and *K. pneumoniae* [4]. These pathogens have been linked to a high proportion of AMR-related deaths globally and in WHO African region [10]. In a 2019 study, *K. pneumoniae* was linked to 184,000 (142,000–236,000) AMR-associated deaths, *E. coli* to 147,000 (112,000–189,000), and *S. aureus* to 136,000 (109,000–172,000) [2,10]. MRSA prevalence was previously estimated below 30% for all the countries included in our study except Nigeria and Gabon, at 30%–50% [10]. In contrast, we found that MRSA prevalence in 2018 exceeded 50% in Nigeria (72.8%, 95% CI [59.9%,82.7%]), Ghana (71.1%, 95% CI [48.5%,86.5%]), Gabon (68.8%, 95% CI [39.2%,88.3%]), Cameroon (62.0%, 95% CI [38.7%,80.9%]), Zambia (62.0%, 95% CI [56.0%,67.6]), and Eswatini (54%, 95% CI [2.7%,98.0%]). In the majority of countries in our study, prevalence estimates for third-generation cephalosporin-resistant *E. coli* were higher compared to the 2019 study [10], except for Eswatini, which had a lower prevalence (19.7%, 95% CI [7.1%,42.6%]) in this study, and Gabon and Senegal where the estimates of both studies were between 30% and 40%. Our estimates consistently exceeded previously modeled estimates across many other drug–pathogen combinations [10]. The levels of carbapenem-resistant Enterobacterales aligned with previous studies, which reported the highest prevalence in East Africa at 35% and 14.2% in Nigeria [19,20]. A recent meta-analysis reports a pooled prevalence of 8% for carbapenem-resistant *P. aeruginosa* in Africa, corresponding closely with the lowest prevalence of 4.4% (95% CI [2.1%,8.9%]) observed in Senegal from our study [21]. The subgroup analysis of aggregated AMR data from all countries suggests differences in the percentage of resistant isolates among culture-positive samples collected across regions, with Central and Southern regions having lower levels of resistant isolates. However, this analysis is limited by the geographical representation of the available data, where Central Africa was represented by one country (Gabon) and Southern Africa by two countries (Eswatini and Zimbabwe). Nevertheless, comparison between samples indicates that the origin of the sample—whether it was isolated in the inpatient or an outpatient setting—may contribute more to the heterogeneity of resistance rates than the type of sample (blood/cerebrospinal fluid versus others). Several factors could impact AMR prevalence in these two patient groups. Hospitalized patients often have longer exposure to broad-spectrum antibiotics, which increases the chances of developing resistant strains [22]. Moreover, healthcare environments tend to promote the spread of these resistant organisms due to close patient interactions, the use of medical devices, and contamination of the surroundings; patients who are critically ill and often need invasive procedures are at a heightened risk for multidrug-resistant infections [23].

The comparisons in this study could point to how inadequate surveillance data across regions and countries could obscure a growing resistance problem; however, it is worth noting that differences in data collection and analysis methods may further enhance uncertainties in AMR estimates. A large survey of the AMR testing capacities in the 14 countries included in this study identified few bacteriology laboratories and even fewer facilities demonstrating sufficient technical capacity and resources to conduct AMR testing [12]. The reported inadequate number of bacteriology laboratories, lack of routine AST testing, predominance of paper-based documentation, and underreporting of clinical data undermine the ability to estimate regional and national AMR prevalence and drivers of resistance. Differences in methodologies, frequency of routine testing, and patient population may have contributed to the heterogeneity in AMR estimates across countries. For example, the number of Enterobacterales tested for cephalosporins and carbapenems varied across several countries, suggesting potential differences in the decision to test for a particular antibiotic. In some settings, carbapenems may have been tested only in isolates with cephalosporin resistance, which could impact the AMR prevalence estimates. The testing frequency, however, greatly influences AMR estimates due to its impact on sample size and the population included in the study. While our findings represent a unique snapshot of AMR prevalence in samples collected from several African countries, corresponding clinical data could enhance their utility for designing targeted public health interventions. Only 12% of the AMR records included patient clinical information, limiting the utility of the findings to an insight into AMR prevalence without any inference to potential drivers and mitigating factors.

The variation in AMR estimates in our study was accompanied by variation in DRI values, where the highest DRI estimate was nearly twice that of the lowest. As a composite measure of antibiotic use and resistance, DRI reflects the overall effectiveness of antibiotics. As an example, high levels of antibiotic use would not lead to a high DRI if antibiotics were effective in treating the respective infections. However, high AMR prevalence to certain antibiotics can lead to a high DRI even if their use is relatively low. Variabilities in the DRI values, which exceeded 40% from data collected in all countries in our study, are consistent with findings from a previous study in which DRIs exceeded 50% in low- and middle-income countries [24]. Of note, high DRI values could reflect high resistance values and also a lack of access to the right antibiotics, considering that many infections could be treated with newer and more effective antibiotics.

AMR prevalence was associated with patient-related factors, such as age and sex, and whether the samples were collected from the inpatient or outpatient setting. Older adults are at higher risk of drug-resistant infections [10], and recent modeling estimates suggest that AMR-related deaths in adults older than 70 years more than doubled between 1990 and 2021 [25]. Samples collected from male patients were associated with higher odds of resistance in this study. However, the findings should be interpreted cautiously, considering the many social and biological confounding factors that must be accounted for. An observational study assessing AMR prevalence in bloodstream infections in 29 European countries showed an interaction between sex and age; male sex was associated with a higher risk in certain age groups [26]. Another study (systematic review) showed that men are less likely to adhere to antibiotic treatments [27]. Antibiotic use is a critical driver of AMR and has been linked to a higher risk of resistant infections in studies [28,29]. Although the findings in our study are limited to data analysis of samples collected from four countries, they align with previous studies; AMR prevalence was higher in samples collected from patients with prior antibiotic use. AMR prevalence among isolates from inpatients was higher than those collected from outpatients, as previously reported in a study from a tertiary healthcare facility in Rwanda [30]. The finding implies that patients who may not have responded to previous treatment are more likely to undergo AST testing, which may be particularly relevant to our context. While our study validates earlier findings, understanding other patient-related factors, such as underlying conditions, prior treatments, and sociocultural factors, is needed to identify confounders of these associations and improve our understanding of the drivers of AMR. The higher percentage of resistant bacterial isolates among culture-positive samples collected from inpatients in this study further supports this finding.

An essential limitation of this study, which also reveals a critical gap in AMR surveillance, is the lack of routine testing reflected in the low data volume from most of the contributing laboratories. In limited-resource settings, patients with severe illness or treatment failure are more likely to be tested than those with noncomplicated and community-acquired infections, which may contribute to overestimating resistance levels. This could partly explain the higher resistance levels in our findings compared to a study that modeled AMR prevalence in the region [10]. Conversely, lack of access to healthcare services and diagnostics in low-resource settings for patients with severe AMR infections that lead to death may contribute to a significant underestimation of the AMR rates. Coupled with variations in laboratory quality and practices, these limitations warrant a cautious interpretation of the data. We accounted for the clustered nature of the AMR data by treating each country and contributing laboratory as clusters and generated CIs around the prevalence estimates using cluster robust errors [15]. However, the high variability between laboratories in testing volume and resistance prevalence was reflected in the wide CIs around the prevalence estimates. Another limitation is bias in selecting the participating facilities. Due to resource and time constraints, the study was limited to data collection from 16 laboratories in each country, with two countries having fewer than eight laboratories with AST capacity. As a result, the estimated AMR prevalences are not generalizable to the national and regional levels.

Several recommendations emerge from this study. First, the number and capacity of bacteriology laboratories capable of conducting AST urgently need to be increased. Where data are available, implementing electronic LISs, ideally linked to an electronic clinical database, can improve data quality, access, and utilization. Second, without clear guidelines for evaluating the quality of AST data used in AMR surveillance, insights into the data quality of submitting facilities could be inferred from compliance with the recommended AST guidelines and good laboratory management practices. These insights could aid in identifying and prioritizing facilities that require additional resources and mentorship for capacity building and strengthening data quality. Furthermore, countries should allocate resources towards establishing and maintaining national AMR surveillance platforms to monitor AMR prevalence. As this study has shown, data may be available in select facilities, and efforts should be made to consolidate the data at a national or regional level.

The high prevalence of AMR among priority pathogens highlighted in this study underscores the urgent need for intensified public health interventions, such as improvements in clean water and sanitation, promotion of hand hygiene practices, and vaccinations. Additionally, prioritizing increasing access to diagnostic options at the various levels of healthcare could significantly improve patient care and ensure the appropriate use of antibiotics. Ideally, these initiatives should be integrated into the national action plans on AMR and allocated resources for implementation.

## Supporting information

S1 STROBE Checklist STROBE checklist for cross-sectional studies. (PDF)

S1 File Prospective analysis plan. (PDF)

S1 Table List of authors in the Mapping AMR and Antimicrobial use Partnership study group. (PDF)

S2 Table Laboratory survey tool for antimicrobial resistance detection capacity. (PDF)

S3 Table Expected resistant phenotypes, based on the Clinical and Laboratory Standards Institute (CLSI) and the European Committee on Antimicrobial Susceptibility Testing (EUCAST) guidelines. (PDF)

S4 Table Select pathogens and antimicrobials used in estimating Drug Resistance Index. (PDF)

S5 Table Multivariable logistic regression analysis between antimicrobial resistance prevalence, prior antibiotic usage, and other factors. (PDF)

S6 Table Population coverage of the laboratories selected for data collection. (PDF)

S7 Table Distribution of collected culture records and data quality scores. (PDF)

S8 Table Antimicrobial resistance prevalence estimates for WHO priority pathogens. (PDF)

S9 Table Antimicrobial resistance prevalence estimates for clinically important pathogens, by regions. (PDF)

S10 Table Antimicrobial resistance prevalence estimates for clinically important pathogens by regions and patients’ departments (inpatient/outpatient). (PDF)

S11 Table Antimicrobial resistance prevalence estimates for clinically important pathogens by region and specimen source (blood/CSF and others). (PDF)

S12 Table Antimicrobial resistance prevalence of all bacterial isolates by region, patient department, and specimen source. (PDF)

S1 Fig Drug Resistance Index for 11 African countries, 2016–2019. (PDF)

## Abbreviations

**:**
AMR: antimicrobial resistance
AMRCCs: AMR coordinating committees
aOR: adjusted odds ratio
CI: confidence intervals
DDD: defined daily dose
GLASS: Global Antimicrobial Resistance and Use Surveillance System
LISs: laboratory information systems
MAAP: Mapping AMR and Antimicrobial use Partnership
MRSA: methicillin-resistant *S. aureus*
STROBE: Strengthening the Reporting of Observational Studies in Epidemiology
WHO: World Health Organization
