# Supplementary material for: Antimicrobial resistance in Africa: A retrospective analysis of data from 14 countries, 2016–2019
Source: PLoS Med. 2025 Jun 24;22(6):e1004638. doi: 10.1371/journal.pmed.1004638 (PMC12186946; doi:10.1371/journal.pmed.1004638)
Supplement: S1 File — (PDF) [file pmed.1004638.s002.pdf]

Prospective Analysis Plan

Fleming Fund Regional Grant (Round 1) Program
Mapping AMR and AMU Partnership (MAAP)

PURPOSE 1

OVERVIEW 1

Table 1: List of WHO priority pathogens 1

DATA ANALYSIS PLAN 2

VARIABLES OF INTEREST 2
Table 2: Variables of interest 2
Figure 1: Decision framework 4
QUALITY ASSESSMENT 4
VALIDATION AND ANALYSIS OF DATA 4
Data validation *4*
Definition of antimicrobial susceptibility *5*
Data analysis and indicators *5*
Duplicate removal *5*
Proportion non-susceptible *5*
Clinical syndromes *6*
Table 3: Metrics 6
Statistical analysis plan for AMR *6*
Drug Resistance Index *8*

APPENDICES 9

APPENDIX A: LABORATORY ELIGIBILITY QUESTIONNAIRE 9
Table A: Laboratory Eligibility Questionnaire 9
APPENDIX B: QUALITY INDICATORS 12
Table B1: Laboratory quality indicators 12
Table B2: Data quality indicators 13
Table B3: Indicators of antimicrobial stewardship (clinic/hospital information) 13

REFERENCES 14

Purpose

This document lays out the methodology for analyzing laboratory data collected under the Mapping AMR and AMU Partnership (MAAP). The goal of the analysis is to assess antimicrobial resistance (AMR) rates and trends across a sample of laboratories in Africa, measure the relationship of AMR to potential drivers of resistance, including the correlation with antimicrobial use/consumption (AMU/AMC) at regional, country, and patient levels.

Overview

The availability of reliable and comprehensive AMR surveillance data from Africa is generally lacking. Furthermore, due to weaknesses in health and laboratory systems, accessing existing representative data is challenging. The World Health Organization (WHO) has published a list of priority pathogens to promote research and development (R&D) of new antibiotics as part of efforts to address the growing global resistance to antimicrobial medicines 1. This list highlights in particular the threat posed by Gram-negative bacteria that are resistant to multiple antibiotics. The WHO list is categorized into three levels according to the urgency of need for new antibiotics: critical, high, and medium priority (Table 1).

Table 1: List of WHO priority pathogens

**Pathogen**	**Resistance**	**Priority**
Acinetobacter baumannii	carbapenem-resistant	Critical
Pseudomonas aeruginosa	carbapenem-resistant	Critical
Enterobacteriaceae	carbapenem-resistant, ESBL-producing	Critical
Enterococcus faecium	vancomycin-resistant	High
Staphylococcus aureus,	methicillin-resistant, vancomycin-intermediate and resistant	High
Helicobacter pylori	clarithromycin-resistant	High
Campylobacter spp.	fluoroquinolone-resistant	High
Salmonellae	fluoroquinolone-resistant	High
Neisseria gonorrhoeae	cephalosporin-resistant, fluoroquinolone-resistant	High
Streptococcus pneumoniae	penicillin-non-susceptible	Medium
Haemophilus influenzae	ampicillin-resistant	Medium
Shigella spp.	fluoroquinolone-resistant	Medium

Data analysis plan

The plan for analyzing the data for the MAAP project consists of four steps: (1) identification of key variables, (2) quality assessment of data and parent facility, (3) validation and analysis of data, and (4) reporting of findings.

Variables of interest

A list of patient-level variables was identified as mandatory or optional for the purpose of attaining the project objectives (Table 2). While 'optional' variables would be particularly useful, they may not be available from the lab and thus are not considered a mandatory element to complete the project. Data collection will begin in the laboratory and then continue at the accompanying clinic/hospital to obtain additional information on patients' demographics, clinical profile, and antimicrobial usage. A decision framework will guide the collection of patients' demographic and clinical variables from the clinic/hospital (Figure 1).

In addition to patient variables, information will also be collected on the structure and processes at the laboratory and the accompanying clinic/hospital.

Table 2: Variables of interest

	Variables	Mandatory/ Optional
	**Patient laboratory variables**	
1	Patient code	Mandatory
2	Specimen type (name)	Mandatory
3	Specimen site	Mandatory
4	Date of specimen collection	Mandatory
5	Culture results – (no growth/contaminated/pathogen name)	Mandatory
6	AST Results	Mandatory
7	AST Standard	Mandatory
10	Resistance mechanism-if available	Optional
	**Patient demographic variables**	
1	Patient code	Mandatory
2	Patient gender	Mandatory
3	Patient age or date of birth	Mandatory
4	Patient location	Mandatory
5	Patient department/specialty	Mandatory
6	Patient admission date	Optional
7	Patient discharge date	Optional
8	Patient level of education	Optional
9	Patient weight and height	Optional
10	Pregnancy status	Optional
11	Premature birth	Optional
12	Whether the patient was transferred from another clinical set-up?	Optional
	**Patient clinical/health variables**	
1	Chief complaint	Mandatory
2	Primary diagnosis at admission	Mandatory
3	ICD code at admission	Mandatory
4	Comorbidities	Optional

5	Whether antibiotics were prescribed to patient prior to sampling; antibiotic(s) name & duration	Optional
6	Was the patient on an indwelling medical device at time of sampling; type of device	Optional
7	Origin of infection - community acquired or hospital acquired	Optional
8	Discharge diagnosis	Optional
9	ICD code at discharge	Optional
10	Patient outcome at discharge (recovered/deteriorated/dead/others)	Optional
	**Laboratory-specific variables** (as available from eligibility questionnaires, Appendix A, Table A1)	
1	Laboratory's level of service (Reference- tier 3 or 4/ Regional/ Intermediate/ District/ Community/ Other	Mandatory
2	Laboratory's affiliation (Government/Ministry of Health/ Private/Non-government organization/ Other)	Mandatory
3	Laboratory co-location with clinic/hospital/pharmacy	Mandatory
4	If laboratory served as a national AMR surveillance site at any time between 2016 and 2018?	Mandatory
5	Facility & Equipment related variables	Mandatory
6	Quality Assurance (QA), accreditation & certification related variables	Mandatory
7	Personnel & training related variables	Mandatory
8	Specimen management related variables	Mandatory
9	Laboratory information system & linkage to clinical data	Mandatory
	**Facility-specific variables** (facility denotes co-located clinic/hospital or even from stand-alone laboratory as applicable; this information is obtained during phase of data collection)	
1	Ownership of facility (public/private/partnership/mission/military etc)	Optional
2	Level of facility (primary, secondary, tertiary)	Optional
3	Facility co-location with pharmacy/lab	Optional
4	Number of inpatient beds in 2018 (and prior years as applicable)	Optional
5	Admissions in 2018 (and prior years as applicable)	Optional
6	Outpatients in 2018 (and prior years as applicable)	Optional
7	Presence of ID Department	Optional
8	No of ID physicians	Optional
9	No of ID nurses	Optional
10	Presence of AMS program	Optional
11	Frequency of AMS meetings	Optional
12	Presence of Medical therapeutic committee (MTC)	Optional
13	Frequency of MTC meet	Optional
14	Presence of HIC committee	Optional
15	Frequency of HIC meet	Optional
16	Number of bacterial cultures processed in 2018 (and prior years as applicable)	Optional
17	Number of fungal cultures processed in 2018 (and prior years as applicable)	Optional
18	Number of positive cerebrospinal fluid cultures in 2018 (and prior years as applicable)	Optional
19	Number of positive blood cultures in 2018 (and prior years as applicable)	Optional
20	Format for storing patient lab records	Optional
21	Format for storing patient clinical records	Optional

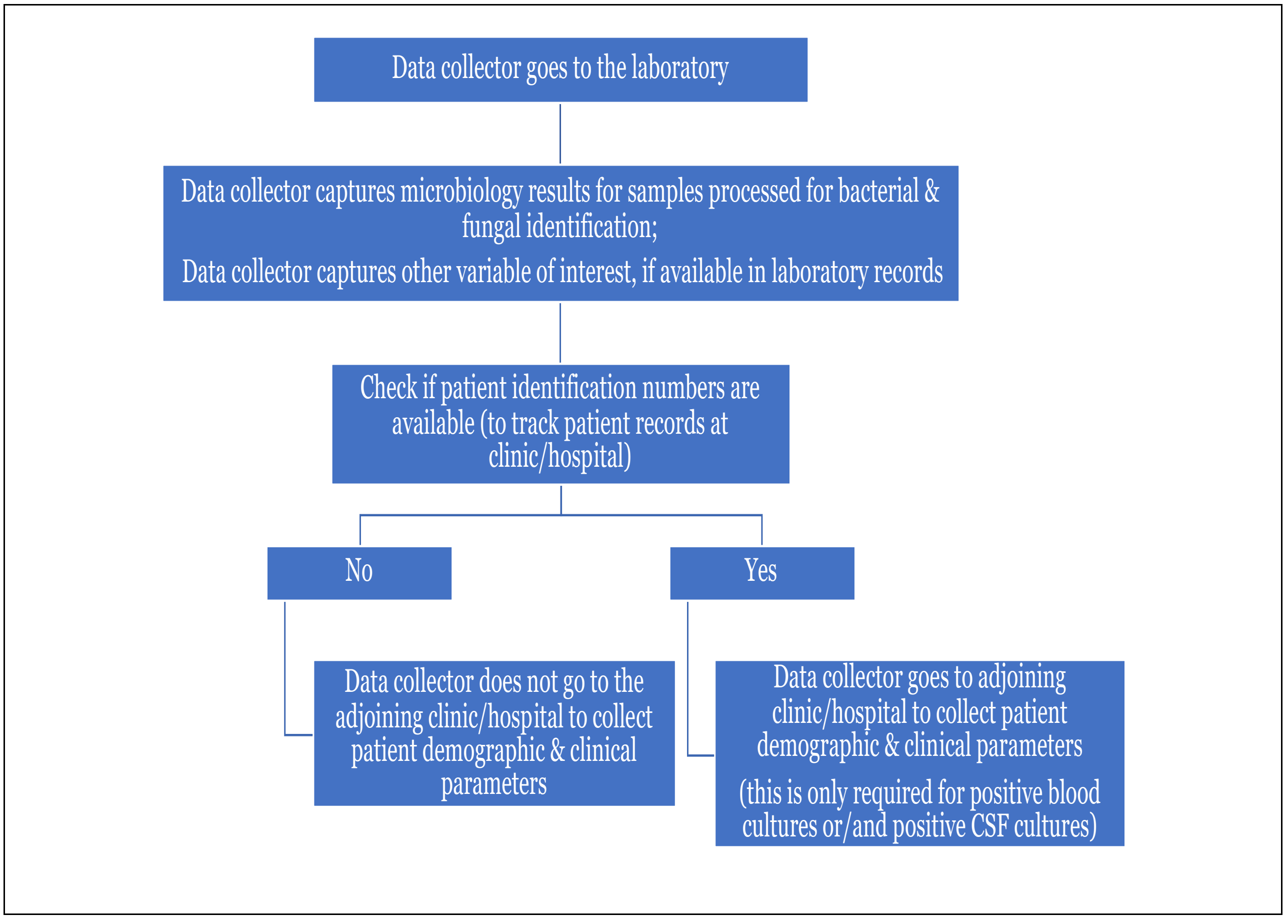

Figure 1: Decision framework

Quality Assessment

The quality of the laboratory will be evaluated based on the information in the eligibility questionnaire (EQ), which each laboratory completed during the selection process. Data quality will be assessed through an evaluation of laboratory import files and will cover parameters such as the completeness and consistency of uploaded data. See **Appendix A** for the complete EQ and **Appendix B** for the quality indicators.

Validation and Analysis of Data

Data validation

The first step in the data analysis process will involve validating the uploaded data files to ensure they accurately capture all required data elements and that the guidance documents are consistent.

Definition of antimicrobial susceptibility

Data capture of resistance interpretation is based on locally relevant standards for resistance in the laboratory. Each lab will submit resulting data and resistance interpretation as determined by the laboratories according to the AST Standard in use in the lab: Clinical and Laboratory Standards Institute (CLSI), European Committee on Antimicrobial Susceptibility Testing (EUCAST), and CASFM-EUCAST (Comité de l'antibiogramme de la Société Française de Microbiologie-European Committee on Antimicrobial Susceptibility Testing). Where labs have provided quantitative results, we will evaluate compliance with standards and re-adjudicate as necessary based on updates to the standards. We will classify all isolates as susceptible or non-susceptible (i.e., we will include intermediate results as resistant).

Data analysis and indicators

Resistance rates will be calculated as the proportion of non-susceptible isolates over the unit of time. The primary analysis will focus on the year level, but where applicable and data allows, seasonal analyses will be conducted using monthly rates. Resistance rates will be determined at the country and regional levels, first for the WHO priority pathogens and then for other significant pathogens that may be important at the country level.

Duplicate removal

Following Clinical and Laboratory Standards Institute (CLSI) criteria[1], we will include only the first isolate per pathogen per patient for the period in question (year 2018), irrespective of body site or antimicrobial susceptibility profile. Pathogens isolated from fewer than 30 patients will be excluded from analysis. Patient identifiers are incorporated into the data collection process. In the event that no patient identifiers are available, we will include all isolate results. A secondary analysis will assess non-susceptibility using two common alternative methods: (i) including all isolates, (ii) including only the most resistant isolate from a patient[2]. We will assess the periods of study for removal of duplicate isolates over the entire time period, as well as, on a year-by-year basis where applicable.

Proportion non-susceptible

Results will be calculated first for the WHO priority pathogens and then for other important pathogens that may be significant at the country level. Rates using only bloodstream and cerebrospinal fluid will serve as the primary analysis unit, as these represent the isolates least likely to contain contaminants. These results will be compared with other specimen sources as well as overall totals, depending on data availability. Results will be reported as the proportion resistant (Table 3) for the country, and where appropriate surveillance exists, by country region/state.

Clinical syndromes

Where available, we will calculate non-susceptibility rates across clinical syndromes based on data availability. The clinical syndromes of interest will include, though not be limited to, respiratory tract infections, infective endocarditis, intravascular catheter-related infections, central nervous system infections, urinary tract infections, soft-tissue infections, osteomyelitis, gastrointestinal infections, sexually transmitted infections, and zoonotic infections.

Table 3: Metrics

**Metrics**			
**Metrics based on specimens sent routinely to laboratories for clinical purposes**			
Indicator	Numerator	Denominator	Example
Cultures per 100,000 inhabitants per year	Number of cultures in a year	Population in the same year	Number of urinary cultures per 100, 000 inhabitants
Proportion of patients with growth of resistant bacteria per specimen type	Number of sampled patients with growth of antibiotic resistant bacteria per specimen type	Number of sampled patients per specimen type	Number of sampled patients with E. coli resistant to fluoroquinolones out of all patients sampled for blood culture
Proportion of sampled patients with positive culture per specimen type	Number of sampled patients with positive culture per specimen type	Number of sampled patients per specimen type	Number of patients sampled with positive blood cultures out of all patients sampled for blood culture
Proportion of samples with growth of antibiotic resistant bacteria per specimen type	Number of samples with antibiotic resistant bacteria	Number of samples with growth of bacteria	Proportion of E. coli resistant to fluoroquinolones per specimen type
**Metrics based on clinical syndromes**			
Indicator	Numerator	Denominator	Example
Prevalence of monitored infections (regardless of pathogen) in the population	Total number of infection episodes that fulfil case definitions verified by bacterial culture	Population covered by participating sites	Number of bloodstream infections per 100000 inhabitants or further specified per age group, gender etc.
Prevalence of infection in the population per syndrome per organism under surveillance	Total number of infections caused by the species	Population covered by participating sites	Number of bloodstream infections caused by E. coli per 100000 population
Prevalence of resistant infections per syndrome per organism under surveillance	Number of infections caused by antibiotic resistant bacteria	Population served by participating sites	Number of bloodstream infections caused by E. coli resistant to fluoroquinolones per 100000 population
Prevalence of infections caused by non-susceptible pathogens per syndrome, pathogen and antibiotic	Number of infections caused by non-susceptible bacteria of the species and antibiotic type under surveillance	Total number of infections caused by the species and tested for the antibiotic under surveillance	Proportion of E. coli resistant to fluoroquinolones of all E. coli that cause bloodstream infections and have been tested for fluoroquinolones

Statistical analysis plan for AMR

Confidence Intervals (CIs) will be calculated to quantify the uncertainty in the estimated resistance rates at the 95% level. CIs will be constructed to account for the considerable variation in quality between data sources (i.e., facility quality, testing methods) as well as the heterogeneity of the population (e.g., socio-demographic status of people visiting the lab/hospital, population catchment).

Typically, CIs for antimicrobial susceptibility data have been constructed using the Wilson Score method 3, 4. This is a binomial calculation that assumes all samples are independent 5. However, the likelihood of resistance is unlikely to be binomial, and there are likely correlations in the data within each lab as well as among labs that draw from similar populations. To account for this lack of data independence, we will use cluster robust confidence intervals, treating each laboratory as a cluster. Preliminary analysis by our team has found that when estimating the proportion of non-susceptibility in a sub-sample from a large set of susceptibility isolates, this method more consistently generates a confidence interval that encompasses the proportion of the whole sample (Appendix C).

Statistical Analysis Plan for Drivers of Resistance

Association between AMR and its potential drivers, at the patient and country-level will examined. This will cover the following factors:

- Socioeconomic and cultural (e.g. education level, financial well-being)
- Structural (e.g. physician density)
- Clinical/health (ex: disease prevalence)
- Antibiotic consumption

The association will be examined by using univariate and multivariate analysis, as described below:

1. Examining the association between AMR rates and qualitative /categorical variables- Qualitative or categorical data can be binary, nominal, or ordinal. Binary variables are characterized by only two possible values (i.e., sex could be male or female). Nominal variables are unordered categorical variables, such as patient location or comorbidity, whereas ordinal variables consist of ordered categories, such as education level (low, medium, high). The association between these variables and AMR rates will be tested using Fisher's exact test or Chi-square test, depending on the sample size.

   To further evaluate any found associations between AMR rates and risk factors, we will calculate the Odds Ratio (OR) for the different categories, where OR < 1.0 represents a negative association and an OR > 1.0 represents a positive association. Logistic regression will be performed between estimated AMR rates and the categorical variables, to obtain the odds ratios, as well as the 95% CI of the OR. Variables with a significant association ($P<0.05$) whose 95% CI for OR does not include the value 1.0 will be selected for further analysis. Additionally, stratification may be performed to obtain adjusted OR, to test for any potential confounders.

2. Examining the association between AMR rates and continuous variables-
   For scalar variables such as weight, height, and age, a linear correlation analysis will be performed to reveal any underlying associations with AMR rates. Pearson's correlation coefficient will be utilized to determine the degree of association ($P<0.05$).

3. Estimate independent AMR drivers-

To adjust for confounders, multivariate logistic regression will be performed on all variables that exhibited a significant association in the logistic regression analysis ($P<0.05$). Significant variables will be regarded as the independent AMR drivers.

Drug Resistance Index

The problem of antibiotic resistance is complicated by the various drugs and their differing levels of efficacy against pathogens. To mitigate these communication challenges, we developed the Drug Resistance Index (DRI) to provide a simple tool for measuring the effectiveness of antibiotic therapy by country. The DRI resembles composite price indices used in economics: it combines measurements of antibiotic consumption and resistance across multiple pathogen–organism combinations to create a single metric representing an aggregate level of drug resistance.

Similar to stock market indices that aggregate market valuations across companies of similar size or from specific sectors, different indices can be created at various geographical levels, ranging from hospital to country, as well as for different types of bacterial infections (e.g., skin and soft tissue infections or Gram-negative infections). We will generate estimates of the DRI for each country using previously published methodology. Briefly, we will compute a composite index score for the overall period and by year by multiplying the proportion of each antibiotic used during the period to treat a set of pathogens by the proportion of all isolates tested during that time period that were resistant to that drug. The resulting score ranges from 0 to 100, where 0 indicates 100% susceptibility and 100 indicates 100% resistance. The following equation will be used to calculate the DRI for each country:

$$DRI = \sum_{k} \rho_k^t q_k^t$$

where ρ_k^t The proportion of resistance among all included pathogens to drug k for time t, and q_k^t is the proportion of drug k used for the treatment of those pathogens in all drugs included in the index for time t. We will calculate country-level DRIs for the WHO priority pathogens.

Appendices

Appendix A: Laboratory Eligibility Questionnaire

The Laboratory Eligibility Questionnaire (EQ) is composed of 6 sections informing on the laboratory affiliation, capacity and quality (Table A). These are (1) Site Information (general structure and function) (2) Facility & Equipment Status (3) Quality Assurance, Accreditation and Certification status (4) Human Resources & Training (5) Specimen Management System (6) Laboratory Information System & Linkage to Clinical Data.

Table A: Laboratory Eligibility Questionnaire

Table A				
**Question**		**Response**	**Exclusion/ Prioritization Criteria**	**Scoring Instructions**
**Part 1: Site Information**				
1	What is the name of the laboratory?			None
2	Between 2016 and 2018, did the laboratory routinely conduct antimicrobial susceptibility testing?	☐ Yes ☐ No	Exclusion criteria 1: Exclude if answer "No" to Part 1, Question 2	Score 0 if "No" Score 1 if "Yes"
3	Is the laboratory willing to share 2016-2018 AST results with the MAAP consortium?	☐ Yes ☐ No	Exclusion criteria 2: Exclude if answer "No" to Part 1, Question 3	Score 0 if "No" Score 1 if "Yes"
4	What is the address of the laboratory?[1]			None
5	What is the laboratory's level of service?[2]	☐ Reference- tier 3 or 4<br>☐ Regional/Intermediate<br>☐ District or community<br>☐ Other		None
6	What is the laboratory's affiliation?[3]	☐ Government/Ministry of Health<br>☐ Private<br>☐ Non-government organization<br>☐ Other		None
7	Is the laboratory co-located in a clinical facility?	☐ Yes ☐ No	Prioritization criteria: Answer "Yes" to Part 1, Question 7	Score 1 for "Yes" and 0 for "No"
8	Is a pharmacy co-located with the laboratory?	☐ Yes ☐ No	Prioritization criteria: Answer "Yes" to Part 1, Question 8	Score 1 for "Yes" and 0 for "No"
9	Did the laboratory serve as a national AMR surveillance site at any time between 2016 and 2018?	☐ Yes ☐ No	Prioritization criteria: Answer "Yes" to Part 1, Question 9	Score 1 for "Yes" and 0 for "No"
10	Is your country participating in World Health Organization's Global Antimicrobial Resistance Surveillance System (WHO GLASS)?	☐ Yes ☐ No		None
**Part 2. Facility & Equipment**				
1	Did the laboratory have the following in place, at any time between 2016-18?		Prioritization criteria: Answer "Yes" to any sub-questions of Part 2, Question 1	Score 1/5 for each sub-question answered "Yes" and 0 for "No." Sum score (out of 1 total possible
	• Regular power supply with functional back up	☐ Yes ☐ No		
	• Continuous water supply	☐ Yes ☐ No		
	• Certified and functional biosafety cabinet	☐ ☐		

[1] Exact address preferred, nearest landmark or street intersection acceptable, where applicable
[2] More than one response is possible; for 'other' enter response as plain text
[3] More than one response is possible; for 'other' enter response as plain text

		Yes No		point for Part 2, Question 1).
	• Automated methods for bacterial identification	☐ ☐<br>Yes No		
	• Automated methods for antimicrobial susceptibility testing	☐ ☐<br>Yes No		
2	Did the laboratory test for mechanisms of antimicrobial resistance[4] at any time between 2016-2018?	☐ ☐<br>Yes No	Prioritization criteria: Answer "Yes" to Part 2, Question 2	Score 1 for "Yes" and 0 for "No"
**Part 3. Quality Assurance (QA), Accreditation & Certification**				
1A	Was the laboratory implementing quality management systems at any time between 2016-2018?	☐ ☐<br>Yes No	Prioritization criteria: Answer "Yes" to Part 3, Question 1A	Score 1 for "Yes" and 0 for "No"
1B	*If you answered 'yes' to question 1A:*<br><br>What quality management tools did the laboratory utilize? (e.g. LQMS, SLIPTA, SLMTA, mentoring, others)			None
2A	Did the laboratory receive a quality certification at any time between 2016-2018?	☐ ☐<br>Yes No	Prioritization criteria: Answer "Yes" to Part 3, Question 2A	Score 1 for "Yes" and 0 for "No"
2B	*If you answered 'yes' to question 2A:*<br><br>What kind of quality certification did the laboratory receive? (e.g. SLIPTA, College of American pathologists)			None
2C	*If you answered 'yes' to question 2A:*<br><br>What was the laboratory's level of quality certification (e.g. star rating for SLIPTA certified laboratories)?			None
3A	Was the laboratory accredited by a national or international body at any time between 2016-2018?	☐ ☐<br>Yes No	Prioritization criteria: Answer "Yes" to Part 3, Question 3A	Score 1 for "Yes" and 0 for "No"
3B	*If you answered 'yes' to question 3A:*<br><br>What was the name of the accreditation body/bodies?			None
4	Did the laboratory participate in an inter laboratory comparison or external quality assessment (EQA) scheme for pathogen identification and AST at any time between 2016-18?	☐ ☐<br>Yes No	Prioritization criteria: Answer "Yes" to Part 3, Question 4	Score 1 for "Yes" and 0 for "No"
5	Did the laboratory utilize reference strains to verify that stains, reagents, and media are working correctly at any time between 2016-18?	☐ ☐<br>Yes No	Prioritization criteria: Answer "Yes" to Part 3, Question 5	Score 1 for "Yes" and 0 for "No"
6	Did the laboratory maintain records of QC results, at any time between 2016-18?	☐ ☐<br>Yes No	Prioritization criteria: Answer "Yes" to Part 3, Question 6	Score 1 for "Yes" and 0 for "No"
7	Was there a quality focal person in your laboratory at any time between 2016-2018?	☐ ☐<br>Yes No	Prioritization criteria: Answer "Yes" to Part 3, Question 7	Score 1 for "Yes" and 0 for "No"
8	Did the laboratory follow standard operating procedures (SOPs) on pathogen identification and AST methodology at any time between 2016-18?	☐ ☐<br>Yes No	Prioritization criteria: Answer "Yes" to Part 3, Question 8	Score 1 for "Yes" and 0 for "No"
9	Did the laboratory comply to any standards (e.g. CLSI, EUCAST, others) for reporting AST results at any time between 2016-18?	☐ ☐<br>Yes No	Prioritization criteria: Answer "Yes" to Part 3, Question 9	Score 1 for "Yes" to any standard and 0 for "No."
**Part 4. Personnel & Training**				
1	Did the laboratory have the following in place at any time between 2016-18?		Prioritization criteria: Answer "Yes" to any sub-question of Part 4, Question 1	Score 1/3 for each sub-question answered "Yes" and 0 for "No." Sum score (out of 1 total possible point for Part 4, Question 1).
	a. At least one qualified microbiologist[5]	☐ ☐<br>Yes No		
	b. A laboratory scientist/technologist /technician experienced in microbiology with skill set in bacteriology	☐ ☐<br>Yes No		
	c. Up to date complete records on staff training and competence record for the microbiology tests they perform	☐ ☐<br>Yes No		

[4] Mechanisms of antimicrobial resistance can be varied. Common mechanisms are production of enzymes (such as extended spectra beta lactamase, carbapenemase, others) and resistance genes (such as mec gene in MRSA, others)

[5] Possesses a postgraduation in microbiology (medical or non-medical)

**Part 5. Specimen Management**				
1	Did the laboratory follow a defined standard operating procedure (SOP) for specimen collection and testing, at any time between 2016-18?	☐ Yes ☐ No	Prioritization criteria: Answer "Yes" to Part 5, Question 1	Score 1 for "Yes" and 0 for "No"
2	Did the laboratory comply to specimen rejection criteria for rejecting inadequate specimen, at any time between 2016-18?	☐ Yes ☐ No	Prioritization criteria: Answer "Yes" to Part 5, Question 2	Score 1 for "Yes" and 0 for "No"
3A	Does the laboratory have information on average number of specimens processed for culture and sensitivity in 2018?	☐ Yes ☐ No	Prioritization criteria: Answer "Yes" to Part 5, Question 3A	Score 1 for "Yes" and 0 for "No"
3B	*If you answered 'yes' to question 3A:*<br><br>What was the average number of specimens processed for bacterial culture in 2018?		Prioritization criteria: Included in 'document on laboratory selection'	Score 0-3 for "3C/3B" ratio
3C	*If you answered 'yes' to question 3A:*<br><br>What was the average number of specimens that yielded bacterial growth and were processed for susceptibility tests, in 2018?		Prioritization criteria: Included in 'document on laboratory selection'	Score 0-3
**Part 6. Laboratory Information System & Linkage to Clinical Data**				
1	Was a specimen (laboratory) identification number assigned to patient specimens received between 2016-18?	☐ Yes ☐ No	Clinical exclusion criteria for patient-level data collection: Exclude laboratory as candidate for patient-level data collection if answer "No" to Part 6, Question 1	Score 1 for "Yes" and 0 for "No"
2A	Was there a system/database to store patient data (demographic, clinical & specimen) at any time between 2016-18?	☐ Yes ☐ No	Clinical prioritization criteria for patient-level data collection: Prioritize laboratory as candidate for patient-level data collection if answer "Yes" to Part 6, Question 2A	Score 1 for "Yes" and 0 for "No"
2B	*If you answered 'yes' to question 2A:*<br><br>What type of data was captured in the system/database?[6]	☐ Patient demographic data (i.e. age, date of birth, gender, location)<br>☐ Patient clinical data (i.e. primary/chief diagnosis, comorbidities, current antibiotic treatment)<br>☐ Patient outcome		None
2C	*If you answered 'yes' to question 2A:*<br><br>What was the format for storage of information?[7]	☐ Paper-based<br>☐ Electronic (laboratory information system, hospital information system, other databases e.g. WHONET)<br>☐ Other		None
2D	*If you answered 'yes' to question 2A:*<br><br>What is the location of this database, or where can this database be accessed from?	☐ Laboratory<br>☐ Clinical facility<br>☐ Other		None
3A	Were patient demographics and clinical information captured on test request forms at any time between 2016-18?	☐ Yes ☐ No		None
3B	*If you answered 'yes' to question 3A:*<br><br>Were test request forms submitted between 2016 and 2018 stored and retrievable?	☐ Yes ☐ No		None

[6] More than one response is possible; for 'other' enter response as plain text
[7] More than one response is possible; for 'other' enter response as plain text

Appendix B: Quality Indicators

Laboratory quality will be assessed based on the responses provided in the EQs. Table B1 lists the laboratory quality indicators and the proposed scoring. The quality of data will be assessed through an analysis of laboratory import files. Table B2 lists the data quality indicators and the proposed scoring. Additionally, information on policies and systems at the clinic or hospital will also serve as indicators of antimicrobial stewardship practices (Table B3).

Table B1: Laboratory quality indicators

**Table B1**				
**Question/Category**		**Response**	**Proposed scoring**	**Indicator type**
**Facility & Equipment (Part 2 of EQ)**				
1	Did the laboratory have the following in place, at any time between 2016-18?			Laboratory quality
	• Regular power supply with functional back up	☐ Yes ☐ No	Score .2 if "Yes" and 0 for "No."	
	• Continuous water supply	☐ Yes ☐ No	Score .2 if "Yes" and 0 for "No."	
	• Certified and functional biosafety cabinet	☐ Yes ☐ No	Score .2 if "Yes" and 0 for "No."	
	• Automated methods for bacterial identification	☐ Yes ☐ No	Score .2 if "Yes" and 0 for "No."	
	• Automated methods for antimicrobial susceptibility testing	☐ Yes ☐ No	Score .2 if "Yes" and 0 for "No."	
2	Did the laboratory test for mechanisms of antimicrobial resistance[8] at any time between 2016-2018?	☐ Yes ☐ No	Score 1 if "Yes" and 0 for "No."	Laboratory quality
**Quality Assurance (QA), Accreditation & Certification (Part 3 of EQ)**				
1	Was the laboratory implementing quality management systems at any time between 2016-2018?	☐ Yes ☐ No	Score 1 if "Yes" and 0 for "No."	Laboratory quality
2	Did the laboratory receive a quality certification at any time between 2016-2018?	☐ Yes ☐ No	Score 1 if "Yes" and 0 for "No."	Laboratory quality
3	Was the laboratory accredited by a national or international body at any time between 2016-2018?	☐ Yes ☐ No	Score 1 if "Yes" and 0 for "No."	Laboratory quality
4	Did the laboratory participate in an inter laboratory comparison or external quality assessment (EQA) scheme for pathogen identification and AST at any time between 2016-18?	☐ Yes ☐ No	Score 1 if "Yes" and 0 for "No."	Laboratory quality
5	Did the laboratory utilize reference strains to verify that stains, reagents, and media are working correctly at any time between 2016-18?	☐ Yes ☐ No	Score 1 if "Yes" and 0 for "No."	Laboratory quality
6	Did the laboratory maintain records of QC results, at any time between 2016-18?	☐ Yes ☐ No	Score 1 if "Yes" and 0 for "No."	Laboratory quality
7	Was there a quality focal person in your laboratory at any time between 2016-2018?	☐ Yes ☐ No	Score 1 if "Yes" and 0 for "No."	Laboratory quality
8	Did the laboratory follow standard operating procedures (SOPs) on pathogen identification and AST methodology at any time between 2016-18?	☐ Yes ☐ No	Score 1 if "Yes" and 0 for "No."	Laboratory quality
9	Did the laboratory comply to any standards (e.g. CLSI, EUCAST, others) for reporting AST results at any time between 2016-18?	☐ Yes ☐ No	Score 1 if "Yes" and 0 for "No."	Laboratory quality
**Personnel & Training (Part 4 of EQ)**				
1	Did the laboratory have the following in place at any time between 2016-18?			Laboratory quality
	• At least one qualified microbiologist[9]	☐ Yes ☐ No	Score .33 if "Yes" and 0 for "No."	

[8] Mechanisms of antimicrobial resistance can be varied. Common mechanisms are production of enzymes (such as extended spectra beta lactamase, carbapenemase, others) and resistance genes (such as mec gene in MRSA, others)
[9] Possesses a postgraduation in microbiology (medical or non-medical)

	• A laboratory scientist/technologist /technician experienced in microbiology with skill set in bacteriology	☐ Yes ☐ No	Score .33 if "Yes" and 0 for "No."	
	• Uptodate complete records on staff training and competence record for the microbiology tests they perform	☐ Yes ☐ No	Score .33 if "Yes" and 0 for "No."	
**Specimen Management (Part 5 of EQ)**				
1	Did the laboratory follow a defined standard operating procedure (SOP) for specimen collection and testing, at any time between 2016-18?	☐ Yes ☐ No	Score 1 if "Yes" and 0 for "No."	Laboratory quality
2	Did the laboratory comply to specimen rejection criteria for rejecting inadequate specimen, at any time between 2016-18?	☐ Yes ☐ No	Score 1 if "Yes" and 0 for "No."	Laboratory quality
3	Does the laboratory have information on average number of specimens processed for culture and sensitivity in 2018?	☐ Yes ☐ No	Score 1 if "Yes" and 0 for "No."	Laboratory quality
**Laboratory Information System & Linkage to Clinical Data (Part 6 of EQ)**				
1	Was a specimen (laboratory) identification number assigned to patient specimens received between 2016-18?	☐ Yes ☐ No	Score 1 if "Yes" and 0 for "No."	Laboratory quality

Table B2: Data quality indicators

**Table B2**				
**Question/Category**		**Response**	**Proposed scoring**	**Indicator type**
Patient information				
1	Is age information missing for less than x% of patients	☐ Yes ☐ No	Score .33 if "Yes" and 0 for "No."	Data quality
2	Is gender information missing for less than x% of patients	☐ Yes ☐ No	Score .33 if "Yes" and 0 for "No."	Data quality
3	Is department information missing for less than x% of patients	☐ Yes ☐ No	Score .33 if "Yes" and 0 for "No."	Data quality
Specimen information				
1	Is specimen type information missing for less than y% of patients	☐ Yes ☐ No	Score .5 if "Yes" and 0 for "No."	Data quality
2	Is 'specimen date of collection' information missing for less than y% of patients	☐ Yes ☐ No	Score .5 if "Yes" and 0 for "No."	Data quality
Laboratory information				
1	Number of cultures/IPD beds in year 2018 (prior years, if applicable)	Proportion	To be determined	Data quality
2	Number of blood cultures/total cultures	Proportion	To be determined	Data quality
3	Number of positive blood cultures/total blood cultures	Proportion	To be determined	Data quality
4	Number of CSF cultures/total cultures	Proportion	To be determined	Data quality
5	Number of positive CSF cultures/total CSF cultures	Proportion	To be determined	Data quality
6	Use of a AST standard	☐ Yes ☐ No	Score 1 for "Yes" and 0 for "No	Data quality
7	Detection of resistance mechanisms	☐ Yes ☐ No	Score 1 for "Yes" and 0 for "No	Data quality
8	Patterns of S/I/R	S:I:R	To be determined	Data quality

Table B3: Indicators of antimicrobial stewardship (clinic/hospital information)

**Table B3**			
**Question/Category**	**Response**	**Proposed scoring**	**Indicator type**
Clinic/hospital information			

1	Presence of Infectious Diseases department	☐ Yes ☐ No	To be determined	antimicrobial stewardship
2	Presence of Antimicrobial surveillance program	☐ Yes ☐ No	To be determined	antimicrobial stewardship
3	Presence of Medical therapeutic committee	☐ Yes ☐ No	To be determined	antimicrobial stewardship
4	Presence of Hospital Infection Committee	☐ Yes ☐ No	To be determined	antimicrobial stewardship

References

1 National Committee for Clinical Laboratory Standards Analysis and presentation of cumulative antimicrobial susceptibility test data; approved guideline, document M39-A (ISBN 1-56238-463-5). Wayne (PA): The Committee. (2002).

2 Li, F. et al. Isolate removal methods and methicillin-resistant Staphylococcus aureus surveillance. Emerg. Infect. Dis. (2005). doi:10.3201/eid1110.050162.
