# Supplementary material for: Antimicrobial resistance in Africa: A retrospective analysis of data from 14 countries, 2016–2019
Source: PLoS Med. 2025 Jun 24;22(6):e1004638. doi: 10.1371/journal.pmed.1004638 (PMC12186946; doi:10.1371/journal.pmed.1004638)
Supplement: S1 Table — (PDF) [file pmed.1004638.s003.pdf]

S1 Table: List of authors in the MAAP study group

Author names	Institutions
Deepak Batra	IQVIA
Abdourahmane Sow	West Africa Health Organization, Bobo Diolasso, Burkina Faso
Martin Matu	Eastern, Central and Southern African Health Community
Manuel Moreira	Innovative Support to Emergencies, Diseases and Disasters, Seattle, WA
Nyambura Moremi	National Public Health Laboratory, Tanzania
Tochi Okwor	Nigeria Centers for Disease Control, Nigeria
Joel Fleury Djoba Siawaya	National Public Health Laboratory, Gabon
Saviour Kwame Yevutsey	Ghana Health Service, Ghana
Mugerwa Ibrahim	Central Public Health Laboratories Ministry of Health, Uganda
Watipaso Kasambara	National Public Health Institute of Malawi, Malawi
Raiva Simbi	Directorate of Laboratory Services, Ministry of Health and Child Care, Zimbabwe
Joseph Chizimu	Zambia Public Health Institute, Zambia
Zizwe Cindi	Department of Livestock and Veterinary Services Ministry of Agriculture Eswatini
Susan Githii	National Public Health Laboratory, Kenya
Alain Etoundi	Directorate for the Control of Disease, Epidemics and Pandemics, Cameroon
Moctar Dieye	Directorate of Public Health, Ministry of Health and Social Action, Senegal
Charles Sawadogo	Directorate of Pharmaceuticals, Drugs and Laboratories, Burkina Faso
Abdoul Salaam Ouedraogo	Centre Muraz, Burkina Faso
Joseph Sam Kanu	National Focal Person,Anti-Microbial Resistance (AMR), Sierra Leone
