# Supplementary material for: Antimicrobial resistance in Africa: A retrospective analysis of data from 14 countries, 2016–2019
Source: PLoS Med. 2025 Jun 24;22(6):e1004638. doi: 10.1371/journal.pmed.1004638 (PMC12186946; doi:10.1371/journal.pmed.1004638)
Supplement: S2 Table — (PDF) [file pmed.1004638.s004.pdf]

S2 Table: Laboratory survey tool for AMR detection capacity

	Question	Response
**Part 1: Site Information**		
1.1	What is the name of the laboratory?	
1.2	Between 2016 and 2018, did the laboratory routinely conduct antimicrobial susceptibility testing (AST)?	Y/N
1.3	Is the laboratory willing to share 2016-2018 AST results with the MAAP consortium?	Y/N
1.4	What is the street address of the laboratory?	
1.5	What is the laboratory's level of service?	☐ Reference (tier 3 or 4)<br>☐ Regional/Intermediate<br>☐ District or community<br>☐ Other (*e.g.* private with no tier level)
1.6	What is the laboratory's affiliation?	☐ Government<br>☐ Private-for-profit<br>☐ Private-not-for-profit<br>☐ Other
1.7	Is the laboratory co-located in a clinical facility?	Y/N
1.8	Is a pharmacy co-located with the laboratory?	Y/N
1.9	Did the laboratory serve as a national AMR surveillance site at any time<br>between 2016 and 2018?	Y/N
**Part 2: Checklist**		
1 Commodity & Equipment (6 points)		
2.1.1	Did the laboratory have regular power supply with functional back up, in<br>place at any time between 2016-18?	Y/N
2.1.2	Did the laboratory have continuous water supply, in place at any time between 2016-18?	Y/N
2.1.3	Did the laboratory have certified and functional biosafety cabinet, in place at any time between 2016-18?	Y/N
2.1.4	Did the laboratory have automated methods for bacterial identification,<br>in place at any time between 2016-18?	Y/N
2.1.5	Did the laboratory have automated methods for antimicrobial susceptibility testing, in place at any time between 2016-18?	Y/N
2.1.6	Did the laboratory test for mechanisms of antimicrobial resistance at any<br>time between 2016-2018?	Y/N
2 Quality management systems, Accreditation & Certification (5 points)		
2.2.1	Was the laboratory implementing quality management systems at any time between 2016-2018?	Y/N
2.2.2	*If you answered 'yes' to question 2.2.1:*<br>What quality management tools did the laboratory utilize? (*one point forat least one checked box*)	☐LQMS (WHO)<br>☐SLIPTA (WHO-AFRO)<br>☐SLMTA<br>☐mentoring<br>☐others

2.2.3	Did the laboratory receive a quality certification at any time between 2016-2018?	Y/N
2.2.4	*If you answered 'yes' to question 2.2.3:*<br>What kind of quality certification did the laboratory receive? (e.g., SLIPTA, College of American pathologists)	
2.2.5	*If you answered 'yes' to question 2.2.3:*<br>What was the laboratory's level of quality certification (e.g., star rating for SLIPTA certified laboratories)?	
2.2.6	Was the laboratory accredited by a national or international body at any<br>time between 2016-2018?	Y/N
2.2.7	*If you answered 'yes' to question 2.2.6:*<br>What was the name of the accreditation body/bodies?	
2.2.8	Did the laboratory participate in an inter laboratory comparison orexternal quality assessment (EQA) proficiency panel scheme for<br>pathogen identification and AST at any time between 2016-18?	Y/N
3 Standardization of AST procedures (5 points)		
3.3.1	Did the laboratory utilize reference strains to verify that stains, reagents,<br>and media are working correctly at any time between 2016-18?	Y/N
3.3.2	Did the laboratory maintain records of QC results, at any time between<br>2016-18?	Y/N
3.3.3	Was there a quality focal person in your laboratory at any time between<br>2016-2018?	Y/N
3.3.4	Did the laboratory follow standard operating procedures (SOPs) on pathogen identification and AST methodology at any time between 2016-18?	Y/N
3.3.5	Did the laboratory comply with any standards (e.g., CLSI, EUCAST, others)for reporting AST results at any time between 2016-18?	Y/N
4 Personnel & Training (3 points)		
2.4.1	Did the laboratory have at least one qualified microbiologist, in place at<br>any time between 2016-18?	Y/N
2.4.2	Did the laboratory have a laboratory scientist/technologist /technician experienced in microbiology with skill set in bacteriology, in place at any<br>time between 2016-18?	Y/N
2.4.3	Did the laboratory have up-to-date complete records on staff training and competence record for the microbiology tests they perform, in place<br>at any time between 2016-18?	Y/N
5 Specimen Management (3 points)		
2.5.1	Did the laboratory follow a defined standard operating procedure (SOP)<br>for specimen collection and testing, at any time between 2016-18?	Y/N
2.5.2	Did the laboratory comply with specimen rejection criteria for rejecting<br>inadequate specimens, at any time between 2016-18?	Y/N

2.5.3	Does the laboratory have information on the average number of specimens processed for culture and sensitivity in 2018?	Y/N
6 Laboratory Information System & Linkage to Clinical Data (4 points)		
2.6.1	Was a laboratory identification number assigned to patient specimens<br>received between 2016-18?	Y/N
2.6.2	Was there a system/database to store patient data (demographic, clinical & specimen) at any time between 2016-18?	Y/N
2.6.3	*If you answered 'yes' to question 2.5.2:*<br>What type of data was captured in the system/database?	☐ Patient demographic data(e.g age, date of birth, gender, location)<br>☐ Patient clinical data (e.g: primary/chief diagnosis, comorbidities, current antibiotic treatment<br>☐ Patient outcome
2.6.4	*If you answered 'yes' to question 2.5.2:*	☐ Paper-based<br>☐ Electronic (laboratory information system, hospitalinformation system, other databases e.g., WHONET)

Scoring framework for the questionnaire:

'Y' and 'N' responses were awarded one and zero points, respectively; for **question 1.4**, the exact address was preferred; however, the nearest landmark or street intersection was acceptable, where applicable; for **questions 1.5 and 1.6**, more than one response was possible and 'other,' responses were entered as plain text; **for question 2.1.6** mechanisms of antimicrobial resistance can vary: common mechanisms are the production of enzymes (extended-spectrum beta-lactamase, carbapenemase, etc.) and activation of resistance genes (mecA gene in MRSA, etc.); for **question 2.4.1**, the qualified microbiologist must possess a postgraduate degree in microbiology (medical or non-medical); for **question 2.6.3 & 2.6.4**, more than one response was possible and 'other,' responses were entered as plain text.
