# Supplementary material for: Antimicrobial resistance in Africa: A retrospective analysis of data from 14 countries, 2016–2019
Source: PLoS Med. 2025 Jun 24;22(6):e1004638. doi: 10.1371/journal.pmed.1004638 (PMC12186946; doi:10.1371/journal.pmed.1004638)
Supplement: S3 Table — (PDF) [file pmed.1004638.s005.pdf]

S3 Table: Expected resistant phenotypes, based on the Clinical & Laboratory Standards Institute (CLSI) and the European Committee on Antimicrobial Susceptibility Testing (EUCAST) guidelines

**Organism name**	**Antibiotic**	**Reference AST* Standard**
Achromobacter xylosoxidans ss. xylosoxidans	Ampicillin	CLSI/EUCAST
Achromobacter xylosoxidans ss. xylosoxidans	Amoxicillin	CLSI/EUCAST
Achromobacter xylosoxidans ss. xylosoxidans	Ceftriaxone	CLSI/EUCAST
Achromobacter xylosoxidans ss. xylosoxidans	Cefotaxime	CLSI/EUCAST
Achromobacter xylosoxidans ss. xylosoxidans	Aztreonam	EUCAST
Achromobacter xylosoxidans ss. xylosoxidans	Ertapenem	CLSI/EUCAST
Acinetobacter baumannii	Ampicillin	CLSI/EUCAST
Acinetobacter baumannii	Amoxicillin	CLSI/EUCAST
Acinetobacter baumannii	Amoxicillin/clavulanic acid	CLSI/EUCAST
Acinetobacter baumannii	Aztreonam	CLSI/EUCAST
Acinetobacter baumannii	Ertapenem	CLSI/EUCAST
Acinetobacter baumannii	Trimethoprim	CLSI/EUCAST
Acinetobacter baumannii	Fosfomycin	CLSI/EUCAST
Acinetobacter baumannii	Ceftriaxone	EUCAST
Acinetobacter baumannii	Cefotaxime	EUCAST
Acinetobacter baumannii	Benzylpenicillin	CLSI
Acinetobacter baumannii	Cefalotin	CLSI
Acinetobacter baumannii	Cefazolin	CLSI
Acinetobacter baumannii	Cefuroxime	CLSI
Acinetobacter baumannii	Cefoxitin	CLSI
Acinetobacter baumannii	Cefotetan	CLSI
Acinetobacter baumannii	Clindamycin	CLSI
Acinetobacter baumannii	Fusidic acid	CLSI
Acinetobacter baumannii	Vancomycin	CLSI
Acinetobacter baumannii	Linezolid	CLSI
Acinetobacter baumannii	Erythromycin	CLSI
Acinetobacter baumannii	Azithromycin	CLSI
Acinetobacter baumannii	Clarithromycin	CLSI
Acinetobacter baumannii	Quinupristin/dalfopristin	CLSI
Acinetobacter baumannii	Rifampicin	CLSI
Acinetobacter baumannii	Tetracycline	EUCAST
Acinetobacter baumannii	Doxycycline	EUCAST
Acinetobacter nosocomialis	Ampicillin	EUCAST
Acinetobacter nosocomialis	Amoxicillin	EUCAST
Acinetobacter nosocomialis	Amoxicillin/clavulanic acid	EUCAST
Acinetobacter nosocomialis	Ceftriaxone	EUCAST
Acinetobacter nosocomialis	Cefotaxime	EUCAST

**Organism name**	**Antibiotic**	**Reference AST* Standard**
Acinetobacter nosocomialis	Aztreonam	EUCAST
Acinetobacter nosocomialis	Ertapenem	EUCAST
Acinetobacter nosocomialis	Trimethoprim	EUCAST
Acinetobacter nosocomialis	Fosfomycin	EUCAST
Acinetobacter nosocomialis	Tetracycline	EUCAST
Acinetobacter nosocomialis	Doxycycline	EUCAST
Acinetobacter pittii	Ampicillin	EUCAST
Acinetobacter pittii	Amoxicillin	EUCAST
Acinetobacter pittii	Amoxicillin/clavulanic acid	EUCAST
Acinetobacter pittii	Ceftriaxone	EUCAST
Acinetobacter pittii	Cefotaxime	EUCAST
Acinetobacter pittii	Aztreonam	EUCAST
Acinetobacter pittii	Ertapenem	EUCAST
Acinetobacter pittii	Trimethoprim	EUCAST
Acinetobacter pittii	Fosfomycin	EUCAST
Acinetobacter pittii	Tetracycline	EUCAST
Acinetobacter pittii	Doxycycline	EUCAST
Aeromonas caviae	Ampicillin	EUCAST
Aeromonas caviae	Amoxicillin	EUCAST
Aeromonas caviae	Ampicillin/sulbactam	EUCAST
Aeromonas dhakensis	Ampicillin	EUCAST
Aeromonas dhakensis	Amoxicillin	EUCAST
Aeromonas dhakensis	Ampicillin/sulbactam	EUCAST
Aeromonas dhakensis	Cefoxitin	EUCAST
Aeromonas hydrophila	Ampicillin	EUCAST
Aeromonas hydrophila	Amoxicillin	EUCAST
Aeromonas hydrophila	Ampicillin/sulbactam	EUCAST
Aeromonas veronii	Ampicillin	EUCAST
Aeromonas veronii	Amoxicillin	EUCAST
Aeromonas veronii	Ampicillin/sulbactam	EUCAST
Aeromonas veronii	Ticarcillin	EUCAST
Campylobacter coli	Fusidic acid	CLSI/EUCAST
Campylobacter coli	Trimethoprim	CLSI/EUCAST
Campylobacter coli	Linezolid	EUCAST
Campylobacter coli	Tedizolid	EUCAST
Campylobacter fetus	Fusidic acid	CLSI/EUCAST
Campylobacter fetus	Trimethoprim	CLSI/EUCAST
Campylobacter fetus	Nalidixic acid	CLSI/EUCAST
Campylobacter fetus	Linezolid	EUCAST
Campylobacter fetus	Tedizolid	EUCAST

**Organism name**	**Antibiotic**	**Reference AST* Standard**
Campylobacter jejuni	Fusidic acid	CLSI/EUCAST
Campylobacter jejuni	Trimethoprim	CLSI/EUCAST
Campylobacter jejuni	Linezolid	EUCAST
Campylobacter jejuni	Tedizolid	EUCAST
Chryseobacterium sp.	Ampicillin	EUCAST
Chryseobacterium sp.	Amoxicillin	EUCAST
Chryseobacterium sp.	Amoxicillin/clavulanic acid	EUCAST
Chryseobacterium sp.	Ampicillin/sulbactam	EUCAST
Chryseobacterium sp.	Ticarcillin	EUCAST
Chryseobacterium sp.	Ticarcillin/clavulanic acid	EUCAST
Chryseobacterium sp.	Cefotaxime	EUCAST
Chryseobacterium sp.	Ceftriaxone	EUCAST
Chryseobacterium sp.	Ceftazidime	EUCAST
Chryseobacterium sp.	Aztreonam	EUCAST
Chryseobacterium sp.	Ertapenem	EUCAST
Chryseobacterium sp.	Imipenem	EUCAST
Chryseobacterium sp.	Meropenem	EUCAST
Chryseobacterium sp.	Polymyxin B	EUCAST
Chryseobacterium sp.	Colistin	EUCAST
Citrobacter braakii	Ampicillin	EUCAST
Citrobacter braakii	Amoxicillin	EUCAST
Citrobacter braakii	Amoxicillin/clavulanic acid	EUCAST
Citrobacter braakii	Ampicillin/sulbactam	EUCAST
Citrobacter braakii	Cefazolin	EUCAST
Citrobacter braakii	Cefalotin	EUCAST
Citrobacter braakii	Cefalexin	EUCAST
Citrobacter braakii	Cefadroxil	EUCAST
Citrobacter braakii	Cefoxitin	EUCAST
Citrobacter farmeri	Ampicillin	EUCAST
Citrobacter farmeri	Amoxicillin	EUCAST
Citrobacter farmeri	Ticarcillin	EUCAST
Citrobacter freundii	Ampicillin	CLSI/EUCAST
Citrobacter freundii	Amoxicillin/clavulanic acid	CLSI/EUCAST
Citrobacter freundii	Ampicillin/sulbactam	CLSI/EUCAST
Citrobacter freundii	Cefazolin	CLSI/EUCAST
Citrobacter freundii	Cefalotin	CLSI/EUCAST
Citrobacter freundii	Cefoxitin	CLSI/EUCAST
Citrobacter freundii	Amoxicillin	EUCAST
Citrobacter freundii	Cefalexin	EUCAST
Citrobacter freundii	Cefadroxil	EUCAST

**Organism name**	**Antibiotic**	**Reference AST* Standard**
Citrobacter koseri (diversus)	Ampicillin	CLSI/EUCAST
Citrobacter koseri (diversus)	Ticarcillin	CLSI/EUCAST
Citrobacter koseri (diversus)	Amoxicillin	EUCAST
Citrobacter sedlakii	Ampicillin	EUCAST
Citrobacter sedlakii	Amoxicillin	EUCAST
Citrobacter sedlakii	Ticarcillin	EUCAST
Citrobacter werkmanii	Ampicillin	EUCAST
Citrobacter werkmanii	Amoxicillin	EUCAST
Citrobacter werkmanii	Amoxicillin/clavulanic acid	EUCAST
Citrobacter werkmanii	Ampicillin/sulbactam	EUCAST
Citrobacter werkmanii	Cefazolin	EUCAST
Citrobacter werkmanii	Cefalotin	EUCAST
Citrobacter werkmanii	Cefalexin	EUCAST
Citrobacter werkmanii	Cefadroxil	EUCAST
Citrobacter werkmanii	Cefoxitin	EUCAST
Citrobacter youngae	Ampicillin	EUCAST
Citrobacter youngae	Amoxicillin	EUCAST
Citrobacter youngae	Amoxicillin/clavulanic acid	EUCAST
Citrobacter youngae	Ampicillin/sulbactam	EUCAST
Citrobacter youngae	Cefazolin	EUCAST
Citrobacter youngae	Cefalotin	EUCAST
Citrobacter youngae	Cefalexin	EUCAST
Citrobacter youngae	Cefadroxil	EUCAST
Citrobacter youngae	Cefoxitin	EUCAST
Clostridium innocuum	Vancomycin	CLSI/EUCAST
Clostridium ramosum	Vancomycin	EUCAST
Corynebacterium sp.	Fosfomycin	EUCAST
Elizabethkingia anophelis	Ampicillin	EUCAST
Elizabethkingia anophelis	Amoxicillin	EUCAST
Elizabethkingia anophelis	Amoxicillin/clavulanic acid	EUCAST
Elizabethkingia anophelis	Ampicillin/sulbactam	EUCAST
Elizabethkingia anophelis	Ticarcillin	EUCAST
Elizabethkingia anophelis	Ticarcillin/clavulanic acid	EUCAST
Elizabethkingia anophelis	Cefotaxime	EUCAST
Elizabethkingia anophelis	Ceftriaxone	EUCAST
Elizabethkingia anophelis	Ceftazidime	EUCAST
Elizabethkingia anophelis	Cefepime	EUCAST
Elizabethkingia anophelis	Aztreonam	EUCAST
Elizabethkingia anophelis	Ertapenem	EUCAST
Elizabethkingia anophelis	Imipenem	EUCAST

**Organism name**	**Antibiotic**	**Reference AST* Standard**
Elizabethkingia anophelis	Meropenem	EUCAST
Enterococcus casseliflavus	Ceftobiprole	CLSI/EUCAST
Enterococcus casseliflavus	Ceftaroline	CLSI/EUCAST
Enterococcus casseliflavus	Ceftolozane/tazobactam	CLSI/EUCAST
Enterococcus casseliflavus	Vancomycin	CLSI/EUCAST
Enterococcus casseliflavus	Clindamycin	CLSI/EUCAST
Enterococcus casseliflavus	Quinupristin/dalfopristin	CLSI/EUCAST
Enterococcus casseliflavus	Fusidic acid	CLSI/EUCAST
Enterococcus casseliflavus	Aztreonam	CLSI
Enterococcus casseliflavus	Polymyxin B	CLSI
Enterococcus casseliflavus	Colistin	CLSI
Enterococcus casseliflavus	Nalidixic acid	CLSI
Enterococcus faecalis	Ceftobiprole	CLSI/EUCAST
Enterococcus faecalis	Ceftaroline	CLSI/EUCAST
Enterococcus faecalis	Ceftolozane/tazobactam	CLSI/EUCAST
Enterococcus faecalis	Clindamycin	CLSI/EUCAST
Enterococcus faecalis	Quinupristin/dalfopristin	CLSI/EUCAST
Enterococcus faecalis	Fusidic acid	CLSI/EUCAST
Enterococcus faecalis	Aztreonam	CLSI
Enterococcus faecalis	Polymyxin B	CLSI
Enterococcus faecalis	Colistin	CLSI
Enterococcus faecalis	Nalidixic acid	CLSI
Enterococcus faecium	Ceftobiprole	CLSI/EUCAST
Enterococcus faecium	Ceftaroline	CLSI/EUCAST
Enterococcus faecium	Ceftolozane/tazobactam	CLSI/EUCAST
Enterococcus faecium	Clindamycin	CLSI
Enterococcus faecium	Fusidic acid	CLSI/EUCAST
Enterococcus faecium	Aztreonam	CLSI
Enterococcus faecium	Polymyxin B	CLSI
Enterococcus faecium	Colistin	CLSI
Enterococcus faecium	Nalidixic acid	CLSI
Enterococcus gallinarum	Ceftobiprole	CLSI/EUCAST
Enterococcus gallinarum	Ceftaroline	CLSI/EUCAST
Enterococcus gallinarum	Ceftolozane/tazobactam	CLSI/EUCAST
Enterococcus gallinarum	Vancomycin	CLSI/EUCAST
Enterococcus gallinarum	Clindamycin	CLSI/EUCAST
Enterococcus gallinarum	Quinupristin/dalfopristin	CLSI/EUCAST
Enterococcus gallinarum	Fusidic acid	CLSI/EUCAST
Enterococcus gallinarum	Aztreonam	CLSI
Enterococcus gallinarum	Polymyxin B	CLSI

Organism name	Antibiotic	Reference AST* Standard
Enterococcus gallinarum	Colistin	CLSI
Enterococcus gallinarum	Nalidixic acid	CLSI
Escherichia hermannii	Ampicillin	CLSI/EUCAST
Escherichia hermannii	Ticarcillin	CLSI/EUCAST
Escherichia hermannii	Amoxicillin	EUCAST
Haemophilus influenzae	Fusidic acid	EUCAST
Haemophilus influenzae	Linezolid	EUCAST
Haemophilus influenzae	Tedizolid	EUCAST
Hafnia alvei	Ampicillin	CLSI/EUCAST
Hafnia alvei	Amoxicillin/clavulanic acid	CLSI/EUCAST
Hafnia alvei	Amoxicillin	EUCAST
Hafnia alvei	Polymyxin B	CLSI/EUCAST
Hafnia alvei	Colistin	CLSI/EUCAST
Klebsiella aerogenes	Ampicillin	CLSI/EUCAST
Klebsiella aerogenes	Amoxicillin/clavulanic acid	CLSI/EUCAST
Klebsiella aerogenes	Ampicillin/sulbactam	CLSI/EUCAST
Klebsiella aerogenes	Cefazolin	CLSI/EUCAST
Klebsiella aerogenes	Cefalotin	CLSI/EUCAST
Klebsiella aerogenes	Cefoxitin	CLSI/EUCAST
Klebsiella aerogenes	Amoxicillin	EUCAST
Klebsiella aerogenes	Cefalexin	EUCAST
Klebsiella aerogenes	Cefadroxil	EUCAST
Klebsiella oxytoca	Ampicillin	CLSI/EUCAST
Klebsiella oxytoca	Ticarcillin	CLSI/EUCAST
Klebsiella oxytoca	Amoxicillin	EUCAST
Klebsiella pneumoniae ss. pneumoniae	Ampicillin	CLSI/EUCAST
Klebsiella pneumoniae ss. pneumoniae	Ticarcillin	CLSI/EUCAST
Klebsiella pneumoniae ss. pneumoniae	Amoxicillin	EUCAST
Klebsiella quasipneumoniae	Ampicillin	EUCAST
Klebsiella quasipneumoniae	Amoxicillin	EUCAST
Klebsiella quasipneumoniae	Ticarcillin	EUCAST
Klebsiella variicola	Ampicillin	CLSI/EUCAST
Klebsiella variicola	Ticarcillin	CLSI/EUCAST
Klebsiella variicola	Amoxicillin	EUCAST
Lactobacillus sp.	Vancomycin	CLSI/EUCAST
Lactobacillus sp.	Teicoplanin	EUCAST
Leclercia adecarboxylata	Fosfomycin	EUCAST
Leuconostoc sp.	Vancomycin	EUCAST
Leuconostoc sp.	Teicoplanin	EUCAST
Listeria monocytogenes	Ceftobiprole	EUCAST

**Organism name**	**Antibiotic**	**Reference AST* Standard**
Listeria monocytogenes	Ceftaroline	EUCAST
Listeria monocytogenes	Ceftolozane/tazobactam	EUCAST
Moraxella (Branh.) catarrhalis	Trimethoprim	EUCAST
Moraxella (Branh.) catarrhalis	Linezolid	EUCAST
Moraxella (Branh.) catarrhalis	Tedizolid	EUCAST
Morganella morganii ss. morganii	Ampicillin	CLSI/EUCAST
Morganella morganii ss. morganii	Amoxicillin/clavulanic acid	CLSI/EUCAST
Morganella morganii ss. morganii	Cefazolin	CLSI/EUCAST
Morganella morganii ss. morganii	Cefalotin	CLSI/EUCAST
Morganella morganii ss. morganii	Tigecycline	CLSI
Morganella morganii ss. morganii	Nitrofurantoin	CLSI/EUCAST
Morganella morganii ss. morganii	Polymyxin B	CLSI/EUCAST
Morganella morganii ss. morganii	Colistin	CLSI/EUCAST
Morganella morganii ss. morganii	Amoxicillin	EUCAST
Morganella morganii ss. morganii	Ampicillin/sulbactam	EUCAST
Morganella morganii ss. morganii	Cefalexin	EUCAST
Morganella morganii ss. morganii	Cefadroxil	EUCAST
Neisseria sp.	Trimethoprim	CLSI/EUCAST
Neisseria sp.	Linezolid	EUCAST
Neisseria sp.	Tedizolid	EUCAST
Ochrobactrum anthropi	Ampicillin	EUCAST
Ochrobactrum anthropi	Amoxicillin	EUCAST
Ochrobactrum anthropi	Amoxicillin/clavulanic acid	EUCAST
Ochrobactrum anthropi	Ampicillin/sulbactam	EUCAST
Ochrobactrum anthropi	Ticarcillin	EUCAST
Ochrobactrum anthropi	Ticarcillin/clavulanic acid	EUCAST
Ochrobactrum anthropi	Piperacillin	EUCAST
Ochrobactrum anthropi	Piperacillin/tazobactam	EUCAST
Ochrobactrum anthropi	Ceftriaxone	EUCAST
Ochrobactrum anthropi	Cefotaxime	EUCAST
Ochrobactrum anthropi	Ceftazidime	EUCAST
Ochrobactrum anthropi	Cefepime	EUCAST
Ochrobactrum anthropi	Aztreonam	EUCAST
Ochrobactrum anthropi	Ertapenem	EUCAST
Pediococcus sp.	Vancomycin	EUCAST
Pediococcus sp.	Teicoplanin	EUCAST
Plesiomonas shigelloides	Ampicillin	EUCAST
Plesiomonas shigelloides	Amoxicillin	EUCAST
Plesiomonas shigelloides	Amoxicillin/clavulanic acid	EUCAST
Plesiomonas shigelloides	Ampicillin/sulbactam	EUCAST

Organism name	Antibiotic	Reference AST* Standard
Proteus mirabilis	Tigecycline	CLSI
Proteus mirabilis	Nitrofurantoin	CLSI/EUCAST
Proteus mirabilis	Polymyxin B	CLSI/EUCAST
Proteus mirabilis	Colistin	CLSI/EUCAST
Proteus penneri	Ampicillin	CLSI/EUCAST
Proteus penneri	Cefazolin	CLSI/EUCAST
Proteus penneri	Cefalotin	CLSI/EUCAST
Proteus penneri	Cefuroxime	CLSI/EUCAST
Proteus penneri	Tigecycline	CLSI
Proteus penneri	Nitrofurantoin	CLSI/EUCAST
Proteus penneri	Polymyxin B	CLSI/EUCAST
Proteus penneri	Colistin	CLSI/EUCAST
Proteus penneri	Amoxicillin	EUCAST
Proteus penneri	Cefalexin	EUCAST
Proteus penneri	Cefadroxil	EUCAST
Proteus vulgaris	Ampicillin	CLSI/EUCAST
Proteus vulgaris	Cefazolin	CLSI/EUCAST
Proteus vulgaris	Cefalotin	CLSI/EUCAST
Proteus vulgaris	Cefuroxime	CLSI/EUCAST
Proteus vulgaris	Tigecycline	CLSI
Proteus vulgaris	Nitrofurantoin	CLSI/EUCAST
Proteus vulgaris	Polymyxin B	CLSI/EUCAST
Proteus vulgaris	Colistin	CLSI/EUCAST
Proteus vulgaris	Amoxicillin	EUCAST
Proteus vulgaris	Cefalexin	EUCAST
Proteus vulgaris	Cefadroxil	EUCAST
Providencia rettgeri	Ampicillin	CLSI/EUCAST
Providencia rettgeri	Amoxicillin/clavulanic acid	CLSI/EUCAST
Providencia rettgeri	Cefazolin	CLSI/EUCAST
Providencia rettgeri	Cefalotin	CLSI/EUCAST
Providencia rettgeri	Tigecycline	CLSI
Providencia rettgeri	Nitrofurantoin	CLSI/EUCAST
Providencia rettgeri	Polymyxin B	CLSI/EUCAST
Providencia rettgeri	Colistin	CLSI/EUCAST
Providencia rettgeri	Amoxicillin	EUCAST
Providencia rettgeri	Ampicillin/sulbactam	EUCAST
Providencia rettgeri	Cefalexin	EUCAST
Providencia rettgeri	Cefadroxil	EUCAST
Providencia stuartii	Ampicillin	CLSI/EUCAST
Providencia stuartii	Amoxicillin/clavulanic acid	CLSI/EUCAST

**Organism name**	**Antibiotic**	**Reference AST* Standard**
Providencia stuartii	Cefazolin	CLSI/EUCAST
Providencia stuartii	Cefalotin	CLSI/EUCAST
Providencia stuartii	Tigecycline	CLSI
Providencia stuartii	Nitrofurantoin	CLSI/EUCAST
Providencia stuartii	Polymyxin B	CLSI/EUCAST
Providencia stuartii	Colistin	CLSI/EUCAST
Providencia stuartii	Amoxicillin	EUCAST
Providencia stuartii	Ampicillin/sulbactam	EUCAST
Providencia stuartii	Cefalexin	EUCAST
Providencia stuartii	Cefadroxil	EUCAST
Providencia stuartii urea +	Ampicillin	CLSI
Providencia stuartii urea +	Amoxicillin/clavulanic acid	CLSI
Providencia stuartii urea +	Cefazolin	CLSI
Providencia stuartii urea +	Cefalotin	CLSI
Providencia stuartii urea +	Tigecycline	CLSI
Providencia stuartii urea +	Nitrofurantoin	CLSI
Providencia stuartii urea +	Polymyxin B	CLSI
Providencia stuartii urea +	Colistin	CLSI
Pseudomonas aeruginosa	Ampicillin	CLSI/EUCAST
Pseudomonas aeruginosa	Amoxicillin	CLSI/EUCAST
Pseudomonas aeruginosa	Ampicillin/sulbactam	CLSI/EUCAST
Pseudomonas aeruginosa	Amoxicillin/clavulanic acid	CLSI/EUCAST
Pseudomonas aeruginosa	Cefotaxime	CLSI/EUCAST
Pseudomonas aeruginosa	Ceftriaxone	CLSI/EUCAST
Pseudomonas aeruginosa	Ertapenem	CLSI/EUCAST
Pseudomonas aeruginosa	Tigecycline	CLSI/EUCAST
Pseudomonas aeruginosa	Trimethoprim	CLSI/EUCAST
Pseudomonas aeruginosa	Chloramphenicol	CLSI/EUCAST
Pseudomonas aeruginosa	Kanamycin	EUCAST
Pseudomonas aeruginosa	Neomycin	EUCAST
Pseudomonas aeruginosa	Benzylpenicillin	CLSI
Pseudomonas aeruginosa	Cefalotin	CLSI
Pseudomonas aeruginosa	Cefazolin	CLSI
Pseudomonas aeruginosa	Cefuroxime	CLSI
Pseudomonas aeruginosa	Cefoxitin	CLSI
Pseudomonas aeruginosa	Cefotetan	CLSI
Pseudomonas aeruginosa	Clindamycin	CLSI
Pseudomonas aeruginosa	Fusidic acid	CLSI
Pseudomonas aeruginosa	Vancomycin	CLSI
Pseudomonas aeruginosa	Linezolid	CLSI

**Organism name**	**Antibiotic**	**Reference AST* Standard**
Pseudomonas aeruginosa	Erythromycin	CLSI
Pseudomonas aeruginosa	Azithromycin	CLSI
Pseudomonas aeruginosa	Clarithromycin	CLSI
Pseudomonas aeruginosa	Quinupristin/dalfopristin	CLSI
Pseudomonas aeruginosa	Rifampicin	CLSI
Raoultella sp.	Ampicillin	CLSI/EUCAST
Raoultella sp.	Ticarcillin	CLSI/EUCAST
Raoultella sp.	Amoxicillin	CLSI/EUCAST
Serratia marcescens	Ampicillin	CLSI/EUCAST
Serratia marcescens	Amoxicillin/clavulanic acid	CLSI/EUCAST
Serratia marcescens	Ampicillin/sulbactam	CLSI/EUCAST
Serratia marcescens	Cefazolin	CLSI/EUCAST
Serratia marcescens	Cefalotin	CLSI/EUCAST
Serratia marcescens	Cefoxitin	CLSI/EUCAST
Serratia marcescens	Cefuroxime	CLSI/EUCAST
Serratia marcescens	Nitrofurantoin	CLSI/EUCAST
Serratia marcescens	Polymyxin B	CLSI/EUCAST
Serratia marcescens	Colistin	CLSI/EUCAST
Serratia marcescens	Amoxicillin	EUCAST
Serratia marcescens	Cefalexin	EUCAST
Serratia marcescens	Cefadroxil	EUCAST
Staphylococcus aureus ss. aureus	Ceftazidime	EUCAST
Staphylococcus aureus ss. aureus	Aztreonam	CLSI
Staphylococcus aureus ss. aureus	Polymyxin B	CLSI
Staphylococcus aureus ss. aureus	Colistin	CLSI
Staphylococcus aureus ss. aureus	Nalidixic acid	CLSI
Staphylococcus capitis ss. capitis	Fosfomycin	CLSI/EUCAST
Staphylococcus capitis ss. capitis	Ceftazidime	EUCAST
Staphylococcus capitis ss. capitis	Aztreonam	CLSI
Staphylococcus capitis ss. capitis	Polymyxin B	CLSI
Staphylococcus capitis ss. capitis	Colistin	CLSI
Staphylococcus capitis ss. capitis	Nalidixic acid	CLSI
Staphylococcus cohnii ss. cohnii	Novobiocin	CLSI/EUCAST
Staphylococcus cohnii ss. cohnii	Ceftazidime	EUCAST
Staphylococcus cohnii ss. cohnii	Aztreonam	CLSI
Staphylococcus cohnii ss. cohnii	Polymyxin B	CLSI
Staphylococcus cohnii ss. cohnii	Colistin	CLSI
Staphylococcus cohnii ss. cohnii	Nalidixic acid	CLSI
Staphylococcus epidermidis	Aztreonam	CLSI
Staphylococcus epidermidis	Polymyxin B	CLSI

**Organism name**	**Antibiotic**	**Reference AST* Standard**
Staphylococcus epidermidis	Colistin	CLSI
Staphylococcus epidermidis	Nalidixic acid	CLSI
Staphylococcus haemolyticus	Aztreonam	CLSI
Staphylococcus haemolyticus	Polymyxin B	CLSI
Staphylococcus haemolyticus	Colistin	CLSI
Staphylococcus haemolyticus	Nalidixic acid	CLSI
Staphylococcus lugdunensis	Aztreonam	CLSI
Staphylococcus lugdunensis	Polymyxin B	CLSI
Staphylococcus lugdunensis	Colistin	CLSI
Staphylococcus lugdunensis	Nalidixic acid	CLSI
Staphylococcus saprophyticus ss. saprophyticus	Novobiocin	CLSI/EUCAST
Staphylococcus saprophyticus ss. saprophyticus	Fosfomycin	CLSI/EUCAST
Staphylococcus saprophyticus ss. saprophyticus	Fusidic acid	CLSI/EUCAST
Staphylococcus saprophyticus ss. saprophyticus	Ceftazidime	EUCAST
Staphylococcus saprophyticus ss. saprophyticus	Aztreonam	CLSI
Staphylococcus saprophyticus ss. saprophyticus	Polymyxin B	CLSI
Staphylococcus saprophyticus ss. saprophyticus	Colistin	CLSI
Staphylococcus saprophyticus ss. saprophyticus	Nalidixic acid	CLSI
Staphylococcus xylosus	Novobiocin	CLSI/EUCAST
Staphylococcus xylosus	Ceftazidime	EUCAST
Staphylococcus xylosus	Aztreonam	CLSI
Staphylococcus xylosus	Polymyxin B	CLSI
Staphylococcus xylosus	Colistin	CLSI
Staphylococcus xylosus	Nalidixic acid	CLSI
Stenotrophomonas maltophilia	Ampicillin	CLSI/EUCAST
Stenotrophomonas maltophilia	Amoxicillin	CLSI/EUCAST
Stenotrophomonas maltophilia	Piperacillin	CLSI/EUCAST
Stenotrophomonas maltophilia	Ticarcillin	CLSI/EUCAST
Stenotrophomonas maltophilia	Ampicillin/sulbactam	CLSI/EUCAST
Stenotrophomonas maltophilia	Amoxicillin/clavulanic acid	CLSI/EUCAST
Stenotrophomonas maltophilia	Piperacillin/tazobactam	CLSI/EUCAST
Stenotrophomonas maltophilia	Cefotaxime	CLSI/EUCAST
Stenotrophomonas maltophilia	Ceftriaxone	CLSI/EUCAST
Stenotrophomonas maltophilia	Aztreonam	CLSI/EUCAST
Stenotrophomonas maltophilia	Imipenem	CLSI/EUCAST
Stenotrophomonas maltophilia	Meropenem	CLSI/EUCAST
Stenotrophomonas maltophilia	Ertapenem	CLSI/EUCAST
Stenotrophomonas maltophilia	Trimethoprim	CLSI
Stenotrophomonas maltophilia	Fosfomycin	CLSI/EUCAST
Stenotrophomonas maltophilia	Tetracycline	CLSI/EUCAST

**Organism name**	**Antibiotic**	**Reference AST* Standard**
Stenotrophomonas maltophilia	Benzylpenicillin	CLSI
Stenotrophomonas maltophilia	Cefalotin	CLSI
Stenotrophomonas maltophilia	Cefazolin	CLSI
Stenotrophomonas maltophilia	Cefuroxime	CLSI
Stenotrophomonas maltophilia	Cefoxitin	CLSI
Stenotrophomonas maltophilia	Cefotetan	CLSI
Stenotrophomonas maltophilia	Clindamycin	CLSI
Stenotrophomonas maltophilia	Fusidic acid	CLSI
Stenotrophomonas maltophilia	Vancomycin	CLSI
Stenotrophomonas maltophilia	Linezolid	CLSI
Stenotrophomonas maltophilia	Erythromycin	CLSI
Stenotrophomonas maltophilia	Azithromycin	CLSI
Stenotrophomonas maltophilia	Clarithromycin	CLSI
Stenotrophomonas maltophilia	Quinupristin/dalfopristin	CLSI
Stenotrophomonas maltophilia	Rifampicin	CLSI
Streptococcus sp.	Fusidic acid	EUCAST
Streptococcus sp.	Ceftazidime	EUCAST
Yersinia enterocolitica	Ampicillin	CLSI/EUCAST
Yersinia enterocolitica	Amoxicillin/clavulanic acid	CLSI/EUCAST
Yersinia enterocolitica	Ticarcillin	CLSI/EUCAST
Yersinia enterocolitica	Cefazolin	CLSI/EUCAST
Yersinia enterocolitica	Cefalotin	CLSI/EUCAST
Yersinia enterocolitica	Amoxicillin	EUCAST
Yersinia enterocolitica	Ampicillin/sulbactam	EUCAST
Yersinia enterocolitica	Cefalexin	EUCAST
Yersinia enterocolitica	Cefadroxil	EUCAST
Yersinia enterocolitica	Cefoxitin	EUCAST
Yersinia pseudotuberculosis	Polymyxin B	CLSI/EUCAST
Yersinia pseudotuberculosis	Colistin	CLSI/EUCAST

*AST: Antimicrobial susceptibility testing
