# Supplementary material for: Antimicrobial resistance in Africa: A retrospective analysis of data from 14 countries, 2016–2019
Source: PLoS Med. 2025 Jun 24;22(6):e1004638. doi: 10.1371/journal.pmed.1004638 (PMC12186946; doi:10.1371/journal.pmed.1004638)
Supplement: S4 Table — (PDF) [file pmed.1004638.s006.pdf]

S4 Table: Select pathogens and antimicrobials used in estimating drug resistance index

**Organism name**	**Antimicrobial/Class**
Acinetobacter baumannii	Aminoglycosides
Escherichia coli	Aminoglycosides
Klebsiella pneumoniae	Aminoglycosides
Pseudomonas aeruginosa	Aminoglycosides
Enterococcus faecalis	Aminoglycosides (High)
Enterococcus faecium	Aminoglycosides (High)
Enterococcus faecalis	Aminopenicillins
Enterococcus faecium	Aminopenicillins
Escherichia coli	Aminopenicillins
Haemophilus influenzae	Aminopenicillins
Acinetobacter baumannii	Carbapenems
Escherichia coli	Carbapenems
Klebsiella pneumoniae	Carbapenems
Pseudomonas aeruginosa	Carbapenems
Streptococcus pneumoniae	Carbapenems
Acinetobacter baumannii	Cephalosporins (3rd generation)
Escherichia coli	Cephalosporins (3rd generation)
Klebsiella pneumoniae	Cephalosporins (3rd generation)
Pseudomonas aeruginosa	Cephalosporins (3rd generation)
Haemophilus influenzae	Cephalosporins (3rd generation)
Streptococcus pneumoniae	Cephalosporins (3rd generation)
Acinetobacter baumannii	Fluoroquinolone
Escherichia coli	Fluoroquinolones
Klebsiella pneumoniae	Fluoroquinolones
Pseudomonas aeruginosa	Fluoroquinolones
Streptococcus pneumoniae	Fluoroquinolones
Shigella Species	Fluoroquinolones
Streptococcus pneumoniae	Macrolides
Staphylococcus aureus	Methicillin
Pseudomonas aeruginosa	Beta-lactam combinations
Streptococcus pneumoniae	Penicillin
Enterococcus faecalis	Vancomycin
Enterococcus faecium	Vancomycin
