# Supplementary material for: Antimicrobial resistance in Africa: A retrospective analysis of data from 14 countries, 2016–2019
Source: PLoS Med. 2025 Jun 24;22(6):e1004638. doi: 10.1371/journal.pmed.1004638 (PMC12186946; doi:10.1371/journal.pmed.1004638)
Supplement: S5 Table — (PDF) [file pmed.1004638.s007.pdf]

S5 Table: Multivariable logistic regression analysis between AMR prevalence, prior antibiotic usage, and other factors[1]

Variable	Options	N (R %)	Adjusted Odds Ratio (95% CI)	P-value
Age, years	18-49	702 (45.3)	Ref	
	<1	189 (45.5)	0.79 (0.47 - 1.35)	0.392
	1-17	405 (42)	0.78 (0.56 - 1.1)	0.156
	50-65	137 (48.9)	1.15 (0.75 - 1.76)	0.520
	>65	117 (52.1)	1.34 (0.88 - 2.05)	0.172
Sex	Female	903 (44)	Ref*	
	Male	647 (47.1)	0.91 (0.75 - 1.11)	0.365
Country	Nigeria	223 (57)	Ref	
	Cameroon	158 (44.3)	0.72 (0.26 - 2.04)	0.539
	Gabon	239 (29.7)	0.57 (0.25 - 1.28)	0.173
	Senegal	33 (48.5)	0.64 (0.27 - 1.5)	0.300
	Uganda	897 (46.6)	0.97 (0.42 - 2.25)	0.945
Specimen source	others	1,062 (42.5)	Ref	
	Blood or CSF	488 (51.4)	1.24 (0.69 - 2.25)	0.469
Department	Outpatient	666 (37.1)	Ref	
	Inpatient	884 (51.5)	1.44 (1.19 - 1.75)	<0.0001
Lab tier level	District or Community	6 (50)	Ref	
	Reference	212 (48.1)	1.09 (0.32 - 3.73)	0.896
	Regional or Intermediate	1,332 (44.8)	0.99 (0.36 - 2.71)	0.985
Prior antibiotic usage	No	770 (38.8)	Ref	
	Yes	780 (51.7)	1.37 (1.05 - 1.8)	0.022

N, number of tested isolates; R%, proportion of resistant isolates; Ref, reference category; CSF, cerebrospinal fluid

[1] Includes samples collected from laboratories in Nigeria, Cameroon, Gabon, Senegal, and Uganda.
