# Supplementary material for: Antimicrobial resistance in Africa: A retrospective analysis of data from 14 countries, 2016–2019
Source: PLoS Med. 2025 Jun 24;22(6):e1004638. doi: 10.1371/journal.pmed.1004638 (PMC12186946; doi:10.1371/journal.pmed.1004638)
Supplement: S6 Table — (PDF) [file pmed.1004638.s008.pdf]

S6 Table: Population coverage of the laboratories selected for data collection

Country	Total population	No. of labs that responded to eligibility questionnaire	No. of labs that were enrolled into MAAP project	Population covered* (%)
Burkina Faso	20,321,382	23	16	33
Cameroon	25,876,387	19	16	31
Gabon	2,172,578	20	16	65
Ghana	30,417,858	64	16	39
Kenya	52,573,967	56	16	68
Malawi	18,628,748	15	15	57
Nigeria	200,963,603	73	25	40
Senegal	16,296,361	22	16	47
Sierra Leone	7,813,207	7	7	29
Eswatini	1,148,133	3	3	62
Uganda	44,269,587	20	16	34
Tanzania	58,005,461	27	16	24
Zambia	17,861,034	22	16	42
Zimbabwe	14,645,472	22	14	44

*Population living within 1 hour travel to the laboratories.
