# Supplementary material for: Antimicrobial resistance in Africa: A retrospective analysis of data from 14 countries, 2016–2019
Source: PLoS Med. 2025 Jun 24;22(6):e1004638. doi: 10.1371/journal.pmed.1004638 (PMC12186946; doi:10.1371/journal.pmed.1004638)
Supplement: S7 Table — (PDF) [file pmed.1004638.s009.pdf]

S7 Table: Distribution of collected culture records and data quality scores.

Country	Participating laboratories (N=205)	Total cultures (N=819,584)	Valid Cultures (N=740,310) n (%t)	Positive cultures with AST* results (N= 187,832) n (%v)	Cultures with clinical variables (N=22,716) n (%v)	Cultures with organisms specified at the species level (N=135,972) n (%p)	Data quality score #
Burkina Faso	16	41,544	41,341 (99·5)	7,765 (18.8)	111 (0.3)	6,238 (80.3)	80.2
Cameroon	16	116,808	116,361 (99·6)	32,545 (28)	457 (0.4)	29,311 (90.1)	69.4
Eswatini	3	9,445	9,386 (99·4)	5,247 (55.9)	11 (0.1)	3,802 (72.5)	77.6
Gabon	16	35,119	31,152 (88·7)	8,425 (27)	788 (2.5)	7,681 (91.2)	72.7
Ghana	15	24,427	17,096 (70)	4,394 (25.7)	573 (3.4)	2,861 (65.1)	76.6
Kenya	16	66,835	64,328 (96·2)	16,027 (24.9)	4,231 (6.6)	12,569 (78.4)	69.6
Malawi	15	70,548	65,698 (93·1)	7,196 (11)	-	5,540 (77)	70.2
Nigeria	25	85,127	84,548 (99·3)	23,963 (28.3)	438 (0.5)	15,427 (64.4)	65.7
Senegal	16	78,304	51,771 (66·1)	8,763 (16.9)	3,528 (6.8)	7,910 (90.3)	80.8
Sierra Leone	7	4,333	4,192 (96·7)	723 (17.2)	-	177 (24.5)	56.4
Tanzania	16	77,553	72,587 (93·6)	13,204 (18.2)	-	7,579 (57.4)	73.5
Uganda	16	88,116	85,096 (96·6)	22,349 (26.3)	869 (1)	17,979 (80.4)	76.0
Zambia	14	44,912	33,790 (75·2)	22,343 (66.1)	11,184 (33.1)	14,631 (65.5)	80.1
Zimbabwe	14	76,513	62,964 (82·3)	14,888 (23.6)	526 (0.8)	4,267 (28.7)	72.8

*AST= antimicrobial susceptibility test; — information not available; (%t) Proportion of the total cultures; (%v) Proportion of the valid cultures; (%p) Proportion of the positive cultures; #Data quality score is a measure of the quality of AMR data obtained in the country which was derived from the proportion of test results with defined AST breakpoints, and the proportion of results indicating nonsusceptibility where resistant
