# Supplementary material for: Antimicrobial resistance in Africa: A retrospective analysis of data from 14 countries, 2016–2019
Source: PLoS Med. 2025 Jun 24;22(6):e1004638. doi: 10.1371/journal.pmed.1004638 (PMC12186946; doi:10.1371/journal.pmed.1004638)
Supplement: S8 Table — (PDF) [file pmed.1004638.s010.pdf]

S8 Table: AMR prevalence estimates for WHO priority pathogens

Country	Pathogen	Antibiotic/class	2016 N; R(%R;95%CI)	2017 N; R (%R;95%CI)	2018 N; R(%R;95%CI)	2019 N; R(%R;95%CI)
Burkina Faso	*Acinetobacter baumannii*	Carbapenems	-	-	99;28 (28.3%; 8.2-63.5)	13;1
Burkina Faso	*Campylobacter species*	Fluoroquinolones	-	-	-	-
Burkina Faso	Enterobacterales	Carbapenems	-	-	2526;223 (8.8%; 4-18.3)	173;19 (11%; 8.2-14.6)
Burkina Faso	Enterobacterales	Cephalosporins (3rd generation)	-	-	4273;2032 (47.6%; 37.6-57.7)	185;112 (60.5%; 51.7-68.8)
Burkina Faso	*Enterococcus faecium*	Vancomycin	-	-	-	-
Burkina Faso	*Haemophilus influenzae*	Ampicillin	-	-	-	-
Burkina Faso	*Helicobacter pylori*	Clarithromycin	-	-	-	-
Burkina Faso	*Neisseria gonorrhoeae*	Cephalosporins (3rd generation)	-	-	-	-
Burkina Faso	*Neisseria gonorrhoeae*	Fluoroquinolones	-	-	-	-
Burkina Faso	*Pseudomonas aeruginosa*	Carbapenems	-	-	131;36 (27.5%; 8.5-60.7)	11;0
Burkina Faso	*Salmonella species*	Fluoroquinolones	-	-	64;8 (12.5%; 3.7-34.6)	6;2
Burkina Faso	*Shigella species*	Fluoroquinolones	-	-	74;23 (31.1%; 15.7-52.2)	-
Burkina Faso	*Staphylococcus aureus*	Methicillin	-	-	589;120 (20.4%; 14.9-27.2)	53;4 (7.5%; 0.1-92.5)
Burkina Faso	*Streptococcus pneumoniae*	Beta-lactam combinations	-	-	3;1	-
Burkina Faso	*Streptococcus pneumoniae*	Penicillins	-	-	5;1	-
Cameroon	*Acinetobacter baumannii*	Carbapenems	-	22;10	58;21 (36.2%; 25.9-47.9)	92;27 (29.3%; 12.6-54.6)
Cameroon	*Campylobacter species*	Fluoroquinolones	-	1;1	-	-
Cameroon	Enterobacterales	Carbapenems	-	946;118 (12.5%; 6.4-22.8)	1476;201 (13.6%; 7.5-23.6)	1654;185 (11.2%; 6.1-19.7)
Cameroon	Enterobacterales	Cephalosporins (3rd generation)	1;1	2386;1221 (51.2%; 44.4-57.9)	3384;1905 (56.3%; 51-61.4)	3066;1669 (54.4%; 48.3-60.4)
Cameroon	*Enterococcus faecium*	Vancomycin	-	2;0	1;0	-
Cameroon	*Haemophilus influenzae*	Ampicillin	-	1;1	1;0	-
Cameroon	*Helicobacter pylori*	Clarithromycin	-	-	-	-

Country	Pathogen	Antibiotic/class	2016<br>N; R(%R;95%CI)	2017<br>N; R (%R;95%CI)	2018<br>N; R(%R;95%CI)	2019<br>N; R(%R;95%CI)
Cameroon	*Neisseria gonorrhoeae*	Cephalosporins (3rd generation)	-	43;14 (32.6%; 15.4-56.1)	41;18 (43.9%; 24.3-65.6)	38;10 (26.3%; 9.6-54.7)
Cameroon	*Neisseria gonorrhoeae*	Fluoroquinolones	-	40;13 (32.5%; 12.5-62)	32;18 (56.2%; 26.9-81.8)	22;8
Cameroon	*Pseudomonas aeruginosa*	Carbapenems	-	66;18 (27.3%; 10.9-53.5)	174;33 (19%; 14.2-24.8)	159;40 (25.2%; 14.6-39.9)
Cameroon	*Salmonella species*	Fluoroquinolones	-	41;3 (7.3%; 2.8-17.7)	61;14 (23%; 15-33.5)	76;16 (21.1%; 11.8-34.8)
Cameroon	*Shigella species*	Fluoroquinolones	-	29;4	46;15 (32.6%; 15.8-55.4)	27;8
Cameroon	*Staphylococcus aureus*	Methicillin	-	325;168 (51.7%; 34.7-68.3)	490;304 (62%; 38.7-80.9)	497;310 (62.4%; 52.9-71)
Cameroon	*Streptococcus pneumoniae*	Beta-lactam combinations	-	13;4	3;2	2;0
Cameroon	*Streptococcus pneumoniae*	Penicillins	-	9;7	4;2	5;1
eSwatini	*Acinetobacter baumannii*	Carbapenems	3;0	1;0	8;2	-
eSwatini	*Campylobacter species*	Fluoroquinolones	-	-	4;4	-
eSwatini	Enterobacterales	Carbapenems	435;5 (1.1%; 0-23.4)	344;8 (2.3%; 0.1-52.8)	366;15 (4.1%; 0.1-67.6)	-
eSwatini	Enterobacterales	Cephalosporins (3rd generation)	584;137 (23.5%; 4.3-67.7)	905;342 (37.8%; 10.5-75.9)	545;165 (30.3%; 5.8-75.3)	-
eSwatini	*Enterococcus faecium*	Vancomycin	5;1	10;2	-	-
eSwatini	*Haemophilus influenzae*	Ampicillin	-	1;1	1;0	-
eSwatini	*Helicobacter pylori*	Clarithromycin	-	-	-	-
eSwatini	*Neisseria gonorrhoeae*	Cephalosporins (3rd generation)	9;0	14;0	5;1	-
eSwatini	*Neisseria gonorrhoeae*	Fluoroquinolones	9;1	14;7	5;3	-
eSwatini	*Pseudomonas aeruginosa*	Carbapenems	18;1	11;0	25;1	-
eSwatini	*Salmonella species*	Fluoroquinolones	7;3	5;1	3;1	-
eSwatini	*Shigella species*	Fluoroquinolones	1;0	3;0	3;1	-
eSwatini	*Staphylococcus aureus*	Methicillin	147;29 (19.7%; 0.8-88.1)	282;96 (34%; 3-89.6)	174;94 (54%; 2.7-98)	-
eSwatini	*Streptococcus pneumoniae*	Beta-lactam combinations	1;0	3;0	-	-
eSwatini	*Streptococcus pneumoniae*	Penicillins	2;0	6;3	2;2	-
Gabon	*Acinetobacter baumannii*	Carbapenems	24;4	38;5 (13.2%; 5.8-27.1)	15;5	1;0
Gabon	*Campylobacter species*	Fluoroquinolones	-	1;1	7;6	1;1
Gabon	Enterobacterales	Carbapenems	582;77 (13.2%; 4.6-32.4)	922;70 (7.6%; 3-17.7)	801;91 (11.4%; 4.8-24.5)	147;34 (23.1%; 6.5-56.4)

Country	Pathogen	Antibiotic/class	2016 N; R(%R;95%CI)	2017 N; R (%R;95%CI)	2018 N; R(%R;95%CI)	2019 N; R(%R;95%CI)
Gabon	Enterobacterales	Cephalosporins (3rd generation)	642;286 (44.5%; 26.8-63.8)	949;349 (36.8%; 23.8-52)	859;391 (45.5%; 31.5-60.2)	254;97 (38.2%; 8.3-80.8)
Gabon	*Enterococcus faecium*	Vancomycin	1;1	-	2;2	-
Gabon	*Haemophilus influenzae*	Ampicillin	-	1;0	2;0	2;0
Gabon	*Helicobacter pylori*	Clarithromycin	-	-	-	-
Gabon	*Neisseria gonorrhoeae*	Cephalosporins (3rd generation)	2;0	4;0	5;2	4;0
Gabon	*Neisseria gonorrhoeae*	Fluoroquinolones	2;0	3;1	5;2	3;1
Gabon	*Pseudomonas aeruginosa*	Carbapenems	19;8	27;8	32;12 (37.5%; 9.7-77)	10;4
Gabon	*Salmonella species*	Fluoroquinolones	9;3	18;7	19;8	15;8
Gabon	*Shigella species*	Fluoroquinolones	3;0	4;1	4;1	23;0
Gabon	*Staphylococcus aureus*	Methicillin	142;109 (76.8%; 44.4-93.2)	130;87 (66.9%; 52.8-78.5)	157;108 (68.8%; 39.2-88.3)	86;36 (41.9%; 0.5-99.1)
Gabon	*Streptococcus pneumoniae*	Beta-lactam combinations	-	-	-	-
Gabon	*Streptococcus pneumoniae*	Penicillins	-	-	3;2	-
Ghana	*Acinetobacter baumannii*	Carbapenems	-	-	10;8	11;7
Ghana	*Campylobacter species*	Fluoroquinolones	-	-	-	-
Ghana	Enterobacterales	Carbapenems	82;78 (95.1%; 76.7-99.1)	156;143 (91.7%; 79.6-96.9)	254;126 (49.6%; 25.8-73.6)	147;77 (52.4%; 42.2-62.3)
Ghana	Enterobacterales	Cephalosporins (3rd generation)	410;294 (71.7%; 60.6-80.7)	493;374 (75.9%; 64.1-84.7)	529;389 (73.5%; 67.7-78.7)	339;227 (67%; 59.5-73.6)
Ghana	*Enterococcus faecium*	Vancomycin	-	-	-	1;0
Ghana	*Haemophilus influenzae*	Ampicillin	-	-	-	-
Ghana	*Helicobacter pylori*	Clarithromycin	-	-	-	-
Ghana	*Neisseria gonorrhoeae*	Cephalosporins (3rd generation)	2;1	2;2	-	1;1
Ghana	*Neisseria gonorrhoeae*	Fluoroquinolones	3;1	3;2	-	1;0
Ghana	*Pseudomonas aeruginosa*	Carbapenems	14;10	3;2	23;20	14;13
Ghana	*Salmonella species*	Fluoroquinolones	6;1	5;1	9;1	8;6
Ghana	*Shigella species*	Fluoroquinolones	1;0	2;0	3;2	2;1
Ghana	*Staphylococcus aureus*	Methicillin	22;17	141;120 (85.1%; 77.8-90.3)	83;59 (71.1%; 48.5-86.5)	102;77 (75.5%; 47.7-91.2)

Country	Pathogen	Antibiotic/class	2016 N; R(%R;95%CI)	2017 N; R (%R;95%CI)	2018 N; R(%R;95%CI)	2019 N; R(%R;95%CI)
Ghana	*Streptococcus pneumoniae*	Beta-lactam combinations	-	2;1	2;1	4;0
Ghana	*Streptococcus pneumoniae*	Penicillins	5;4	11;8	14;14	19;8
Kenya	*Acinetobacter baumannii*	Carbapenems	8;7	9;9	11;11	-
Kenya	*Campylobacter species*	Fluoroquinolones	-	-	-	-
Kenya	Enterobacterales	Carbapenems	609;117 (19.2%; 3-64.7)	655;128 (19.5%; 3.9-59.3)	612;164 (26.8%; 9.6-55.7)	-
Kenya	Enterobacterales	Cephalosporins (3rd generation)	1307;866 (66.3%; 36.8-86.9)	1267;783 (61.8%; 38.5-80.7)	1332;891 (66.9%; 51.5-79.3)	-
Kenya	*Enterococcus faecium*	Vancomycin	-	3;0	3;0	-
Kenya	*Haemophilus influenzae*	Ampicillin	-	-	-	-
Kenya	*Helicobacter pylori*	Clarithromycin	-	-	-	-
Kenya	*Neisseria gonorrhoeae*	Cephalosporins (3rd generation)	5;0	4;0	6;0	-
Kenya	*Neisseria gonorrhoeae*	Fluoroquinolones	8;1	4;1	9;1	-
Kenya	*Pseudomonas aeruginosa*	Carbapenems	37;19 (51.4%; 5.8-94.8)	59;22 (37.3%; 15.2-66.4)	71;26 (36.6%; 19-58.7)	-
Kenya	*Salmonella species*	Fluoroquinolones	18;1	14;0	10;1	-
Kenya	*Shigella species*	Fluoroquinolones	11;1	6;2	10;1	-
Kenya	*Staphylococcus aureus*	Methicillin	75;32 (42.7%; 17.2-72.7)	75;37 (49.3%; 27.5-71.4)	58;23 (39.7%; 25.2-56.2)	-
Kenya	*Streptococcus pneumoniae*	Beta-lactam combinations	-	1;0	1;1	-
Kenya	*Streptococcus pneumoniae*	Penicillins	10;4	4;1	7;4	-
Malawi	*Acinetobacter baumannii*	Carbapenems	-	1;0	57;5 (8.8%; 2.4-27.2)	-
Malawi	*Campylobacter species*	Fluoroquinolones	-	-	-	-
Malawi	Enterobacterales	Carbapenems	81;0 (0%; 0-0)	325;176 (54.2%; 15.4-88.5)	299;3 (1%; 0.2-5)	-
Malawi	Enterobacterales	Cephalosporins (3rd generation)	1063;260 (24.5%; 18.2-32)	1549;627 (40.5%; 19.8-65.2)	1521;651 (42.8%; 27.9-59.2)	7;4
Malawi	*Enterococcus faecium*	Vancomycin	-	-	-	-
Malawi	*Haemophilus influenzae*	Ampicillin	11;6	5;3	6;5	-
Malawi	*Helicobacter pylori*	Clarithromycin	-	-	-	-
Malawi	*Neisseria gonorrhoeae*	Cephalosporins (3rd generation)	4;1	62;0 (0%; 0-0)	68;0 (0%; 0-0)	-

Country	Pathogen	Antibiotic/class	2016<br>N; R(%R;95%CI)	2017<br>N; R (%R;95%CI)	2018<br>N; R(%R;95%CI)	2019<br>N; R(%R;95%CI)
Malawi	*Neisseria gonorrhoeae*	Fluoroquinolones	5;2	40;23 (57.5%; 25.4-84.3)	66;30 (45.5%; 34-57.4)	-
Malawi	*Pseudomonas aeruginosa*	Carbapenems	11;5	8;5	43;7 (16.3%; 7.5-31.8)	-
Malawi	*Salmonella species*	Fluoroquinolones	468;4 (0.9%; 0.1-6.3)	426;6 (1.4%; 0.1-14.4)	387;7 (1.8%; 0.2-14.7)	1;0
Malawi	*Shigella species*	Fluoroquinolones	6;0	8;1	6;1	-
Malawi	*Staphylococcus aureus*	Methicillin	129;23 (17.8%; 13-24)	132;24 (18.2%; 13.1-24.7)	194;66 (34%; 12.4-65.3)	-
Malawi	*Streptococcus pneumoniae*	Beta-lactam combinations	-	2;1	2;0	-
Malawi	*Streptococcus pneumoniae*	Penicillins	2;1	4;2	5;2	-
Nigeria	*Acinetobacter baumannii*	Carbapenems	6;1	19;11	24;8	-
Nigeria	*Campylobacter species*	Fluoroquinolones	-	-	-	-
Nigeria	Enterobacterales	Carbapenems	458;76 (16.6%; 7-34.6)	1342;218 (16.2%; 10.7-24)	927;99 (10.7%; 7-16)	-
Nigeria	Enterobacterales	Cephalosporins (3rd generation)	2341;1395 (59.6%; 47.8-70.3)	3028;1719 (56.8%; 47.7-65.4)	2528;1261 (49.9%; 34-65.8)	-
Nigeria	*Enterococcus faecium*	Vancomycin	-	-	-	-
Nigeria	*Haemophilus influenzae*	Ampicillin	-	-	1;1	-
Nigeria	*Helicobacter pylori*	Clarithromycin	-	-	-	-
Nigeria	*Neisseria gonorrhoeae*	Cephalosporins (3rd generation)	3;1	2;1	1;0	-
Nigeria	*Neisseria gonorrhoeae*	Fluoroquinolones	3;1	2;0	1;0	-
Nigeria	*Pseudomonas aeruginosa*	Carbapenems	55;28 (50.9%; 31.9-69.7)	361;104 (28.8%; 19.6-40.2)	129;42 (32.6%; 20.5-47.5)	-
Nigeria	*Salmonella species*	Fluoroquinolones	100;35 (35%; 24.5-47.2)	130;20 (15.4%; 5.8-35.1)	100;29 (29%; 20-40)	-
Nigeria	*Shigella species*	Fluoroquinolones	14;5	5;1	5;1	-
Nigeria	*Staphylococcus aureus*	Methicillin	458;304 (66.4%; 51.6-78.5)	1152;605 (52.5%; 34.3-70.1)	646;470 (72.8%; 59.9-82.7)	-
Nigeria	*Streptococcus pneumoniae*	Beta-lactam combinations	18;10	59;31 (52.5%; 44-60.9)	7;4	-
Nigeria	*Streptococcus pneumoniae*	Penicillins	3;2	12;10	1;1	-
Senegal	*Acinetobacter baumannii*	Carbapenems	-	6;0	11;1	32;6 (18.8%; 12.4-27.4)
Senegal	*Campylobacter species*	Fluoroquinolones	-	-	-	-
Senegal	Enterobacterales	Carbapenems	168;2 (1.2%; 0.1-14.1)	2182;29 (1.3%; 0.7-2.4)	2015;62 (3.1%; 1.4-6.5)	2329;96 (4.1%; 2.5-6.7)

Country	Pathogen	Antibiotic/class	2016<br>N; R(%R;95%CI)	2017<br>N; R (%R;95%CI)	2018<br>N; R(%R;95%CI)	2019<br>N; R(%R;95%CI)
Senegal	Enterobacterales	Cephalosporins (3rd generation)	213;80 (37.6%; 15.1-67.1)	2941;1125 (38.3%; 30.6-46.5)	2807;1131 (40.3%; 33.5-47.5)	3127;1265 (40.5%; 34.1-47.2)
Senegal	*Enterococcus faecium*	Vancomycin	-	-	-	-
Senegal	*Haemophilus influenzae*	Ampicillin	-	-	-	-
Senegal	*Helicobacter pylori*	Clarithromycin	-	-	-	-
Senegal	*Neisseria gonorrhoeae*	Cephalosporins (3rd generation)	-	5;0	4;2	5;1
Senegal	*Neisseria gonorrhoeae*	Fluoroquinolones	-	2;0	3;3	4;1
Senegal	*Pseudomonas aeruginosa*	Carbapenems	7;0	157;5 (3.2%; 1-10)	114;5 (4.4%; 2.1-8.9)	193;10 (5.2%; 2.4-10.9)
Senegal	*Salmonella species*	Fluoroquinolones	-	17;1	10;1	12;3
Senegal	*Shigella species*	Fluoroquinolones	-	4;1	4;1	5;1
Senegal	*Staphylococcus aureus*	Methicillin	31;13 (41.9%; 6.2-88.8)	442;142 (32.1%; 21.7-44.7)	346;87 (25.1%; 15.5-38)	506;202 (39.9%; 25.3-56.6)
Senegal	*Streptococcus pneumoniae*	Beta-lactam combinations	-	5;1	-	-
Senegal	*Streptococcus pneumoniae*	Penicillins	2;2	8;4	-	2;1
Sierra Leone	*Acinetobacter baumannii*	Carbapenems	-	-	-	-
Sierra Leone	*Campylobacter species*	Fluoroquinolones	-	-	-	-
Sierra Leone	Enterobacterales	Carbapenems	-	-	7;1	-
Sierra Leone	Enterobacterales	Cephalosporins (3rd generation)	-	3;2	22;15	-
Sierra Leone	*Enterococcus faecium*	Vancomycin	-	-	-	-
Sierra Leone	*Haemophilus influenzae*	Ampicillin	-	-	-	-
Sierra Leone	*Helicobacter pylori*	Clarithromycin	-	-	-	-
Sierra Leone	*Neisseria gonorrhoeae*	Cephalosporins (3rd generation)	-	1;0	1;0	-
Sierra Leone	*Neisseria gonorrhoeae*	Fluoroquinolones	5;0	17;9	1;0	-
Sierra Leone	*Pseudomonas aeruginosa*	Carbapenems	-	-	1;0	-
Sierra Leone	*Salmonella species*	Fluoroquinolones	-	-	1;0	-
Sierra Leone	*Shigella species*	Fluoroquinolones	-	-	2;2	-
Sierra Leone	*Staphylococcus aureus*	Methicillin	26;20	5;2	8;7	-
Sierra Leone	*Streptococcus pneumoniae*	Beta-lactam combinations	-	1;1	-	-

Country	Pathogen	Antibiotic/class	2016<br>N; R(%R;95%CI)	2017<br>N; R (%R;95%CI)	2018<br>N; R(%R;95%CI)	2019<br>N; R(%R;95%CI)
Sierra Leone	*Streptococcus pneumoniae*	Penicillins	1;1	5;3	1;1	-
Tanzania	*Acinetobacter baumannii*	Carbapenems	-	1;0	2;2	-
Tanzania	*Campylobacter species*	Fluoroquinolones	1;0	1;1	-	-
Tanzania	Enterobacterales	Carbapenems	74;18 (24.3%; 2.1-82.9)	614;106 (17.3%; 6-40.5)	1052;155 (14.7%; 4.3-40.2)	-
Tanzania	Enterobacterales	Cephalosporins (3rd generation)	614;320 (52.1%; 39.8-64.2)	1374;754 (54.9%; 46.4-63.1)	1530;897 (58.6%; 53.4-63.7)	-
Tanzania	*Enterococcus faecium*	Vancomycin	-	-	1;0	-
Tanzania	*Haemophilus influenzae*	Ampicillin	-	-	23;1	-
Tanzania	*Helicobacter pylori*	Clarithromycin	-	-	-	-
Tanzania	*Neisseria gonorrhoeae*	Cephalosporins (3rd generation)	1;0	8;3	11;2	-
Tanzania	*Neisseria gonorrhoeae*	Fluoroquinolones	2;1	10;4	9;7	-
Tanzania	*Pseudomonas aeruginosa*	Carbapenems	4;2	83;19 (22.9%; 11.4-40.6)	139;41 (29.5%; 11.9-56.4)	-
Tanzania	*Salmonella species*	Fluoroquinolones	13;2	26;8	25;1	-
Tanzania	*Shigella species*	Fluoroquinolones	37;3 (8.1%; 4.5-14.1)	33;7 (21.2%; 7.5-47.2)	44;5 (11.4%; 4.9-24.2)	-
Tanzania	*Staphylococcus aureus*	Methicillin	85;21 (24.7%; 7.3-57.9)	189;62 (32.8%; 11.6-64.4)	303;98 (32.3%; 13.7-59.1)	-
Tanzania	*Streptococcus pneumoniae*	Beta-lactam combinations	1;0	6;2	1;1	-
Tanzania	*Streptococcus pneumoniae*	Penicillins	2;2	14;9	25;10	-
Uganda	*Acinetobacter baumannii*	Carbapenems	50;27 (54%; 39.3-68)	66;20 (30.3%; 22.7-39.1)	60;26 (43.3%; 23.5-65.6)	-
Uganda	*Campylobacter species*	Fluoroquinolones	-	-	1;1	-
Uganda	Enterobacterales	Carbapenems	1953;64 (3.3%; 1-10.1)	2545;64 (2.5%; 1-6.2)	2698;66 (2.4%; 0.9-6.3)	-
Uganda	Enterobacterales	Cephalosporins (3rd generation)	2395;1136 (47.4%; 34.4-60.8)	2903;1534 (52.8%; 47.8-57.8)	3218;1587 (49.3%; 42.1-56.5)	-
Uganda	*Enterococcus faecium*	Vancomycin	15;0	3;0	8;0	-
Uganda	*Haemophilus influenzae*	Ampicillin	1;0	1;1	1;1	-
Uganda	*Helicobacter pylori*	Clarithromycin	-	-	-	-
Uganda	*Neisseria gonorrhoeae*	Cephalosporins (3rd generation)	21;2	17;1	36;3 (8.3%; 1.4-36.6)	-
Uganda	*Neisseria gonorrhoeae*	Fluoroquinolones	20;11	23;9	35;10 (28.6%; 18.4-41.5)	-
Uganda	*Pseudomonas aeruginosa*	Carbapenems	126;12 (9.5%; 4.6-18.6)	179;37 (20.7%; 9.1-40.5)	150;26 (17.3%; 9.8-28.9)	-

Country	Pathogen	Antibiotic/class	2016<br>N; R(%R;95%CI)	2017<br>N; R (%R;95%CI)	2018<br>N; R(%R;95%CI)	2019<br>N; R(%R;95%CI)
Uganda	*Salmonella species*	Fluoroquinolones	41;0 (0%; 0-0)	63;7 (11.1%; 3.4-30.9)	78;12 (15.4%; 6.5-32.4)	-
Uganda	*Shigella species*	Fluoroquinolones	3;0	15;3	27;5	-
Uganda	*Staphylococcus aureus*	Methicillin	727;236 (32.5%; 11.6-63.8)	622;217 (34.9%; 8.4-75.9)	676;232 (34.3%; 15.3-60.2)	-
Uganda	*Streptococcus pneumoniae*	Beta-lactam combinations	13;1	11;1	15;0	-
Uganda	*Streptococcus pneumoniae*	Penicillins	67;18 (26.9%; 7.7-61.7)	46;17 (37%; 14.5-67)	61;25 (41%; 30.6-52.3)	-
Zambia	*Acinetobacter baumannii*	Carbapenems	51;9 (17.6%; 9.4-30.6)	51;1 (2%; -0.5-11.5)	12;0	1;0
Zambia	*Campylobacter species*	Fluoroquinolones	-	-	-	-
Zambia	Enterobacterales	Carbapenems	810;10 (1.2%; 0.6-2.3)	679;23 (3.4%; 1-11)	397;10 (2.5%; 0.8-8)	311;7 (2.3%; 1-4.7)
Zambia	Enterobacterales	Cephalosporins (3rd generation)	1583;918 (58%; 57-59)	1763;1060 (60.1%; 55.5-64.6)	1722;1009 (58.6%; 47.2-69.2)	1315;947 (72%; 62-80.2)
Zambia	*Enterococcus faecium*	Vancomycin	45;1 (2.2%; 0-12.8)	28;0	3;0	-
Zambia	*Haemophilus influenzae*	Ampicillin	15;3	2;0	8;2	1;0
Zambia	*Helicobacter pylori*	Clarithromycin	-	-	-	-
Zambia	*Neisseria gonorrhoeae*	Cephalosporins (3rd generation)	1;1	-	2;1	-
Zambia	*Neisseria gonorrhoeae*	Fluoroquinolones	-	1;1	1;0	1;1
Zambia	*Pseudomonas aeruginosa*	Carbapenems	70;3 (4.3%; 1-12.5)	67;8 (11.9%; 6-22.2)	62;3 (4.8%; 0.1-78.9)	53;1 (1.9%; -0.5-11.1)
Zambia	*Salmonella species*	Fluoroquinolones	70;46 (65.7%; 52.6-76.8)	68;42 (61.8%; 42.9-77.7)	80;32 (40%; 17.6-67.5)	59;7 (11.9%; 5.6-22.9)
Zambia	*Shigella species*	Fluoroquinolones	36;5 (13.9%; 5.7-29.3)	18;3	14;6	2;1
Zambia	*Staphylococcus aureus*	Methicillin	495;146 (29.5%; 25.6-33.7)	417;173 (41.5%; 33.5-50)	376;233 (62%; 56-67.6)	696;392 (56.3%; 53.1-59.5)
Zambia	*Streptococcus pneumoniae*	Beta-lactam combinations	-	7;0	-	-
Zambia	*Streptococcus pneumoniae*	Penicillins	23;3	10;0	19;11	8;2
Zimbabwe	*Acinetobacter baumannii*	Carbapenems	4;1	5;1	10;4	-
Zimbabwe	*Campylobacter species*	Fluoroquinolones	-	-	-	-
Zimbabwe	Enterobacterales	Carbapenems	122;1 (0.8%; 0.1-4.4)	238;13 (5.5%; 2.9-10)	224;18 (8%; 3.2-18.9)	-
Zimbabwe	Enterobacterales	Cephalosporins (3rd generation)	269;106 (39.4%; 18.4-65.2)	539;268 (49.7%; 30-69.5)	363;184 (50.7%; 41.3-60.1)	-
Zimbabwe	*Enterococcus faecium*	Vancomycin	1;0	2;0	3;0	-

Country	Pathogen	Antibiotic/class	2016<br>N; R(%R;95%CI)	2017<br>N; R (%R;95%CI)	2018<br>N; R(%R;95%CI)	2019<br>N; R(%R;95%CI)
Zimbabwe	*Haemophilus influenzae*	Ampicillin	-	-	-	-
Zimbabwe	*Helicobacter pylori*	Clarithromycin	-	-	-	-
Zimbabwe	*Neisseria gonorrhoeae*	Cephalosporins (3rd generation)	-	-	-	-
Zimbabwe	*Neisseria gonorrhoeae*	Fluoroquinolones	-	-	-	-
Zimbabwe	*Pseudomonas aeruginosa*	Carbapenems	10;0	25;14	34;10 (29.4%; 13.4-52.9)	-
Zimbabwe	*Salmonella species*	Fluoroquinolones	64;11 (17.2%; 1.9-68.6)	97;20 (20.6%; 6.6-49)	11;3	-
Zimbabwe	*Shigella species*	Fluoroquinolones	10;0	25;5	12;3	-
Zimbabwe	*Staphylococcus aureus*	Methicillin	81;26 (32.1%; 22.3-43.8)	103;45 (43.7%; 26-63.2)	291;132 (45.4%; 38.2-52.7)	-
Zimbabwe	*Streptococcus pneumoniae*	Beta-lactam combinations	-	-	-	-
Zimbabwe	*Streptococcus pneumoniae*	Penicillins	2;0	1;1	2;1	-

N = number of tested isolates; R = resistant isolates; %R and 95%CI are shown only if ≥30 isolates/ year; — information not available
