# Supplementary material for: Antimicrobial resistance in Africa: A retrospective analysis of data from 14 countries, 2016–2019
Source: PLoS Med. 2025 Jun 24;22(6):e1004638. doi: 10.1371/journal.pmed.1004638 (PMC12186946; doi:10.1371/journal.pmed.1004638)
Supplement: S9 Table — (PDF) [file pmed.1004638.s011.pdf]

S9 Table: AMR prevalence estimates for clinically important pathogens, by regions

Africa GBD[#] Region	Pathogen	Antimicrobial agent/class	2016 N; R(%R; 95%CI)	2017 N; R(%R; 95%CI)	2018 N; R(%R; 95%CI)	2019 N; R(%R; 95%CI)
Central	*Acinetobacter baumannii*	Aminoglycosides	27;8	43;13 (30.2%; 17-47.7)	20;7	2;1
Central	*Acinetobacter baumannii*	Beta-lactam combinations (Antipseudomonal)	21;5	38;6 (15.8%; 4.8-40.9)	15;3	-
Central	*Acinetobacter baumannii*	Carbapenems	24;4	38;5 (13.2%; 5.8-27.1)	15;5	1;0
Central	*Acinetobacter baumannii*	Cephalosporins (3rd generation)	27;19	37;28 (75.7%; 32.9-95.2)	17;11	2;2
Central	*Acinetobacter baumannii*	Cephalosporins (4th generation)	21;11	29;8	12;5	-
Central	*Acinetobacter baumannii*	Fluoroquinolones	27;14	41;23 (56.1%; 27-81.5)	18;7	2;1
Central	*Citrobacter*	Aminoglycosides	79;29 (36.7%; 22.8-53.2)	74;25 (33.8%; 21.7-48.5)	52;18 (34.6%; 20.2-52.5)	16;6
Central	*Citrobacter*	Beta-lactam combinations (Antipseudomonal)	54;8 (14.8%; 5.8-32.9)	59;9 (15.3%; 7.7-27.9)	39;8 (20.5%; 9.3-39.4)	14;0
Central	*Citrobacter*	Carbapenems	62;7 (11.3%; 2.9-35.2)	66;8 (12.1%; 5.3-25.5)	45;6 (13.3%; 3.8-37.6)	13;4
Central	*Citrobacter*	Cephalosporins (3rd generation)	63;37 (58.7%; 44.7-71.5)	67;29 (43.3%; 23.7-65.3)	47;27 (57.4%; 34.1-77.9)	16;10
Central	*Citrobacter*	Cephalosporins (4th generation)	54;14 (25.9%; 14-42.9)	58;11 (19%; 6.1-45.8)	42;9 (21.4%; 5.9-54.2)	12;1
Central	*Citrobacter*	Fluoroquinolones	78;36 (46.2%; 24.4-69.4)	72;31 (43.1%; 25.5-62.5)	52;24 (46.2%; 25.2-68.6)	16;8
Central	*Enterobacter*	Aminoglycosides	45;21 (46.7%; 18.1-77.6)	62;18 (29%; 18.3-42.7)	44;9 (20.5%; 9.2-39.5)	18;3
Central	*Enterobacter*	Beta-lactam combinations (Antipseudomonal)	32;5 (15.6%; 4.5-42.1)	41;3 (7.3%; 2.3-20.9)	29;5	11;2
Central	*Enterobacter*	Carbapenems	35;9 (25.7%; 9.1-54.6)	51;6 (11.8%; 4.9-25.5)	38;4 (10.5%; 4.5-22.9)	10;2
Central	*Enterobacter*	Cephalosporins (4th generation)	32;11 (34.4%; 10.6-69.8)	36;4 (11.1%; 4.1-26.8)	24;4	9;3
Central	*Enterobacter*	Fluoroquinolones	44;21 (47.7%; 22.4-74.3)	63;23 (36.5%; 22.7-52.9)	43;18 (41.9%; 20.5-66.8)	19;6
Central	*Enterococcus*	Aminopenicillins	17;7	24;14	26;11	10;5
Central	*Enterococcus faecalis*	Fluoroquinolones	21;10	38;21 (55.3%; 26.6-80.8)	38;18 (47.4%; 10.9-86.9)	10;9
Central	*Enterococcus faecalis*	Vancomycin	17;4	18;2	19;10	1;0
Central	*Enterococcus faecium*	Fluoroquinolones	1;0	-	2;0	-
Central	*Enterococcus faecium*	Vancomycin	1;1	-	2;2	-
Central	*Escherichia coli*	Aminoglycosides	341;122 (35.8%; 22.5-51.7)	485;169 (34.8%; 25.1-46)	396;122 (30.8%; 22.2-41)	119;53 (44.5%; 12.8-81.4)
Central	*Escherichia coli*	Aminopenicillins	315;242 (76.8%; 62.9-86.6)	444;344 (77.5%; 71.9-82.2)	387;303 (78.3%; 71.9-83.5)	118;85 (72%; 49.6-87.1)

Africa GBD[#] Region	Pathogen	Antimicrobial agent/class	2016<br>N; R(%R; 95%CI)	2017<br>N; R(%R; 95%CI)	2018<br>N; R(%R; 95%CI)	2019<br>N; R(%R; 95%CI)
Central	*Escherichia coli*	Carbapenems	279;34 (12.2%; 3.6-34.1)	433;24 (5.5%; 1.7-16.6)	357;25 (7%; 1.9-22.6)	72;15 (20.8%; 4.6-58.9)
Central	*Escherichia coli*	Cephalosporins (3rd generation)	323;129 (39.9%; 20.9-62.5)	450;131 (29.1%; 18.9-41.9)	376;125 (33.2%; 20-49.8)	120;42 (35%; 6.9-79.6)
Central	*Escherichia coli*	Fluoroquinolones	328;151 (46%; 28-65.1)	479;213 (44.5%; 32.3-57.3)	388;189 (48.7%; 40.9-56.6)	121;54 (44.6%; 12.4-82)
Central	*Escherichia coli*	Trimethoprim/Sulfamethoxazole	10;9	57;42 (73.7%; 3.5-99.5)	64;41 (64.1%; 59-68.9)	-
Central	*Haemophilus influenzae*	Aminopenicillins	-	1;0	2;0	2;0
Central	*Haemophilus influenzae*	Cephalosporins (3rd generation)	-	1;0	2;0	2;0
Central	*Klebsiella pneumoniae*	Aminoglycosides	83;36 (43.4%; 24.4-64.6)	166;60 (36.1%; 26.7-46.8)	142;52 (36.6%; 24.1-51.3)	21;5
Central	*Klebsiella pneumoniae*	Carbapenems	64;3 (4.7%; 1.2-16.3)	141;5 (3.5%; 0.7-16.3)	125;10 (8%; 2-27.2)	13;2
Central	*Klebsiella pneumoniae*	Cephalosporins (3rd generation)	74;38 (51.4%; 26.6-75.4)	153;63 (41.2%; 26.9-57.1)	137;74 (54%; 37.5-69.7)	21;7
Central	*Klebsiella pneumoniae*	Fluoroquinolones	81;35 (43.2%; 23.1-65.8)	163;58 (35.6%; 25-47.8)	139;57 (41%; 33.3-49.2)	21;5
Central	*Morganella*	Cephalosporins (3rd generation)	5;2	7;3	7;4	1;1
Central	*Morganella*	Cephalosporins (4th generation)	3;1	7;2	6;2	1;1
Central	*Morganella*	Fluoroquinolones	5;2	7;2	7;3	1;1
Central	*Neisseria gonorrhoeae*	Cephalosporins (3rd generation)	2;0	4;0	5;2	4;0
Central	*Neisseria gonorrhoeae*	Fluoroquinolones	2;0	3;1	5;2	3;1
Central	*Neisseria gonorrhoeae*	Macrolides	2;2	-	3;1	1;1
Central	*Neisseria gonorrhoeae*	Quinolones	2;2	1;1	-	2;2
Central	*Neisseria gonorrhoeae*	Tetracyclines	2;2	2;1	5;4	3;3
Central	*Non-typhoidal Salmonella*	Fluoroquinolones	7;3	18;7	17;8	10;7
Central	*Proteus*	Aminoglycosides	27;6	35;15 (42.9%; 19.8-69.5)	44;13 (29.5%; 14.8-50.3)	12;6
Central	*Proteus*	Aminopenicillins	26;13	33;20 (60.6%; 43.8-75.2)	38;21 (55.3%; 29.9-78.2)	11;4
Central	*Proteus*	Cephalosporins (3rd generation)	19;3	36;10 (27.8%; 6.3-68.6)	32;11 (34.4%; 17.3-56.8)	12;5
Central	*Proteus*	Cephalosporins (4th generation)	13;0	24;3	25;8	9;1
Central	*Proteus*	Fluoroquinolones	27;2	35;11 (31.4%; 9.7-66.2)	48;14 (29.2%; 13.9-51.2)	11;5
Central	*Pseudomonas aeruginosa*	Aminoglycosides	21;15	29;14	38;22 (57.9%; 30.7-81)	18;7
Central	*Pseudomonas aeruginosa*	Beta-lactam combinations (Antipseudomonal)	14;2	23;4	28;5	12;4
Central	*Pseudomonas aeruginosa*	Carbapenems	19;8	27;8	32;12 (37.5%; 9.7-77)	10;4
Central	*Pseudomonas aeruginosa*	Cephalosporins (3rd generation)	18;14	28;22	34;28 (82.4%; 47.1-96.1)	10;10

Africa GBD[#] Region	Pathogen	Antimicrobial agent/class	2016 N; R(%R; 95%CI)	2017 N; R(%R; 95%CI)	2018 N; R(%R; 95%CI)	2019 N; R(%R; 95%CI)
Central	*Pseudomonas aeruginosa*	Cephalosporins (4th generation)	14;7	21;4	32;12 (37.5%; 8.9-78.7)	9;3
Central	*Pseudomonas aeruginosa*	Fluoroquinolones	20;11	30;16 (53.3%; 31.2-74.2)	39;27 (69.2%; 37.2-89.5)	18;9
Central	*Salmonella Typhi*	Fluoroquinolones	2;0	-	2;0	5;1
Central	*Serratia*	Aminoglycosides	51;19 (37.3%; 15.7-65.4)	100;22 (22%; 7.4-49.7)	69;35 (50.7%; 26.8-74.3)	10;6
Central	*Serratia*	Carbapenems	46;10 (21.7%; 9.2-43.2)	86;7 (8.1%; 2.2-25.6)	73;17 (23.3%; 17.4-30.5)	6;2
Central	*Serratia*	Cephalosporins (3rd generation)	53;20 (37.7%; 13.9-69.4)	88;40 (45.5%; 22.1-71)	77;49 (63.6%; 49.6-75.7)	9;7
Central	*Serratia*	Cephalosporins (4th generation)	39;13 (33.3%; 14.2-60.1)	67;11 (16.4%; 6.4-36.1)	67;21 (31.3%; 23.5-40.4)	7;4
Central	*Serratia*	Fluoroquinolones	49;27 (55.1%; 23.5-83.1)	88;37 (42%; 19.5-68.5)	72;37 (51.4%; 42.4-60.3)	10;6
Central	*Shigella*	Fluoroquinolones	3;0	4;1	4;1	23;0
Central	*Staphylococcus aureus*	Beta-lactam combinations (Antipseudomonal)	9;1	17;10	12;1	11;1
Central	*Staphylococcus aureus*	Fluoroquinolones	176;90 (51.1%; 33.8-68.2)	213;91 (42.7%; 30.4-56)	223;106 (47.5%; 37.6-57.7)	73;43 (58.9%; 36.9-77.9)
Central	*Staphylococcus aureus*	Macrolides	194;128 (66%; 45.8-81.6)	204;123 (60.3%; 46.6-72.6)	229;140 (61.1%; 46.3-74.2)	117;57 (48.7%; 9.2-89.9)
Central	*Staphylococcus aureus*	Trimethoprim/Sulfamethoxazole	11;2	26;11	20;11	-
Central	*Staphylococcus aureus*	Methicillin	142;109 (76.8%; 44.4-93.2)	130;87 (66.9%; 52.8-78.5)	157;108 (68.8%; 39.2-88.3)	86;36 (41.9%; 0.5-99.1)
Central	*Streptococcus agalactiae*	Fluoroquinolones	16;1	23;2	42;8 (19%; 9.8-33.7)	-
Central	*Streptococcus agalactiae*	Macrolides	4;0	5;1	20;12	-
Central	*Streptococcus agalactiae*	Penicillins	1;0	5;1	9;2	-
Central	*Streptococcus pneumoniae*	Carbapenems	-	-	2;0	-
Central	*Streptococcus pneumoniae*	Cephalosporins (3rd generation)	-	-	2;0	-
Central	*Streptococcus pneumoniae*	Fluoroquinolones	-	-	2;1	-
Central	*Streptococcus pneumoniae*	Macrolides	1;0	-	3;3	1;0
Central	*Streptococcus pneumoniae*	Penicillins	-	-	3;2	-
Eastern	*Acinetobacter baumannii*	Aminoglycosides	155;56 (36.1%; 15.6-63.4)	181;68 (37.6%; 23.2-54.5)	225;114 (50.7%; 39.8-61.4)	4;3
Eastern	*Acinetobacter baumannii*	Beta-lactam combinations (Antipseudomonal)	39;17 (43.6%; 35.6-52)	73;20 (27.4%; 15.9-42.9)	74;26 (35.1%; 13.3-65.6)	2;0
Eastern	*Acinetobacter baumannii*	Carbapenems	109;43 (39.4%; 10.9-77.6)	128;30 (23.4%; 6.3-58.1)	142;44 (31%; 10.5-63.2)	1;0
Eastern	*Acinetobacter baumannii*	Cephalosporins (3rd generation)	140;75 (53.6%; 23.8-81)	175;102 (58.3%; 25.4-85.2)	199;124 (62.3%; 31.2-85.7)	4;3
Eastern	*Acinetobacter baumannii*	Cephalosporins (4th generation)	56;29 (51.8%; 46.8-56.7)	73;26 (35.6%; 9-75.6)	71;39 (54.9%; 26.9-80.1)	-
Eastern	*Acinetobacter baumannii*	Fluoroquinolones	114;63 (55.3%; 51.9-58.6)	166;83 (50%; 31.3-68.7)	209;111 (53.1%; 47.1-59)	3;2

Africa GBD# Region	Pathogen	Antimicrobial agent/class	2016<br>N; R(%R; 95%CI)	2017<br>N; R(%R; 95%CI)	2018<br>N; R(%R; 95%CI)	2019<br>N; R(%R; 95%CI)
Eastern	*Citrobacter*	Aminoglycosides	149;61 (40.9%; 36.1-46)	248;104 (41.9%; 36.2-47.9)	321;130 (40.5%; 35.2-46)	53;19 (35.8%; 24.3-49.4)
Eastern	*Citrobacter*	Beta-lactam combinations (Antipseudomonal)	30;11 (36.7%; 28.8-45.3)	38;16 (42.1%; 25.8-60.3)	50;11 (22%; 5.5-57.6)	33;8 (24.2%; 12.7-41.3)
Eastern	*Citrobacter*	Carbapenems	56;5 (8.9%; 2.3-28.8)	86;10 (11.6%; 7-18.7)	140;16 (11.4%; 4.6-25.8)	20;0
Eastern	*Citrobacter*	Cephalosporins (3rd generation)	154;82 (53.2%; 33.7-71.8)	227;134 (59%; 40.3-75.4)	307;169 (55%; 52.5-57.6)	52;27 (51.9%; 38.7-64.9)
Eastern	*Citrobacter*	Cephalosporins (4th generation)	29;11	37;14 (37.8%; 24.3-53.5)	43;15 (34.9%; 25-46.2)	37;17 (45.9%; 31.1-61.6)
Eastern	*Citrobacter*	Fluoroquinolones	226;87 (38.5%; 36.3-40.7)	338;137 (40.5%; 35-46.4)	455;189 (41.5%; 29.8-54.3)	152;67 (44.1%; 36.4-52)
Eastern	*Enterobacter*	Aminoglycosides	271;133 (49.1%; 34.5-63.8)	557;260 (46.7%; 30.6-63.5)	704;310 (44%; 36.6-51.8)	114;42 (36.8%; 13.5-68.5)
Eastern	*Enterobacter*	Beta-lactam combinations (Antipseudomonal)	59;27 (45.8%; 14.4-80.9)	133;52 (39.1%; 21.4-60.2)	178;46 (25.8%; 12.4-46.2)	64;11 (17.2%; 9.8-28.5)
Eastern	*Enterobacter*	Carbapenems	135;16 (11.9%; 2.7-39.3)	306;71 (23.2%; 11.8-40.6)	286;47 (16.4%; 8.2-30.2)	20;0
Eastern	*Enterobacter*	Cephalosporins (4th generation)	84;40 (47.6%; 18.6-78.3)	144;74 (51.4%; 44.1-58.6)	87;54 (62.1%; 49.7-73)	48;26 (54.2%; 40.3-67.4)
Eastern	*Enterobacter*	Fluoroquinolones	491;228 (46.4%; 42.4-50.6)	810;390 (48.1%; 43.5-52.8)	1086;562 (51.7%; 45.2-58.2)	237;110 (46.4%; 40.2-52.8)
Eastern	*Enterococcus*	Aminopenicillins	439;136 (31%; 8.7-67.9)	655;111 (16.9%; 3.6-52.5)	555;82 (14.8%; 1.6-65.6)	11;8
Eastern	*Enterococcus faecalis*	Fluoroquinolones	383;148 (38.6%; 19.2-62.6)	640;204 (31.9%; 23-42.3)	606;205 (33.8%; 20.6-50.1)	13;10
Eastern	*Enterococcus faecalis*	Vancomycin	347;17 (4.9%; 0.7-27.3)	503;12 (2.4%; 0.5-11.6)	516;32 (6.2%; 3.1-11.9)	12;0
Eastern	*Enterococcus faecium*	Fluoroquinolones	60;45 (75%; 0.1-100)	41;36 (87.8%; 80.6-92.6)	15;10	-
Eastern	*Enterococcus faecium*	Vancomycin	60;1 (1.7%; 0-91.6)	34;0 (0%; 0-0)	15;0	-
Eastern	*Escherichia coli*	Aminoglycosides	3213;998 (31.1%; 27.8-34.5)	3979;1299 (32.6%; 24.3-42.3)	4366;1369 (31.4%; 24.9-38.6)	327;172 (52.6%; 44-61)
Eastern	*Escherichia coli*	Aminopenicillins	2816;2424 (86.1%; 81.8-89.5)	3637;2984 (82%; 57.8-93.8)	3883;3086 (79.5%; 61.7-90.3)	564;513 (91%; 89.2-92.5)
Eastern	*Escherichia coli*	Carbapenems	1786;52 (2.9%; 0.9-9)	2669;197 (7.4%; 1.4-30.6)	2772;144 (5.2%; 1.2-19.7)	92;5 (5.4%; 2.1-12.5)
Eastern	*Escherichia coli*	Cephalosporins (3rd generation)	3010;1440 (47.8%; 41.8-54)	3961;2139 (54%; 50.5-57.5)	4200;2250 (53.6%; 47.1-60)	344;242 (70.3%; 69-71.7)
Eastern	*Escherichia coli*	Fluoroquinolones	3579;1869 (52.2%; 42.5-61.8)	4140;2310 (55.8%; 49.3-62.1)	4468;2428 (54.3%; 49.2-59.4)	790;425 (53.8%; 50.1-57.5)
Eastern	*Escherichia coli*	Trimethoprim/Sulfamethoxazole	1863;1611 (86.5%; 85-87.8)	2550;2200 (86.3%; 81.8-89.8)	2309;1868 (80.9%; 76.7-84.5)	85;74 (87.1%; 78.1-92.7)
Eastern	*Haemophilus influenzae*	Aminopenicillins	31;12 (38.7%; 14.5-70.1)	10;4	41;16 (39%; 14-71.6)	1;0
Eastern	*Haemophilus influenzae*	Cephalosporins (3rd generation)	21;1	12;1	23;6	1;0
Eastern	*Klebsiella pneumoniae*	Aminoglycosides	1190;661 (55.5%; 34.3-74.9)	1367;778 (56.9%; 39.5-72.8)	1196;670 (56%; 37.4-73.1)	279;173 (62%; 59.8-64.2)
Eastern	*Klebsiella pneumoniae*	Carbapenems	761;77 (10.1%; 1.7-42.5)	792;76 (9.6%; 2.2-33.8)	713;54 (7.6%; 2.6-20.1)	91;0 (0%; 0-0)

Africa GBD[#] Region	Pathogen	Antimicrobial agent/class	2016 N; R(%R; 95%CI)	2017 N; R(%R; 95%CI)	2018 N; R(%R; 95%CI)	2019 N; R(%R; 95%CI)
Eastern	*Klebsiella pneumoniae*	Cephalosporins (3rd generation)	1251;943 (75.4%; 65-83.5)	1378;1016 (73.7%; 65.4-80.6)	1206;910 (75.5%; 65-83.6)	289;244 (84.4%; 81.5-87)
Eastern	*Klebsiella pneumoniae*	Fluoroquinolones	1212;629 (51.9%; 43.6-60.1)	1479;732 (49.5%; 36.3-62.7)	1181;542 (45.9%; 33.4-58.9)	467;260 (55.7%; 51.1-60.1)
Eastern	*Morganella*	Cephalosporins (3rd generation)	42;27 (64.3%; 46.4-78.9)	46;31 (67.4%; 55.5-77.4)	58;24 (41.4%; 33.2-50)	3;1
Eastern	*Morganella*	Cephalosporins (4th generation)	5;3	4;1	3;2	-
Eastern	*Morganella*	Fluoroquinolones	35;18 (51.4%; 35.1-67.5)	35;17 (48.6%; 30.9-66.6)	62;25 (40.3%; 30.3-51.2)	4;2
Eastern	*Neisseria gonorrhoeae*	Cephalosporins (3rd generation)	32;4 (12.5%; 4.9-28.3)	91;4 (4.4%; 0.3-43.5)	123;6 (4.9%; 0.9-22.3)	-
Eastern	*Neisseria gonorrhoeae*	Fluoroquinolones	35;15 (42.9%; 19.4-70)	78;38 (48.7%; 35.2-62.4)	120;48 (40%; 27.1-54.4)	1;1
Eastern	*Neisseria gonorrhoeae*	Macrolides	24;11	15;8	29;20	-
Eastern	*Neisseria gonorrhoeae*	Quinolones	6;5	2;2	6;5	-
Eastern	*Neisseria gonorrhoeae*	Tetracyclines	13;8	70;66 (94.3%; 56.2-99.5)	90;75 (83.3%; 60.3-94.3)	-
Eastern	*Non-typhoidal Salmonella*	Fluoroquinolones	136;37 (27.2%; 5.2-71.8)	140;38 (27.1%; 6.9-65.3)	110;30 (27.3%; 7.6-63)	27;6
Eastern	*Proteus*	Aminoglycosides	596;163 (27.3%; 17-41)	688;243 (35.3%; 25.6-46.5)	834;313 (37.5%; 28.9-47.1)	242;111 (45.9%; 44.2-47.5)
Eastern	*Proteus*	Aminopenicillins	517;389 (75.2%; 68.4-81)	659;446 (67.7%; 53.1-79.5)	699;472 (67.5%; 55.5-77.6)	255;211 (82.7%; 81.5-83.9)
Eastern	*Proteus*	Cephalosporins (3rd generation)	564;211 (37.4%; 29.7-45.9)	825;332 (40.2%; 34.4-46.3)	817;392 (48%; 38.3-57.8)	258;192 (74.4%; 72.8-76)
Eastern	*Proteus*	Cephalosporins (4th generation)	62;31 (50%; 38.3-61.7)	88;25 (28.4%; 12.5-52.5)	52;18 (34.6%; 8.9-74.1)	96;71 (74%; 64.3-81.7)
Eastern	*Proteus*	Fluoroquinolones	651;229 (35.2%; 22-51.1)	805;274 (34%; 26.5-42.5)	839;317 (37.8%; 28.6-47.9)	295;107 (36.3%; 30.3-42.7)
Eastern	*Pseudomonas aeruginosa*	Aminoglycosides	493;142 (28.8%; 20.1-39.4)	711;215 (30.2%; 17.2-47.5)	839;197 (23.5%; 15.4-34)	337;105 (31.2%; 26.5-36.3)
Eastern	*Pseudomonas aeruginosa*	Beta-lactam combinations (Antipseudomonal)	140;22 (15.7%; 8.5-27.2)	237;45 (19%; 11.8-29.1)	373;51 (13.7%; 7.6-23.3)	127;16 (12.6%; 7.8-19.6)
Eastern	*Pseudomonas aeruginosa*	Carbapenems	248;41 (16.5%; 5.3-41.2)	396;91 (23%; 16.6-31)	465;103 (22.2%; 14-33.2)	53;1 (1.9%; -0.5-11.1)
Eastern	*Pseudomonas aeruginosa*	Cephalosporins (3rd generation)	382;198 (51.8%; 38.6-64.8)	631;305 (48.3%; 30.4-66.7)	753;404 (53.7%; 39.7-67)	306;185 (60.5%; 54.9-65.8)
Eastern	*Pseudomonas aeruginosa*	Cephalosporins (4th generation)	134;44 (32.8%; 19.3-50)	211;59 (28%; 10.8-55.6)	148;47 (31.8%; 6.4-76)	74;31 (41.9%; 31.3-53.3)
Eastern	*Pseudomonas aeruginosa*	Fluoroquinolones	444;102 (23%; 19.8-26.4)	594;153 (25.8%; 21.4-30.6)	752;173 (23%; 15.9-32.1)	390;71 (18.2%; 14.7-22.4)
Eastern	*Salmonella Paratyphi*	Fluoroquinolones	1;0	5;1	2;1	2;0
Eastern	*Salmonella Typhi*	Fluoroquinolones	473;16 (3.4%; 0.2-33.4)	452;24 (5.3%; 0.4-42.2)	468;22 (4.7%; 0.8-24.3)	31;1 (3.2%; -0.7-17.8)
Eastern	*Serratia*	Aminoglycosides	76;25 (32.9%; 17.3-53.5)	138;46 (33.3%; 12.4-63.9)	157;45 (28.7%; 19.9-39.4)	4;2
Eastern	*Serratia*	Carbapenems	37;9 (24.3%; 2.5-79.9)	70;11 (15.7%; 2.8-54.5)	62;14 (22.6%; 3.6-69.6)	1;0
Eastern	*Serratia*	Cephalosporins (3rd generation)	80;49 (61.3%; 48.6-72.6)	138;70 (50.7%; 43.2-58.2)	187;85 (45.5%; 35.7-55.6)	5;3
Eastern	*Serratia*	Cephalosporins (4th generation)	24;8	61;18 (29.5%; 18.9-43)	55;18 (32.7%; 13.6-60)	-

Africa GBD[#] Region	Pathogen	Antimicrobial agent/class	2016 N; R(%R; 95%CI)	2017 N; R(%R; 95%CI)	2018 N; R(%R; 95%CI)	2019 N; R(%R; 95%CI)
Eastern	*Serratia*	Fluoroquinolones	66;18 (27.3%; 23.4-31.6)	117;47 (40.2%; 32.5-48.3)	133;43 (32.3%; 25-40.6)	3;1
Eastern	*Shigella*	Fluoroquinolones	93;9 (9.7%; 6-15.2)	80;16 (20%; 16.6-23.9)	101;18 (17.8%; 9.6-30.8)	2;1
Eastern	*Staphylococcus aureus*	Beta-lactam combinations (Antipseudomonal)	68;21 (30.9%; 25.1-37.3)	32;23 (71.9%; 27.7-94.5)	50;11 (22%; 9.3-43.8)	1;0
Eastern	*Staphylococcus aureus*	Fluoroquinolones	1467;403 (27.5%; 26.3-28.7)	1790;597 (33.4%; 28.2-39)	2380;698 (29.3%; 18.9-42.4)	689;243 (35.3%; 32.9-37.7)
Eastern	*Staphylococcus aureus*	Macrolides	2184;1116 (51.1%; 35-67)	2484;1334 (53.7%; 44.9-62.3)	2650;1419 (53.5%; 45.1-61.8)	553;243 (43.9%; 43.1-44.8)
Eastern	*Staphylococcus aureus*	Trimethoprim/Sulfamethoxazole	1199;860 (71.7%; 69-74.3)	1251;909 (72.7%; 65.2-79.1)	1546;1129 (73%; 69.6-76.2)	169;119 (70.4%; 63.1-76.8)
Eastern	*Staphylococcus aureus*	Methicillin	1511;458 (30.3%; 19-44.6)	1435;513 (35.7%; 20.4-54.7)	1607;652 (40.6%; 27.1-55.6)	696;392 (56.3%; 53.1-59.5)
Eastern	*Streptococcus agalactiae*	Fluoroquinolones	37;9 (24.3%; 21-28)	36;7 (19.4%; 11.8-30.3)	25;3	-
Eastern	*Streptococcus agalactiae*	Macrolides	43;10 (23.3%; 6.8-55.9)	41;15 (36.6%; 17.1-61.8)	25;5	-
Eastern	*Streptococcus agalactiae*	Penicillins	30;4 (13.3%; 2.5-48.1)	31;12 (38.7%; 9.2-79.7)	19;2	-
Eastern	*Streptococcus pneumoniae*	Carbapenems	26;0	9;0	45;3 (6.7%; 4.8-9.2)	-
Eastern	*Streptococcus pneumoniae*	Cephalosporins (3rd generation)	123;5 (4.1%; 0.8-17.5)	95;6 (6.3%; 1.7-20.7)	134;18 (13.4%; 4.6-33.2)	6;0
Eastern	*Streptococcus pneumoniae*	Fluoroquinolones	63;13 (20.6%; 10.6-36.3)	54;15 (27.8%; 21.6-35)	94;8 (8.5%; 3.3-20.2)	-
Eastern	*Streptococcus pneumoniae*	Macrolides	151;61 (40.4%; 12.2-76.8)	107;39 (36.4%; 32.1-41)	176;91 (51.7%; 31.2-71.6)	1;0
Eastern	*Streptococcus pneumoniae*	Penicillins	104;28 (26.9%; 19.1-36.5)	78;29 (37.2%; 22.6-54.5)	117;52 (44.4%; 37.5-51.6)	8;2
Eastern	*Streptococcus pneumoniae*	Trimethoprim/Sulfamethoxazole	63;56 (88.9%; 77.2-95)	44;35 (79.5%; 67.9-87.7)	71;61 (85.9%; 70.8-93.9)	-
Eastern	*Streptococcus pyogenes*	Macrolides	92;27 (29.3%; 17.7-44.5)	195;61 (31.3%; 25.7-37.5)	104;45 (43.3%; 28.8-59)	-
Southern	*Acinetobacter baumannii*	Aminoglycosides	10;5	15;5	31;16 (51.6%; 48.7-54.5)	-
Southern	*Acinetobacter baumannii*	Beta-lactam combinations (Antipseudomonal)	1;0	1;0	-	-
Southern	*Acinetobacter baumannii*	Carbapenems	7;1	6;1	18;6	-
Southern	*Acinetobacter baumannii*	Cephalosporins (3rd generation)	11;7	25;14	28;21	-
Southern	*Acinetobacter baumannii*	Cephalosporins (4th generation)	2;0	1;0	1;1	-
Southern	*Acinetobacter baumannii*	Fluoroquinolones	11;4	33;11 (33.3%; 12.4-63.9)	30;11 (36.7%; 13-69.2)	-
Southern	*Citrobacter*	Aminoglycosides	4;0	25;2	18;5	-
Southern	*Citrobacter*	Beta-lactam combinations (Antipseudomonal)	-	2;0	-	-
Southern	*Citrobacter*	Carbapenems	2;0	5;0	6;1	-
Southern	*Citrobacter*	Cephalosporins (3rd generation)	4;0	24;6	18;11	-
Southern	*Citrobacter*	Cephalosporins (4th generation)	2;0	2;0	1;1	-

Africa GBD[#] Region	Pathogen	Antimicrobial agent/class	2016<br>N; R(%R; 95%CI)	2017<br>N; R(%R; 95%CI)	2018<br>N; R(%R; 95%CI)	2019<br>N; R(%R; 95%CI)
Southern	*Citrobacter*	Fluoroquinolones	5;0	23;2	13;5	-
Southern	*Enterobacter*	Aminoglycosides	10;2	33;4 (12.1%; 1.5-55.8)	16;7	-
Southern	*Enterobacter*	Beta-lactam combinations (Antipseudomonal)	2;1	4;0	5;4	-
Southern	*Enterobacter*	Carbapenems	6;1	7;1	12;0	-
Southern	*Enterobacter*	Cephalosporins (4th generation)	4;1	4;0	7;6	-
Southern	*Enterobacter*	Fluoroquinolones	8;1	33;9 (27.3%; 24.3-30.4)	16;7	-
Southern	*Enterococcus*	Aminopenicillins	28;9	68;29 (42.6%; 41.4-43.9)	25;9	-
Southern	*Enterococcus faecalis*	Fluoroquinolones	20;2	63;7 (11.1%; 4.7-23.9)	22;9	-
Southern	*Enterococcus faecalis*	Vancomycin	20;0	61;6 (9.8%; 6.4-14.9)	18;1	-
Southern	*Enterococcus faecium*	Fluoroquinolones	9;7	13;8	1;1	-
Southern	*Enterococcus faecium*	Vancomycin	6;1	12;2	3;0	-
Southern	*Escherichia coli*	Aminoglycosides	635;133 (20.9%; 4.3-61.2)	730;181 (24.8%; 4.4-70.2)	594;169 (28.5%; 9.2-60.9)	-
Southern	*Escherichia coli*	Aminopenicillins	589;471 (80%; 72.8-85.6)	676;552 (81.7%; 76.9-85.6)	532;411 (77.3%; 75.3-79.1)	-
Southern	*Escherichia coli*	Carbapenems	378;0 (0%; 0-0)	321;7 (2.2%; 1.9-2.5)	352;14 (4%; 0.4-31.7)	-
Southern	*Escherichia coli*	Cephalosporins (3rd generation)	455;101 (22.2%; 8.6-46.3)	640;251 (39.2%; 26.1-54.1)	459;113 (24.6%; 10.8-46.8)	-
Southern	*Escherichia coli*	Fluoroquinolones	508;153 (30.1%; 26.4-34.1)	669;234 (35%; 33.1-36.9)	480;153 (31.9%; 21.2-44.9)	-
Southern	*Escherichia coli*	Trimethoprim/Sulfamethoxazole	435;323 (74.3%; 54.6-87.4)	375;282 (75.2%; 53.6-88.8)	420;308 (73.3%; 56-85.6)	-
Southern	*Haemophilus influenzae*	Aminopenicillins	-	1;1	1;0	-
Southern	*Haemophilus influenzae*	Cephalosporins (3rd generation)	-	-	1;0	-
Southern	*Klebsiella pneumoniae*	Aminoglycosides	59;17 (28.8%; 2-89)	100;17 (17%; 2-66.9)	48;22 (45.8%; 41-50.8)	-
Southern	*Klebsiella pneumoniae*	Carbapenems	46;0 (0%; 0-0)	29;0	29;0	-
Southern	*Klebsiella pneumoniae*	Cephalosporins (3rd generation)	53;37 (69.8%; 43.6-87.4)	100;56 (56%; 55.2-56.8)	49;25 (51%; 38-63.9)	-
Southern	*Klebsiella pneumoniae*	Fluoroquinolones	60;23 (38.3%; 27.5-50.5)	97;33 (34%; 27.8-40.8)	43;14 (32.6%; 14.1-58.7)	-
Southern	*Morganella*	Cephalosporins (3rd generation)	2;0	8;4	5;3	-
Southern	*Morganella*	Cephalosporins (4th generation)	-	1;0	1;0	-
Southern	*Morganella*	Fluoroquinolones	3;1	9;2	5;1	-
Southern	*Neisseria gonorrhoeae*	Cephalosporins (3rd generation)	9;0	14;0	5;1	-
Southern	*Neisseria gonorrhoeae*	Fluoroquinolones	9;1	14;7	5;3	-

Africa GBD# Region	Pathogen	Antimicrobial agent/class	2016 N; R(%R; 95%CI)	2017 N; R(%R; 95%CI)	2018 N; R(%R; 95%CI)	2019 N; R(%R; 95%CI)
Southern	*Neisseria gonorrhoeae*	Macrolides	-	-	1;0	-
Southern	*Neisseria gonorrhoeae*	Quinolones	-	-	1;1	-
Southern	*Neisseria gonorrhoeae*	Tetracyclines	9;2	16;11	4;3	-
Southern	*Non-typhoidal Salmonella*	Fluoroquinolones	14;3	29;5	9;4	-
Southern	*Proteus*	Aminoglycosides	226;60 (26.5%; 5.4-69.5)	268;54 (20.1%; 3.9-61)	130;41 (31.5%; 11.2-62.7)	-
Southern	*Proteus*	Aminopenicillins	204;143 (70.1%; 49-85.1)	226;155 (68.6%; 67.4-69.8)	87;60 (69%; 45.2-85.7)	-
Southern	*Proteus*	Cephalosporins (3rd generation)	114;33 (28.9%; 10.3-59.2)	242;80 (33.1%; 23.3-44.5)	113;39 (34.5%; 22.9-48.3)	-
Southern	*Proteus*	Cephalosporins (4th generation)	26;3	26;3	14;2	-
Southern	*Proteus*	Fluoroquinolones	145;29 (20%; 5.8-50.4)	268;50 (18.7%; 4.5-52.9)	120;34 (28.3%; 15-46.9)	-
Southern	*Pseudomonas aeruginosa*	Aminoglycosides	135;29 (21.5%; 14.2-31.2)	194;70 (36.1%; 11-72.1)	184;63 (34.2%; 17.4-56.3)	1;0
Southern	*Pseudomonas aeruginosa*	Beta-lactam combinations (Antipseudomonal)	9;0	10;1	12;0	-
Southern	*Pseudomonas aeruginosa*	Carbapenems	28;1	36;14 (38.9%; 4.6-89.3)	59;11 (18.6%; 3-62.9)	-
Southern	*Pseudomonas aeruginosa*	Cephalosporins (3rd generation)	104;34 (32.7%; 31.9-33.4)	159;111 (69.8%; 64.6-74.6)	145;73 (50.3%; 39.6-61.1)	-
Southern	*Pseudomonas aeruginosa*	Cephalosporins (4th generation)	16;2	10;1	28;3	-
Southern	*Pseudomonas aeruginosa*	Fluoroquinolones	146;36 (24.7%; 20-29.9)	181;31 (17.1%; 5.9-40.4)	178;41 (23%; 17-30.5)	1;0
Southern	*Salmonella Typhi*	Fluoroquinolones	57;11 (19.3%; 2.2-71.7)	73;16 (21.9%; 7.9-48)	5;0	-
Southern	*Serratia*	Aminoglycosides	9;2	22;4	32;3 (9.4%; 0.6-64.6)	-
Southern	*Serratia*	Carbapenems	4;0	1;0	9;1	-
Southern	*Serratia*	Cephalosporins (3rd generation)	9;4	23;11	32;19 (59.4%; 48.2-69.7)	-
Southern	*Serratia*	Cephalosporins (4th generation)	3;1	-	2;2	-
Southern	*Serratia*	Fluoroquinolones	8;4	22;7	33;9 (27.3%; 12.6-49.4)	-
Southern	*Shigella*	Fluoroquinolones	11;0	28;5	15;4	-
Southern	*Staphylococcus aureus*	Fluoroquinolones	336;91 (27.1%; 21.8-33.1)	416;77 (18.5%; 13-25.6)	456;108 (23.7%; 18.4-30)	-
Southern	*Staphylococcus aureus*	Macrolides	329;120 (36.5%; 35-37.9)	438;139 (31.7%; 24.4-40.1)	475;154 (32.4%; 28.1-37)	1;0
Southern	*Staphylococcus aureus*	Trimethoprim/Sulfamethoxazole	323;182 (56.3%; 35.6-75.1)	338;201 (59.5%; 21.4-88.7)	347;217 (62.5%; 46.9-76)	1;0
Southern	*Staphylococcus aureus*	Methicillin	228;55 (24.1%; 7.4-55.8)	385;141 (36.6%; 15.6-64.4)	465;226 (48.6%; 25.5-72.3)	-
Southern	*Streptococcus agalactiae*	Fluoroquinolones	1;0	3;1	3;2	-
Southern	*Streptococcus agalactiae*	Macrolides	-	2;0	2;0	-

Africa GBD# Region	Pathogen	Antimicrobial agent/class	2016 N; R(%R; 95%CI)	2017 N; R(%R; 95%CI)	2018 N; R(%R; 95%CI)	2019 N; R(%R; 95%CI)
Southern	*Streptococcus agalactiae*	Penicillins	2;0	9;3	4;1	-
Southern	*Streptococcus pneumoniae*	Carbapenems	2;0	-	1;1	-
Southern	*Streptococcus pneumoniae*	Cephalosporins (3rd generation)	5;0	4;0	2;1	-
Southern	*Streptococcus pneumoniae*	Fluoroquinolones	2;0	5;0	3;1	-
Southern	*Streptococcus pneumoniae*	Macrolides	5;0	8;1	3;2	-
Southern	*Streptococcus pneumoniae*	Penicillins	4;0	7;4	4;3	-
Southern	*Streptococcus pneumoniae*	Trimethoprim/Sulfamethoxazole	1;0	4;4	1;1	-
Southern	*Streptococcus pyogenes*	Macrolides	10;3	17;8	14;4	-
Western	*Acinetobacter baumannii*	Aminoglycosides	8;3	79;28 (35.4%; 19.1-56.2)	314;118 (37.6%; 29.1-46.9)	173;93 (53.8%; 49.8-57.7)
Western	*Acinetobacter baumannii*	Beta-lactam combinations (Antipseudomonal)	4;1	20;14	95;46 (48.4%; 23.7-73.9)	68;37 (54.4%; 48.5-60.2)
Western	*Acinetobacter baumannii*	Carbapenems	6;1	47;21 (44.7%; 25.2-66)	202;66 (32.7%; 25-41.4)	148;41 (27.7%; 19.1-38.4)
Western	*Acinetobacter baumannii*	Cephalosporins (3rd generation)	7;5	80;65 (81.2%; 78-84.1)	264;187 (70.8%; 61.5-78.7)	150;101 (67.3%; 53.9-78.4)
Western	*Acinetobacter baumannii*	Cephalosporins (4th generation)	5;2	37;26 (70.3%; 39-89.7)	94;54 (57.4%; 37.4-75.3)	27;18
Western	*Acinetobacter baumannii*	Fluoroquinolones	8;3	72;32 (44.4%; 26.2-64.3)	270;112 (41.5%; 35.6-47.6)	162;74 (45.7%; 36-55.7)
Western	*Citrobacter*	Aminoglycosides	70;32 (45.7%; 23.3-70)	263;97 (36.9%; 20.4-57.1)	374;125 (33.4%; 25.1-42.9)	274;108 (39.4%; 28.7-51.2)
Western	*Citrobacter*	Beta-lactam combinations (Antipseudomonal)	6;1	27;17	76;46 (60.5%; 53.2-67.4)	77;45 (58.4%; 54.4-62.4)
Western	*Citrobacter*	Carbapenems	13;7	105;23 (21.9%; 3.7-67.5)	211;32 (15.2%; 5.8-34.3)	177;17 (9.6%; 4.1-21.1)
Western	*Citrobacter*	Cephalosporins (3rd generation)	65;39 (60%; 45.7-72.7)	250;131 (52.4%; 34.7-69.5)	397;202 (50.9%; 40.4-61.3)	276;143 (51.8%; 33.5-69.7)
Western	*Citrobacter*	Cephalosporins (4th generation)	1;1	44;17 (38.6%; 18.1-64.2)	84;35 (41.7%; 29.6-54.9)	52;22 (42.3%; 37.6-47.1)
Western	*Citrobacter*	Fluoroquinolones	69;34 (49.3%; 31.8-67)	205;85 (41.5%; 27.2-57.4)	322;120 (37.3%; 29.7-45.5)	228;98 (43%; 34.2-52.2)
Western	*Enterobacter*	Aminoglycosides	80;33 (41.2%; 30.6-52.7)	439;187 (42.6%; 33.4-52.4)	581;216 (37.2%; 31.8-42.8)	433;175 (40.4%; 39.1-41.7)
Western	*Enterobacter*	Beta-lactam combinations (Antipseudomonal)	13;6	63;28 (44.4%; 42.2-46.7)	106;52 (49.1%; 28.8-69.6)	88;52 (59.1%; 46.9-70.2)
Western	*Enterobacter*	Carbapenems	24;3	283;53 (18.7%; 2.4-68.2)	374;61 (16.3%; 7-33.6)	320;38 (11.9%; 4.3-28.9)
Western	*Enterobacter*	Cephalosporins (4th generation)	9;5	95;42 (44.2%; 30.9-58.4)	130;78 (60%; 49.1-70)	96;46 (47.9%; 44.1-51.7)
Western	*Enterobacter*	Fluoroquinolones	76;34 (44.7%; 29.7-60.8)	403;167 (41.4%; 34-49.3)	575;235 (40.9%; 33.7-48.4)	413;181 (43.8%; 39.1-48.7)
Western	*Enterococcus*	Aminopenicillins	8;5	34;20 (58.8%; 27.1-84.6)	67;22 (32.8%; 22.8-44.8)	20;7
Western	*Enterococcus faecalis*	Fluoroquinolones	7;4	42;20 (47.6%; 27.9-68.1)	105;38 (36.2%; 21.7-53.7)	8;4
Western	*Enterococcus faecalis*	Vancomycin	2;0	22;6	38;4 (10.5%; 3.7-26.7)	10;2

Africa GBD# Region	Pathogen	Antimicrobial agent/class	2016<br>N; R(%R; 95%CI)	2017<br>N; R(%R; 95%CI)	2018<br>N; R(%R; 95%CI)	2019<br>N; R(%R; 95%CI)
Western	*Enterococcus faecium*	Fluoroquinolones	-	2;1	2;1	2;2
Western	*Enterococcus faecium*	Vancomycin	-	2;0	1;0	1;0
Western	*Escherichia coli*	Aminoglycosides	1764;621 (35.2%; 28.3-42.8)	4447;1509 (33.9%; 29-39.3)	6754;2247 (33.3%; 28.5-38.4)	3135;1068 (34.1%; 31.5-36.8)
Western	*Escherichia coli*	Aminopenicillins	1780;1377 (77.4%; 73.2-81)	4327;3447 (79.7%; 65.6-89)	6898;5628 (81.6%; 76.5-85.8)	3284;2716 (82.7%; 76.3-87.7)
Western	*Escherichia coli*	Carbapenems	280;49 (17.5%; 3.4-56)	2154;138 (6.4%; 1.5-23.6)	3803;243 (6.4%; 4.3-9.4)	2061;138 (6.7%; 3.4-12.9)
Western	*Escherichia coli*	Cephalosporins (3rd generation)	1589;939 (59.1%; 51.4-66.3)	4190;1905 (45.5%; 33.2-58.3)	7091;3417 (48.2%; 41.9-54.6)	3336;1539 (46.1%; 35.9-56.7)
Western	*Escherichia coli*	Fluoroquinolones	1876;753 (40.1%; 29.6-51.7)	3897;1840 (47.2%; 38.2-56.4)	6545;3500 (53.5%; 40.3-66.2)	2944;1530 (52%; 48.8-55.1)
Western	*Escherichia coli*	Trimethoprim/Sulfamethoxazole	568;368 (64.8%; 55.6-73)	1124;761 (67.7%; 48.3-82.5)	2825;2284 (80.8%; 72.7-87)	1035;799 (77.2%; 66.3-85.4)
Western	*Haemophilus influenzae*	Aminopenicillins	1;1	4;3	5;4	1;1
Western	*Haemophilus influenzae*	Cephalosporins (3rd generation)	1;1	7;2	5;4	1;0
Western	*Klebsiella pneumoniae*	Aminoglycosides	262;142 (54.2%; 50.9-57.4)	2052;946 (46.1%; 40.8-51.5)	2346;878 (37.4%; 34.2-40.8)	1489;608 (40.8%; 33.2-48.9)
Western	*Klebsiella pneumoniae*	Carbapenems	155;19 (12.3%; 5.3-25.7)	1203;134 (11.1%; 3.9-28.1)	1614;157 (9.7%; 5.5-16.6)	1036;77 (7.4%; 3.1-16.9)
Western	*Klebsiella pneumoniae*	Cephalosporins (3rd generation)	281;180 (64.1%; 59.5-68.4)	2039;1179 (57.8%; 48.4-66.7)	2552;1553 (60.9%; 52.1-68.9)	1504;846 (56.2%; 44.7-67.1)
Western	*Klebsiella pneumoniae*	Fluoroquinolones	254;118 (46.5%; 32.7-60.8)	1829;841 (46%; 39.7-52.4)	2155;995 (46.2%; 42.1-50.3)	1313;565 (43%; 41-45.1)
Western	*Morganella*	Cephalosporins (3rd generation)	4;2	42;12 (28.6%; 9.9-59.3)	63;24 (38.1%; 22.8-56.2)	57;21 (36.8%; 15.1-65.7)
Western	*Morganella*	Cephalosporins (4th generation)	-	9;0	11;3	10;6
Western	*Morganella*	Fluoroquinolones	4;2	37;17 (45.9%; 38.3-53.8)	50;30 (60%; 40-77.2)	56;36 (64.3%; 32.2-87.2)
Western	*Neisseria gonorrhoeae*	Cephalosporins (3rd generation)	5;2	53;17 (32.1%; 23.2-42.5)	47;20 (42.6%; 38-47.2)	44;12 (27.3%; 22.3-32.9)
Western	*Neisseria gonorrhoeae*	Fluoroquinolones	11;2	64;24 (37.5%; 25.3-51.5)	37;21 (56.8%; 46.2-66.8)	27;9
Western	*Neisseria gonorrhoeae*	Macrolides	3;2	40;18 (45%; 25.6-66)	24;7	10;2
Western	*Neisseria gonorrhoeae*	Quinolones	3;1	13;8	11;8	9;7
Western	*Neisseria gonorrhoeae*	Tetracyclines	6;3	36;16 (44.4%; 26-64.5)	14;8	13;7
Western	*Non-typhoidal Salmonella*	Fluoroquinolones	96;34 (35.4%; 30.5-40.7)	162;22 (13.6%; 7-24.6)	218;51 (23.4%; 15.6-33.5)	92;25 (27.2%; 15.9-42.4)
Western	*Proteus*	Aminoglycosides	229;82 (35.8%; 32.6-39.1)	467;148 (31.7%; 29.9-33.5)	614;138 (22.5%; 16.7-29.5)	335;106 (31.6%; 19.4-47.1)
Western	*Proteus*	Aminopenicillins	229;178 (77.7%; 62.3-88)	440;281 (63.9%; 52.1-74.2)	565;361 (63.9%; 51.9-74.3)	311;216 (69.5%; 64.1-74.4)
Western	*Proteus*	Cephalosporins (3rd generation)	202;111 (55%; 40.3-68.8)	435;173 (39.8%; 24.4-57.4)	642;223 (34.7%; 24.1-47.2)	333;115 (34.5%; 18.8-54.6)

Africa GBD[#] Region	Pathogen	Antimicrobial agent/class	2016<br>N; R(%R; 95%CI)	2017<br>N; R(%R; 95%CI)	2018<br>N; R(%R; 95%CI)	2019<br>N; R(%R; 95%CI)
Western	*Proteus*	Cephalosporins (4th generation)	5;3	54;6 (11.1%; 1-60)	82;34 (41.5%; 21.2-65.2)	43;12 (27.9%; 23.3-33.1)
Western	*Proteus*	Fluoroquinolones	241;86 (35.7%; 28.9-43.1)	435;143 (32.9%; 31.9-33.9)	630;170 (27%; 19.7-35.8)	311;111 (35.7%; 30-41.8)
Western	*Pseudomonas aeruginosa*	Aminoglycosides	201;74 (36.8%; 27.5-47.3)	757;305 (40.3%; 38.4-42.2)	834;338 (40.5%; 30.5-51.4)	507;220 (43.4%; 35.5-51.6)
Western	*Pseudomonas aeruginosa*	Beta-lactam combinations (Antipseudomonal)	40;12 (30%; 2.2-89.2)	239;61 (25.5%; 13.4-43.1)	353;133 (37.7%; 18.4-61.9)	167;76 (45.5%; 18.1-75.9)
Western	*Pseudomonas aeruginosa*	Carbapenems	76;38 (50%; 34.1-65.9)	587;129 (22%; 10.2-41)	572;136 (23.8%; 14.1-37.3)	377;63 (16.7%; 5.2-42.2)
Western	*Pseudomonas aeruginosa*	Cephalosporins (3rd generation)	214;146 (68.2%; 60.3-75.2)	766;434 (56.7%; 49.8-63.3)	765;444 (58%; 49.5-66.1)	445;278 (62.5%; 57-67.6)
Western	*Pseudomonas aeruginosa*	Cephalosporins (4th generation)	33;16 (48.5%; 0.1-99.8)	152;34 (22.4%; 16.4-29.7)	259;107 (41.3%; 33.8-49.2)	90;47 (52.2%; 41.6-62.6)
Western	*Pseudomonas aeruginosa*	Fluoroquinolones	181;77 (42.5%; 23.1-64.6)	738;280 (37.9%; 29.2-47.6)	775;297 (38.3%; 26.4-51.9)	450;150 (33.3%; 20.8-48.7)
Western	*Salmonella Paratyphi*	Fluoroquinolones	1;0	3;1	6;1	4;1
Western	*Salmonella Typhi*	Fluoroquinolones	9;2	28;2	21;1	6;1
Western	*Serratia*	Aminoglycosides	4;2	97;20 (20.6%; 15.1-27.6)	135;46 (34.1%; 28.4-40.3)	100;33 (33%; 15.7-56.6)
Western	*Serratia*	Carbapenems	2;0	39;6 (15.4%; 7.1-30.1)	89;26 (29.2%; 9.4-62)	59;10 (16.9%; 8.3-31.5)
Western	*Serratia*	Cephalosporins (3rd generation)	4;3	94;44 (46.8%; 32.9-61.3)	152;94 (61.8%; 43.2-77.6)	98;52 (53.1%; 41.5-64.3)
Western	*Serratia*	Cephalosporins (4th generation)	1;0	13;5	39;27 (69.2%; 33.4-91)	11;3
Western	*Serratia*	Fluoroquinolones	4;2	65;17 (26.2%; 15.2-41.2)	130;50 (38.5%; 28.7-49.2)	90;32 (35.6%; 15.4-62.5)
Western	*Shigella*	Fluoroquinolones	15;5	40;6 (15%; 11.4-19.5)	134;44 (32.8%; 29.2-36.7)	34;10 (29.4%; 24.7-34.6)
Western	*Staphylococcus aureus*	Beta-lactam combinations (Antipseudomonal)	6;4	65;24 (36.9%; 15.5-65.2)	62;24 (38.7%; 8-82.2)	19;8
Western	*Staphylococcus aureus*	Fluoroquinolones	1402;521 (37.2%; 34.5-39.9)	3095;1184 (38.3%; 33.4-43.3)	3362;1120 (33.3%; 28.7-38.3)	1392;487 (35%; 24.4-47.2)
Western	*Staphylococcus aureus*	Macrolides	1092;515 (47.2%; 41.3-53.1)	3007;1348 (44.8%; 36.7-53.3)	2688;1198 (44.6%; 38.4-50.9)	1159;470 (40.6%; 26.3-56.6)
Western	*Staphylococcus aureus*	Trimethoprim/Sulfamethoxazole	393;288 (73.3%; 64.8-80.4)	596;428 (71.8%; 54.1-84.6)	1011;631 (62.4%; 42-79.2)	479;286 (59.7%; 43.7-73.9)
Western	*Staphylococcus aureus*	Methicillin	537;354 (65.9%; 53.4-76.5)	2065;1037 (50.2%; 39.5-60.9)	2162;1047 (48.4%; 37.8-59.2)	1158;593 (51.2%; 40.9-61.5)
Western	*Streptococcus agalactiae*	Fluoroquinolones	-	7;3	23;5	2;0
Western	*Streptococcus agalactiae*	Macrolides	-	7;3	17;7	2;0
Western	*Streptococcus agalactiae*	Penicillins	-	8;7	23;8	3;0
Western	*Streptococcus pneumoniae*	Carbapenems	4;3	16;5	6;2	9;4
Western	*Streptococcus pneumoniae*	Cephalosporins (3rd generation)	23;14	88;55 (62.5%; 38.2-81.8)	17;7	17;3
Western	*Streptococcus pneumoniae*	Fluoroquinolones	22;5	94;42 (44.7%; 27.2-63.6)	25;9	21;4

Africa GBD[#] Region	Pathogen	Antimicrobial agent/class	2016 N; R(%R; 95%CI)	2017 N; R(%R; 95%CI)	2018 N; R(%R; 95%CI)	2019 N; R(%R; 95%CI)
Western	*Streptococcus pneumoniae*	Macrolides	23;6	87;51 (58.6%; 47.1-69.3)	34;14 (41.2%; 27.6-56.3)	19;6
Western	*Streptococcus pneumoniae*	Penicillins	11;9	45;32 (71.1%; 57.1-82)	25;19	26;10
Western	*Streptococcus pneumoniae*	Trimethoprim/Sulfamethoxazole	11;11	12;11	10;8	4;3
Western	*Streptococcus pyogenes*	Macrolides	1;1	24;9	16;9	5;1

[#]GBD=Global burden of disease; N = number of tested isolates; R = resistant isolates; %R and 95%CI are shown only if ≥30 isolates/ year; — information not available. Regions (**Central** - Gabon; **Eastern** - Malawi, Kenya, Uganda, Tanzania and Zambia; **Southern** - Eswatini and Zimbabwe; **Western** – Burkina Faso, Cameroon, Ghana, Nigeria, Senegal, and Sierra Leone).
