# Supplementary material for: Antimicrobial resistance in Africa: A retrospective analysis of data from 14 countries, 2016–2019
Source: PLoS Med. 2025 Jun 24;22(6):e1004638. doi: 10.1371/journal.pmed.1004638 (PMC12186946; doi:10.1371/journal.pmed.1004638)
Supplement: S10 Table — (PDF) [file pmed.1004638.s012.pdf]

S10 Table: AMR prevalence estimates for clinically important pathogens by regions and patients' departments (Inpatient/Outpatient)

Africa GBD[#] Region	Pathogen	Antimicrobial agent/class	Patient Departmen t	2016 N; R(%R; 95%CI)	2017 N; R(%R; 95%CI)	2018 N; R(%R; 95%CI)	2019 N; R(%R; 95%CI)
Central	*Acinetobacter baumannii*	Aminoglycosides	Outpatient	2;1	6;1	6;3	1;1
Central	*Acinetobacter baumannii*	Beta-lactam combinations (Antipseudomonal)	Outpatient	2;1	5;1	5;0	-
Central	*Acinetobacter baumannii*	Carbapenems	Outpatient	1;1	6;1	6;2	1;0
Central	*Acinetobacter baumannii*	Cephalosporins (3rd generation)	Outpatient	2;2	6;4	5;3	1;1
Central	*Acinetobacter baumannii*	Cephalosporins (4th generation)	Outpatient	1;1	4;1	4;2	-
Central	*Acinetobacter baumannii*	Fluoroquinolones	Outpatient	2;1	6;4	4;2	1;1
Central	*Citrobacter*	Aminoglycosides	Inpatient	-	1;1	1;1	-
Central	*Citrobacter*	Aminoglycosides	Outpatient	22;7	19;6	21;9	9;5
Central	*Citrobacter*	Beta-lactam combinations (Antipseudomonal)	Inpatient	-	1;0	1;1	-
Central	*Citrobacter*	Beta-lactam combinations (Antipseudomonal)	Outpatient	10;3	15;4	13;2	8;0
Central	*Citrobacter*	Carbapenems	Inpatient	-	1;1	1;0	-
Central	*Citrobacter*	Carbapenems	Outpatient	17;5	16;4	18;4	8;3
Central	*Citrobacter*	Cephalosporins (3rd generation)	Inpatient	-	1;1	1;1	-
Central	*Citrobacter*	Cephalosporins (3rd generation)	Outpatient	13;9	17;7	19;13	9;8
Central	*Citrobacter*	Cephalosporins (4th generation)	Inpatient	-	1;1	1;0	-
Central	*Citrobacter*	Cephalosporins (4th generation)	Outpatient	10;4	10;2	13;4	7;1
Central	*Citrobacter*	Fluoroquinolones	Inpatient	-	1;1	1;1	-
Central	*Citrobacter*	Fluoroquinolones	Outpatient	21;6	19;8	22;11	9;7
Central	*Enterobacter*	Aminoglycosides	Inpatient	-	4;1	-	-
Central	*Enterobacter*	Aminoglycosides	Outpatient	25;14	24;7	21;4	8;1
Central	*Enterobacter*	Beta-lactam combinations (Antipseudomonal)	Outpatient	17;4	15;1	11;1	6;1
Central	*Enterobacter*	Carbapenems	Inpatient	-	3;0	-	-
Central	*Enterobacter*	Carbapenems	Outpatient	20;7	22;4	15;2	5;1
Central	*Enterobacter*	Cephalosporins (4th generation)	Inpatient	-	2;0	-	-

Central	*Enterobacter*	Cephalosporins (4th generation)	Outpatient	17;9	13;3	7;3	4;1
Central	*Enterobacter*	Fluoroquinolones	Inpatient	-	4;2	-	-
Central	*Enterobacter*	Fluoroquinolones	Outpatient	25;13	25;11	19;9	9;4
Central	*Enterococcus*	Aminopenicillins	Outpatient	4;2	6;2	9;4	-
Central	*Enterococcus faecalis*	Fluoroquinolones	Outpatient	10;5	14;5	21;4	-
Central	*Enterococcus faecalis*	Vancomycin	Outpatient	8;2	9;0	15;9	-
Central	*Enterococcus faecium*	Fluoroquinolones	Outpatient	-	-	2;0	-
Central	*Enterococcus faecium*	Vancomycin	Outpatient	-	-	1;1	-
Central	*Escherichia coli*	Aminoglycosides	Inpatient	14;5	4;2	2;1	2;1
Central	*Escherichia coli*	Aminoglycosides	Outpatient	182;61 (33.5%; 12.9-63.2)	185;54 (29.2%; 17.1-45.1)	141;39 (27.7%; 16.6-42.3)	31;14 (45.2%; 5.3-92.4)
Central	*Escherichia coli*	Aminopenicillins	Inpatient	10;6	4;2	2;2	2;1
Central	*Escherichia coli*	Aminopenicillins	Outpatient	175;133 (76%; 52.4-90.1)	168;131 (78%; 67.3-85.9)	130;102 (78.5%; 68.4-86)	31;20 (64.5%; 22.4-92)
Central	*Escherichia coli*	Carbapenems	Inpatient	2;0	2;0	-	-
Central	*Escherichia coli*	Carbapenems	Outpatient	145;30 (20.7%; 7.2-46.8)	162;10 (6.2%; 2.1-16.6)	108;4 (3.7%; 0.9-14.7)	15;6
Central	*Escherichia coli*	Cephalosporins (3rd generation)	Inpatient	14;3	4;2	2;1	2;0
Central	*Escherichia coli*	Cephalosporins (3rd generation)	Outpatient	170;74 (43.5%; 14.4-77.9)	163;49 (30.1%; 14-53.2)	111;34 (30.6%; 18-47)	31;8 (25.8%; 1.5-89)
Central	*Escherichia coli*	Fluoroquinolones	Inpatient	12;5	4;2	-	2;0
Central	*Escherichia coli*	Fluoroquinolones	Outpatient	171;84 (49.1%; 20-78.8)	182;66 (36.3%; 19.9-56.5)	139;59 (42.4%; 33.6-51.8)	31;14 (45.2%; 2.6-96.3)
Central	*Escherichia coli*	Trimethoprim/Sulfamethoxazole	Inpatient	-	2;2	-	-
Central	*Escherichia coli*	Trimethoprim/Sulfamethoxazole	Outpatient	10;9	24;15	10;6	-
Central	*Haemophilus influenzae*	Aminopenicillins	Outpatient	-	1;0	-	1;0
Central	*Haemophilus influenzae*	Cephalosporins (3rd generation)	Outpatient	-	1;0	-	1;0
Central	*Klebsiella pneumoniae*	Aminoglycosides	Inpatient	2;1	-	4;2	-
Central	*Klebsiella pneumoniae*	Aminoglycosides	Outpatient	41;11 (26.8%; 14.1-45.1)	54;12 (22.2%; 10.8-40.2)	36;10 (27.8%; 11.7-52.6)	5;1
Central	*Klebsiella pneumoniae*	Carbapenems	Inpatient	1;0	-	3;0	-
Central	*Klebsiella pneumoniae*	Carbapenems	Outpatient	28;3	42;2 (4.8%; 0.8-22.9)	23;1	3;1
Central	*Klebsiella pneumoniae*	Cephalosporins (3rd generation)	Inpatient	2;0	-	4;2	-
Central	*Klebsiella pneumoniae*	Cephalosporins (3rd generation)	Outpatient	35;14 (40%; 18.9-65.5)	51;16 (31.4%; 14.2-55.7)	27;10	5;1

Central	*Klebsiella pneumoniae*	Fluoroquinolones	Inpatient	1;1	-	3;1	-
Central	*Klebsiella pneumoniae*	Fluoroquinolones	Outpatient	39;13 (33.3%; 14.3-59.9)	54;12 (22.2%; 7.6-49.9)	36;13 (36.1%; 22.1-53)	5;2
Central	*Morganella*	Cephalosporins (3rd generation)	Outpatient	3;0	-	2;1	-
Central	*Morganella*	Cephalosporins (4th generation)	Outpatient	1;0	-	2;1	-
Central	*Morganella*	Fluoroquinolones	Outpatient	3;0	-	2;1	-
Central	*Neisseria gonorrhoeae*	Cephalosporins (3rd generation)	Outpatient	2;0	1;0	-	2;0
Central	*Neisseria gonorrhoeae*	Fluoroquinolones	Outpatient	2;0	-	-	2;1
Central	*Neisseria gonorrhoeae*	Macrolides	Outpatient	2;2	-	-	1;1
Central	*Neisseria gonorrhoeae*	Quinolones	Outpatient	2;2	1;1	-	2;2
Central	*Neisseria gonorrhoeae*	Tetracyclines	Outpatient	2;2	1;1	-	2;2
Central	*Non-typhoidal Salmonella*	Fluoroquinolones	Inpatient	-	1;1	-	-
Central	*Non-typhoidal Salmonella*	Fluoroquinolones	Outpatient	7;3	5;2	6;3	7;5
Central	*Proteus*	Aminoglycosides	Inpatient	-	1;0	-	1;0
Central	*Proteus*	Aminoglycosides	Outpatient	20;5	13;4	21;5	2;1
Central	*Proteus*	Aminopenicillins	Inpatient	-	1;1	-	1;0
Central	*Proteus*	Aminopenicillins	Outpatient	19;8	14;8	20;10	2;0
Central	*Proteus*	Cephalosporins (3rd generation)	Inpatient	-	1;1	-	1;0
Central	*Proteus*	Cephalosporins (3rd generation)	Outpatient	12;2	14;2	13;3	2;1
Central	*Proteus*	Cephalosporins (4th generation)	Inpatient	-	1;0	-	-
Central	*Proteus*	Cephalosporins (4th generation)	Outpatient	6;0	7;2	9;2	2;0
Central	*Proteus*	Fluoroquinolones	Inpatient	-	1;1	-	1;0
Central	*Proteus*	Fluoroquinolones	Outpatient	20;2	15;3	22;5	2;1
Central	*Pseudomonas aeruginosa*	Aminoglycosides	Inpatient	1;1	-	-	3;0
Central	*Pseudomonas aeruginosa*	Aminoglycosides	Outpatient	11;5	14;5	16;5	3;1
Central	*Pseudomonas aeruginosa*	Beta-lactam combinations (Antipseudomonal)	Outpatient	6;0	9;1	7;0	2;0
Central	*Pseudomonas aeruginosa*	Carbapenems	Inpatient	1;1	-	-	-
Central	*Pseudomonas aeruginosa*	Carbapenems	Outpatient	9;2	12;2	9;0	2;0
Central	*Pseudomonas aeruginosa*	Cephalosporins (3rd generation)	Inpatient	1;1	-	-	1;1
Central	*Pseudomonas aeruginosa*	Cephalosporins (3rd generation)	Outpatient	8;5	12;9	11;6	2;2

Central	*Pseudomonas aeruginosa*	Cephalosporins (4th generation)	Inpatient	1;1	-	-	-
Central	*Pseudomonas aeruginosa*	Cephalosporins (4th generation)	Outpatient	5;4	6;0	10;1	2;0
Central	*Pseudomonas aeruginosa*	Fluoroquinolones	Inpatient	1;1	-	-	3;0
Central	*Pseudomonas aeruginosa*	Fluoroquinolones	Outpatient	11;2	14;5	16;7	3;2
Central	*Salmonella Typhi*	Fluoroquinolones	Inpatient	1;0	-	-	-
Central	*Salmonella Typhi*	Fluoroquinolones	Outpatient	1;0	-	1;0	1;0
Central	*Serratia*	Aminoglycosides	Inpatient	-	3;1	1;1	-
Central	*Serratia*	Aminoglycosides	Outpatient	16;8	25;7	5;1	2;1
Central	*Serratia*	Carbapenems	Inpatient	-	3;0	1;0	-
Central	*Serratia*	Carbapenems	Outpatient	14;5	26;3	7;1	2;0
Central	*Serratia*	Cephalosporins (3rd generation)	Inpatient	-	3;1	1;0	-
Central	*Serratia*	Cephalosporins (3rd generation)	Outpatient	16;6	25;15	8;5	2;2
Central	*Serratia*	Cephalosporins (4th generation)	Inpatient	-	2;0	1;1	-
Central	*Serratia*	Cephalosporins (4th generation)	Outpatient	15;7	17;0	6;3	2;0
Central	*Serratia*	Fluoroquinolones	Inpatient	-	2;1	1;1	-
Central	*Serratia*	Fluoroquinolones	Outpatient	16;10	24;13	7;3	2;2
Central	*Shigella*	Fluoroquinolones	Outpatient	1;0	3;1	-	9;0
Central	*Staphylococcus aureus*	Beta-lactam combinations (Antipseudomonal)	Outpatient	3;1	6;4	4;0	10;1
Central	*Staphylococcus aureus*	Fluoroquinolones	Inpatient	-	-	1;0	-
Central	*Staphylococcus aureus*	Fluoroquinolones	Outpatient	121;59 (48.8%; 24.3-73.8)	106;38 (35.8%; 20.7-54.4)	88;35 (39.8%; 27.1-54)	34;18 (52.9%; 20.8-82.8)
Central	*Staphylococcus aureus*	Macrolides	Inpatient	7;2	1;1	-	5;2
Central	*Staphylococcus aureus*	Macrolides	Outpatient	132;98 (74.2%; 49.4-89.5)	114;67 (58.8%; 37.2-77.4)	101;55 (54.5%; 40.4-67.8)	30;24 (80%; 4.1-99.7)
Central	*Staphylococcus aureus*	Methicillin	Inpatient	-	-	-	5;3
Central	*Staphylococcus aureus*	Methicillin	Outpatient	91;76 (83.5%; 22.6-98.9)	49;30 (61.2%; 46.5-74.2)	36;29 (80.6%; 52.9-93.9)	6;3
Central	*Staphylococcus aureus*	Trimethoprim/Sulfamethoxazole	Inpatient	-	-	1;1	-
Central	*Staphylococcus aureus*	Trimethoprim/Sulfamethoxazole	Outpatient	11;2	15;5	6;1	-
Central	*Streptococcus agalactiae*	Fluoroquinolones	Outpatient	16;1	20;1	42;8 (19%; 9.8-33.7)	-
Central	*Streptococcus agalactiae*	Macrolides	Outpatient	4;0	2;1	20;12	-

Central	*Streptococcus agalactiae*	Penicillins	Outpatient	1;0	1;0	9;2	-
Central	*Streptococcus pneumoniae*	Macrolides	Outpatient	1;0	-	-	1;0
Eastern	*Acinetobacter baumannii*	Aminoglycosides	Inpatient	46;14 (30.4%; 1.1-94.7)	26;9	74;49 (66.2%; 36.7-86.9)	2;1
Eastern	*Acinetobacter baumannii*	Aminoglycosides	Outpatient	6;0	17;5	14;7	-
Eastern	*Acinetobacter baumannii*	Beta-lactam combinations (Antipseudomonal)	Inpatient	8;3	7;3	31;18 (58.1%; 6.3-96.6)	2;0
Eastern	*Acinetobacter baumannii*	Beta-lactam combinations (Antipseudomonal)	Outpatient	1;1	4;3	3;1	-
Eastern	*Acinetobacter baumannii*	Carbapenems	Inpatient	22;10	18;9	63;22 (34.9%; 10.3-71.4)	1;0
Eastern	*Acinetobacter baumannii*	Carbapenems	Outpatient	4;2	5;1	10;1	-
Eastern	*Acinetobacter baumannii*	Cephalosporins (3rd generation)	Inpatient	26;12	25;11	58;31 (53.4%; 5.4-95.8)	2;2
Eastern	*Acinetobacter baumannii*	Cephalosporins (3rd generation)	Outpatient	5;1	9;8	11;4	-
Eastern	*Acinetobacter baumannii*	Cephalosporins (4th generation)	Inpatient	16;11	15;12	34;26 (76.5%; 39.6-94.2)	-
Eastern	*Acinetobacter baumannii*	Cephalosporins (4th generation)	Outpatient	6;2	2;0	1;0	-
Eastern	*Acinetobacter baumannii*	Fluoroquinolones	Inpatient	19;14	14;11	63;46 (73%; 60.5-82.7)	1;1
Eastern	*Acinetobacter baumannii*	Fluoroquinolones	Outpatient	2;0	12;8	14;6	-
Eastern	*Citrobacter*	Aminoglycosides	Inpatient	28;15	98;46 (46.9%; 40.7-53.2)	89;47 (52.8%; 40.3-64.9)	2;0
Eastern	*Citrobacter*	Aminoglycosides	Outpatient	30;12 (40%; 30.6-50.2)	40;19 (47.5%; 33.8-61.6)	93;31 (33.3%; 24.8-43.1)	6;2
Eastern	*Citrobacter*	Beta-lactam combinations (Antipseudomonal)	Inpatient	5;3	2;2	4;1	-
Eastern	*Citrobacter*	Beta-lactam combinations (Antipseudomonal)	Outpatient	8;1	4;2	7;0	1;1
Eastern	*Citrobacter*	Carbapenems	Inpatient	6;1	19;2	35;9 (25.7%; 11.7-47.5)	1;0
Eastern	*Citrobacter*	Carbapenems	Outpatient	4;0	11;1	37;2 (5.4%; 1-24.9)	3;0
Eastern	*Citrobacter*	Cephalosporins (3rd generation)	Inpatient	33;23 (69.7%; 52.3-82.8)	83;45 (54.2%; 46.4-61.8)	96;62 (64.6%; 59.8-69.1)	1;0
Eastern	*Citrobacter*	Cephalosporins (3rd generation)	Outpatient	38;17 (44.7%; 23.4-68.2)	41;25 (61%; 45.9-74.2)	80;27 (33.8%; 23.8-45.4)	5;2
Eastern	*Citrobacter*	Cephalosporins (4th generation)	Inpatient	7;4	6;3	5;1	1;0
Eastern	*Citrobacter*	Cephalosporins (4th generation)	Outpatient	11;2	11;4	14;0	5;3
Eastern	*Citrobacter*	Fluoroquinolones	Inpatient	33;17 (51.5%; 31.3-71.2)	97;40 (41.2%; 29.6-53.9)	93;49 (52.7%; 32.2-72.3)	5;3
Eastern	*Citrobacter*	Fluoroquinolones	Outpatient	45;15 (33.3%; 27.6-39.6)	51;13 (25.5%; 14.4-41.1)	106;31 (29.2%; 13.2-53)	9;5
Eastern	*Enterobacter*	Aminoglycosides	Inpatient	44;19 (43.2%; 35.3-51.4)	107;54 (50.5%; 30.3-70.5)	143;68 (47.6%; 35.4-60)	7;1

Eastern	*Enterobacter*	Aminoglycosides	Outpatient	40;15 (37.5%; 25.8-50.8)	44;20 (45.5%; 20.7-72.7)	130;46 (35.4%; 28.3-43.2)	18;5
Eastern	*Enterobacter*	Beta-lactam combinations (Antipseudomonal)	Inpatient	5;4	30;7 (23.3%; 2.9-75.7)	33;6 (18.2%; 1.7-74.2)	2;0
Eastern	*Enterobacter*	Beta-lactam combinations (Antipseudomonal)	Outpatient	8;5	6;4	12;7	6;0
Eastern	*Enterobacter*	Carbapenems	Inpatient	15;1	55;8 (14.5%; 4.2-40.1)	70;6 (8.6%; 3.8-18.3)	2;0
Eastern	*Enterobacter*	Carbapenems	Outpatient	10;1	14;6	43;1 (2.3%; 1.3-4.2)	-
Eastern	*Enterobacter*	Cephalosporins (4th generation)	Inpatient	10;7	21;16	14;7	3;2
Eastern	*Enterobacter*	Cephalosporins (4th generation)	Outpatient	6;3	3;1	4;2	2;1
Eastern	*Enterobacter*	Fluoroquinolones	Inpatient	47;22 (46.8%; 31.1-63.2)	102;42 (41.2%; 22-63.5)	148;69 (46.6%; 23.4-71.4)	5;2
Eastern	*Enterobacter*	Fluoroquinolones	Outpatient	47;17 (36.2%; 23.8-50.7)	74;31 (41.9%; 28.2-56.9)	142;52 (36.6%; 23.3-52.4)	22;6
Eastern	*Enterococcus*	Aminopenicillins	Inpatient	96;55 (57.3%; 25.9-83.8)	43;20 (46.5%; 14.2-82.1)	55;11 (20%; 2.2-73.3)	2;2
Eastern	*Enterococcus*	Aminopenicillins	Outpatient	83;28 (33.7%; 18.4-53.4)	105;33 (31.4%; 14.5-55.4)	80;8 (10%; 0.2-83.9)	-
Eastern	*Enterococcus faecalis*	Fluoroquinolones	Inpatient	85;48 (56.5%; 18.9-87.8)	38;18 (47.4%; 16.1-80.9)	87;36 (41.4%; 5.8-89.1)	2;2
Eastern	*Enterococcus faecalis*	Fluoroquinolones	Outpatient	63;16 (25.4%; 10.2-50.5)	114;18 (15.8%; 5.3-38.6)	119;26 (21.8%; 3.8-66.2)	-
Eastern	*Enterococcus faecalis*	Vancomycin	Inpatient	109;15 (13.8%; 3.3-43)	42;6 (14.3%; 7.9-24.6)	82;7 (8.5%; 5.4-13.3)	2;0
Eastern	*Enterococcus faecalis*	Vancomycin	Outpatient	22;1	46;2 (4.3%; 2.1-8.8)	29;6	-
Eastern	*Enterococcus faecium*	Fluoroquinolones	Inpatient	46;38 (82.6%; 27.9-98.3)	22;20	6;5	-
Eastern	*Enterococcus faecium*	Fluoroquinolones	Outpatient	-	-	1;0	-
Eastern	*Enterococcus faecium*	Vancomycin	Inpatient	44;1 (2.3%; 0.7-7)	19;0	6;0	-
Eastern	*Enterococcus faecium*	Vancomycin	Outpatient	-	-	1;0	-
Eastern	*Escherichia coli*	Aminoglycosides	Inpatient	411;171 (41.6%; 35.6-47.8)	746;294 (39.4%; 30.8-48.8)	887;369 (41.6%; 30.5-53.6)	17;11
Eastern	*Escherichia coli*	Aminoglycosides	Outpatient	660;184 (27.9%; 22.6-33.8)	656;190 (29%; 21.3-38)	1121;306 (27.3%; 21.7-33.7)	38;13 (34.2%; 14.8-60.8)
Eastern	*Escherichia coli*	Aminopenicillins	Inpatient	201;179 (89.1%; 85.3-91.9)	484;358 (74%; 29.3-95.1)	685;546 (79.7%; 44.9-95)	16;13
Eastern	*Escherichia coli*	Aminopenicillins	Outpatient	571;479 (83.9%; 80.1-87.1)	673;531 (78.9%; 50.8-93.1)	907;691 (76.2%; 57.1-88.5)	39;35 (89.7%; 32.4-99.4)
Eastern	*Escherichia coli*	Carbapenems	Inpatient	213;17 (8%; 4-15.2)	382;71 (18.6%; 7.5-39)	573;58 (10.1%; 2.9-29.5)	5;0
Eastern	*Escherichia coli*	Carbapenems	Outpatient	274;13 (4.7%; 2-10.7)	382;49 (12.8%; 2.5-46.1)	584;33 (5.7%; 1.5-19.4)	7;0

Eastern	*Escherichia coli*	Cephalosporins (3rd generation)	Inpatient	406;263 (64.8%; 46.5-79.6)	638;437 (68.5%; 56.8-78.2)	888;642 (72.3%; 66.8-77.2)	18;16
Eastern	*Escherichia coli*	Cephalosporins (3rd generation)	Outpatient	631;244 (38.7%; 28.1-50.4)	694;322 (46.4%; 38.6-54.4)	1029;473 (46%; 41.4-50.6)	36;23 (63.9%; 31.2-87.3)
Eastern	*Escherichia coli*	Fluoroquinolones	Inpatient	316;186 (58.9%; 44.4-72)	575;330 (57.4%; 53.4-61.3)	754;483 (64.1%; 51.3-75.1)	22;14
Eastern	*Escherichia coli*	Fluoroquinolones	Outpatient	694;294 (42.4%; 32.9-52.4)	735;336 (45.7%; 42.6-48.9)	1140;551 (48.3%; 39.2-57.6)	49;19 (38.8%; 16-67.9)
Eastern	*Escherichia coli*	Trimethoprim/Sulfamethoxazole	Inpatient	127;120 (94.5%; 92.3-96.1)	278;233 (83.8%; 73.1-90.8)	408;329 (80.6%; 72.1-87.1)	2;2
Eastern	*Escherichia coli*	Trimethoprim/Sulfamethoxazole	Outpatient	287;252 (87.8%; 85.3-90)	377;277 (73.5%; 66.3-79.6)	381;282 (74%; 51-88.6)	8;8
Eastern	*Haemophilus influenzae*	Aminopenicillins	Inpatient	1;0	-	7;2	-
Eastern	*Haemophilus influenzae*	Aminopenicillins	Outpatient	2;0	3;1	16;4	-
Eastern	*Haemophilus influenzae*	Cephalosporins (3rd generation)	Inpatient	1;0	-	4;1	-
Eastern	*Haemophilus influenzae*	Cephalosporins (3rd generation)	Outpatient	2;0	6;1	9;3	-
Eastern	*Klebsiella pneumoniae*	Aminoglycosides	Inpatient	393;276 (70.2%; 32.4-92.1)	364;240 (65.9%; 31-89.3)	275;155 (56.4%; 31.7-78.3)	33;25 (75.8%; 32.4-95.3)
Eastern	*Klebsiella pneumoniae*	Aminoglycosides	Outpatient	130;53 (40.8%; 23.9-60.1)	138;61 (44.2%; 31.9-57.3)	239;115 (48.1%; 31.7-64.9)	14;8
Eastern	*Klebsiella pneumoniae*	Carbapenems	Inpatient	289;15 (5.2%; 0.4-45.6)	198;18 (9.1%; 0.9-52.6)	215;12 (5.6%; 1.1-24.8)	12;0
Eastern	*Klebsiella pneumoniae*	Carbapenems	Outpatient	88;29 (33%; 3.5-86.8)	81;13 (16%; 2.8-56)	188;11 (5.9%; 1.3-22.9)	4;0
Eastern	*Klebsiella pneumoniae*	Cephalosporins (3rd generation)	Inpatient	425;391 (92%; 79.3-97.2)	331;294 (88.8%; 80-94)	283;238 (84.1%; 74.7-90.4)	30;30 (100%; 86.2-102)
Eastern	*Klebsiella pneumoniae*	Cephalosporins (3rd generation)	Outpatient	189;119 (63%; 32.4-85.8)	162;96 (59.3%; 43.4-73.4)	243;142 (58.4%; 49.9-66.5)	14;10
Eastern	*Klebsiella pneumoniae*	Fluoroquinolones	Inpatient	344;171 (49.7%; 38.6-60.8)	299;174 (58.2%; 26.6-84.2)	262;124 (47.3%; 24.5-71.4)	38;28 (73.7%; 57.8-85.1)
Eastern	*Klebsiella pneumoniae*	Fluoroquinolones	Outpatient	126;58 (46%; 36.6-55.7)	128;59 (46.1%; 30.4-62.6)	225;94 (41.8%; 18.5-69.4)	17;9
Eastern	*Morganella*	Cephalosporins (3rd generation)	Inpatient	1;0	4;2	13;6	-
Eastern	*Morganella*	Cephalosporins (3rd generation)	Outpatient	20;18	16;13	20;4	-
Eastern	*Morganella*	Cephalosporins (4th generation)	Outpatient	3;2	2;0	1;0	-
Eastern	*Morganella*	Fluoroquinolones	Inpatient	1;1	4;1	8;5	-
Eastern	*Morganella*	Fluoroquinolones	Outpatient	12;7	6;1	18;8	-
Eastern	*Neisseria gonorrhoeae*	Cephalosporins (3rd generation)	Inpatient	-	2;0	2;1	-
Eastern	*Neisseria gonorrhoeae*	Cephalosporins (3rd generation)	Outpatient	19;1	16;3	33;4 (12.1%; 5.2-25.9)	-

Eastern	*Neisseria gonorrhoeae*	Fluoroquinolones	Inpatient	-	2;1	1;1	-
Eastern	*Neisseria gonorrhoeae*	Fluoroquinolones	Outpatient	18;10	20;7	30;12 (40%; 9.6-80.7)	-
Eastern	*Neisseria gonorrhoeae*	Macrolides	Inpatient	-	1;1	1;0	-
Eastern	*Neisseria gonorrhoeae*	Macrolides	Outpatient	17;11	10;5	23;20	-
Eastern	*Neisseria gonorrhoeae*	Quinolones	Inpatient	-	-	1;1	-
Eastern	*Neisseria gonorrhoeae*	Quinolones	Outpatient	3;2	2;2	4;4	-
Eastern	*Neisseria gonorrhoeae*	Tetracyclines	Inpatient	-	-	1;1	-
Eastern	*Neisseria gonorrhoeae*	Tetracyclines	Outpatient	2;1	8;8	11;9	-
Eastern	*Non-typhoidal Salmonella*	Fluoroquinolones	Inpatient	34;4 (11.8%; 0.5-77.6)	41;4 (9.8%; 0.7-61.6)	20;9	-
Eastern	*Non-typhoidal Salmonella*	Fluoroquinolones	Outpatient	15;2	25;4	25;7	1;0
Eastern	*Proteus*	Aminoglycosides	Inpatient	133;47 (35.3%; 23.4-49.5)	168;69 (41.1%; 28.2-55.3)	238;112 (47.1%; 37.7-56.6)	4;3
Eastern	*Proteus*	Aminoglycosides	Outpatient	182;36 (19.8%; 8.3-40.1)	164;53 (32.3%; 24.3-41.5)	263;78 (29.7%; 22-38.7)	33;14 (42.4%; 16.2-73.7)
Eastern	*Proteus*	Aminopenicillins	Inpatient	86;63 (73.3%; 59.2-83.8)	109;62 (56.9%; 35.5-75.9)	197;145 (73.6%; 49.1-88.9)	4;4
Eastern	*Proteus*	Aminopenicillins	Outpatient	146;123 (84.2%; 71.8-91.8)	209;160 (76.6%; 49.7-91.5)	215;158 (73.5%; 62.4-82.2)	27;20
Eastern	*Proteus*	Cephalosporins (3rd generation)	Inpatient	108;52 (48.1%; 44-52.3)	160;76 (47.5%; 33.5-61.9)	254;148 (58.3%; 48.8-67.2)	5;4
Eastern	*Proteus*	Cephalosporins (3rd generation)	Outpatient	203;59 (29.1%; 20.5-39.4)	281;96 (34.2%; 26.4-42.8)	268;95 (35.4%; 29.8-41.5)	35;28 (80%; 20.8-98.4)
Eastern	*Proteus*	Cephalosporins (4th generation)	Inpatient	24;13	32;7 (21.9%; 3.6-67.6)	20;10	-
Eastern	*Proteus*	Cephalosporins (4th generation)	Outpatient	16;7	29;6	5;1	16;13
Eastern	*Proteus*	Fluoroquinolones	Inpatient	117;55 (47%; 31.2-63.4)	120;41 (34.2%; 17.1-56.6)	232;109 (47%; 36.6-57.6)	5;3
Eastern	*Proteus*	Fluoroquinolones	Outpatient	219;50 (22.8%; 14.1-34.8)	306;98 (32%; 25.7-39.1)	283;75 (26.5%; 16.9-38.9)	37;10 (27%; 5.3-70.9)
Eastern	*Pseudomonas aeruginosa*	Aminoglycosides	Inpatient	108;27 (25%; 17.5-34.3)	221;62 (28.1%; 16.1-44.3)	238;75 (31.5%; 17.3-50.2)	16;7
Eastern	*Pseudomonas aeruginosa*	Aminoglycosides	Outpatient	67;12 (17.9%; 10.2-29.6)	98;26 (26.5%; 11.1-51.2)	177;45 (25.4%; 16.2-37.5)	23;7
Eastern	*Pseudomonas aeruginosa*	Beta-lactam combinations (Antipseudomonal)	Inpatient	30;4 (13.3%; 0.9-72.9)	78;11 (14.1%; 10.8-18.2)	92;18 (19.6%; 5.9-48.5)	5;0
Eastern	*Pseudomonas aeruginosa*	Beta-lactam combinations (Antipseudomonal)	Outpatient	18;7	29;10	53;7 (13.2%; 7.2-23)	10;1
Eastern	*Pseudomonas aeruginosa*	Carbapenems	Inpatient	51;12 (23.5%; 5.2-63.4)	126;32 (25.4%; 12.5-44.7)	156;43 (27.6%; 17.7-40.2)	3;0

Eastern	*Pseudomonas aeruginosa*	Carbapenems	Outpatient	34;0 (0%; 0-0)	61;6 (9.8%; 1.9-38.3)	105;19 (18.1%; 7.8-36.7)	5;0
Eastern	*Pseudomonas aeruginosa*	Cephalosporins (3rd generation)	Inpatient	110;69 (62.7%; 50.9-73.2)	211;130 (61.6%; 38.4-80.5)	231;154 (66.7%; 54.5-76.9)	17;10
Eastern	*Pseudomonas aeruginosa*	Cephalosporins (3rd generation)	Outpatient	58;28 (48.3%; 39.1-57.6)	99;62 (62.6%; 52-72.2)	169;78 (46.2%; 38.2-54.3)	22;14
Eastern	*Pseudomonas aeruginosa*	Cephalosporins (4th generation)	Inpatient	42;16 (38.1%; 17.9-63.5)	76;31 (40.8%; 12.5-76.9)	46;24 (52.2%; 18.4-84)	3;0
Eastern	*Pseudomonas aeruginosa*	Cephalosporins (4th generation)	Outpatient	21;8	38;12 (31.6%; 24.4-39.7)	13;5	5;3
Eastern	*Pseudomonas aeruginosa*	Fluoroquinolones	Inpatient	86;23 (26.7%; 19.2-36)	147;41 (27.9%; 22.8-33.6)	189;68 (36%; 22.3-52.4)	23;4
Eastern	*Pseudomonas aeruginosa*	Fluoroquinolones	Outpatient	63;7 (11.1%; 5.9-20)	96;22 (22.9%; 13.3-36.5)	180;31 (17.2%; 9.8-28.6)	28;4
Eastern	*Salmonella Paratyphi*	Fluoroquinolones	Outpatient	1;0	-	1;1	-
Eastern	*Salmonella Typhi*	Fluoroquinolones	Inpatient	3;1	1;1	10;2	1;0
Eastern	*Salmonella Typhi*	Fluoroquinolones	Outpatient	11;1	19;7	15;6	-
Eastern	*Serratia*	Aminoglycosides	Inpatient	9;4	19;7	24;13	-
Eastern	*Serratia*	Aminoglycosides	Outpatient	24;8	32;15 (46.9%; 31.6-62.7)	55;12 (21.8%; 13.7-32.9)	2;1
Eastern	*Serratia*	Carbapenems	Inpatient	6;3	9;3	9;3	-
Eastern	*Serratia*	Carbapenems	Outpatient	10;1	14;4	8;0	-
Eastern	*Serratia*	Cephalosporins (3rd generation)	Inpatient	9;6	16;12	29;19	-
Eastern	*Serratia*	Cephalosporins (3rd generation)	Outpatient	24;13	28;8	59;18 (30.5%; 24.3-37.5)	3;2
Eastern	*Serratia*	Cephalosporins (4th generation)	Inpatient	2;2	5;3	6;4	-
Eastern	*Serratia*	Cephalosporins (4th generation)	Outpatient	6;2	12;2	3;1	-
Eastern	*Serratia*	Fluoroquinolones	Inpatient	5;3	11;6	22;10	-
Eastern	*Serratia*	Fluoroquinolones	Outpatient	24;4	36;5 (13.9%; 8.6-21.7)	52;11 (21.2%; 11.2-36.4)	1;0
Eastern	*Shigella*	Fluoroquinolones	Inpatient	7;1	3;1	8;4	-
Eastern	*Shigella*	Fluoroquinolones	Outpatient	39;3 (7.7%; 2.4-22)	35;5 (14.3%; 7.7-25.1)	44;6 (13.6%; 1.9-55.8)	-
Eastern	*Staphylococcus aureus*	Beta-lactam combinations (Antipseudomonal)	Inpatient	41;17 (41.5%; 18.2-69.3)	14;12	5;1	-
Eastern	*Staphylococcus aureus*	Beta-lactam combinations (Antipseudomonal)	Outpatient	12;1	1;0	20;3	-
Eastern	*Staphylococcus aureus*	Fluoroquinolones	Inpatient	248;81 (32.7%; 26-40.1)	413;169 (40.9%; 38.8-43.1)	597;188 (31.5%; 17.5-49.9)	18;8
Eastern	*Staphylococcus aureus*	Fluoroquinolones	Outpatient	595;137 (23%; 20.1-26.2)	617;163 (26.4%; 19.8-34.4)	1073;276 (25.7%; 14.5-41.4)	62;15 (24.2%; 15.2-36.3)
Eastern	*Staphylococcus aureus*	Macrolides	Inpatient	496;258 (52%; 39.7-64.1)	668;388 (58.1%; 55.3-60.8)	739;405 (54.8%; 43.9-65.2)	6;2

Eastern	*Staphylococcus aureus*	Macrolides	Outpatient	657;454 (69.1%; 65.8-72.2)	772;471 (61%; 57.5-64.4)	935;589 (63%; 59.9-66)	56;22 (39.3%; 17.7-66)
Eastern	*Staphylococcus aureus*	Methicillin	Inpatient	207;76 (36.7%; 23.2-52.7)	172;91 (52.9%; 40.2-65.2)	317;151 (47.6%; 33.8-61.9)	19;12
Eastern	*Staphylococcus aureus*	Methicillin	Outpatient	352;175 (49.7%; 25.7-73.8)	358;214 (59.8%; 29.4-84.2)	401;191 (47.6%; 29.8-66.1)	65;27 (41.5%; 22.2-63.8)
Eastern	*Staphylococcus aureus*	Trimethoprim/Sulfamethoxazole	Inpatient	160;117 (73.1%; 59.1-83.7)	194;156 (80.4%; 73.4-86)	363;279 (76.9%; 68.7-83.4)	3;1
Eastern	*Staphylococcus aureus*	Trimethoprim/Sulfamethoxazole	Outpatient	319;295 (92.5%; 88.5-95.1)	367;319 (86.9%; 70.8-94.8)	478;377 (78.9%; 61.6-89.7)	13;11
Eastern	*Streptococcus agalactiae*	Fluoroquinolones	Inpatient	2;0	3;0	1;0	-
Eastern	*Streptococcus agalactiae*	Fluoroquinolones	Outpatient	12;3	22;6	3;1	-
Eastern	*Streptococcus agalactiae*	Macrolides	Inpatient	2;0	2;0	2;0	-
Eastern	*Streptococcus agalactiae*	Macrolides	Outpatient	8;1	14;8	6;3	-
Eastern	*Streptococcus agalactiae*	Penicillins	Inpatient	1;0	2;0	2;0	-
Eastern	*Streptococcus agalactiae*	Penicillins	Outpatient	8;1	11;8	4;1	-
Eastern	*Streptococcus pneumoniae*	Carbapenems	Inpatient	5;0	4;0	16;2	-
Eastern	*Streptococcus pneumoniae*	Carbapenems	Outpatient	19;0	4;0	20;1	-
Eastern	*Streptococcus pneumoniae*	Cephalosporins (3rd generation)	Inpatient	29;3	23;2	36;9 (25%; 6.7-60.7)	-
Eastern	*Streptococcus pneumoniae*	Cephalosporins (3rd generation)	Outpatient	36;0 (0%; 0-0)	24;3	40;5 (12.5%; 4-33)	2;0
Eastern	*Streptococcus pneumoniae*	Fluoroquinolones	Inpatient	16;3	11;2	30;3 (10%; 0.5-72.1)	-
Eastern	*Streptococcus pneumoniae*	Fluoroquinolones	Outpatient	29;9	20;7	42;3 (7.1%; 3.9-12.7)	-
Eastern	*Streptococcus pneumoniae*	Macrolides	Inpatient	30;15 (50%; 31.3-68.7)	22;5	47;30 (63.8%; 50.7-75.2)	-
Eastern	*Streptococcus pneumoniae*	Macrolides	Outpatient	35;28 (80%; 61.9-90.8)	21;7	57;36 (63.2%; 36.7-83.5)	-
Eastern	*Streptococcus pneumoniae*	Penicillins	Inpatient	32;10 (31.2%; 11.4-61.7)	30;12 (40%; 31.8-48.8)	38;20 (52.6%; 28.9-75.2)	-
Eastern	*Streptococcus pneumoniae*	Penicillins	Outpatient	37;7 (18.9%; 4.6-53.3)	20;10	48;21 (43.8%; 34.4-53.6)	2;1
Eastern	*Streptococcus pneumoniae*	Trimethoprim/Sulfamethoxazole	Inpatient	16;14	12;11	23;18	-
Eastern	*Streptococcus pneumoniae*	Trimethoprim/Sulfamethoxazole	Outpatient	26;25	15;13	29;28	-
Eastern	*Streptococcus pyogenes*	Macrolides	Inpatient	25;9	35;17 (48.6%; 24.2-73.7)	31;14 (45.2%; 17-76.8)	-
Eastern	*Streptococcus pyogenes*	Macrolides	Outpatient	23;8	115;32 (27.8%; 26.1-29.6)	48;19 (39.6%; 29.9-50.2)	-
Southern	*Acinetobacter baumannii*	Aminoglycosides	Inpatient	9;5	12;4	27;13	-
Southern	*Acinetobacter baumannii*	Aminoglycosides	Outpatient	-	1;0	3;2	-

Southern	*Acinetobacter baumannii*	Carbapenems	Inpatient	6;1	5;1	14;6	-
Southern	*Acinetobacter baumannii*	Carbapenems	Outpatient	-	-	3;0	-
Southern	*Acinetobacter baumannii*	Cephalosporins (3rd generation)	Inpatient	9;7	21;12	25;19	-
Southern	*Acinetobacter baumannii*	Cephalosporins (3rd generation)	Outpatient	1;0	3;2	2;1	-
Southern	*Acinetobacter baumannii*	Cephalosporins (4th generation)	Inpatient	1;0	-	-	-
Southern	*Acinetobacter baumannii*	Fluoroquinolones	Inpatient	9;4	26;9	26;9	-
Southern	*Acinetobacter baumannii*	Fluoroquinolones	Outpatient	1;0	4;2	3;1	-
Southern	*Citrobacter*	Aminoglycosides	Inpatient	2;0	13;1	8;2	-
Southern	*Citrobacter*	Aminoglycosides	Outpatient	2;0	10;1	10;3	-
Southern	*Citrobacter*	Carbapenems	Inpatient	1;0	2;0	5;1	-
Southern	*Citrobacter*	Carbapenems	Outpatient	1;0	1;0	1;0	-
Southern	*Citrobacter*	Cephalosporins (3rd generation)	Inpatient	2;0	11;2	7;5	-
Southern	*Citrobacter*	Cephalosporins (3rd generation)	Outpatient	2;0	11;3	11;6	-
Southern	*Citrobacter*	Cephalosporins (4th generation)	Inpatient	1;0	-	1;1	-
Southern	*Citrobacter*	Cephalosporins (4th generation)	Outpatient	1;0	-	-	-
Southern	*Citrobacter*	Fluoroquinolones	Inpatient	3;0	11;1	7;3	-
Southern	*Citrobacter*	Fluoroquinolones	Outpatient	2;0	10;1	6;2	-
Southern	*Enterobacter*	Aminoglycosides	Inpatient	4;1	22;4	7;1	-
Southern	*Enterobacter*	Aminoglycosides	Outpatient	3;0	5;0	2;0	-
Southern	*Enterobacter*	Carbapenems	Inpatient	3;1	3;1	3;0	-
Southern	*Enterobacter*	Carbapenems	Outpatient	1;0	-	2;0	-
Southern	*Enterobacter*	Cephalosporins (4th generation)	Inpatient	1;0	-	-	-
Southern	*Enterobacter*	Cephalosporins (4th generation)	Outpatient	1;0	-	-	-
Southern	*Enterobacter*	Fluoroquinolones	Inpatient	3;1	20;9	7;2	-
Southern	*Enterobacter*	Fluoroquinolones	Outpatient	3;0	7;0	2;0	-
Southern	*Enterococcus*	Aminopenicillins	Inpatient	4;4	23;17	11;7	-
Southern	*Enterococcus*	Aminopenicillins	Outpatient	6;5	12;9	2;1	-
Southern	*Enterococcus faecalis*	Fluoroquinolones	Inpatient	1;1	22;2	10;5	-
Southern	*Enterococcus faecalis*	Fluoroquinolones	Outpatient	4;1	12;1	2;1	-
Southern	*Enterococcus faecalis*	Vancomycin	Inpatient	1;0	20;4	7;1	-

Southern	*Enterococcus faecalis*	Vancomycin	Outpatient	3;0	14;2	2;0	-
Southern	*Enterococcus faecium*	Fluoroquinolones	Inpatient	5;5	7;6	1;1	-
Southern	*Enterococcus faecium*	Fluoroquinolones	Outpatient	2;2	2;0	-	-
Southern	*Enterococcus faecium*	Vancomycin	Inpatient	3;1	6;0	3;0	-
Southern	*Enterococcus faecium*	Vancomycin	Outpatient	1;0	2;1	-	-
Southern	*Escherichia coli*	Aminoglycosides	Inpatient	128;29 (22.7%; 8.7-47.4)	215;64 (29.8%; 3.3-84.2)	138;54 (39.1%; 25.7-54.4)	-
Southern	*Escherichia coli*	Aminoglycosides	Outpatient	197;66 (33.5%; 8.2-74)	260;78 (30%; 5.7-75.1)	168;67 (39.9%; 27-54.3)	-
Southern	*Escherichia coli*	Aminopenicillins	Inpatient	112;90 (80.4%; 69.9-87.8)	206;186 (90.3%; 86.5-93.1)	115;90 (78.3%; 76.2-80.2)	-
Southern	*Escherichia coli*	Aminopenicillins	Outpatient	170;152 (89.4%; 85.1-92.6)	212;182 (85.8%; 82.2-88.9)	130;103 (79.2%; 78.5-80)	-
Southern	*Escherichia coli*	Carbapenems	Inpatient	31;0 (0%; 0-0)	53;5 (9.4%; 0.3-78.1)	46;6 (13%; 2.9-43)	-
Southern	*Escherichia coli*	Carbapenems	Outpatient	53;0 (0%; 0-0)	27;2	38;7 (18.4%; 17.4-19.5)	-
Southern	*Escherichia coli*	Cephalosporins (3rd generation)	Inpatient	77;37 (48.1%; 35.3-61)	196;130 (66.3%; 50.9-78.9)	83;41 (49.4%; 39.5-59.3)	-
Southern	*Escherichia coli*	Cephalosporins (3rd generation)	Outpatient	76;26 (34.2%; 7.4-77.3)	189;69 (36.5%; 35.5-37.6)	86;25 (29.1%; 25-33.5)	-
Southern	*Escherichia coli*	Fluoroquinolones	Inpatient	98;43 (43.9%; 25.1-64.6)	203;83 (40.9%; 40-41.7)	94;51 (54.3%; 54.1-54.4)	-
Southern	*Escherichia coli*	Fluoroquinolones	Outpatient	107;34 (31.8%; 26-38.2)	208;69 (33.2%; 22.5-45.8)	107;29 (27.1%; 24.7-29.6)	-
Southern	*Escherichia coli*	Trimethoprim/Sulfamethoxazole	Inpatient	45;39 (86.7%; 77.2-92.6)	70;65 (92.9%; 78.4-97.9)	77;70 (90.9%; 89.8-91.9)	-
Southern	*Escherichia coli*	Trimethoprim/Sulfamethoxazole	Outpatient	90;77 (85.6%; 77.8-90.9)	62;50 (80.6%; 46.3-95.3)	65;50 (76.9%; 71.9-81.3)	-
Southern	*Haemophilus influenzae*	Aminopenicillins	Inpatient	-	1;1	-	-
Southern	*Klebsiella pneumoniae*	Aminoglycosides	Inpatient	33;11 (33.3%; 0.9-96.6)	53;12 (22.6%; 0.5-94.9)	28;14	-
Southern	*Klebsiella pneumoniae*	Aminoglycosides	Outpatient	18;3	24;1	6;3	-
Southern	*Klebsiella pneumoniae*	Carbapenems	Inpatient	26;0	11;0	17;0	-
Southern	*Klebsiella pneumoniae*	Carbapenems	Outpatient	12;0	1;0	-	-
Southern	*Klebsiella pneumoniae*	Cephalosporins (3rd generation)	Inpatient	28;23	55;43 (78.2%; 71.5-83.6)	28;19	-
Southern	*Klebsiella pneumoniae*	Cephalosporins (3rd generation)	Outpatient	17;11	22;7	6;2	-
Southern	*Klebsiella pneumoniae*	Fluoroquinolones	Inpatient	34;19 (55.9%; 32.5-76.9)	54;21 (38.9%; 29.7-48.9)	26;11	-
Southern	*Klebsiella pneumoniae*	Fluoroquinolones	Outpatient	18;3	21;4	3;0	-
Southern	*Morganella*	Cephalosporins (3rd generation)	Inpatient	2;0	3;2	2;0	-

Southern	*Morganella*	Cephalosporins (3rd generation)	Outpatient	-	4;1	2;2	-
Southern	*Morganella*	Fluoroquinolones	Inpatient	3;1	3;2	2;0	-
Southern	*Morganella*	Fluoroquinolones	Outpatient	-	5;0	2;1	-
Southern	*Neisseria gonorrhoeae*	Cephalosporins (3rd generation)	Inpatient	-	-	1;1	-
Southern	*Neisseria gonorrhoeae*	Fluoroquinolones	Inpatient	-	-	1;1	-
Southern	*Neisseria gonorrhoeae*	Quinolones	Inpatient	-	-	1;1	-
Southern	*Neisseria gonorrhoeae*	Tetracyclines	Outpatient	-	2;1	-	-
Southern	*Non-typhoidal Salmonella*	Fluoroquinolones	Inpatient	3;2	2;2	7;4	-
Southern	*Non-typhoidal Salmonella*	Fluoroquinolones	Outpatient	8;0	20;1	1;0	-
Southern	*Proteus*	Aminoglycosides	Inpatient	112;36 (32.1%; 14.6-56.7)	137;34 (24.8%; 6-63)	66;22 (33.3%; 19.4-51)	-
Southern	*Proteus*	Aminoglycosides	Outpatient	78;24 (30.8%; 8.7-67.4)	91;18 (19.8%; 4.6-56)	40;17 (42.5%; 18.1-71.1)	-
Southern	*Proteus*	Aminopenicillins	Inpatient	99;79 (79.8%; 70.4-86.7)	108;79 (73.1%; 54.3-86.2)	44;30 (68.2%; 55.3-78.8)	-
Southern	*Proteus*	Aminopenicillins	Outpatient	70;51 (72.9%; 61.3-82)	79;61 (77.2%; 60.6-88.2)	21;18	-
Southern	*Proteus*	Cephalosporins (3rd generation)	Inpatient	43;19 (44.2%; 18.6-73.3)	119;46 (38.7%; 34.7-42.8)	60;26 (43.3%; 32.6-54.8)	-
Southern	*Proteus*	Cephalosporins (3rd generation)	Outpatient	36;12 (33.3%; 25-42.9)	83;32 (38.6%; 36.7-40.5)	29;12	-
Southern	*Proteus*	Cephalosporins (4th generation)	Inpatient	15;2	5;2	3;1	-
Southern	*Proteus*	Cephalosporins (4th generation)	Outpatient	2;0	-	2;0	-
Southern	*Proteus*	Fluoroquinolones	Inpatient	56;13 (23.2%; 8.4-49.8)	126;33 (26.2%; 7.1-62.3)	66;19 (28.8%; 23-35.4)	-
Southern	*Proteus*	Fluoroquinolones	Outpatient	54;11 (20.4%; 3.6-63.9)	101;14 (13.9%; 2.2-53.4)	29;12	-
Southern	*Pseudomonas aeruginosa*	Aminoglycosides	Inpatient	70;18 (25.7%; 20.6-31.6)	109;49 (45%; 22.4-69.8)	129;45 (34.9%; 28.1-42.3)	1;0
Southern	*Pseudomonas aeruginosa*	Aminoglycosides	Outpatient	54;11 (20.4%; 17-24.2)	74;21 (28.4%; 8.3-63.3)	32;17 (53.1%; 29.9-75)	-
Southern	*Pseudomonas aeruginosa*	Carbapenems	Inpatient	10;1	18;6	34;9 (26.5%; 13.7-45)	-
Southern	*Pseudomonas aeruginosa*	Carbapenems	Outpatient	10;0	8;8	5;1	-
Southern	*Pseudomonas aeruginosa*	Cephalosporins (3rd generation)	Inpatient	48;21 (43.8%; 41.3-46.2)	90;70 (77.8%; 76.1-79.4)	100;59 (59%; 54.2-63.7)	-
Southern	*Pseudomonas aeruginosa*	Cephalosporins (3rd generation)	Outpatient	49;13 (26.5%; 15.7-41.2)	59;40 (67.8%; 37.9-87.9)	21;11	-
Southern	*Pseudomonas aeruginosa*	Cephalosporins (4th generation)	Inpatient	5;2	-	9;1	-
Southern	*Pseudomonas aeruginosa*	Cephalosporins (4th generation)	Outpatient	2;0	-	1;0	-
Southern	*Pseudomonas aeruginosa*	Fluoroquinolones	Inpatient	68;11 (16.2%; 14.5-18)	86;17 (19.8%; 9.7-36.2)	131;35 (26.7%; 25.8-27.7)	1;0

Southern	*Pseudomonas aeruginosa*	Fluoroquinolones	Outpatient	67;22 (32.8%; 28.4-37.6)	85;14 (16.5%; 5.4-40.3)	25;4	-
Southern	*Salmonella Typhi*	Fluoroquinolones	Inpatient	5;3	-	-	-
Southern	*Salmonella Typhi*	Fluoroquinolones	Outpatient	52;8 (15.4%; 2.2-59.7)	51;10 (19.6%; 3.5-61.9)	2;0	-
Southern	*Serratia*	Aminoglycosides	Inpatient	4;2	13;3	16;1	-
Southern	*Serratia*	Aminoglycosides	Outpatient	4;0	9;1	14;2	-
Southern	*Serratia*	Carbapenems	Inpatient	1;0	1;0	5;1	-
Southern	*Serratia*	Carbapenems	Outpatient	2;0	-	2;0	-
Southern	*Serratia*	Cephalosporins (3rd generation)	Inpatient	4;2	13;7	16;11	-
Southern	*Serratia*	Cephalosporins (3rd generation)	Outpatient	4;1	9;4	14;6	-
Southern	*Serratia*	Cephalosporins (4th generation)	Outpatient	2;1	-	-	-
Southern	*Serratia*	Fluoroquinolones	Inpatient	3;2	13;5	18;8	-
Southern	*Serratia*	Fluoroquinolones	Outpatient	4;2	8;2	13;1	-
Southern	*Shigella*	Fluoroquinolones	Inpatient	2;0	7;2	4;1	-
Southern	*Shigella*	Fluoroquinolones	Outpatient	9;0	20;3	8;1	-
Southern	*Staphylococcus aureus*	Fluoroquinolones	Inpatient	186;59 (31.7%; 30.4-33.1)	212;45 (21.2%; 11.4-36)	297;70 (23.6%; 19-28.9)	-
Southern	*Staphylococcus aureus*	Fluoroquinolones	Outpatient	133;32 (24.1%; 22.6-25.6)	158;23 (14.6%; 13.6-15.6)	130;36 (27.7%; 23.2-32.7)	-
Southern	*Staphylococcus aureus*	Macrolides	Inpatient	152;71 (46.7%; 40.4-53.1)	206;72 (35%; 28.8-41.7)	273;89 (32.6%; 30-35.3)	-
Southern	*Staphylococcus aureus*	Macrolides	Outpatient	104;35 (33.7%; 16.4-56.7)	155;47 (30.3%; 22.4-39.6)	143;46 (32.2%; 20-47.4)	1;0
Southern	*Staphylococcus aureus*	Methicillin	Inpatient	88;35 (39.8%; 32.4-47.7)	171;88 (51.5%; 44.2-58.6)	262;150 (57.3%; 37.6-74.8)	-
Southern	*Staphylococcus aureus*	Methicillin	Outpatient	51;18 (35.3%; 14.4-63.8)	112;50 (44.6%; 29-61.4)	128;70 (54.7%; 22.8-83.2)	-
Southern	*Staphylococcus aureus*	Trimethoprim/Sulfamethoxazole	Inpatient	117;75 (64.1%; 48.9-77)	134;104 (77.6%; 72.5-82)	186;136 (73.1%; 71.5-74.7)	-
Southern	*Staphylococcus aureus*	Trimethoprim/Sulfamethoxazole	Outpatient	120;74 (61.7%; 38.8-80.3)	106;69 (65.1%; 61.2-68.8)	82;42 (51.2%; 45.6-56.8)	1;0
Southern	*Streptococcus agalactiae*	Fluoroquinolones	Inpatient	1;0	2;1	2;2	-
Southern	*Streptococcus agalactiae*	Fluoroquinolones	Outpatient	-	1;0	1;0	-
Southern	*Streptococcus agalactiae*	Macrolides	Inpatient	-	1;0	1;0	-
Southern	*Streptococcus agalactiae*	Macrolides	Outpatient	-	1;0	1;0	-
Southern	*Streptococcus agalactiae*	Penicillins	Inpatient	1;0	2;0	1;0	-

Southern	*Streptococcus agalactiae*	Penicillins	Outpatient	1;0	6;2	3;1	-
Southern	*Streptococcus pneumoniae*	Carbapenems	Inpatient	2;0	-	1;1	-
Southern	*Streptococcus pneumoniae*	Cephalosporins (3rd generation)	Inpatient	3;0	-	1;1	-
Southern	*Streptococcus pneumoniae*	Fluoroquinolones	Inpatient	2;0	2;0	2;1	-
Southern	*Streptococcus pneumoniae*	Fluoroquinolones	Outpatient	-	3;0	1;0	-
Southern	*Streptococcus pneumoniae*	Macrolides	Inpatient	2;0	2;0	2;2	-
Southern	*Streptococcus pneumoniae*	Macrolides	Outpatient	2;0	3;0	1;0	-
Southern	*Streptococcus pneumoniae*	Penicillins	Inpatient	1;0	2;2	3;2	-
Southern	*Streptococcus pneumoniae*	Penicillins	Outpatient	1;0	1;1	-	-
Southern	*Streptococcus pneumoniae*	Trimethoprim/Sulfamethoxazole	Inpatient	-	1;1	1;1	-
Southern	*Streptococcus pyogenes*	Macrolides	Inpatient	1;0	3;1	3;1	-
Southern	*Streptococcus pyogenes*	Macrolides	Outpatient	5;3	11;7	11;3	-
Western	*Acinetobacter baumannii*	Aminoglycosides	Inpatient	8;3	45;22 (48.9%; 35.1-62.8)	88;41 (46.6%; 39.3-54.1)	32;15 (46.9%; 40.3-53.6)
Western	*Acinetobacter baumannii*	Aminoglycosides	Outpatient	-	21;2	64;18 (28.1%; 18.1-40.9)	25;14
Western	*Acinetobacter baumannii*	Beta-lactam combinations (Antipseudomonal)	Inpatient	4;1	16;12	31;18 (58.1%; 50.3-65.5)	19;14
Western	*Acinetobacter baumannii*	Beta-lactam combinations (Antipseudomonal)	Outpatient	-	3;1	6;4	10;2
Western	*Acinetobacter baumannii*	Carbapenems	Inpatient	6;1	27;16	56;20 (35.7%; 26.1-46.7)	32;8 (25%; 8.5-54.4)
Western	*Acinetobacter baumannii*	Carbapenems	Outpatient	-	9;2	28;15	15;1
Western	*Acinetobacter baumannii*	Cephalosporins (3rd generation)	Inpatient	7;5	44;38 (86.4%; 49.9-97.6)	62;49 (79%; 54.2-92.3)	24;22
Western	*Acinetobacter baumannii*	Cephalosporins (3rd generation)	Outpatient	-	23;16	69;48 (69.6%; 56.2-80.3)	23;16
Western	*Acinetobacter baumannii*	Cephalosporins (4th generation)	Inpatient	5;2	25;19	27;14	11;8
Western	*Acinetobacter baumannii*	Cephalosporins (4th generation)	Outpatient	-	9;7	26;19	5;1
Western	*Acinetobacter baumannii*	Fluoroquinolones	Inpatient	8;3	45;21 (46.7%; 23.6-71.2)	72;28 (38.9%; 20.4-61.3)	33;15 (45.5%; 21.5-71.8)
Western	*Acinetobacter baumannii*	Fluoroquinolones	Outpatient	-	16;5	43;17 (39.5%; 23-58.8)	18;6
Western	*Citrobacter*	Aminoglycosides	Inpatient	13;7	49;20 (40.8%; 13-76)	84;31 (36.9%; 21.5-55.6)	40;25 (62.5%; 53.4-70.8)
Western	*Citrobacter*	Aminoglycosides	Outpatient	36;20 (55.6%; 29.1-79.2)	106;23 (21.7%; 10.2-40.3)	127;36 (28.3%; 20.4-38)	95;33 (34.7%; 21-51.5)
Western	*Citrobacter*	Beta-lactam combinations (Antipseudomonal)	Inpatient	-	5;1	23;15	21;13
Western	*Citrobacter*	Beta-lactam combinations (Antipseudomonal)	Outpatient	-	8;7	25;12	26;15

Western	*Citrobacter*	Carbapenems	Inpatient	3;2	17;5	41;7 (17.1%; 4.7-46.2)	25;5
Western	*Citrobacter*	Carbapenems	Outpatient	5;3	30;6 (20%; 3.1-66.1)	52;7 (13.5%; 3.9-37.4)	48;2 (4.2%; 0.4-33.7)
Western	*Citrobacter*	Cephalosporins (3rd generation)	Inpatient	13;9	47;28 (59.6%; 39.8-76.7)	88;50 (56.8%; 34.7-76.5)	43;31 (72.1%; 62.8-79.8)
Western	*Citrobacter*	Cephalosporins (3rd generation)	Outpatient	34;20 (58.8%; 27.6-84.2)	98;44 (44.9%; 28.1-62.9)	136;62 (45.6%; 36.9-54.6)	101;44 (43.6%; 33.5-54.2)
Western	*Citrobacter*	Cephalosporins (4th generation)	Inpatient	1;1	10;4	23;9	15;8
Western	*Citrobacter*	Cephalosporins (4th generation)	Outpatient	-	24;9	29;8	21;9
Western	*Citrobacter*	Fluoroquinolones	Inpatient	13;11	36;17 (47.2%; 22.6-73.3)	65;20 (30.8%; 22.9-40)	35;19 (54.3%; 36.6-70.9)
Western	*Citrobacter*	Fluoroquinolones	Outpatient	35;14 (40%; 22.2-60.9)	69;25 (36.2%; 19.6-57)	91;35 (38.5%; 27.1-51.2)	70;29 (41.4%; 34.9-48.2)
Western	*Enterobacter*	Aminoglycosides	Inpatient	46;23 (50%; 23.8-76.2)	123;54 (43.9%; 22.2-68.2)	142;62 (43.7%; 36.3-51.3)	100;48 (48%; 43.4-52.6)
Western	*Enterobacter*	Aminoglycosides	Outpatient	23;7	126;37 (29.4%; 13.7-52.1)	187;59 (31.6%; 18.1-48.9)	131;57 (43.5%; 35.5-51.9)
Western	*Enterobacter*	Beta-lactam combinations (Antipseudomonal)	Inpatient	6;3	21;10	34;19 (55.9%; 29.5-79.3)	33;19 (57.6%; 38.3-74.8)
Western	*Enterobacter*	Beta-lactam combinations (Antipseudomonal)	Outpatient	1;0	13;3	21;12	7;4
Western	*Enterobacter*	Carbapenems	Inpatient	9;2	73;25 (34.2%; 3.7-87.7)	89;20 (22.5%; 6-56.7)	75;13 (17.3%; 5.7-42)
Western	*Enterobacter*	Carbapenems	Outpatient	7;0	83;11 (13.3%; 0.8-73.4)	83;10 (12%; 2.3-44.2)	78;3 (3.8%; 1.5-9.6)
Western	*Enterobacter*	Cephalosporins (4th generation)	Inpatient	7;5	38;19 (50%; 41-59)	46;25 (54.3%; 29.4-77.3)	37;25 (67.6%; 54.9-78.1)
Western	*Enterobacter*	Cephalosporins (4th generation)	Outpatient	2;0	29;10	35;24 (68.6%; 46.1-84.8)	32;16 (50%; 42-58)
Western	*Enterobacter*	Fluoroquinolones	Inpatient	46;19 (41.3%; 22.7-62.8)	118;48 (40.7%; 25-58.5)	133;59 (44.4%; 37.8-51.1)	112;54 (48.2%; 35.1-61.6)
Western	*Enterobacter*	Fluoroquinolones	Outpatient	23;11	103;39 (37.9%; 32.6-43.4)	165;61 (37%; 19.7-58.4)	107;55 (51.4%; 48.8-54)
Western	*Enterococcus*	Aminopenicillins	Inpatient	1;1	8;3	18;8	5;3
Western	*Enterococcus*	Aminopenicillins	Outpatient	5;2	19;11	5;2	5;2
Western	*Enterococcus faecalis*	Fluoroquinolones	Inpatient	1;1	9;3	13;8	1;0
Western	*Enterococcus faecalis*	Fluoroquinolones	Outpatient	5;2	27;13	4;1	2;1
Western	*Enterococcus faecalis*	Vancomycin	Inpatient	-	7;2	15;2	1;0
Western	*Enterococcus faecalis*	Vancomycin	Outpatient	1;0	10;3	2;1	3;1
Western	*Enterococcus faecium*	Fluoroquinolones	Outpatient	-	-	-	1;1
Western	*Enterococcus faecium*	Vancomycin	Inpatient	-	-	1;0	-
Western	*Escherichia coli*	Aminoglycosides	Inpatient	373;153 (41%; 38.5-43.6)	746;276 (37%; 29.4-45.3)	908;354 (39%; 30.5-48.3)	421;154 (36.6%; 26.4-48.2)

Western	*Escherichia coli*	Aminoglycosides	Outpatient	699;274 (39.2%; 25.9-54.4)	1723;445 (25.8%; 20.4-32.1)	2175;627 (28.8%; 25.1-32.8)	1121;349 (31.1%; 29.5-32.8)
Western	*Escherichia coli*	Aminopenicillins	Inpatient	375;282 (75.2%; 69.8-79.9)	720;575 (79.9%; 53.2-93.3)	930;784 (84.3%; 68.6-93)	451;384 (85.1%; 79.9-89.2)
Western	*Escherichia coli*	Aminopenicillins	Outpatient	704;598 (84.9%; 82.5-87.1)	1684;1373 (81.5%; 72.6-88.1)	2238;1857 (83%; 78.1-87)	1222;949 (77.7%; 64.7-86.8)
Western	*Escherichia coli*	Carbapenems	Inpatient	54;12 (22.2%; 11.3-39.1)	298;36 (12.1%; 3.9-31.7)	521;64 (12.3%; 7.6-19.3)	285;22 (7.7%; 2.1-24.3)
Western	*Escherichia coli*	Carbapenems	Outpatient	108;28 (25.9%; 1.8-86.7)	759;44 (5.8%; 0.8-32.1)	950;51 (5.4%; 2.3-11.8)	599;22 (3.7%; 1.5-8.9)
Western	*Escherichia coli*	Cephalosporins (3rd generation)	Inpatient	417;258 (61.9%; 60.1-63.6)	774;382 (49.4%; 38.3-60.5)	1018;654 (64.2%; 54.1-73.3)	473;239 (50.5%; 38.5-62.5)
Western	*Escherichia coli*	Cephalosporins (3rd generation)	Outpatient	707;439 (62.1%; 48.9-73.7)	1683;644 (38.3%; 22.9-56.4)	2300;986 (42.9%; 34.1-52.1)	1234;509 (41.2%; 36.3-46.4)
Western	*Escherichia coli*	Fluoroquinolones	Inpatient	370;171 (46.2%; 37.3-55.4)	652;320 (49.1%; 43.5-54.7)	887;519 (58.5%; 42.5-72.9)	386;208 (53.9%; 46.5-61.2)
Western	*Escherichia coli*	Fluoroquinolones	Outpatient	766;331 (43.2%; 28.7-59)	1489;648 (43.5%; 34.6-52.9)	1969;918 (46.6%; 33.4-60.4)	954;452 (47.4%; 39.9-55)
Western	*Escherichia coli*	Trimethoprim/Sulfamethoxazole	Inpatient	40;35 (87.5%; 79.1-92.8)	136;93 (68.4%; 65.7-70.9)	311;266 (85.5%; 81.4-88.9)	141;109 (77.3%; 68.9-84)
Western	*Escherichia coli*	Trimethoprim/Sulfamethoxazole	Outpatient	160;105 (65.6%; 39.1-85)	488;351 (71.9%; 65.6-77.5)	838;662 (79%; 67-87.5)	376;277 (73.7%; 67-79.4)
Western	*Haemophilus influenzae*	Aminopenicillins	Outpatient	-	2;2	2;2	1;1
Western	*Haemophilus influenzae*	Cephalosporins (3rd generation)	Inpatient	-	1;0	-	-
Western	*Haemophilus influenzae*	Cephalosporins (3rd generation)	Outpatient	-	4;2	2;2	1;0
Western	*Klebsiella pneumoniae*	Aminoglycosides	Inpatient	111;81 (73%; 63.4-80.8)	444;230 (51.8%; 40.4-63)	528;201 (38.1%; 28.6-48.6)	218;103 (47.2%; 43.3-51.3)
Western	*Klebsiella pneumoniae*	Aminoglycosides	Outpatient	71;26 (36.6%; 23.2-52.5)	586;199 (34%; 25.8-43.2)	601;183 (30.4%; 26.8-34.4)	387;121 (31.3%; 25.6-37.5)
Western	*Klebsiella pneumoniae*	Carbapenems	Inpatient	58;10 (17.2%; 7.3-35.5)	242;47 (19.4%; 10.8-32.4)	345;39 (11.3%; 7.1-17.6)	137;10 (7.3%; 5.7-9.4)
Western	*Klebsiella pneumoniae*	Carbapenems	Outpatient	41;2 (4.9%; 0.4-36.9)	289;35 (12.1%; 3.1-37.3)	285;20 (7%; 3.5-13.5)	219;9 (4.1%; 2-8.3)
Western	*Klebsiella pneumoniae*	Cephalosporins (3rd generation)	Inpatient	116;88 (75.9%; 66.6-83.2)	455;297 (65.3%; 55-74.3)	570;404 (70.9%; 60.8-79.2)	222;150 (67.6%; 55.9-77.4)
Western	*Klebsiella pneumoniae*	Cephalosporins (3rd generation)	Outpatient	83;35 (42.2%; 27.6-58.2)	603;286 (47.4%; 40.8-54.2)	665;336 (50.5%; 38.4-62.6)	405;175 (43.2%; 33.6-53.3)
Western	*Klebsiella pneumoniae*	Fluoroquinolones	Inpatient	113;52 (46%; 26.9-66.4)	418;189 (45.2%; 41.8-48.7)	491;234 (47.7%; 40.6-54.8)	174;81 (46.6%; 30.4-63.5)
Western	*Klebsiella pneumoniae*	Fluoroquinolones	Outpatient	75;36 (48%; 33-63.4)	496;184 (37.1%; 28.7-46.3)	481;175 (36.4%; 32-41)	295;102 (34.6%; 30.3-39.1)
Western	*Morganella*	Cephalosporins (3rd generation)	Inpatient	2;2	8;2	11;4	13;4

Western	*Morganella*	Cephalosporins (3rd generation)	Outpatient	-	12;1	17;4	18;5
Western	*Morganella*	Cephalosporins (4th generation)	Inpatient	-	7;0	3;1	2;1
Western	*Morganella*	Cephalosporins (4th generation)	Outpatient	-	2;0	2;1	4;2
Western	*Morganella*	Fluoroquinolones	Inpatient	2;1	7;3	10;6	13;8
Western	*Morganella*	Fluoroquinolones	Outpatient	-	11;5	11;8	13;7
Western	*Neisseria gonorrhoeae*	Cephalosporins (3rd generation)	Inpatient	1;0	2;1	1;0	-
Western	*Neisseria gonorrhoeae*	Cephalosporins (3rd generation)	Outpatient	4;2	29;13	25;14	29;5
Western	*Neisseria gonorrhoeae*	Fluoroquinolones	Inpatient	1;0	2;1	1;0	-
Western	*Neisseria gonorrhoeae*	Fluoroquinolones	Outpatient	5;2	26;7	17;10	12;5
Western	*Neisseria gonorrhoeae*	Macrolides	Inpatient	-	1;0	-	-
Western	*Neisseria gonorrhoeae*	Macrolides	Outpatient	1;0	20;6	13;5	3;1
Western	*Neisseria gonorrhoeae*	Quinolones	Inpatient	1;1	-	-	-
Western	*Neisseria gonorrhoeae*	Quinolones	Outpatient	-	8;4	5;3	6;4
Western	*Neisseria gonorrhoeae*	Tetracyclines	Inpatient	-	1;1	-	-
Western	*Neisseria gonorrhoeae*	Tetracyclines	Outpatient	4;1	14;3	4;1	7;2
Western	*Non-typhoidal Salmonella*	Fluoroquinolones	Inpatient	4;3	22;1	17;4	13;3
Western	*Non-typhoidal Salmonella*	Fluoroquinolones	Outpatient	29;8	33;2 (6.1%; 2.1-16.2)	44;12 (27.3%; 9.7-56.6)	22;8
Western	*Proteus*	Aminoglycosides	Inpatient	70;30 (42.9%; 24.7-63.1)	104;41 (39.4%; 30.3-49.4)	150;52 (34.7%; 28.5-41.3)	74;24 (32.4%; 20.9-46.6)
Western	*Proteus*	Aminoglycosides	Outpatient	64;23 (35.9%; 29.3-43.2)	121;37 (30.6%; 25.5-36.2)	175;23 (13.1%; 8.4-20.1)	116;31 (26.7%; 11.2-51.3)
Western	*Proteus*	Aminopenicillins	Inpatient	72;54 (75%; 51.3-89.5)	94;67 (71.3%; 56.7-82.4)	131;88 (67.2%; 49.6-81)	73;51 (69.9%; 66-73.4)
Western	*Proteus*	Aminopenicillins	Outpatient	61;54 (88.5%; 79.9-93.7)	116;76 (65.5%; 57.9-72.5)	145;86 (59.3%; 49.8-68.2)	106;59 (55.7%; 45.6-65.3)
Western	*Proteus*	Cephalosporins (3rd generation)	Inpatient	76;43 (56.6%; 42-70.1)	99;40 (40.4%; 16.9-69.3)	161;68 (42.2%; 29.3-56.4)	76;30 (39.5%; 18.9-64.6)
Western	*Proteus*	Cephalosporins (3rd generation)	Outpatient	65;41 (63.1%; 41.1-80.7)	127;38 (29.9%; 18.2-45)	179;43 (24%; 13.8-38.4)	123;38 (30.9%; 11.2-61.2)
Western	*Proteus*	Cephalosporins (4th generation)	Inpatient	5;3	16;1	27;15	6;3
Western	*Proteus*	Cephalosporins (4th generation)	Outpatient	-	24;2	18;4	25;4
Western	*Proteus*	Fluoroquinolones	Inpatient	72;30 (41.7%; 33.4-50.4)	96;44 (45.8%; 38.5-53.3)	168;57 (33.9%; 26.8-41.9)	72;22 (30.6%; 25.1-36.6)
Western	*Proteus*	Fluoroquinolones	Outpatient	65;27 (41.5%; 29.6-54.5)	116;29 (25%; 21.8-28.4)	172;28 (16.3%; 9.9-25.5)	108;38 (35.2%; 24.6-47.5)

Western	*Pseudomonas aeruginosa*	Aminoglycosides	Inpatient	95;40 (42.1%; 39-45.2)	238;115 (48.3%; 42.9-53.8)	206;95 (46.1%; 32.1-60.8)	81;49 (60.5%; 37.3-79.7)
Western	*Pseudomonas aeruginosa*	Aminoglycosides	Outpatient	56;15 (26.8%; 14-45.1)	219;66 (30.1%; 27.1-33.3)	132;43 (32.6%; 25.2-40.9)	105;51 (48.6%; 22.5-75.4)
Western	*Pseudomonas aeruginosa*	Beta-lactam combinations (Antipseudomonal)	Inpatient	29;6	133;29 (21.8%; 10.8-39.1)	105;32 (30.5%; 11.3-60.3)	49;21 (42.9%; 21.2-67.6)
Western	*Pseudomonas aeruginosa*	Beta-lactam combinations (Antipseudomonal)	Outpatient	7;5	57;16 (28.1%; 12.5-51.7)	37;7 (18.9%; 8.9-35.8)	28;8
Western	*Pseudomonas aeruginosa*	Carbapenems	Inpatient	26;12	168;35 (20.8%; 10.1-38.1)	132;27 (20.5%; 7.9-43.5)	62;11 (17.7%; 4.1-52.2)
Western	*Pseudomonas aeruginosa*	Carbapenems	Outpatient	20;9	160;29 (18.1%; 10.4-29.6)	70;18 (25.7%; 9.1-54.4)	55;11 (20%; 4.2-58.9)
Western	*Pseudomonas aeruginosa*	Cephalosporins (3rd generation)	Inpatient	96;66 (68.8%; 58.7-77.3)	230;111 (48.3%; 28.1-69)	189;90 (47.6%; 33.1-62.6)	79;42 (53.2%; 34.8-70.7)
Western	*Pseudomonas aeruginosa*	Cephalosporins (3rd generation)	Outpatient	58;37 (63.8%; 46.2-78.3)	221;117 (52.9%; 43.6-62.1)	122;66 (54.1%; 36.7-70.5)	75;48 (64%; 37.1-84.3)
Western	*Pseudomonas aeruginosa*	Cephalosporins (4th generation)	Inpatient	27;12	97;14 (14.4%; 11.9-17.4)	72;32 (44.4%; 34.2-55.2)	19;9
Western	*Pseudomonas aeruginosa*	Cephalosporins (4th generation)	Outpatient	6;4	30;8 (26.7%; 9.4-56)	37;8 (21.6%; 9.1-43.2)	13;7
Western	*Pseudomonas aeruginosa*	Fluoroquinolones	Inpatient	70;38 (54.3%; 43.6-64.6)	236;101 (42.8%; 28.1-58.8)	186;78 (41.9%; 30.4-54.4)	76;31 (40.8%; 21.1-63.9)
Western	*Pseudomonas aeruginosa*	Fluoroquinolones	Outpatient	55;9 (16.4%; 4.8-43.2)	211;59 (28%; 21.6-35.4)	112;31 (27.7%; 19.5-37.7)	74;13 (17.6%; 13.4-22.7)
Western	*Salmonella Paratyphi*	Fluoroquinolones	Inpatient	-	1;0	1;1	-
Western	*Salmonella Paratyphi*	Fluoroquinolones	Outpatient	1;0	-	1;0	3;1
Western	*Salmonella Typhi*	Fluoroquinolones	Inpatient	4;1	9;0	8;0	1;0
Western	*Salmonella Typhi*	Fluoroquinolones	Outpatient	2;1	15;2	1;0	3;0
Western	*Serratia*	Aminoglycosides	Inpatient	4;2	9;6	23;9	18;9
Western	*Serratia*	Aminoglycosides	Outpatient	-	43;5 (11.6%; 7.4-17.7)	44;9 (20.5%; 12.6-31.4)	29;5
Western	*Serratia*	Carbapenems	Inpatient	2;0	5;3	14;1	12;3
Western	*Serratia*	Carbapenems	Outpatient	-	8;0	18;4	11;2
Western	*Serratia*	Cephalosporins (3rd generation)	Inpatient	4;3	9;4	24;16	19;11
Western	*Serratia*	Cephalosporins (3rd generation)	Outpatient	-	43;20 (46.5%; 36-57.3)	47;26 (55.3%; 34.9-74.1)	30;13 (43.3%; 27.1-61.1)
Western	*Serratia*	Cephalosporins (4th generation)	Inpatient	1;0	1;0	10;7	5;2
Western	*Serratia*	Cephalosporins (4th generation)	Outpatient	-	2;1	8;6	1;0
Western	*Serratia*	Fluoroquinolones	Inpatient	4;2	7;2	18;6	16;8
Western	*Serratia*	Fluoroquinolones	Outpatient	-	17;2	35;15 (42.9%; 26.3-61.2)	25;6

Western	*Shigella*	Fluoroquinolones	Inpatient	2;0	4;1	3;3	2;1
Western	*Shigella*	Fluoroquinolones	Outpatient	13;5	15;2	17;3	6;0
Western	*Staphylococcus aureus*	Beta-lactam combinations (Antipseudomonal)	Inpatient	3;3	24;6	11;2	11;3
Western	*Staphylococcus aureus*	Beta-lactam combinations (Antipseudomonal)	Outpatient	2;1	18;11	6;2	3;3
Western	*Staphylococcus aureus*	Fluoroquinolones	Inpatient	344;150 (43.6%; 41.4-45.9)	820;308 (37.6%; 26.8-49.8)	811;307 (37.9%; 29.9-46.5)	328;105 (32%; 19.9-47.1)
Western	*Staphylococcus aureus*	Fluoroquinolones	Outpatient	626;232 (37.1%; 32.7-41.7)	1092;386 (35.3%; 30.1-40.9)	1131;344 (30.4%; 26.4-34.8)	352;117 (33.2%; 24.2-43.7)
Western	*Staphylococcus aureus*	Macrolides	Inpatient	339;183 (54%; 45.2-62.5)	825;378 (45.8%; 34.2-58)	741;307 (41.4%; 33.7-49.6)	353;133 (37.7%; 22.5-55.8)
Western	*Staphylococcus aureus*	Macrolides	Outpatient	429;193 (45%; 37.6-52.7)	1038;406 (39.1%; 32.9-45.7)	882;370 (42%; 37.6-46.5)	339;122 (36%; 25.4-48.1)
Western	*Staphylococcus aureus*	Methicillin	Inpatient	125;85 (68%; 52.1-80.6)	491;249 (50.7%; 37.7-63.7)	447;233 (52.1%; 36-67.9)	310;148 (47.7%; 29.3-66.8)
Western	*Staphylococcus aureus*	Methicillin	Outpatient	234;145 (62%; 44.2-77)	726;334 (46%; 33-59.5)	720;400 (55.6%; 39.6-70.5)	281;122 (43.4%; 31-56.7)
Western	*Staphylococcus aureus*	Trimethoprim/Sulfamethoxazole	Inpatient	94;72 (76.6%; 42.6-93.5)	163;103 (63.2%; 53.1-72.2)	191;96 (50.3%; 29.3-71.1)	122;63 (51.6%; 33.7-69.2)
Western	*Staphylococcus aureus*	Trimethoprim/Sulfamethoxazole	Outpatient	177;126 (71.2%; 61.4-79.3)	180;131 (72.8%; 50.3-87.6)	305;202 (66.2%; 43.1-83.5)	146;86 (58.9%; 49.4-67.8)
Western	*Streptococcus agalactiae*	Fluoroquinolones	Inpatient	-	3;1	2;2	-
Western	*Streptococcus agalactiae*	Fluoroquinolones	Outpatient	-	4;2	4;1	-
Western	*Streptococcus agalactiae*	Macrolides	Inpatient	-	4;1	2;0	-
Western	*Streptococcus agalactiae*	Macrolides	Outpatient	-	3;2	2;1	-
Western	*Streptococcus agalactiae*	Penicillins	Inpatient	-	3;3	3;2	-
Western	*Streptococcus agalactiae*	Penicillins	Outpatient	-	5;4	3;0	-
Western	*Streptococcus pneumoniae*	Carbapenems	Inpatient	1;0	5;3	-	7;3
Western	*Streptococcus pneumoniae*	Carbapenems	Outpatient	2;2	1;0	4;1	1;1
Western	*Streptococcus pneumoniae*	Cephalosporins (3rd generation)	Inpatient	6;1	27;13	4;3	10;1
Western	*Streptococcus pneumoniae*	Cephalosporins (3rd generation)	Outpatient	1;1	37;25 (67.6%; 42.4-85.5)	8;3	5;2
Western	*Streptococcus pneumoniae*	Fluoroquinolones	Inpatient	3;0	29;9	5;2	8;1
Western	*Streptococcus pneumoniae*	Fluoroquinolones	Outpatient	3;2	35;21 (60%; 49.8-69.4)	9;3	9;2
Western	*Streptococcus pneumoniae*	Macrolides	Inpatient	4;1	25;12	7;4	1;0
Western	*Streptococcus pneumoniae*	Macrolides	Outpatient	5;2	38;23 (60.5%; 47.3-72.4)	10;5	9;4

Western	*Streptococcus pneumoniae*	Penicillins	Inpatient	1;1	13;7	4;4	10;3
Western	*Streptococcus pneumoniae*	Penicillins	Outpatient	4;4	11;8	6;3	8;4
Western	*Streptococcus pneumoniae*	Trimethoprim/Sulfamethoxazole	Inpatient	-	4;4	1;1	-
Western	*Streptococcus pneumoniae*	Trimethoprim/Sulfamethoxazole	Outpatient	4;4	2;1	4;2	2;2
Western	*Streptococcus pyogenes*	Macrolides	Inpatient	-	7;0	5;2	-
Western	*Streptococcus pyogenes*	Macrolides	Outpatient	1;1	12;5	6;3	-

[#]GBD=Global burden of disease; N = number of tested isolates; R = resistant isolates; %R and 95%CI are shown only if ≥30 isolates/ year; — information not available. Regions (**Central** - Gabon; **Eastern** - Malawi, Kenya, Uganda, Tanzania and Zambia; **Southern** - Eswatini and Zimbabwe; **Western** – Burkina Faso, Cameroon, Ghana, Nigeria, Senegal, and Sierra Leone).
