# Supplementary material for: Antimicrobial resistance in Africa: A retrospective analysis of data from 14 countries, 2016–2019
Source: PLoS Med. 2025 Jun 24;22(6):e1004638. doi: 10.1371/journal.pmed.1004638 (PMC12186946; doi:10.1371/journal.pmed.1004638)
Supplement: S11 Table — (PDF) [file pmed.1004638.s013.pdf]

S11 Table: AMR prevalence estimates for clinically important pathogens by region and specimen source (Blood/CSF and others)

Africa GBD# Region	Pathogen	Antimicrobial agent/class	Specimen source	2016 N; R(%R; 95%CI)	2017 N; R(%R; 95%CI)	2018 N; R(%R; 95%CI)	2019 N; R(%R; 95%CI)
Central	*Acinetobacter baumannii*	Aminoglycosides	Blood/CSF	1;1	1;0	5;1	-
Central	*Acinetobacter baumannii*	Aminoglycosides	Others	26;7	42;13 (31%; 17.2-49.2)	15;6	2;1
Central	*Acinetobacter baumannii*	Beta-lactam combinations (Antipseudomonal)	Blood/CSF	1;1	1;0	3;0	-
Central	*Acinetobacter baumannii*	Beta-lactam combinations (Antipseudomonal)	Others	20;4	37;6 (16.2%; 5.1-41.2)	12;3	-
Central	*Acinetobacter baumannii*	Carbapenems	Blood/CSF	-	1;0	3;1	-
Central	*Acinetobacter baumannii*	Carbapenems	Others	24;4	37;5 (13.5%; 5.9-28.2)	12;4	1;0
Central	*Acinetobacter baumannii*	Cephalosporins (3rd generation)	Blood/CSF	1;1	1;1	3;2	-
Central	*Acinetobacter baumannii*	Cephalosporins (3rd generation)	Others	26;18	36;27 (75%; 31.6-95.1)	14;9	2;2
Central	*Acinetobacter baumannii*	Cephalosporins (4th generation)	Blood/CSF	-	1;0	2;0	-
Central	*Acinetobacter baumannii*	Cephalosporins (4th generation)	Others	21;11	28;8	10;5	-
Central	*Acinetobacter baumannii*	Fluoroquinolones	Blood/CSF	1;1	1;0	5;2	-
Central	*Acinetobacter baumannii*	Fluoroquinolones	Others	26;13	40;23 (57.5%; 27.9-82.6)	13;5	2;1
Central	*Citrobacter*	Aminoglycosides	Blood/CSF	-	1;0	4;4	1;1
Central	*Citrobacter*	Aminoglycosides	Others	79;29 (36.7%; 22.8-53.2)	73;25 (34.2%; 21.9-49.1)	48;14 (29.2%; 15.6-47.8)	15;5
Central	*Citrobacter*	Beta-lactam combinations (Antipseudomonal)	Blood/CSF	-	1;0	3;2	1;0
Central	*Citrobacter*	Beta-lactam combinations (Antipseudomonal)	Others	54;8 (14.8%; 5.8-32.9)	58;9 (15.5%; 7.8-28.4)	36;6 (16.7%; 7.3-33.6)	13;0
Central	*Citrobacter*	Carbapenems	Blood/CSF	-	1;0	5;2	1;0
Central	*Citrobacter*	Carbapenems	Others	62;7 (11.3%; 2.9-35.2)	65;8 (12.3%; 5.3-26.2)	40;4 (10%; 1.6-43.4)	12;4
Central	*Citrobacter*	Cephalosporins (3rd generation)	Blood/CSF	-	1;0	5;4	1;1
Central	*Citrobacter*	Cephalosporins (3rd generation)	Others	63;37 (58.7%; 44.7-71.5)	66;29 (43.9%; 24.4-65.6)	42;23 (54.8%; 30.5-77)	15;9

Africa GBD[#] Region	Pathogen	Antimicrobial agent/class	Specimen source	2016 N; R(%R; 95%CI)	2017 N; R(%R; 95%CI)	2018 N; R(%R; 95%CI)	2019 N; R(%R; 95%CI)
Central	*Citrobacter*	Cephalosporins (4th generation)	Blood/CSF	-	1;0	4;3	1;0
Central	*Citrobacter*	Cephalosporins (4th generation)	Others	54;14 (25.9%; 14-42.9)	57;11 (19.3%; 6.3-46.1)	38;6 (15.8%; 4.2-44.6)	11;1
Central	*Citrobacter*	Fluoroquinolones	Blood/CSF	-	1;0	4;3	1;0
Central	*Citrobacter*	Fluoroquinolones	Others	78;36 (46.2%; 24.4-69.4)	71;31 (43.7%; 26.1-63)	48;21 (43.8%; 23.5-66.3)	15;8
Central	*Enterobacter*	Aminoglycosides	Blood/CSF	2;2	1;1	5;4	-
Central	*Enterobacter*	Aminoglycosides	Others	43;19 (44.2%; 14.8-78.2)	61;17 (27.9%; 17.2-41.8)	39;5 (12.8%; 5.9-25.8)	18;3
Central	*Enterobacter*	Beta-lactam combinations (Antipseudomonal)	Blood/CSF	2;1	1;0	4;3	-
Central	*Enterobacter*	Beta-lactam combinations (Antipseudomonal)	Others	30;4 (13.3%; 2.5-48.3)	40;3 (7.5%; 2.3-21.6)	25;2	11;2
Central	*Enterobacter*	Carbapenems	Blood/CSF	2;1	1;0	5;1	-
Central	*Enterobacter*	Carbapenems	Others	33;8 (24.2%; 7-57.7)	50;6 (12%; 5-25.9)	33;3 (9.1%; 3.3-22.8)	10;2
Central	*Enterobacter*	Cephalosporins (4th generation)	Blood/CSF	2;2	1;0	2;2	-
Central	*Enterobacter*	Cephalosporins (4th generation)	Others	30;9 (30%; 6.2-73.7)	35;4 (11.4%; 4.2-27.3)	22;2	9;3
Central	*Enterobacter*	Fluoroquinolones	Blood/CSF	2;2	1;0	5;4	-
Central	*Enterobacter*	Fluoroquinolones	Others	42;19 (45.2%; 19.7-73.6)	62;23 (37.1%; 23.1-53.7)	38;14 (36.8%; 15.6-64.8)	19;6
Central	*Enterococcus*	Aminopenicillins	Others	17;7	24;14	26;11	10;5
Central	*Enterococcus faecalis*	Fluoroquinolones	Others	21;10	38;21 (55.3%; 26.6-80.8)	38;18 (47.4%; 10.9-86.9)	10;9
Central	*Enterococcus faecalis*	Vancomycin	Others	17;4	18;2	19;10	1;0
Central	*Enterococcus faecium*	Fluoroquinolones	Others	1;0	-	2;0	-
Central	*Enterococcus faecium*	Vancomycin	Others	1;1	-	2;2	-
Central	*Escherichia coli*	Aminoglycosides	Blood/CSF	1;0	2;2	7;3	3;1
Central	*Escherichia coli*	Aminoglycosides	Others	340;122 (35.9%; 22.6-51.8)	483;167 (34.6%; 24.8-45.8)	389;119 (30.6%; 22.1-40.6)	116;52 (44.8%; 12.8-81.8)
Central	*Escherichia coli*	Aminopenicillins	Blood/CSF	1;1	2;2	6;6	3;3
Central	*Escherichia coli*	Aminopenicillins	Others	314;241 (76.8%; 62.7-86.7)	442;342 (77.4%; 71.8-82.2)	381;297 (78%; 71.3-83.4)	115;82 (71.3%; 46.9-87.5)

Africa GBD# Region	Pathogen	Antimicrobial agent/class	Specimen source	2016 N; R(%R; 95%CI)	2017 N; R(%R; 95%CI)	2018 N; R(%R; 95%CI)	2019 N; R(%R; 95%CI)
Central	*Escherichia coli*	Carbapenems	Blood/CSF	-	2;0	7;2	2;0
Central	*Escherichia coli*	Carbapenems	Others	279;34 (12.2%; 3.6-34.1)	431;24 (5.6%; 1.7-16.7)	350;23 (6.6%; 1.7-21.8)	70;15 (21.4%; 3.7-65.7)
Central	*Escherichia coli*	Cephalosporins (3rd generation)	Blood/CSF	1;1	2;1	7;5	3;1
Central	*Escherichia coli*	Cephalosporins (3rd generation)	Others	322;128 (39.8%; 20.6-62.6)	448;130 (29%; 18.8-41.9)	369;120 (32.5%; 19.8-48.4)	117;41 (35%; 6.7-80.2)
Central	*Escherichia coli*	Fluoroquinolones	Blood/CSF	-	2;2	6;5	3;1
Central	*Escherichia coli*	Fluoroquinolones	Others	328;151 (46%; 28-65.1)	477;211 (44.2%; 32.2-57)	382;184 (48.2%; 40.5-55.9)	118;53 (44.9%; 12.4-82.5)
Central	*Escherichia coli*	Trimethoprim/Sulfamethoxazole	Blood/CSF	-	1;1	6;3	-
Central	*Escherichia coli*	Trimethoprim/Sulfamethoxazole	Others	10;9	56;41 (73.2%; 3.5-99.5)	58;38 (65.5%; 54.4-75.1)	-
Central	*Haemophilus influenzae*	Aminopenicillins	Blood/CSF	-	-	2;0	-
Central	*Haemophilus influenzae*	Aminopenicillins	Others	-	1;0	-	2;0
Central	*Haemophilus influenzae*	Cephalosporins (3rd generation)	Blood/CSF	-	-	2;0	-
Central	*Haemophilus influenzae*	Cephalosporins (3rd generation)	Others	-	1;0	-	2;0
Central	*Klebsiella pneumoniae*	Aminoglycosides	Blood/CSF	2;2	6;5	11;5	1;0
Central	*Klebsiella pneumoniae*	Aminoglycosides	Others	81;34 (42%; 24.2-62.1)	160;55 (34.4%; 25.2-44.9)	131;47 (35.9%; 23.5-50.4)	20;5
Central	*Klebsiella pneumoniae*	Carbapenems	Blood/CSF	2;0	6;3	9;4	-
Central	*Klebsiella pneumoniae*	Carbapenems	Others	62;3 (4.8%; 1.3-16.8)	135;2 (1.5%; 0.3-6.6)	116;6 (5.2%; 1-22.4)	13;2
Central	*Klebsiella pneumoniae*	Cephalosporins (3rd generation)	Blood/CSF	2;2	6;6	11;7	2;0
Central	*Klebsiella pneumoniae*	Cephalosporins (3rd generation)	Others	72;36 (50%; 26.5-73.5)	147;57 (38.8%; 25.3-54.3)	126;67 (53.2%; 36.1-69.5)	20;7
Central	*Klebsiella pneumoniae*	Fluoroquinolones	Blood/CSF	2;1	6;3	11;5	2;0
Central	*Klebsiella pneumoniae*	Fluoroquinolones	Others	79;34 (43%; 22.7-66)	157;55 (35%; 24.4-47.4)	128;52 (40.6%; 32.6-49.1)	20;5
Central	*Morganella*	Cephalosporins (3rd generation)	Others	5;2	7;3	7;4	1;1
Central	*Morganella*	Cephalosporins (4th generation)	Others	3;1	7;2	6;2	1;1
Central	*Morganella*	Fluoroquinolones	Others	5;2	7;2	7;3	1;1

Africa GBD# Region	Pathogen	Antimicrobial agent/class	Specimen source	2016 N; R(%R; 95%CI)	2017 N; R(%R; 95%CI)	2018 N; R(%R; 95%CI)	2019 N; R(%R; 95%CI)
Central	*Neisseria gonorrhoeae*	Cephalosporins (3rd generation)	Others	2;0	4;0	5;2	4;0
Central	*Neisseria gonorrhoeae*	Fluoroquinolones	Others	2;0	3;1	5;2	3;1
Central	*Neisseria gonorrhoeae*	Macrolides	Others	2;2	-	3;1	1;1
Central	*Neisseria gonorrhoeae*	Quinolones	Others	2;2	1;1	-	2;2
Central	*Neisseria gonorrhoeae*	Tetracyclines	Others	2;2	2;1	5;4	3;3
Central	*Non-typhoidal Salmonella*	Fluoroquinolones	Blood/CSF	-	2;0	-	-
Central	*Non-typhoidal Salmonella*	Fluoroquinolones	Others	7;3	16;7	17;8	10;7
Central	*Proteus*	Aminoglycosides	Blood/CSF	-	1;0	2;0	1;1
Central	*Proteus*	Aminoglycosides	Others	27;6	34;15 (44.1%; 19.9-71.6)	42;13 (31%; 15.8-51.8)	11;5
Central	*Proteus*	Aminopenicillins	Blood/CSF	-	1;0	2;0	1;1
Central	*Proteus*	Aminopenicillins	Others	26;13	32;20 (62.5%; 46.7-76)	36;21 (58.3%; 30.4-81.8)	10;3
Central	*Proteus*	Cephalosporins (3rd generation)	Blood/CSF	-	1;0	2;0	1;1
Central	*Proteus*	Cephalosporins (3rd generation)	Others	19;3	35;10 (28.6%; 6.5-69.6)	30;11 (36.7%; 18.1-60.2)	11;4
Central	*Proteus*	Cephalosporins (4th generation)	Blood/CSF	-	1;0	-	1;0
Central	*Proteus*	Cephalosporins (4th generation)	Others	13;0	23;3	25;8	8;1
Central	*Proteus*	Fluoroquinolones	Blood/CSF	-	1;0	2;0	1;1
Central	*Proteus*	Fluoroquinolones	Others	27;2	34;11 (32.4%; 9.9-67.5)	46;14 (30.4%; 14.7-52.6)	10;4
Central	*Pseudomonas aeruginosa*	Aminoglycosides	Blood/CSF	-	-	2;1	-
Central	*Pseudomonas aeruginosa*	Aminoglycosides	Others	21;15	29;14	36;21 (58.3%; 31.8-80.8)	18;7
Central	*Pseudomonas aeruginosa*	Beta-lactam combinations (Antipseudomonal)	Blood/CSF	-	-	2;0	-
Central	*Pseudomonas aeruginosa*	Beta-lactam combinations (Antipseudomonal)	Others	14;2	23;4	26;5	12;4
Central	*Pseudomonas aeruginosa*	Carbapenems	Blood/CSF	-	-	2;0	-

Africa GBD# Region	Pathogen	Antimicrobial agent/class	Specimen source	2016 N; R(%R; 95%CI)	2017 N; R(%R; 95%CI)	2018 N; R(%R; 95%CI)	2019 N; R(%R; 95%CI)
Central	*Pseudomonas aeruginosa*	Carbapenems	Others	19;8	27;8	30;12 (40%; 9.8-80.4)	10;4
Central	*Pseudomonas aeruginosa*	Cephalosporins (3rd generation)	Blood/CSF	-	-	2;2	-
Central	*Pseudomonas aeruginosa*	Cephalosporins (3rd generation)	Others	18;14	28;22	32;26 (81.2%; 45.1-95.8)	10;10
Central	*Pseudomonas aeruginosa*	Cephalosporins (4th generation)	Blood/CSF	-	-	2;0	-
Central	*Pseudomonas aeruginosa*	Cephalosporins (4th generation)	Others	14;7	21;4	30;12 (40%; 9.1-81.7)	9;3
Central	*Pseudomonas aeruginosa*	Fluoroquinolones	Blood/CSF	-	-	2;2	-
Central	*Pseudomonas aeruginosa*	Fluoroquinolones	Others	20;11	30;16 (53.3%; 31.2-74.2)	37;25 (67.6%; 35.2-88.9)	18;9
Central	*Salmonella Typhi*	Fluoroquinolones	Blood/CSF	1;0	-	1;0	4;0
Central	*Salmonella Typhi*	Fluoroquinolones	Others	1;0	-	1;0	1;1
Central	*Serratia*	Aminoglycosides	Blood/CSF	-	6;4	7;7	-
Central	*Serratia*	Aminoglycosides	Others	51;19 (37.3%; 15.7-65.4)	94;18 (19.1%; 6.9-43.2)	62;28 (45.2%; 18.3-75.1)	10;6
Central	*Serratia*	Carbapenems	Blood/CSF	-	6;2	7;5	-
Central	*Serratia*	Carbapenems	Others	46;10 (21.7%; 9.2-43.2)	80;5 (6.2%; 1.7-20.2)	66;12 (18.2%; 8.8-33.9)	6;2
Central	*Serratia*	Cephalosporins (3rd generation)	Blood/CSF	-	6;3	7;6	-
Central	*Serratia*	Cephalosporins (3rd generation)	Others	53;20 (37.7%; 13.9-69.4)	82;37 (45.1%; 20.9-71.9)	70;43 (61.4%; 47.2-73.9)	9;7
Central	*Serratia*	Cephalosporins (4th generation)	Blood/CSF	-	5;2	6;2	-
Central	*Serratia*	Cephalosporins (4th generation)	Others	39;13 (33.3%; 14.2-60.1)	62;9 (14.5%; 5.9-31.5)	61;19 (31.1%; 22.6-41.2)	7;4
Central	*Serratia*	Fluoroquinolones	Blood/CSF	-	6;1	7;6	-
Central	*Serratia*	Fluoroquinolones	Others	49;27 (55.1%; 23.5-83.1)	82;36 (43.9%; 18.7-72.7)	65;31 (47.7%; 37.8-57.7)	10;6
Central	*Shigella*	Fluoroquinolones	Blood/CSF	-	-	-	3;0
Central	*Shigella*	Fluoroquinolones	Others	3;0	4;1	4;1	20;0
Central	*Staphylococcus aureus*	Beta-lactam combinations (Antipseudomonal)	Others	9;1	17;10	12;1	11;1

Africa GBD# Region	Pathogen	Antimicrobial agent/class	Specimen source	2016 N; R(%R; 95%CI)	2017 N; R(%R; 95%CI)	2018 N; R(%R; 95%CI)	2019 N; R(%R; 95%CI)
Central	*Staphylococcus aureus*	Fluoroquinolones	Blood/CSF	3;2	8;1	15;7	4;2
Central	*Staphylococcus aureus*	Fluoroquinolones	Others	173;88 (50.9%; 33.5-68)	205;90 (43.9%; 31.2-57.4)	208;99 (47.6%; 37.7-57.7)	69;41 (59.4%; 38.4-77.5)
Central	*Staphylococcus aureus*	Macrolides	Blood/CSF	3;1	6;3	17;10	7;2
Central	*Staphylococcus aureus*	Macrolides	Others	191;127 (66.5%; 46.5-81.9)	198;120 (60.6%; 46.5-73.2)	212;130 (61.3%; 46.4-74.4)	110;55 (50%; 9.6-90.4)
Central	*Staphylococcus aureus*	Methicillin	Blood/CSF	2;1	6;3	11;10	6;2
Central	*Staphylococcus aureus*	Methicillin	Others	140;108 (77.1%; 44.6-93.4)	124;84 (67.7%; 55.2-78.2)	146;98 (67.1%; 36.1-88.1)	80;34 (42.5%; 0.5-99)
Central	*Staphylococcus aureus*	Trimethoprim/Sulfamethoxazole	Blood/CSF	-	2;1	3;2	-
Central	*Staphylococcus aureus*	Trimethoprim/Sulfamethoxazole	Others	11;2	24;10	17;9	-
Central	*Streptococcus agalactiae*	Fluoroquinolones	Others	16;1	23;2	42;8 (19%; 9.8-33.7)	-
Central	*Streptococcus agalactiae*	Macrolides	Others	4;0	5;1	20;12	-
Central	*Streptococcus agalactiae*	Penicillins	Others	1;0	5;1	9;2	-
Central	*Streptococcus pneumoniae*	Carbapenems	Others	-	-	2;0	-
Central	*Streptococcus pneumoniae*	Cephalosporins (3rd generation)	Others	-	-	2;0	-
Central	*Streptococcus pneumoniae*	Fluoroquinolones	Others	-	-	2;1	-
Central	*Streptococcus pneumoniae*	Macrolides	Others	1;0	-	3;3	1;0
Central	*Streptococcus pneumoniae*	Penicillins	Others	-	-	3;2	-
Eastern	*Acinetobacter baumannii*	Aminoglycosides	Blood/CSF	49;22 (44.9%; 31.9-58.7)	77;29 (37.7%; 16.1-65.5)	120;52 (43.3%; 34.6-52.5)	3;3
Eastern	*Acinetobacter baumannii*	Aminoglycosides	Others	106;34 (32.1%; 9.2-68.8)	104;39 (37.5%; 20.6-58.2)	105;62 (59%; 32.8-81)	1;0
Eastern	*Acinetobacter baumannii*	Beta-lactam combinations (Antipseudomonal)	Blood/CSF	12;4	20;4	16;3	1;0
Eastern	*Acinetobacter baumannii*	Beta-lactam combinations (Antipseudomonal)	Others	27;13	53;16 (30.2%; 27-33.6)	58;23 (39.7%; 9.2-81)	1;0
Eastern	*Acinetobacter baumannii*	Carbapenems	Blood/CSF	37;13 (35.1%; 3.4-89.3)	36;3 (8.3%; 0.7-55.6)	43;7 (16.3%; 3.7-49.6)	-

Africa GBD# Region	Pathogen	Antimicrobial agent/class	Specimen source	2016 N; R(%R; 95%CI)	2017 N; R(%R; 95%CI)	2018 N; R(%R; 95%CI)	2019 N; R(%R; 95%CI)
Eastern	*Acinetobacter baumannii*	Carbapenems	Others	72;30 (41.7%; 13.9-76)	92;27 (29.3%; 7.3-68.7)	99;37 (37.4%; 13.3-69.8)	1;0
Eastern	*Acinetobacter baumannii*	Cephalosporins (3rd generation)	Blood/CSF	51;33 (64.7%; 22-92.3)	76;54 (71.1%; 14.7-97.2)	108;78 (72.2%; 39.1-91.3)	3;2
Eastern	*Acinetobacter baumannii*	Cephalosporins (3rd generation)	Others	89;42 (47.2%; 16.7-80)	99;48 (48.5%; 24.4-73.3)	91;46 (50.5%; 17-83.6)	1;1
Eastern	*Acinetobacter baumannii*	Cephalosporins (4th generation)	Blood/CSF	18;8	16;1	18;6	-
Eastern	*Acinetobacter baumannii*	Cephalosporins (4th generation)	Others	38;21 (55.3%; 50.8-59.6)	57;25 (43.9%; 12-81.7)	54;33 (61.1%; 41.1-78)	-
Eastern	*Acinetobacter baumannii*	Fluoroquinolones	Blood/CSF	46;26 (56.5%; 44.9-67.5)	74;32 (43.2%; 15.3-76.3)	114;46 (40.4%; 31.1-50.3)	2;1
Eastern	*Acinetobacter baumannii*	Fluoroquinolones	Others	68;37 (54.4%; 46.6-62)	92;51 (55.4%; 34.2-74.8)	95;65 (68.4%; 58.2-77.1)	1;1
Eastern	*Citrobacter*	Aminoglycosides	Blood/CSF	14;5	30;18 (60%; 53-66.6)	45;20 (44.4%; 30-59.9)	10;2
Eastern	*Citrobacter*	Aminoglycosides	Others	135;56 (41.5%; 36.8-46.4)	218;86 (39.4%; 34.4-44.7)	276;110 (39.9%; 33.4-46.7)	43;17 (39.5%; 26.4-54.5)
Eastern	*Citrobacter*	Beta-lactam combinations (Antipseudomonal)	Blood/CSF	5;3	1;0	4;0	5;1
Eastern	*Citrobacter*	Beta-lactam combinations (Antipseudomonal)	Others	25;8	37;16 (43.2%; 28.6-59.2)	46;11 (23.9%; 6.9-57.1)	28;7
Eastern	*Citrobacter*	Carbapenems	Blood/CSF	9;1	19;2	13;1	6;0
Eastern	*Citrobacter*	Carbapenems	Others	47;4 (8.5%; 2.1-28.6)	67;8 (11.9%; 7.4-18.7)	127;15 (11.8%; 4.4-28.1)	14;0
Eastern	*Citrobacter*	Cephalosporins (3rd generation)	Blood/CSF	17;11	29;21	44;29 (65.9%; 41.6-84)	12;4
Eastern	*Citrobacter*	Cephalosporins (3rd generation)	Others	137;71 (51.8%; 35.3-68)	198;113 (57.1%; 38.8-73.6)	263;140 (53.2%; 46.7-59.7)	40;23 (57.5%; 42.2-71.5)
Eastern	*Citrobacter*	Cephalosporins (4th generation)	Blood/CSF	3;1	1;1	1;1	8;3
Eastern	*Citrobacter*	Cephalosporins (4th generation)	Others	26;10	36;13 (36.1%; 27.6-45.6)	42;14 (33.3%; 22.8-45.8)	29;14
Eastern	*Citrobacter*	Fluoroquinolones	Blood/CSF	17;6	33;12 (36.4%; 23.3-51.8)	51;21 (41.2%; 28.3-55.4)	21;6
Eastern	*Citrobacter*	Fluoroquinolones	Others	209;81 (38.8%; 37.7-39.8)	305;125 (41%; 35.9-46.3)	404;168 (41.6%; 29.5-54.8)	131;61 (46.6%; 38.3-55.1)
Eastern	*Enterobacter*	Aminoglycosides	Blood/CSF	63;39 (61.9%; 33.6-83.9)	129;74 (57.4%; 30.2-80.7)	175;80 (45.7%; 37.5-54.2)	29;13
Eastern	*Enterobacter*	Aminoglycosides	Others	208;94 (45.2%; 37.7-52.9)	429;187 (43.6%; 28.2-60.3)	530;230 (43.4%; 35.3-51.9)	85;29 (34.1%; 9.2-72.5)
Eastern	*Enterobacter*	Beta-lactam combinations (Antipseudomonal)	Blood/CSF	6;2	8;4	37;6 (16.2%; 8.5-28.9)	16;2

Africa GBD# Region	Pathogen	Antimicrobial agent/class	Specimen source	2016 N; R(%R; 95%CI)	2017 N; R(%R; 95%CI)	2018 N; R(%R; 95%CI)	2019 N; R(%R; 95%CI)
Eastern	*Enterobacter*	Beta-lactam combinations (Antipseudomonal)	Others	53;25 (47.2%; 13.8-83.3)	125;48 (38.4%; 18-63.8)	141;40 (28.4%; 13.4-50.3)	48;9 (18.8%; 10.1-32.3)
Eastern	*Enterobacter*	Carbapenems	Blood/CSF	15;1	52;4 (7.7%; 2.3-22.7)	64;7 (10.9%; 3.7-27.9)	6;0
Eastern	*Enterobacter*	Carbapenems	Others	120;15 (12.5%; 2.7-42.7)	255;68 (26.7%; 13.1-46.6)	223;40 (17.9%; 8.9-32.9)	14;0
Eastern	*Enterobacter*	Cephalosporins (4th generation)	Blood/CSF	4;2	18;9	16;9	12;9
Eastern	*Enterobacter*	Cephalosporins (4th generation)	Others	80;38 (47.5%; 18.2-78.7)	126;65 (51.6%; 43.3-59.8)	72;46 (63.9%; 52.3-74.1)	36;17 (47.2%; 32-63)
Eastern	*Enterobacter*	Fluoroquinolones	Blood/CSF	64;35 (54.7%; 25.3-81.1)	110;50 (45.5%; 20.3-73.1)	174;86 (49.4%; 38.3-60.6)	42;17 (40.5%; 27.1-55.5)
Eastern	*Enterobacter*	Fluoroquinolones	Others	427;193 (45.2%; 43.3-47.1)	701;341 (48.6%; 46-51.3)	919;478 (52%; 45.1-58.9)	195;93 (47.7%; 40.8-54.7)
Eastern	*Enterococcus*	Aminopenicillins	Blood/CSF	133;81 (60.9%; 50.3-70.6)	111;59 (53.2%; 29.8-75.2)	101;59 (58.4%; 38.4-76)	1;0
Eastern	*Enterococcus*	Aminopenicillins	Others	306;55 (18%; 6.2-42.3)	544;52 (9.6%; 3.1-26)	454;23 (5.1%; 0.9-24.8)	10;8
Eastern	*Enterococcus faecalis*	Fluoroquinolones	Blood/CSF	66;29 (43.9%; 25.5-64.2)	37;18 (48.6%; 36.5-61)	42;22 (52.4%; 41.8-62.8)	1;0
Eastern	*Enterococcus faecalis*	Fluoroquinolones	Others	319;120 (37.6%; 17.4-63.3)	604;186 (30.8%; 23.2-39.6)	564;183 (32.4%; 20.1-47.8)	12;10
Eastern	*Enterococcus faecalis*	Vancomycin	Blood/CSF	73;3 (4.1%; 3.1-5.5)	42;1 (2.4%; 0.2-20.8)	56;6 (10.7%; 6.9-16.3)	1;0
Eastern	*Enterococcus faecalis*	Vancomycin	Others	275;14 (5.1%; 0.4-41.3)	462;11 (2.4%; 0.4-12.2)	460;26 (5.7%; 2.6-11.8)	11;0
Eastern	*Enterococcus faecium*	Fluoroquinolones	Blood/CSF	45;37 (82.2%; 16.2-99.1)	29;27	6;5	-
Eastern	*Enterococcus faecium*	Fluoroquinolones	Others	15;8	12;9	9;5	-
Eastern	*Enterococcus faecium*	Vancomycin	Blood/CSF	44;1 (2.3%; 1.3-4)	25;0	7;0	-
Eastern	*Enterococcus faecium*	Vancomycin	Others	16;0	9;0	8;0	-
Eastern	*Escherichia coli*	Aminoglycosides	Blood/CSF	280;98 (35%; 24.5-47.2)	401;149 (37.2%; 25.1-51.1)	498;181 (36.3%; 29-44.3)	86;38 (44.2%; 34.2-54.7)
Eastern	*Escherichia coli*	Aminoglycosides	Others	2936;900 (30.7%; 27.3-34.2)	3579;1150 (32.1%; 23.8-41.8)	3869;1188 (30.7%; 24.3-38)	241;134 (55.6%; 42.6-67.9)
Eastern	*Escherichia coli*	Aminopenicillins	Blood/CSF	251;227 (90.4%; 78.1-96.2)	358;293 (81.8%; 50-95.3)	411;349 (84.9%; 68.2-93.7)	103;90 (87.4%; 79.4-92.6)
Eastern	*Escherichia coli*	Aminopenicillins	Others	2565;2197 (85.7%; 81.3-89.1)	3282;2691 (82%; 57-94)	3473;2737 (78.8%; 59.1-90.5)	461;423 (91.8%; 89.7-93.4)
Eastern	*Escherichia coli*	Carbapenems	Blood/CSF	112;3 (2.7%; 1.5-4.7)	168;9 (5.4%; 3.7-7.6)	184;20 (10.9%; 4.3-24.9)	31;1 (3.2%; -0.7-17.8)

Africa GBD[#] Region	Pathogen	Antimicrobial agent/class	Specimen source	2016 N; R(%R; 95%CI)	2017 N; R(%R; 95%CI)	2018 N; R(%R; 95%CI)	2019 N; R(%R; 95%CI)
Eastern	*Escherichia coli*	Carbapenems	Others	1675;49 (2.9%; 0.8-9.9)	2503;189 (7.6%; 1.3-33.6)	2588;124 (4.8%; 1-20.1)	61;4 (6.6%; 2.2-16.3)
Eastern	*Escherichia coli*	Cephalosporins (3rd generation)	Blood/CSF	289;147 (50.9%; 27.2-74.1)	417;217 (52%; 35.4-68.3)	477;268 (56.2%; 33.5-76.6)	90;59 (65.6%; 55.2-74.5)
Eastern	*Escherichia coli*	Cephalosporins (3rd generation)	Others	2723;1294 (47.5%; 42.9-52.2)	3548;1926 (54.3%; 50.5-58)	3727;1985 (53.3%; 47.5-59)	254;183 (72%; 70.9-73.2)
Eastern	*Escherichia coli*	Fluoroquinolones	Blood/CSF	270;114 (42.2%; 26.4-59.8)	340;173 (50.9%; 35.9-65.7)	454;228 (50.2%; 33.7-66.7)	135;64 (47.4%; 39.2-55.8)
Eastern	*Escherichia coli*	Fluoroquinolones	Others	3310;1755 (53%; 44-61.8)	3803;2139 (56.2%; 50.2-62.2)	4016;2202 (54.8%; 50.6-59)	655;361 (55.1%; 50.4-59.7)
Eastern	*Escherichia coli*	Trimethoprim/Sulfamethoxazole	Blood/CSF	87;79 (90.8%; 87-93.6)	118;102 (86.4%; 82.1-89.9)	168;143 (85.1%; 79.1-89.6)	14;13
Eastern	*Escherichia coli*	Trimethoprim/Sulfamethoxazole	Others	1776;1532 (86.3%; 84.8-87.6)	2432;2098 (86.3%; 81.5-90)	2141;1725 (80.6%; 76.5-84.1)	71;61 (85.9%; 75.7-92.3)
Eastern	*Haemophilus influenzae*	Aminopenicillins	Blood/CSF	14;6	6;4	7;6	1;0
Eastern	*Haemophilus influenzae*	Aminopenicillins	Others	17;6	4;0	34;10 (29.4%; 17.9-44.3)	-
Eastern	*Haemophilus influenzae*	Cephalosporins (3rd generation)	Blood/CSF	13;0	8;1	6;0	1;0
Eastern	*Haemophilus influenzae*	Cephalosporins (3rd generation)	Others	8;1	4;0	17;6	-
Eastern	*Klebsiella pneumoniae*	Aminoglycosides	Blood/CSF	437;348 (79.6%; 61.4-90.6)	458;357 (77.9%; 58.4-89.9)	423;296 (70%; 43.2-87.7)	63;36 (57.1%; 44.9-68.6)
Eastern	*Klebsiella pneumoniae*	Aminoglycosides	Others	754;313 (41.5%; 27.1-57.6)	909;421 (46.3%; 31.6-61.7)	775;374 (48.3%; 33.1-63.8)	216;137 (63.4%; 60.2-66.5)
Eastern	*Klebsiella pneumoniae*	Carbapenems	Blood/CSF	305;10 (3.3%; 0.7-14.5)	233;10 (4.3%; 0.6-26.5)	166;14 (8.4%; 4.2-16.2)	29;0
Eastern	*Klebsiella pneumoniae*	Carbapenems	Others	459;67 (14.6%; 2.8-50.7)	561;67 (11.9%; 2.5-41.7)	547;40 (7.3%; 1.9-24.3)	62;0 (0%; 0-0)
Eastern	*Klebsiella pneumoniae*	Cephalosporins (3rd generation)	Blood/CSF	459;420 (91.5%; 79.4-96.8)	466;416 (89.3%; 82.5-93.6)	427;365 (85.5%; 75.3-91.9)	62;54 (87.1%; 76.2-93.5)
Eastern	*Klebsiella pneumoniae*	Cephalosporins (3rd generation)	Others	796;525 (66%; 54-76.2)	915;602 (65.8%; 62.5-69)	780;546 (70%; 61.6-77.2)	227;190 (83.7%; 79.7-87)
Eastern	*Klebsiella pneumoniae*	Fluoroquinolones	Blood/CSF	425;217 (51.1%; 39.8-62.3)	426;172 (40.4%; 15.2-71.9)	409;170 (41.6%; 28.4-56)	82;44 (53.7%; 42.9-64)
Eastern	*Klebsiella pneumoniae*	Fluoroquinolones	Others	789;413 (52.3%; 43.4-61.1)	1055;562 (53.3%; 47.5-59)	772;372 (48.2%; 34.8-61.8)	385;216 (56.1%; 51.1-61)
Eastern	*Morganella*	Cephalosporins (3rd generation)	Blood/CSF	1;0	1;1	5;2	-
Eastern	*Morganella*	Cephalosporins (3rd generation)	Others	41;27 (65.9%; 44.3-82.4)	45;30 (66.7%; 53.2-77.8)	53;22 (41.5%; 34.6-48.8)	3;1
Eastern	*Morganella*	Cephalosporins (4th generation)	Others	5;3	4;1	3;2	-

Africa GBD# Region	Pathogen	Antimicrobial agent/class	Specimen source	2016 N; R(%R; 95%CI)	2017 N; R(%R; 95%CI)	2018 N; R(%R; 95%CI)	2019 N; R(%R; 95%CI)
Eastern	*Morganella*	Fluoroquinolones	Blood/CSF	1;0	-	5;2	-
Eastern	*Morganella*	Fluoroquinolones	Others	34;18 (52.9%; 38-67.4)	35;17 (48.6%; 30.9-66.6)	57;23 (40.4%; 29.7-51.9)	4;2
Eastern	*Neisseria gonorrhoeae*	Cephalosporins (3rd generation)	Blood/CSF	-	3;0	5;0	-
Eastern	*Neisseria gonorrhoeae*	Cephalosporins (3rd generation)	Others	32;4 (12.5%; 4.9-28.3)	88;4 (4.5%; 0.3-46)	118;6 (5.1%; 1-22.6)	-
Eastern	*Neisseria gonorrhoeae*	Fluoroquinolones	Blood/CSF	1;0	4;0	5;1	-
Eastern	*Neisseria gonorrhoeae*	Fluoroquinolones	Others	34;15 (44.1%; 19.4-72.1)	74;38 (51.4%; 35.9-66.5)	115;47 (40.9%; 26.6-56.9)	1;1
Eastern	*Neisseria gonorrhoeae*	Macrolides	Blood/CSF	-	1;0	-	-
Eastern	*Neisseria gonorrhoeae*	Macrolides	Others	24;11	14;8	29;20	-
Eastern	*Neisseria gonorrhoeae*	Quinolones	Others	6;5	2;2	6;5	-
Eastern	*Neisseria gonorrhoeae*	Tetracyclines	Blood/CSF	-	1;1	4;4	-
Eastern	*Neisseria gonorrhoeae*	Tetracyclines	Others	13;8	69;65 (94.2%; 56.1-99.5)	86;71 (82.6%; 60.1-93.7)	-
Eastern	*Non-typhoidal Salmonella*	Fluoroquinolones	Blood/CSF	66;9 (13.6%; 0.7-77.2)	68;6 (8.8%; 1.2-44.4)	62;13 (21%; 4-62.9)	5;0
Eastern	*Non-typhoidal Salmonella*	Fluoroquinolones	Others	70;28 (40%; 18.5-66.2)	72;32 (44.4%; 20.4-71.4)	48;17 (35.4%; 10.4-72.1)	22;6
Eastern	*Proteus*	Aminoglycosides	Blood/CSF	22;8	41;12 (29.3%; 15.4-48.4)	69;25 (36.2%; 21.4-54.2)	41;20 (48.8%; 34.3-63.5)
Eastern	*Proteus*	Aminoglycosides	Others	574;155 (27%; 16.8-40.4)	648;231 (35.6%; 25.7-47)	766;289 (37.7%; 28.3-48.2)	201;91 (45.3%; 43.3-47.2)
Eastern	*Proteus*	Aminopenicillins	Blood/CSF	25;22	38;23 (60.5%; 43.1-75.6)	47;30 (63.8%; 46.7-78)	41;35 (85.4%; 71.1-93.4)
Eastern	*Proteus*	Aminopenicillins	Others	492;367 (74.6%; 67.1-80.9)	621;423 (68.1%; 52.3-80.6)	652;442 (67.8%; 55-78.4)	214;176 (82.2%; 80.8-83.6)
Eastern	*Proteus*	Cephalosporins (3rd generation)	Blood/CSF	22;8	40;17 (42.5%; 27.6-58.9)	58;23 (39.7%; 29.4-50.9)	43;30 (69.8%; 54.8-81.4)
Eastern	*Proteus*	Cephalosporins (3rd generation)	Others	542;203 (37.5%; 29.6-46.1)	785;315 (40.1%; 34.3-46.3)	760;370 (48.7%; 37.9-59.6)	215;162 (75.3%; 73.3-77.3)
Eastern	*Proteus*	Cephalosporins (4th generation)	Blood/CSF	2;1	6;2	3;1	15;13
Eastern	*Proteus*	Cephalosporins (4th generation)	Others	60;30 (50%; 34.2-65.8)	83;23 (27.7%; 10.4-56)	49;17 (34.7%; 9.1-73.7)	81;58 (71.6%; 60.9-80.3)
Eastern	*Proteus*	Fluoroquinolones	Blood/CSF	24;11	34;10 (29.4%; 21.2-39.3)	48;15 (31.2%; 20.3-44.8)	48;20 (41.7%; 28.9-55.7)

Africa GBD# Region	Pathogen	Antimicrobial agent/class	Specimen source	2016 N; R(%R; 95%CI)	2017 N; R(%R; 95%CI)	2018 N; R(%R; 95%CI)	2019 N; R(%R; 95%CI)
Eastern	*Proteus*	Fluoroquinolones	Others	627;218 (34.8%; 21.3-51.2)	771;264 (34.2%; 26.5-42.9)	791;302 (38.2%; 28.6-48.7)	247;87 (35.2%; 28.4-42.8)
Eastern	*Pseudomonas aeruginosa*	Aminoglycosides	Blood/CSF	50;11 (22%; 12.1-36.6)	103;24 (23.3%; 10.3-44.7)	103;18 (17.5%; 9.1-30.9)	45;15 (33.3%; 21.4-48)
Eastern	*Pseudomonas aeruginosa*	Aminoglycosides	Others	443;131 (29.6%; 21-39.9)	608;191 (31.4%; 17.6-49.5)	736;179 (24.3%; 15.7-35.6)	292;90 (30.8%; 25.8-36.4)
Eastern	*Pseudomonas aeruginosa*	Beta-lactam combinations (Antipseudomonal)	Blood/CSF	17;3	18;4	35;10 (28.6%; 7.8-65.4)	17;1
Eastern	*Pseudomonas aeruginosa*	Beta-lactam combinations (Antipseudomonal)	Others	123;19 (15.4%; 8.3-27)	219;41 (18.7%; 11-30)	338;41 (12.1%; 7.5-19.1)	110;15 (13.6%; 8.4-21.5)
Eastern	*Pseudomonas aeruginosa*	Carbapenems	Blood/CSF	30;2 (6.7%; 1.5-24.6)	46;11 (23.9%; 20-28.4)	42;13 (31%; 21.2-42.8)	13;0
Eastern	*Pseudomonas aeruginosa*	Carbapenems	Others	218;39 (17.9%; 5.7-44.2)	350;80 (22.9%; 16-31.5)	423;90 (21.3%; 13.2-32.5)	40;1 (2.5%; -0.6-14.3)
Eastern	*Pseudomonas aeruginosa*	Cephalosporins (3rd generation)	Blood/CSF	38;21 (55.3%; 45-65.1)	69;35 (50.7%; 33.8-67.5)	60;36 (60%; 50.9-68.4)	41;25 (61%; 45.7-74.3)
Eastern	*Pseudomonas aeruginosa*	Cephalosporins (3rd generation)	Others	345;177 (51.3%; 37.6-64.8)	563;270 (48%; 29.9-66.6)	693;368 (53.1%; 38.4-67.3)	265;160 (60.4%; 54.4-66.1)
Eastern	*Pseudomonas aeruginosa*	Cephalosporins (4th generation)	Blood/CSF	11;3	12;4	9;4	10;6
Eastern	*Pseudomonas aeruginosa*	Cephalosporins (4th generation)	Others	124;41 (33.1%; 20-49.4)	199;55 (27.6%; 10.5-55.4)	139;43 (30.9%; 6.1-75.4)	64;25 (39.1%; 28.1-51.3)
Eastern	*Pseudomonas aeruginosa*	Fluoroquinolones	Blood/CSF	51;8 (15.7%; 3.7-47.6)	85;15 (17.6%; 6.6-39.4)	96;14 (14.6%; 6.3-30.4)	48;6 (12.5%; 5.6-25.2)
Eastern	*Pseudomonas aeruginosa*	Fluoroquinolones	Others	393;94 (23.9%; 19.9-28.5)	509;138 (27.1%; 23-31.7)	656;159 (24.2%; 16.7-33.8)	342;65 (19%; 15.2-23.5)
Eastern	*Salmonella Paratyphi*	Fluoroquinolones	Blood/CSF	1;0	2;0	-	1;0
Eastern	*Salmonella Paratyphi*	Fluoroquinolones	Others	-	3;1	2;1	1;0
Eastern	*Salmonella Typhi*	Fluoroquinolones	Blood/CSF	454;12 (2.6%; 0.2-30.2)	428;17 (4%; 0.2-42.5)	448;16 (3.6%; 0.6-18.9)	26;1
Eastern	*Salmonella Typhi*	Fluoroquinolones	Others	19;4	24;7	20;6	5;0
Eastern	*Serratia*	Aminoglycosides	Blood/CSF	7;2	37;6 (16.2%; 1.1-76.9)	29;8	1;1
Eastern	*Serratia*	Aminoglycosides	Others	69;23 (33.3%; 15.6-57.6)	101;40 (39.6%; 21.1-61.6)	129;37 (28.7%; 24.5-33.2)	3;1
Eastern	*Serratia*	Carbapenems	Blood/CSF	4;2	8;2	7;2	-
Eastern	*Serratia*	Carbapenems	Others	33;7 (21.2%; 1.5-82.6)	62;9 (14.5%; 2.4-54.2)	56;12 (21.4%; 3.4-67.9)	1;0

Africa GBD# Region	Pathogen	Antimicrobial agent/class	Specimen source	2016 N; R(%R; 95%CI)	2017 N; R(%R; 95%CI)	2018 N; R(%R; 95%CI)	2019 N; R(%R; 95%CI)
Eastern	*Serratia*	Cephalosporins (3rd generation)	Blood/CSF	10;5	37;13 (35.1%; 10.1-72.4)	41;12 (29.3%; 6.2-72.3)	1;1
Eastern	*Serratia*	Cephalosporins (3rd generation)	Others	70;44 (62.9%; 55.5-69.7)	101;57 (56.4%; 43.6-68.4)	147;73 (49.7%; 42.5-56.8)	4;2
Eastern	*Serratia*	Cephalosporins (4th generation)	Blood/CSF	5;0	24;2	19;1	-
Eastern	*Serratia*	Cephalosporins (4th generation)	Others	19;8	37;16 (43.2%; 12.3-80.5)	36;17 (47.2%; 38.4-56.2)	-
Eastern	*Serratia*	Fluoroquinolones	Blood/CSF	8;2	16;9	13;4	1;1
Eastern	*Serratia*	Fluoroquinolones	Others	58;16 (27.6%; 24.1-31.3)	101;38 (37.6%; 33.8-41.6)	121;39 (32.2%; 24.6-40.9)	2;0
Eastern	*Shigella*	Fluoroquinolones	Blood/CSF	2;0	9;1	6;1	-
Eastern	*Shigella*	Fluoroquinolones	Others	91;9 (9.9%; 6.1-15.7)	71;15 (21.1%; 17.9-24.8)	95;17 (17.9%; 9.1-32.3)	2;1
Eastern	*Staphylococcus aureus*	Beta-lactam combinations (Antipseudomonal)	Blood/CSF	25;9	11;7	3;0	-
Eastern	*Staphylococcus aureus*	Beta-lactam combinations (Antipseudomonal)	Others	43;12 (27.9%; 22.9-33.5)	21;16	48;11 (22.9%; 9.6-45.5)	1;0
Eastern	*Staphylococcus aureus*	Fluoroquinolones	Blood/CSF	219;83 (37.9%; 29.2-47.5)	457;190 (41.6%; 38-45.2)	453;170 (37.5%; 32.9-42.4)	177;59 (33.3%; 26.8-40.6)
Eastern	*Staphylococcus aureus*	Fluoroquinolones	Others	1250;320 (25.6%; 22.9-28.5)	1337;407 (30.4%; 25.7-35.7)	1928;528 (27.4%; 16.9-41.1)	512;184 (35.9%; 32.8-39.2)
Eastern	*Staphylococcus aureus*	Macrolides	Blood/CSF	543;291 (53.6%; 38.1-68.4)	780;442 (56.7%; 43.1-69.3)	647;369 (57%; 32.3-78.7)	90;38 (42.2%; 32.6-52.5)
Eastern	*Staphylococcus aureus*	Macrolides	Others	1642;826 (50.3%; 31.4-69.1)	1706;892 (52.3%; 41.7-62.7)	2004;1050 (52.4%; 46.5-58.2)	463;205 (44.3%; 43.2-45.3)
Eastern	*Staphylococcus aureus*	Methicillin	Blood/CSF	301;68 (22.6%; 13.2-35.8)	367;133 (36.2%; 18.6-58.5)	449;192 (42.8%; 24.9-62.8)	176;100 (56.8%; 49.4-63.9)
Eastern	*Staphylococcus aureus*	Methicillin	Others	1210;390 (32.2%; 19.8-47.8)	1070;381 (35.6%; 18.7-57)	1159;460 (39.7%; 25.7-55.6)	520;292 (56.2%; 51.8-60.4)
Eastern	*Staphylococcus aureus*	Trimethoprim/Sulfamethoxazole	Blood/CSF	145;93 (64.1%; 48.2-77.5)	243;194 (79.8%; 60.2-91.2)	235;164 (69.8%; 56.2-80.6)	37;27 (73%; 56.8-84.7)
Eastern	*Staphylococcus aureus*	Trimethoprim/Sulfamethoxazole	Others	1054;767 (72.8%; 69.8-75.6)	1008;715 (70.9%; 62.6-78)	1312;966 (73.6%; 69.2-77.6)	132;92 (69.7%; 61.4-76.9)
Eastern	*Streptococcus agalactiae*	Fluoroquinolones	Blood/CSF	1;0	2;0	2;0	-
Eastern	*Streptococcus agalactiae*	Fluoroquinolones	Others	36;9 (25%; 19-32.2)	34;7 (20.6%; 14-29.3)	23;3	-
Eastern	*Streptococcus agalactiae*	Macrolides	Blood/CSF	3;0	1;0	4;2	-

Africa GBD# Region	Pathogen	Antimicrobial agent/class	Specimen source	2016 N; R(%R; 95%CI)	2017 N; R(%R; 95%CI)	2018 N; R(%R; 95%CI)	2019 N; R(%R; 95%CI)
Eastern	*Streptococcus agalactiae*	Macrolides	Others	40;10 (25%; 9.4-51.6)	40;15 (37.5%; 18-62.2)	21;3	-
Eastern	*Streptococcus agalactiae*	Penicillins	Blood/CSF	3;0	1;0	2;0	-
Eastern	*Streptococcus agalactiae*	Penicillins	Others	27;4	30;12 (40%; 9.6-80.7)	17;2	-
Eastern	*Streptococcus pneumoniae*	Carbapenems	Blood/CSF	2;0	3;0	2;0	-
Eastern	*Streptococcus pneumoniae*	Carbapenems	Others	24;0	6;0	43;3 (7%; 5.3-9.1)	-
Eastern	*Streptococcus pneumoniae*	Cephalosporins (3rd generation)	Blood/CSF	64;4 (6.2%; 1.2-27)	57;1 (1.8%; 0.2-15.8)	57;6 (10.5%; 1.2-52.9)	3;0
Eastern	*Streptococcus pneumoniae*	Cephalosporins (3rd generation)	Others	59;1 (1.7%; 0.1-30.8)	39;5 (12.8%; 5.2-28.4)	77;12 (15.6%; 4.7-40.8)	3;0
Eastern	*Streptococcus pneumoniae*	Fluoroquinolones	Blood/CSF	16;1	15;3	15;1	-
Eastern	*Streptococcus pneumoniae*	Fluoroquinolones	Others	47;12 (25.5%; 15.7-38.7)	39;12 (30.8%; 15.8-51.3)	79;7 (8.9%; 4.3-17.4)	-
Eastern	*Streptococcus pneumoniae*	Macrolides	Blood/CSF	70;13 (18.6%; 2.5-67.2)	50;15 (30%; 17.1-47.1)	65;21 (32.3%; 22.8-43.6)	-
Eastern	*Streptococcus pneumoniae*	Macrolides	Others	81;48 (59.3%; 29.4-83.6)	57;24 (42.1%; 36.9-47.5)	111;70 (63.1%; 36.6-83.5)	1;0
Eastern	*Streptococcus pneumoniae*	Penicillins	Blood/CSF	32;14 (43.8%; 26.6-62.5)	34;7 (20.6%; 10.4-36.6)	27;12	3;1
Eastern	*Streptococcus pneumoniae*	Penicillins	Others	72;14 (19.4%; 8.8-37.6)	44;22 (50%; 26.4-73.6)	90;40 (44.4%; 39.3-49.7)	5;1
Eastern	*Streptococcus pneumoniae*	Trimethoprim/Sulfamethoxazole	Blood/CSF	13;11	16;13	15;12	-
Eastern	*Streptococcus pneumoniae*	Trimethoprim/Sulfamethoxazole	Others	50;45 (90%; 65.5-97.7)	28;22	56;49 (87.5%; 65.8-96.2)	-
Eastern	*Streptococcus pyogenes*	Macrolides	Blood/CSF	7;3	14;4	14;11	-
Eastern	*Streptococcus pyogenes*	Macrolides	Others	85;24 (28.2%; 15-46.8)	181;57 (31.5%; 24.3-39.7)	90;34 (37.8%; 24.5-53.1)	-
Southern	*Acinetobacter baumannii*	Aminoglycosides	Blood/CSF	8;5	9;3	22;11	-
Southern	*Acinetobacter baumannii*	Aminoglycosides	Others	2;0	6;2	9;5	-
Southern	*Acinetobacter baumannii*	Beta-lactam combinations (Antipseudomonal)	Others	1;0	1;0	-	-

Africa GBD# Region	Pathogen	Antimicrobial agent/class	Specimen source	2016 N; R(%R; 95%CI)	2017 N; R(%R; 95%CI)	2018 N; R(%R; 95%CI)	2019 N; R(%R; 95%CI)
Southern	*Acinetobacter baumannii*	Carbapenems	Blood/CSF	5;1	5;1	11;4	-
Southern	*Acinetobacter baumannii*	Carbapenems	Others	2;0	1;0	7;2	-
Southern	*Acinetobacter baumannii*	Cephalosporins (3rd generation)	Blood/CSF	7;5	8;6	20;15	-
Southern	*Acinetobacter baumannii*	Cephalosporins (3rd generation)	Others	4;2	17;8	8;6	-
Southern	*Acinetobacter baumannii*	Cephalosporins (4th generation)	Others	2;0	1;0	1;1	-
Southern	*Acinetobacter baumannii*	Fluoroquinolones	Blood/CSF	8;3	12;5	21;6	-
Southern	*Acinetobacter baumannii*	Fluoroquinolones	Others	3;1	21;6	9;5	-
Southern	*Citrobacter*	Aminoglycosides	Blood/CSF	1;0	7;0	1;0	-
Southern	*Citrobacter*	Aminoglycosides	Others	3;0	18;2	17;5	-
Southern	*Citrobacter*	Beta-lactam combinations (Antipseudomonal)	Others	-	2;0	-	-
Southern	*Citrobacter*	Carbapenems	Blood/CSF	1;0	-	-	-
Southern	*Citrobacter*	Carbapenems	Others	1;0	5;0	6;1	-
Southern	*Citrobacter*	Cephalosporins (3rd generation)	Blood/CSF	1;0	6;1	1;1	-
Southern	*Citrobacter*	Cephalosporins (3rd generation)	Others	3;0	18;5	17;10	-
Southern	*Citrobacter*	Cephalosporins (4th generation)	Blood/CSF	1;0	-	-	-
Southern	*Citrobacter*	Cephalosporins (4th generation)	Others	1;0	2;0	1;1	-
Southern	*Citrobacter*	Fluoroquinolones	Blood/CSF	1;0	7;0	1;1	-
Southern	*Citrobacter*	Fluoroquinolones	Others	4;0	16;2	13;5	-
Southern	*Enterobacter*	Aminoglycosides	Blood/CSF	3;1	9;2	5;2	-
Southern	*Enterobacter*	Aminoglycosides	Others	7;1	24;2	11;5	-

Africa GBD# Region	Pathogen	Antimicrobial agent/class	Specimen source	2016 N; R(%R; 95%CI)	2017 N; R(%R; 95%CI)	2018 N; R(%R; 95%CI)	2019 N; R(%R; 95%CI)
Southern	*Enterobacter*	Beta-lactam combinations (Antipseudomonal)	Blood/CSF	1;0	-	1;1	-
Southern	*Enterobacter*	Beta-lactam combinations (Antipseudomonal)	Others	1;1	4;0	4;3	-
Southern	*Enterobacter*	Carbapenems	Blood/CSF	2;1	3;1	3;0	-
Southern	*Enterobacter*	Carbapenems	Others	4;0	4;0	9;0	-
Southern	*Enterobacter*	Cephalosporins (4th generation)	Blood/CSF	1;0	-	1;1	-
Southern	*Enterobacter*	Cephalosporins (4th generation)	Others	3;1	4;0	6;5	-
Southern	*Enterobacter*	Fluoroquinolones	Blood/CSF	2;1	8;1	5;1	-
Southern	*Enterobacter*	Fluoroquinolones	Others	6;0	25;8	11;6	-
Southern	*Enterococcus*	Aminopenicillins	Blood/CSF	6;2	14;7	9;4	-
Southern	*Enterococcus*	Aminopenicillins	Others	22;7	54;22 (40.7%; 31.7-50.4)	16;5	-
Southern	*Enterococcus faecalis*	Fluoroquinolones	Blood/CSF	4;0	10;2	8;5	-
Southern	*Enterococcus faecalis*	Fluoroquinolones	Others	16;2	53;5 (9.4%; 1.2-46.5)	14;4	-
Southern	*Enterococcus faecalis*	Vancomycin	Blood/CSF	3;0	11;0	7;0	-
Southern	*Enterococcus faecalis*	Vancomycin	Others	17;0	50;6 (12%; 0.1-96.6)	11;1	-
Southern	*Enterococcus faecium*	Fluoroquinolones	Blood/CSF	3;2	3;3	-	-
Southern	*Enterococcus faecium*	Fluoroquinolones	Others	6;5	10;5	1;1	-
Southern	*Enterococcus faecium*	Vancomycin	Blood/CSF	2;0	3;0	2;0	-
Southern	*Enterococcus faecium*	Vancomycin	Others	4;1	9;2	1;0	-
Southern	*Escherichia coli*	Aminoglycosides	Blood/CSF	10;1	29;3	18;4	-
Southern	*Escherichia coli*	Aminoglycosides	Others	625;132 (21.1%; 4.1-62.5)	701;178 (25.4%; 4.6-70.6)	577;165 (28.6%; 9.2-61.3)	-

Africa GBD# Region	Pathogen	Antimicrobial agent/class	Specimen source	2016 N; R(%R; 95%CI)	2017 N; R(%R; 95%CI)	2018 N; R(%R; 95%CI)	2019 N; R(%R; 95%CI)
Southern	*Escherichia coli*	Aminopenicillins	Blood/CSF	14;11	30;23 (76.7%; 63-86.4)	11;5	-
Southern	*Escherichia coli*	Aminopenicillins	Others	575;460 (80%; 71.3-86.6)	646;529 (81.9%; 77.4-85.7)	521;406 (77.9%; 74.3-81.2)	-
Southern	*Escherichia coli*	Carbapenems	Blood/CSF	7;0	6;0	13;1	-
Southern	*Escherichia coli*	Carbapenems	Others	371;0 (0%; 0-0)	315;7 (2.2%; 2-2.4)	339;13 (3.8%; 0.3-38.6)	-
Southern	*Escherichia coli*	Cephalosporins (3rd generation)	Blood/CSF	15;7	31;13 (41.9%; 26.6-59)	20;8	-
Southern	*Escherichia coli*	Cephalosporins (3rd generation)	Others	440;94 (21.4%; 7-49.4)	609;238 (39.1%; 24.7-55.6)	440;106 (24.1%; 11-45)	-
Southern	*Escherichia coli*	Fluoroquinolones	Blood/CSF	14;5	31;10 (32.3%; 30.5-34)	21;11	-
Southern	*Escherichia coli*	Fluoroquinolones	Others	494;148 (30%; 25.8-34.5)	638;224 (35.1%; 33.1-37.2)	460;143 (31.1%; 22.8-40.8)	-
Southern	*Escherichia coli*	Trimethoprim/Sulfamethoxazole	Blood/CSF	2;2	4;3	11;9	-
Southern	*Escherichia coli*	Trimethoprim/Sulfamethoxazole	Others	433;321 (74.1%; 54.2-87.4)	371;279 (75.2%; 54.3-88.6)	409;299 (73.1%; 58.1-84.2)	-
Southern	*Haemophilus influenzae*	Aminopenicillins	Others	-	1;1	1;0	-
Southern	*Haemophilus influenzae*	Cephalosporins (3rd generation)	Others	-	-	1;0	-
Southern	*Klebsiella pneumoniae*	Aminoglycosides	Blood/CSF	18;8	24;10	22;12	-
Southern	*Klebsiella pneumoniae*	Aminoglycosides	Others	41;9 (22%; 1.3-86)	76;7 (9.2%; 8.2-10.3)	27;10	-
Southern	*Klebsiella pneumoniae*	Carbapenems	Blood/CSF	19;0	13;0	16;0	-
Southern	*Klebsiella pneumoniae*	Carbapenems	Others	27;0	16;0	13;0	-
Southern	*Klebsiella pneumoniae*	Cephalosporins (3rd generation)	Blood/CSF	19;15	21;12	21;14	-
Southern	*Klebsiella pneumoniae*	Cephalosporins (3rd generation)	Others	34;22 (64.7%; 21.9-92.3)	79;44 (55.7%; 51.2-60.1)	29;12	-
Southern	*Klebsiella pneumoniae*	Fluoroquinolones	Blood/CSF	21;10	23;8	22;9	-
Southern	*Klebsiella pneumoniae*	Fluoroquinolones	Others	39;13 (33.3%; 28.1-39)	74;25 (33.8%; 30.1-37.7)	22;5	-

Africa GBD# Region	Pathogen	Antimicrobial agent/class	Specimen source	2016 N; R(%R; 95%CI)	2017 N; R(%R; 95%CI)	2018 N; R(%R; 95%CI)	2019 N; R(%R; 95%CI)
Southern	*Morganella*	Cephalosporins (3rd generation)	Blood/CSF	1;0	-	2;0	-
Southern	*Morganella*	Cephalosporins (3rd generation)	Others	1;0	8;4	3;3	-
Southern	*Morganella*	Cephalosporins (4th generation)	Others	-	1;0	1;0	-
Southern	*Morganella*	Fluoroquinolones	Blood/CSF	2;1	-	2;0	-
Southern	*Morganella*	Fluoroquinolones	Others	1;0	9;2	3;1	-
Southern	*Neisseria gonorrhoeae*	Cephalosporins (3rd generation)	Others	9;0	14;0	5;1	-
Southern	*Neisseria gonorrhoeae*	Fluoroquinolones	Others	9;1	14;7	5;3	-
Southern	*Neisseria gonorrhoeae*	Macrolides	Others	-	-	1;0	-
Southern	*Neisseria gonorrhoeae*	Quinolones	Others	-	-	1;1	-
Southern	*Neisseria gonorrhoeae*	Tetracyclines	Others	9;2	16;11	4;3	-
Southern	*Non-typhoidal Salmonella*	Fluoroquinolones	Blood/CSF	1;0	7;1	3;0	-
Southern	*Non-typhoidal Salmonella*	Fluoroquinolones	Others	13;3	22;4	6;4	-
Southern	*Proteus*	Aminoglycosides	Blood/CSF	10;1	8;1	4;2	-
Southern	*Proteus*	Aminoglycosides	Others	216;59 (27.3%; 6.3-67.8)	260;53 (20.4%; 3.7-63.3)	128;40 (31.2%; 10-65)	-
Southern	*Proteus*	Aminopenicillins	Blood/CSF	9;3	5;3	1;1	-
Southern	*Proteus*	Aminopenicillins	Others	195;140 (71.8%; 52.9-85.2)	221;152 (68.8%; 67.2-70.3)	86;59 (68.6%; 43.6-86.1)	-
Southern	*Proteus*	Cephalosporins (3rd generation)	Blood/CSF	9;2	6;1	3;0	-
Southern	*Proteus*	Cephalosporins (3rd generation)	Others	105;31 (29.5%; 10.1-60.9)	236;79 (33.5%; 24-44.5)	112;39 (34.8%; 23.6-48)	-
Southern	*Proteus*	Cephalosporins (4th generation)	Blood/CSF	4;0	-	1;0	-
Southern	*Proteus*	Cephalosporins (4th generation)	Others	22;3	26;3	13;2	-

Africa GBD# Region	Pathogen	Antimicrobial agent/class	Specimen source	2016 N; R(%R; 95%CI)	2017 N; R(%R; 95%CI)	2018 N; R(%R; 95%CI)	2019 N; R(%R; 95%CI)
Southern	*Proteus*	Fluoroquinolones	Blood/CSF	9;0	7;0	4;0	-
Southern	*Proteus*	Fluoroquinolones	Others	136;29 (21.3%; 6.8-50.2)	261;50 (19.2%; 4.6-54.1)	118;34 (28.8%; 15.4-47.4)	-
Southern	*Pseudomonas aeruginosa*	Aminoglycosides	Blood/CSF	6;0	8;1	6;0	-
Southern	*Pseudomonas aeruginosa*	Aminoglycosides	Others	129;29 (22.5%; 16.2-30.3)	186;69 (37.1%; 12.3-71.2)	178;63 (35.4%; 17.3-58.9)	1;0
Southern	*Pseudomonas aeruginosa*	Beta-lactam combinations (Antipseudomonal)	Others	9;0	10;1	12;0	-
Southern	*Pseudomonas aeruginosa*	Carbapenems	Blood/CSF	1;0	-	2;0	-
Southern	*Pseudomonas aeruginosa*	Carbapenems	Others	27;1	36;14 (38.9%; 4.6-89.3)	57;11 (19.3%; 2.8-66.3)	-
Southern	*Pseudomonas aeruginosa*	Cephalosporins (3rd generation)	Blood/CSF	5;2	5;3	4;1	-
Southern	*Pseudomonas aeruginosa*	Cephalosporins (3rd generation)	Others	99;32 (32.3%; 28.8-36.1)	154;108 (70.1%; 65.7-74.2)	141;72 (51.1%; 39.2-62.8)	-
Southern	*Pseudomonas aeruginosa*	Cephalosporins (4th generation)	Blood/CSF	1;0	-	-	-
Southern	*Pseudomonas aeruginosa*	Cephalosporins (4th generation)	Others	15;2	10;1	28;3	-
Southern	*Pseudomonas aeruginosa*	Fluoroquinolones	Blood/CSF	6;1	8;2	5;1	-
Southern	*Pseudomonas aeruginosa*	Fluoroquinolones	Others	140;35 (25%; 19.8-31.1)	173;29 (16.8%; 5-43.6)	173;40 (23.1%; 16.9-30.8)	1;0
Southern	*Salmonella Typhi*	Fluoroquinolones	Blood/CSF	18;5	26;3	-	-
Southern	*Salmonella Typhi*	Fluoroquinolones	Others	39;6 (15.4%; 7-30.2)	47;13 (27.7%; 0.5-96.9)	5;0	-
Southern	*Serratia*	Aminoglycosides	Blood/CSF	1;0	3;0	3;0	-
Southern	*Serratia*	Aminoglycosides	Others	8;2	19;4	30;3 (10%; 0.6-66.2)	-
Southern	*Serratia*	Carbapenems	Blood/CSF	-	-	3;1	-
Southern	*Serratia*	Carbapenems	Others	4;0	1;0	6;0	-
Southern	*Serratia*	Cephalosporins (3rd generation)	Blood/CSF	1;1	2;0	3;3	-

Africa GBD# Region	Pathogen	Antimicrobial agent/class	Specimen source	2016 N; R(%R; 95%CI)	2017 N; R(%R; 95%CI)	2018 N; R(%R; 95%CI)	2019 N; R(%R; 95%CI)
Southern	*Serratia*	Cephalosporins (3rd generation)	Others	8;3	21;11	30;17 (56.7%; 44.1-68.4)	-
Southern	*Serratia*	Cephalosporins (4th generation)	Blood/CSF	-	-	1;1	-
Southern	*Serratia*	Cephalosporins (4th generation)	Others	3;1	-	1;1	-
Southern	*Serratia*	Fluoroquinolones	Blood/CSF	1;1	2;1	4;0	-
Southern	*Serratia*	Fluoroquinolones	Others	7;3	20;6	30;9 (30%; 14.1-52.7)	-
Southern	*Shigella*	Fluoroquinolones	Blood/CSF	-	1;0	-	-
Southern	*Shigella*	Fluoroquinolones	Others	11;0	27;5	15;4	-
Southern	*Staphylococcus aureus*	Fluoroquinolones	Blood/CSF	63;15 (23.8%; 17.8-31.2)	97;19 (19.6%; 14.2-26.4)	92;21 (22.8%; 22.3-23.3)	-
Southern	*Staphylococcus aureus*	Fluoroquinolones	Others	273;76 (27.8%; 22.4-34.1)	319;58 (18.2%; 12.5-25.7)	366;87 (23.8%; 16.7-32.6)	-
Southern	*Staphylococcus aureus*	Macrolides	Blood/CSF	74;26 (35.1%; 11-70.3)	98;29 (29.6%; 22.9-37.3)	77;32 (41.6%; 39.9-43.2)	1;0
Southern	*Staphylococcus aureus*	Macrolides	Others	255;94 (36.9%; 30.7-43.5)	340;110 (32.4%; 24-42.1)	399;123 (30.8%; 24.2-38.3)	-
Southern	*Staphylococcus aureus*	Methicillin	Blood/CSF	70;21 (30%; 16-49)	81;36 (44.4%; 18.2-74.3)	98;52 (53.1%; 42.6-63.3)	-
Southern	*Staphylococcus aureus*	Methicillin	Others	158;34 (21.5%; 3.3-68.7)	304;105 (34.5%; 10.4-70.5)	369;176 (47.7%; 21.3-75.4)	-
Southern	*Staphylococcus aureus*	Trimethoprim/Sulfamethoxazole	Blood/CSF	8;4	26;20	39;31 (79.5%; 75.2-83.2)	1;0
Southern	*Staphylococcus aureus*	Trimethoprim/Sulfamethoxazole	Others	315;178 (56.5%; 34.7-76.1)	312;181 (58%; 18.8-89.2)	308;186 (60.4%; 44.3-74.5)	-
Southern	*Streptococcus agalactiae*	Fluoroquinolones	Blood/CSF	1;0	1;0	-	-
Southern	*Streptococcus agalactiae*	Fluoroquinolones	Others	-	2;1	3;2	-
Southern	*Streptococcus agalactiae*	Macrolides	Others	-	2;0	2;0	-
Southern	*Streptococcus agalactiae*	Penicillins	Blood/CSF	1;0	2;1	-	-
Southern	*Streptococcus agalactiae*	Penicillins	Others	1;0	7;2	4;1	-

Africa GBD# Region	Pathogen	Antimicrobial agent/class	Specimen source	2016 N; R(%R; 95%CI)	2017 N; R(%R; 95%CI)	2018 N; R(%R; 95%CI)	2019 N; R(%R; 95%CI)
Southern	*Streptococcus pneumoniae*	Carbapenems	Blood/CSF	2;0	-	-	-
Southern	*Streptococcus pneumoniae*	Carbapenems	Others	-	-	1;1	-
Southern	*Streptococcus pneumoniae*	Cephalosporins (3rd generation)	Blood/CSF	5;0	2;0	1;0	-
Southern	*Streptococcus pneumoniae*	Cephalosporins (3rd generation)	Others	-	2;0	1;1	-
Southern	*Streptococcus pneumoniae*	Fluoroquinolones	Blood/CSF	2;0	1;0	1;0	-
Southern	*Streptococcus pneumoniae*	Fluoroquinolones	Others	-	4;0	2;1	-
Southern	*Streptococcus pneumoniae*	Macrolides	Blood/CSF	5;0	2;0	1;1	-
Southern	*Streptococcus pneumoniae*	Macrolides	Others	-	6;1	2;1	-
Southern	*Streptococcus pneumoniae*	Penicillins	Blood/CSF	4;0	3;2	3;2	-
Southern	*Streptococcus pneumoniae*	Penicillins	Others	-	4;2	1;1	-
Southern	*Streptococcus pneumoniae*	Trimethoprim/Sulfamethoxazole	Blood/CSF	1;0	1;1	1;1	-
Southern	*Streptococcus pneumoniae*	Trimethoprim/Sulfamethoxazole	Others	-	3;3	-	-
Southern	*Streptococcus pyogenes*	Macrolides	Blood/CSF	1;0	-	-	-
Southern	*Streptococcus pyogenes*	Macrolides	Others	9;3	17;8	14;4	-
Western	*Acinetobacter baumannii*	Aminoglycosides	Blood/CSF	5;2	33;14 (42.4%; 27.8-58.5)	53;19 (35.8%; 31.4-40.6)	34;15 (44.1%; 15.2-77.6)
Western	*Acinetobacter baumannii*	Aminoglycosides	Others	3;1	46;14 (30.4%; 16-50.1)	261;99 (37.9%; 27.1-50.1)	139;78 (56.1%; 47-64.9)
Western	*Acinetobacter baumannii*	Beta-lactam combinations (Antipseudomonal)	Blood/CSF	1;0	11;8	27;12	14;5
Western	*Acinetobacter baumannii*	Beta-lactam combinations (Antipseudomonal)	Others	3;1	9;6	68;34 (50%; 20.5-79.5)	54;32 (59.3%; 56.6-61.9)
Western	*Acinetobacter baumannii*	Carbapenems	Blood/CSF	3;0	17;10	34;15 (44.1%; 15.7-77)	30;7 (23.3%; 1.6-85.4)
Western	*Acinetobacter baumannii*	Carbapenems	Others	3;1	30;11 (36.7%; 14.9-65.6)	168;51 (30.4%; 25.9-35.2)	118;34 (28.8%; 18.7-41.6)
Western	*Acinetobacter baumannii*	Cephalosporins (3rd generation)	Blood/CSF	5;4	33;27 (81.8%; 37.8-97.1)	51;36 (70.6%; 33.1-92.1)	28;20

Africa GBD# Region	Pathogen	Antimicrobial agent/class	Specimen source	2016 N; R(%R; 95%CI)	2017 N; R(%R; 95%CI)	2018 N; R(%R; 95%CI)	2019 N; R(%R; 95%CI)
Western	*Acinetobacter baumannii*	Cephalosporins (3rd generation)	Others	2;1	47;38 (80.9%; 72.8-87)	213;151 (70.9%; 66.1-75.3)	122;81 (66.4%; 64.4-68.3)
Western	*Acinetobacter baumannii*	Cephalosporins (4th generation)	Blood/CSF	2;1	23;17	18;6	8;5
Western	*Acinetobacter baumannii*	Cephalosporins (4th generation)	Others	3;1	14;9	76;48 (63.2%; 49.3-75.1)	19;13
Western	*Acinetobacter baumannii*	Fluoroquinolones	Blood/CSF	5;1	35;11 (31.4%; 4.7-81)	47;8 (17%; 2.4-63)	30;8 (26.7%; 4.1-75.8)
Western	*Acinetobacter baumannii*	Fluoroquinolones	Others	3;2	37;21 (56.8%; 33.6-77.3)	223;104 (46.6%; 44.8-48.5)	132;66 (50%; 44.5-55.5)
Western	*Citrobacter*	Aminoglycosides	Blood/CSF	20;15	71;32 (45.1%; 41.7-48.5)	55;19 (34.5%; 28.8-40.8)	36;19 (52.8%; 51.7-53.8)
Western	*Citrobacter*	Aminoglycosides	Others	50;17 (34%; 12.6-64.7)	192;65 (33.9%; 14.7-60.3)	319;106 (33.2%; 23.4-44.8)	238;89 (37.4%; 26.1-50.3)
Western	*Citrobacter*	Beta-lactam combinations (Antipseudomonal)	Blood/CSF	-	12;8	11;5	15;7
Western	*Citrobacter*	Beta-lactam combinations (Antipseudomonal)	Others	6;1	15;9	65;41 (63.1%; 55.1-70.4)	62;38 (61.3%; 60.3-62.3)
Western	*Citrobacter*	Carbapenems	Blood/CSF	5;4	19;13	16;6	16;4
Western	*Citrobacter*	Carbapenems	Others	8;3	86;10 (11.6%; 1.9-47.1)	195;26 (13.3%; 5.1-30.4)	161;13 (8.1%; 4.2-15)
Western	*Citrobacter*	Cephalosporins (3rd generation)	Blood/CSF	20;12	63;49 (77.8%; 57-90.2)	54;36 (66.7%; 45.5-82.8)	37;24 (64.9%; 52.4-75.6)
Western	*Citrobacter*	Cephalosporins (3rd generation)	Others	45;27 (60%; 35.6-80.3)	187;82 (43.9%; 39-48.9)	343;166 (48.4%; 41.3-55.5)	239;119 (49.8%; 28.7-70.9)
Western	*Citrobacter*	Cephalosporins (4th generation)	Blood/CSF	1;1	3;1	6;2	11;5
Western	*Citrobacter*	Cephalosporins (4th generation)	Others	-	41;16 (39%; 17.6-65.7)	78;33 (42.3%; 31.9-53.4)	41;17 (41.5%; 29.8-54.1)
Western	*Citrobacter*	Fluoroquinolones	Blood/CSF	20;11	70;25 (35.7%; 24.3-49.1)	51;8 (15.7%; 8.2-28.1)	30;14 (46.7%; 31.3-62.7)
Western	*Citrobacter*	Fluoroquinolones	Others	49;23 (46.9%; 22.6-72.8)	135;60 (44.4%; 28.7-61.4)	271;112 (41.3%; 35.4-47.5)	198;84 (42.4%; 32.8-52.7)
Western	*Enterobacter*	Aminoglycosides	Blood/CSF	45;21 (46.7%; 27.2-67.2)	135;60 (44.4%; 31.6-58)	99;53 (53.5%; 36-70.2)	71;30 (42.3%; 28.9-56.9)
Western	*Enterobacter*	Aminoglycosides	Others	35;12 (34.3%; 23-47.7)	304;127 (41.8%; 31.3-53)	482;163 (33.8%; 30.5-37.3)	363;145 (39.9%; 38.5-41.4)
Western	*Enterobacter*	Beta-lactam combinations (Antipseudomonal)	Blood/CSF	6;2	14;8	18;10	21;10
Western	*Enterobacter*	Beta-lactam combinations (Antipseudomonal)	Others	7;4	49;20 (40.8%; 28.2-54.8)	88;42 (47.7%; 25.8-70.5)	67;42 (62.7%; 57.1-68)

Africa GBD# Region	Pathogen	Antimicrobial agent/class	Specimen source	2016 N; R(%R; 95%CI)	2017 N; R(%R; 95%CI)	2018 N; R(%R; 95%CI)	2019 N; R(%R; 95%CI)
Western	*Enterobacter*	Carbapenems	Blood/CSF	11;2	88;42 (47.7%; 6.6-92.2)	57;18 (31.6%; 7.1-73.5)	54;14 (25.9%; 4.6-71.7)
Western	*Enterobacter*	Carbapenems	Others	13;1	195;11 (5.6%; 3.8-8.2)	317;43 (13.6%; 5.7-28.8)	266;24 (9%; 3.8-19.8)
Western	*Enterobacter*	Cephalosporins (4th generation)	Blood/CSF	9;5	25;16	35;26 (74.3%; 14-98.1)	35;23 (65.7%; 57.6-73)
Western	*Enterobacter*	Cephalosporins (4th generation)	Others	-	70;26 (37.1%; 13.5-69.1)	95;52 (54.7%; 46.3-63)	61;23 (37.7%; 23.5-54.4)
Western	*Enterobacter*	Fluoroquinolones	Blood/CSF	45;20 (44.4%; 27.4-62.9)	127;46 (36.2%; 19.8-56.7)	91;49 (53.8%; 36.5-70.3)	64;26 (40.6%; 28.5-54)
Western	*Enterobacter*	Fluoroquinolones	Others	31;14 (45.2%; 28.7-62.7)	276;121 (43.8%; 41-46.7)	484;186 (38.4%; 31.6-45.7)	350;156 (44.6%; 38.9-50.4)
Western	*Enterococcus*	Aminopenicillins	Blood/CSF	1;0	11;4	16;7	3;1
Western	*Enterococcus*	Aminopenicillins	Others	7;5	23;16	51;15 (29.4%; 16.9-46)	17;6
Western	*Enterococcus faecalis*	Fluoroquinolones	Blood/CSF	1;0	12;9	14;11	2;1
Western	*Enterococcus faecalis*	Fluoroquinolones	Others	6;4	30;11 (36.7%; 29.4-44.7)	91;27 (29.7%; 24.5-35.5)	6;3
Western	*Enterococcus faecalis*	Vancomycin	Blood/CSF	1;0	6;1	14;1	2;1
Western	*Enterococcus faecalis*	Vancomycin	Others	1;0	16;5	24;3	8;1
Western	*Enterococcus faecium*	Fluoroquinolones	Blood/CSF	-	-	-	1;1
Western	*Enterococcus faecium*	Fluoroquinolones	Others	-	2;1	2;1	1;1
Western	*Enterococcus faecium*	Vancomycin	Blood/CSF	-	-	1;0	1;0
Western	*Enterococcus faecium*	Vancomycin	Others	-	2;0	-	-
Western	*Escherichia coli*	Aminoglycosides	Blood/CSF	282;97 (34.4%; 23-48)	426;149 (35%; 31.8-38.3)	324;117 (36.1%; 26.5-47)	145;44 (30.3%; 17.4-47.5)
Western	*Escherichia coli*	Aminoglycosides	Others	1482;524 (35.4%; 29.1-42.2)	4028;1360 (33.8%; 28-40.1)	6441;2134 (33.1%; 28.1-38.6)	2997;1025 (34.2%; 31.5-37.1)
Western	*Escherichia coli*	Aminopenicillins	Blood/CSF	272;181 (66.5%; 62.9-70)	393;228 (58%; 34.8-78.1)	308;222 (72.1%; 60.4-81.4)	137;105 (76.6%; 59.1-88.2)
Western	*Escherichia coli*	Aminopenicillins	Others	1508;1196 (79.3%; 75.2-82.9)	3941;3225 (81.8%; 72.3-88.6)	6601;5416 (82%; 77.1-86.1)	3153;2615 (82.9%; 76-88.2)
Western	*Escherichia coli*	Carbapenems	Blood/CSF	21;8	132;27 (20.5%; 6.5-48.9)	148;17 (11.5%; 2.8-36.7)	82;10 (12.2%; 9.3-15.8)
Western	*Escherichia coli*	Carbapenems	Others	259;41 (15.8%; 3.3-51.2)	2022;111 (5.5%; 1.2-21.1)	3657;226 (6.2%; 4.2-9)	1980;128 (6.5%; 3.1-12.8)

Africa GBD# Region	Pathogen	Antimicrobial agent/class	Specimen source	2016 N; R(%R; 95%CI)	2017 N; R(%R; 95%CI)	2018 N; R(%R; 95%CI)	2019 N; R(%R; 95%CI)
Western	*Escherichia coli*	Cephalosporins (3rd generation)	Blood/CSF	177;114 (64.4%; 46.8-78.8)	336;207 (61.6%; 55.3-67.5)	311;197 (63.3%; 53.6-72.1)	141;74 (52.5%; 49.2-55.7)
Western	*Escherichia coli*	Cephalosporins (3rd generation)	Others	1412;825 (58.4%; 50.3-66.1)	3859;1698 (44%; 32.7-55.9)	6793;3226 (47.5%; 41.2-53.9)	3201;1465 (45.8%; 35.4-56.5)
Western	*Escherichia coli*	Fluoroquinolones	Blood/CSF	260;82 (31.5%; 15.9-52.9)	398;143 (35.9%; 28.3-44.3)	284;119 (41.9%; 31.9-52.6)	115;49 (42.6%; 21.9-66.3)
Western	*Escherichia coli*	Fluoroquinolones	Others	1616;671 (41.5%; 31.9-51.8)	3500;1697 (48.5%; 40.8-56.2)	6265;3382 (54%; 41.1-66.4)	2830;1481 (52.3%; 48.1-56.6)
Western	*Escherichia coli*	Trimethoprim/Sulfamethoxazole	Blood/CSF	116;57 (49.1%; 48.2-50.1)	142;56 (39.4%; 19.8-63.2)	75;44 (58.7%; 29.8-82.6)	58;34 (58.6%; 53.1-64)
Western	*Escherichia coli*	Trimethoprim/Sulfamethoxazole	Others	452;311 (68.8%; 57.3-78.4)	982;705 (71.8%; 60.5-80.8)	2750;2240 (81.5%; 74.3-87)	978;766 (78.3%; 65.4-87.3)
Western	*Haemophilus influenzae*	Aminopenicillins	Blood/CSF	-	-	2;1	-
Western	*Haemophilus influenzae*	Aminopenicillins	Others	1;1	4;3	3;3	1;1
Western	*Haemophilus influenzae*	Cephalosporins (3rd generation)	Blood/CSF	-	1;0	2;1	-
Western	*Haemophilus influenzae*	Cephalosporins (3rd generation)	Others	1;1	6;2	3;3	1;0
Western	*Klebsiella pneumoniae*	Aminoglycosides	Blood/CSF	90;74 (82.2%; 79.1-85)	427;263 (61.6%; 55.7-67.2)	287;144 (50.2%; 32-68.3)	191;91 (47.6%; 45.6-49.7)
Western	*Klebsiella pneumoniae*	Aminoglycosides	Others	172;68 (39.5%; 33.3-46.1)	1630;684 (42%; 36.3-47.9)	2066;735 (35.6%; 32.9-38.4)	1304;519 (39.8%; 31.4-48.9)
Western	*Klebsiella pneumoniae*	Carbapenems	Blood/CSF	47;9 (19.1%; 3.7-59.3)	229;56 (24.5%; 19.7-29.9)	181;28 (15.5%; 7.3-30)	113;19 (16.8%; 2.1-65.3)
Western	*Klebsiella pneumoniae*	Carbapenems	Others	108;10 (9.3%; 5-16.6)	974;78 (8%; 2.4-23.4)	1435;129 (9%; 5.3-14.9)	924;58 (6.3%; 3.1-12.3)
Western	*Klebsiella pneumoniae*	Cephalosporins (3rd generation)	Blood/CSF	83;71 (85.5%; 77.5-91)	426;321 (75.4%; 71.8-78.6)	272;233 (85.7%; 81.4-89.1)	181;145 (80.1%; 71.5-86.6)
Western	*Klebsiella pneumoniae*	Cephalosporins (3rd generation)	Others	198;109 (55.1%; 45.6-64.2)	1618;859 (53.1%; 46.8-59.3)	2287;1324 (57.9%; 49.3-66)	1330;705 (53%; 41.4-64.3)
Western	*Klebsiella pneumoniae*	Fluoroquinolones	Blood/CSF	88;37 (42%; 15.3-74.5)	408;202 (49.5%; 41.9-57.2)	252;105 (41.7%; 32.7-51.3)	142;58 (40.8%; 19.9-65.7)
Western	*Klebsiella pneumoniae*	Fluoroquinolones	Others	166;81 (48.8%; 41.2-56.4)	1422;639 (44.9%; 38.2-51.9)	1907;890 (46.7%; 42.7-50.7)	1174;507 (43.2%; 42.1-44.2)
Western	*Morganella*	Cephalosporins (3rd generation)	Blood/CSF	2;1	1;0	4;2	1;1
Western	*Morganella*	Cephalosporins (3rd generation)	Others	2;1	41;12 (29.3%; 10.4-59.7)	59;22 (37.3%; 22.7-54.6)	56;20 (35.7%; 15.2-63.3)
Western	*Morganella*	Cephalosporins (4th generation)	Blood/CSF	-	-	-	1;1

Africa GBD# Region	Pathogen	Antimicrobial agent/class	Specimen source	2016 N; R(%R; 95%CI)	2017 N; R(%R; 95%CI)	2018 N; R(%R; 95%CI)	2019 N; R(%R; 95%CI)
Western	*Morganella*	Cephalosporins (4th generation)	Others	-	9;0	11;3	9;5
Western	*Morganella*	Fluoroquinolones	Blood/CSF	2;0	1;0	4;2	-
Western	*Morganella*	Fluoroquinolones	Others	2;2	36;17 (47.2%; 40-54.5)	46;28 (60.9%; 41.4-77.4)	56;36 (64.3%; 32.2-87.2)
Western	*Neisseria gonorrhoeae*	Cephalosporins (3rd generation)	Blood/CSF	2;1	1;1	1;0	-
Western	*Neisseria gonorrhoeae*	Cephalosporins (3rd generation)	Others	3;1	52;16 (30.8%; 22.7-40.2)	46;20 (43.5%; 40.4-46.6)	44;12 (27.3%; 22.3-32.9)
Western	*Neisseria gonorrhoeae*	Fluoroquinolones	Blood/CSF	3;1	2;2	1;0	-
Western	*Neisseria gonorrhoeae*	Fluoroquinolones	Others	8;1	62;22 (35.5%; 23.6-49.5)	36;21 (58.3%; 46.6-69.2)	27;9
Western	*Neisseria gonorrhoeae*	Macrolides	Others	3;2	40;18 (45%; 25.6-66)	24;7	10;2
Western	*Neisseria gonorrhoeae*	Quinolones	Others	3;1	13;8	11;8	9;7
Western	*Neisseria gonorrhoeae*	Tetracyclines	Blood/CSF	3;1	2;1	-	-
Western	*Neisseria gonorrhoeae*	Tetracyclines	Others	3;2	34;15 (44.1%; 24.7-65.5)	14;8	13;7
Western	*Non-typhoidal Salmonella*	Fluoroquinolones	Blood/CSF	6;4	52;5 (9.6%; 4.5-19.3)	25;1	14;2
Western	*Non-typhoidal Salmonella*	Fluoroquinolones	Others	90;30 (33.3%; 29.8-37.1)	110;17 (15.5%; 7.6-29)	193;50 (25.9%; 16.3-38.5)	78;23 (29.5%; 18.8-43.1)
Western	*Proteus*	Aminoglycosides	Blood/CSF	46;13 (28.3%; 18.6-40.5)	63;19 (30.2%; 27.1-33.5)	37;10 (27%; 15.9-42)	9;3
Western	*Proteus*	Aminoglycosides	Others	183;69 (37.7%; 36-39.5)	405;129 (31.9%; 29.7-34.1)	577;128 (22.2%; 16.3-29.4)	326;103 (31.6%; 18.7-48.1)
Western	*Proteus*	Aminopenicillins	Blood/CSF	45;35 (77.8%; 60.5-88.9)	63;33 (52.4%; 18.2-84.5)	34;28 (82.4%; 45.6-96.3)	10;7
Western	*Proteus*	Aminopenicillins	Others	184;143 (77.7%; 62-88.2)	377;248 (65.8%; 58.7-72.2)	531;333 (62.7%; 52.2-72.2)	301;209 (69.4%; 63.6-74.7)
Western	*Proteus*	Cephalosporins (3rd generation)	Blood/CSF	20;15	36;21 (58.3%; 34.7-78.7)	35;18 (51.4%; 27.9-74.4)	10;7
Western	*Proteus*	Cephalosporins (3rd generation)	Others	182;96 (52.7%; 37.1-67.9)	400;152 (38%; 22.5-56.5)	607;205 (33.8%; 23.7-45.6)	323;108 (33.4%; 18-53.4)
Western	*Proteus*	Cephalosporins (4th generation)	Blood/CSF	-	2;0	3;1	-
Western	*Proteus*	Cephalosporins (4th generation)	Others	5;3	52;6 (11.5%; 1-62.1)	79;33 (41.8%; 20.3-66.9)	43;12 (27.9%; 23.3-33.1)
Western	*Proteus*	Fluoroquinolones	Blood/CSF	46;11 (23.9%; 14.5-36.9)	63;15 (23.8%; 15.8-34.1)	36;13 (36.1%; 31.4-41.1)	9;4

Africa GBD# Region	Pathogen	Antimicrobial agent/class	Specimen source	2016 N; R(%R; 95%CI)	2017 N; R(%R; 95%CI)	2018 N; R(%R; 95%CI)	2019 N; R(%R; 95%CI)
Western	*Proteus*	Fluoroquinolones	Others	195;75 (38.5%; 31.9-45.4)	373;128 (34.3%; 33.6-35.1)	594;157 (26.4%; 19.2-35.3)	302;107 (35.4%; 30-41.2)
Western	*Pseudomonas aeruginosa*	Aminoglycosides	Blood/CSF	60;19 (31.7%; 24.2-40.2)	115;56 (48.7%; 38.3-59.2)	72;27 (37.5%; 30.5-45.1)	24;6
Western	*Pseudomonas aeruginosa*	Aminoglycosides	Others	141;55 (39%; 28.1-51.1)	642;249 (38.8%; 34.8-42.9)	762;311 (40.8%; 29.8-52.8)	483;214 (44.3%; 36.6-52.2)
Western	*Pseudomonas aeruginosa*	Beta-lactam combinations (Antipseudomonal)	Blood/CSF	10;7	52;10 (19.2%; 6.6-44.7)	33;10 (30.3%; 8.4-67.3)	7;3
Western	*Pseudomonas aeruginosa*	Beta-lactam combinations (Antipseudomonal)	Others	30;5 (16.7%; 8.5-30.2)	187;51 (27.3%; 15.4-43.6)	320;123 (38.4%; 18.4-63.4)	160;73 (45.6%; 15.4-79.5)
Western	*Pseudomonas aeruginosa*	Carbapenems	Blood/CSF	21;6	71;28 (39.4%; 30.1-49.6)	33;14 (42.4%; 14.6-76.1)	8;4
Western	*Pseudomonas aeruginosa*	Carbapenems	Others	55;32 (58.2%; 53-63.2)	516;101 (19.6%; 8.6-38.7)	539;122 (22.6%; 14-34.4)	369;59 (16%; 5-40.7)
Western	*Pseudomonas aeruginosa*	Cephalosporins (3rd generation)	Blood/CSF	59;41 (69.5%; 64.9-73.7)	113;63 (55.8%; 39.6-70.7)	60;36 (60%; 41.2-76.3)	20;12
Western	*Pseudomonas aeruginosa*	Cephalosporins (3rd generation)	Others	155;105 (67.7%; 54.9-78.4)	653;371 (56.8%; 49.7-63.7)	705;408 (57.9%; 49.8-65.5)	425;266 (62.6%; 57.1-67.8)
Western	*Pseudomonas aeruginosa*	Cephalosporins (4th generation)	Blood/CSF	9;6	37;9 (24.3%; 11.7-43.7)	18;9	2;2
Western	*Pseudomonas aeruginosa*	Cephalosporins (4th generation)	Others	24;10	115;25 (21.7%; 14.3-31.7)	241;98 (40.7%; 32.3-49.6)	88;45 (51.1%; 44.2-58)
Western	*Pseudomonas aeruginosa*	Fluoroquinolones	Blood/CSF	31;11 (35.5%; 9.4-74.4)	108;48 (44.4%; 34.7-54.6)	58;23 (39.7%; 34.6-44.9)	21;6
Western	*Pseudomonas aeruginosa*	Fluoroquinolones	Others	150;66 (44%; 26.8-62.7)	630;232 (36.8%; 28.5-46)	717;274 (38.2%; 25.6-52.6)	429;144 (33.6%; 20.3-50.1)
Western	*Salmonella Paratyphi*	Fluoroquinolones	Blood/CSF	-	-	1;1	1;0
Western	*Salmonella Paratyphi*	Fluoroquinolones	Others	1;0	3;1	5;0	3;1
Western	*Salmonella Typhi*	Fluoroquinolones	Blood/CSF	8;2	20;0	16;0	2;1
Western	*Salmonella Typhi*	Fluoroquinolones	Others	1;0	8;2	5;1	4;0
Western	*Serratia*	Aminoglycosides	Blood/CSF	3;1	10;2	30;13 (43.3%; 26.3-62)	12;6
Western	*Serratia*	Aminoglycosides	Others	1;1	87;18 (20.7%; 16.9-25.1)	105;33 (31.4%; 25.8-37.6)	88;27 (30.7%; 10.4-62.7)
Western	*Serratia*	Carbapenems	Blood/CSF	2;0	4;1	19;5	6;1
Western	*Serratia*	Carbapenems	Others	-	35;5 (14.3%; 7.6-25.2)	70;21 (30%; 9.2-64.4)	53;9 (17%; 5.7-40.7)

Africa GBD# Region	Pathogen	Antimicrobial agent/class	Specimen source	2016 N; R(%R; 95%CI)	2017 N; R(%R; 95%CI)	2018 N; R(%R; 95%CI)	2019 N; R(%R; 95%CI)
Western	*Serratia*	Cephalosporins (3rd generation)	Blood/CSF	3;2	10;4	27;21	13;7
Western	*Serratia*	Cephalosporins (3rd generation)	Others	1;1	84;40 (47.6%; 34-61.7)	125;73 (58.4%; 41-74)	85;45 (52.9%; 41.8-63.8)
Western	*Serratia*	Cephalosporins (4th generation)	Blood/CSF	1;0	1;1	12;9	4;2
Western	*Serratia*	Cephalosporins (4th generation)	Others	-	12;4	27;18	7;1
Western	*Serratia*	Fluoroquinolones	Blood/CSF	3;1	6;4	25;4	10;4
Western	*Serratia*	Fluoroquinolones	Others	1;1	59;13 (22%; 15.3-30.6)	105;46 (43.8%; 32.1-56.2)	80;28 (35%; 13.8-64.4)
Western	*Shigella*	Fluoroquinolones	Blood/CSF	-	4;1	4;1	2;1
Western	*Shigella*	Fluoroquinolones	Others	15;5	36;5 (13.9%; 9.1-20.6)	130;43 (33.1%; 29.2-37.2)	32;9 (28.1%; 21.4-36)
Western	*Staphylococcus aureus*	Beta-lactam combinations (Antipseudomonal)	Blood/CSF	4;3	14;6	7;3	13;6
Western	*Staphylococcus aureus*	Beta-lactam combinations (Antipseudomonal)	Others	2;1	51;18 (35.3%; 22.5-50.6)	55;21 (38.2%; 6.8-83.8)	6;2
Western	*Staphylococcus aureus*	Fluoroquinolones	Blood/CSF	420;164 (39%; 37.5-40.6)	1078;399 (37%; 32.6-41.7)	588;227 (38.6%; 32.8-44.8)	290;99 (34.1%; 28.4-40.4)
Western	*Staphylococcus aureus*	Fluoroquinolones	Others	984;357 (36.3%; 33.2-39.5)	2023;787 (38.9%; 32.2-46.1)	2779;893 (32.1%; 25.6-39.4)	1106;388 (35.1%; 23-49.4)
Western	*Staphylococcus aureus*	Macrolides	Blood/CSF	401;209 (52.1%; 47.6-56.6)	1183;609 (51.5%; 47-56)	514;238 (46.3%; 42.2-50.4)	293;128 (43.7%; 37.3-50.3)
Western	*Staphylococcus aureus*	Macrolides	Others	693;306 (44.2%; 38.3-50.2)	1829;741 (40.5%; 30.6-51.3)	2178;960 (44.1%; 36.9-51.5)	872;344 (39.4%; 23-58.7)
Western	*Staphylococcus aureus*	Methicillin	Blood/CSF	169;100 (59.2%; 41.5-74.8)	828;452 (54.6%; 37.8-70.4)	330;190 (57.6%; 45.7-68.6)	231;138 (59.7%; 47.6-70.8)
Western	*Staphylococcus aureus*	Methicillin	Others	368;254 (69%; 56.3-79.4)	1239;585 (47.2%; 36.3-58.5)	1832;857 (46.8%; 35.4-58.5)	931;459 (49.3%; 37.6-61.1)
Western	*Staphylococcus aureus*	Trimethoprim/Sulfamethoxazole	Blood/CSF	104;83 (79.8%; 56.5-92.3)	207;152 (73.4%; 60.2-83.4)	162;110 (67.9%; 49.6-82)	96;70 (72.9%; 68.5-76.9)
Western	*Staphylococcus aureus*	Trimethoprim/Sulfamethoxazole	Others	289;205 (70.9%; 68.5-73.3)	389;276 (71%; 48.1-86.6)	849;521 (61.4%; 38.6-80)	385;218 (56.6%; 40.5-71.5)
Western	*Streptococcus agalactiae*	Fluoroquinolones	Blood/CSF	-	1;1	4;2	2;0
Western	*Streptococcus agalactiae*	Fluoroquinolones	Others	-	6;2	19;3	-
Western	*Streptococcus agalactiae*	Macrolides	Blood/CSF	-	1;0	3;0	2;0

Africa GBD# Region	Pathogen	Antimicrobial agent/class	Specimen source	2016 N; R(%R; 95%CI)	2017 N; R(%R; 95%CI)	2018 N; R(%R; 95%CI)	2019 N; R(%R; 95%CI)
Western	*Streptococcus agalactiae*	Macrolides	Others	-	6;3	14;7	-
Western	*Streptococcus agalactiae*	Penicillins	Blood/CSF	-	-	5;4	2;0
Western	*Streptococcus agalactiae*	Penicillins	Others	-	8;7	18;4	1;0
Western	*Streptococcus pneumoniae*	Carbapenems	Blood/CSF	2;1	8;3	1;1	7;2
Western	*Streptococcus pneumoniae*	Carbapenems	Others	2;2	8;2	5;1	2;2
Western	*Streptococcus pneumoniae*	Cephalosporins (3rd generation)	Blood/CSF	9;3	19;6	5;2	12;0
Western	*Streptococcus pneumoniae*	Cephalosporins (3rd generation)	Others	14;11	69;49 (71%; 50.3-85.6)	12;5	5;3
Western	*Streptococcus pneumoniae*	Fluoroquinolones	Blood/CSF	7;3	21;7	8;1	13;1
Western	*Streptococcus pneumoniae*	Fluoroquinolones	Others	15;2	73;35 (47.9%; 31-65.3)	17;8	8;3
Western	*Streptococcus pneumoniae*	Macrolides	Blood/CSF	8;2	17;7	12;3	9;2
Western	*Streptococcus pneumoniae*	Macrolides	Others	15;4	70;44 (62.9%; 51.5-72.9)	22;11	10;4
Western	*Streptococcus pneumoniae*	Penicillins	Blood/CSF	4;4	15;10	10;10	17;6
Western	*Streptococcus pneumoniae*	Penicillins	Others	7;5	30;22 (73.3%; 50.6-88.1)	15;9	9;4
Western	*Streptococcus pneumoniae*	Trimethoprim/Sulfamethoxazole	Blood/CSF	2;2	3;3	2;1	1;0
Western	*Streptococcus pneumoniae*	Trimethoprim/Sulfamethoxazole	Others	9;9	9;8	8;7	3;3
Western	*Streptococcus pyogenes*	Macrolides	Blood/CSF	-	1;0	2;0	1;1
Western	*Streptococcus pyogenes*	Macrolides	Others	1;1	23;9	14;9	4;0

#GBD=Global burden of disease; N = number of tested isolates; R = resistant isolates; %R and 95%CI are shown only if ≥30 isolates/ year; — information not available. Regions (**Central** - Gabon; **Eastern** - Malawi, Kenya, Uganda, Tanzania and Zambia; **Southern** - Eswatini and Zimbabwe; **Western** – Burkina Faso, Cameroon, Ghana, Nigeria, Senegal, and Sierra Leone).

.
