# Supplementary material for: Antimicrobial resistance in Africa: A retrospective analysis of data from 14 countries, 2016–2019
Source: PLoS Med. 2025 Jun 24;22(6):e1004638. doi: 10.1371/journal.pmed.1004638 (PMC12186946; doi:10.1371/journal.pmed.1004638)
Supplement: S12 Table — (PDF) [file pmed.1004638.s014.pdf]

S12 Table: Antimicrobial resistance prevalence of all bacterial isolates by region, patient department, and specimen source.

Variable		Resistant isolates	Total isolates	%R	Tukey-Kramer Test P-value
Region	Central	11532	33,127	34.8	
	Eastern	90786	213,371	42.5	
	Southern	10163	29,947	33.9	
	Western	108889	268,915	40.5	
Department	Inpatient	47427	104,717	45.3	
	Outpatient	56168	148,092	37.9	
Specimen source	Blood and CSF	31032	74,263	41.8	
	Others	190338	471,097	40.4	

CSF, cerebrospinal fluid

Note: Countries are categorized into regions based on the Global Burden of Disease classification (Central—Gabon; Eastern—Malawi, Kenya, Uganda, Tanzania, and Zambia; Southern—Eswatini and Zimbabwe; Western—Burkina Faso, Cameroon, Ghana, Nigeria, Senegal, and Sierra Leone)
