# Supplementary material for: Antimicrobial resistance in Africa: A retrospective analysis of data from 14 countries, 2016–2019
Source: PLoS Med. 2025 Jun 24;22(6):e1004638. doi: 10.1371/journal.pmed.1004638 (PMC12186946; doi:10.1371/journal.pmed.1004638)

S1 Figure: Drug Resistance Index for 11 African countries, 2016–2019

a) Frequency of use represents the proportion of each antibiotic class consumed. b) Resistance reflects the proportion of the resistant isolates for each antibiotic class. c) Estimated Drug Resistance Index.

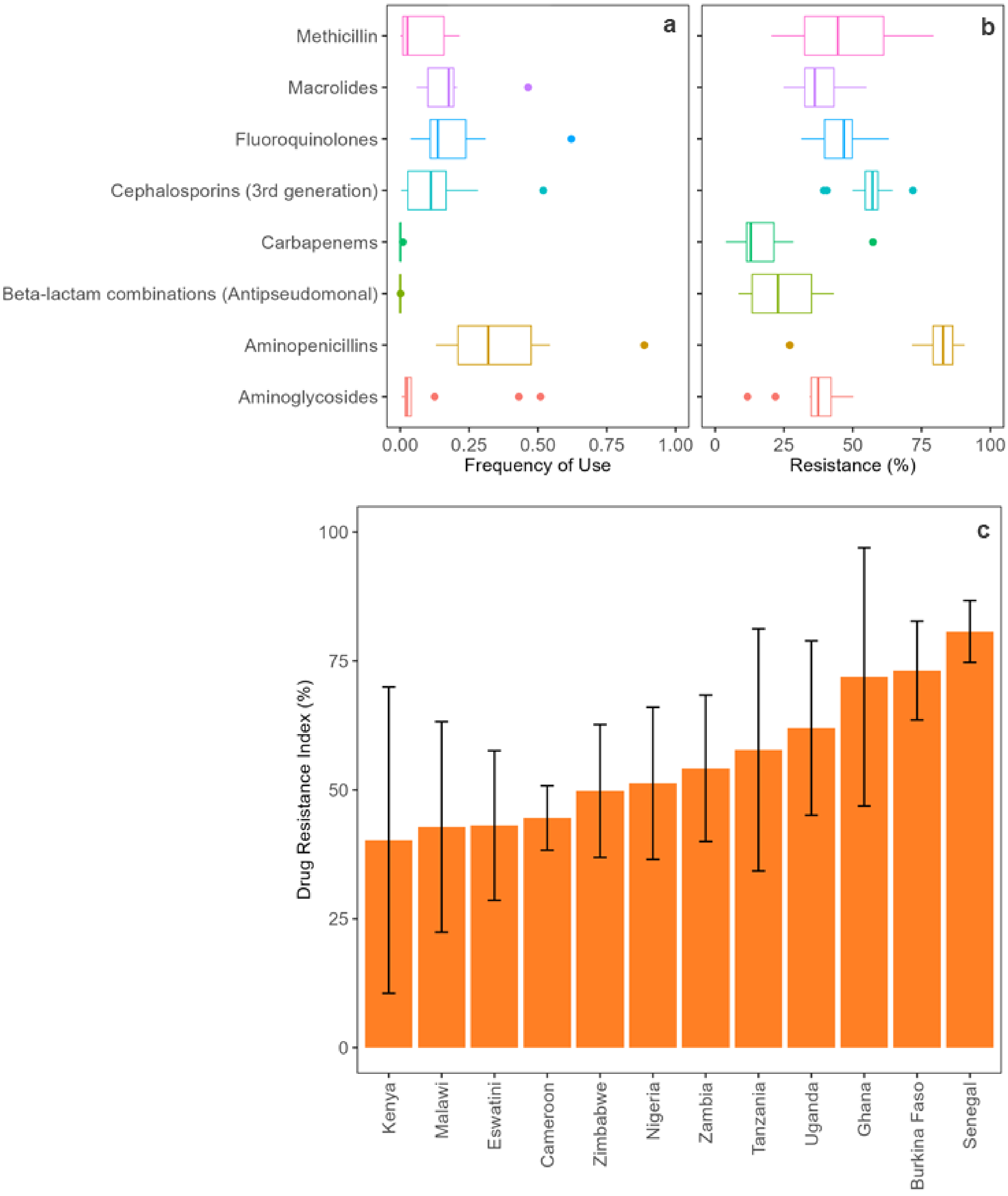

Supplement: S1 Fig — (PDF) [file pmed.1004638.s015.pdf]
